# Supplementary material for: Computational study of potential inhibitors for fat mass and obesity-associated protein from seaweed and plant compounds
Source: PeerJ. 2022 Oct 21;10:e14256. doi: 10.7717/peerj.14256 (PMC9590420; doi:10.7717/peerj.14256)
Supplement: Supplemental Information 1 — 100 anti-obesity plant compounds. [file peerj-10-14256-s001.docx]

| Compound ID | Compound name | Plant Source |
| --- | --- | --- |
| Comp1 | (9) Methyl(E)-11-methoxy9-oxononadec-10-enoate | *Hibiscus rosa-sinensis* |
| Comp2 | Quercetin | *Tridax procumbens* |
| Comp3 | 8 nonynoic acid | *Lepidium sativum* |
| Comp4 | 9 Decynoic acid | *Elaeis oleifera* |
| Comp5 | 9,12,15 Octadecatrienoic acid | *Calendula officinalis* |
| Comp6 | Acetogenin | *Annona reticulata* |
| Comp7 | Punicalin | *Punica granatum* |
| Comp8 | Annnomuricin B | [*Annona muricata*](https://www.ncbi.nlm.nih.gov/pmc/articles/PMC4519917/) |
| Comp9 | Annohexocin | *Annona muricata* |
| Comp10 | Annoionol A | *Annona muricata* |
| Comp11 | Annoionol B | *Annona muricata* |
| Comp12 | Annomuricin A | *Annona muricata* |
| Comp13 | Annonacin | [*Annona muricata*](https://en.wikipedia.org/wiki/Annona_muricata) |
| Comp14 | Annonacin A | [*Annona muricata*](https://en.wikipedia.org/wiki/Annona_muricata) |
| Comp15 | Annonacinone | [*Annona muricata*](https://en.wikipedia.org/wiki/Annona_muricata) |
| Comp16 | Annonaine | *Annona reticulata* |
| Comp17 | Annopentocin A | *Annona muricata)* |
| Comp18 | Annoreticuin 9one | [*Annona reticulata*](https://www.sciencedirect.com/science/article/pii/S2225411015000504) |
| Comp19 | Anomurine | *Annona muricata* |
| Comp20 | Quercetin 3,30 diglucoside | *Azadirachta indica* |
| Comp21 | Apigenin | *Petroselinum crispum* |
| Comp22 | Arianacin | *Annona muricata* |
| Comp23 | Ascorbic acid | *Ribes uva-crispa* |
| Comp24 | Asimicin | *Asimina triloba* |
| Comp25 | Asimilobine | [*Nelumbo nucifera*](https://www.sciencedirect.com/science/article/pii/B9780128192122000402) |
| Comp26 | Beta rosasterol | *Brassica napus* |
| Comp27 | Beta sitosterol | *Oryza sativa* |
| Comp28 | Quercetin 3,4' diglucoside | *Allium cepa* |
| Comp29 | Brevifolin | *Punica granatum* |
| Comp30 | Bullaticin | *Annona squamosa* |
| Comp31 | Caffeic acid | *Coffea Arabica* |
| Comp32 | Campesterol | *Brassica campestris* |
| Comp33 | Capsaicin | *Capsicum annuum* |
| Comp34 | Capsiate | *Capsicum annuum* |
| Comp35 | Carnosic acid | *Rosmarinus officinalis* |
| Comp36 | Carnosol | *Salvia officinalis* |
| Comp37 | Catechin | *Camellia sinensis* |
| Comp38 | Cathinone | *Catha edulis* |
| Comp39 | Celastrol | *Tripterygium wilfordii* |
| Comp40 | Quercetin 3,7 diglucoside | *Delonix elata* |
| Comp41 | Resveratrol | [*Vitis vinifera*](https://en.wikipedia.org/wiki/Vitis_vinifera) |
| Comp42 | Chlorogenic acid | *Coffea canephora* |
| Comp43 | Cis- annonacin10one | [*Annona muricata*](https://www.ncbi.nlm.nih.gov/pmc/articles/PMC4519917/) |
| Comp44 | Cis- annoreticuin | *Annona muricata* |
| Comp45 | Cis- goniothalamicin | *Annona muricata* |
| Comp46 | Cis- solamin | *Annona muricata* |
| Comp47 | Cis- uvariamic | *Annona muricata* |
| Comp48 | Cis anthenole | *Foeniculum vulgare* |
| Comp49 | Citroside A | *Epipremnum pinnatum* |
| Comp50 | Corilagin | *Caesalpinia coriaria* |
| Comp51 | Coumaric acid | *Vitis vinifera L.* |
| Comp52 | Curcumin | *Curcuma longa* |
| Comp53 | Cyanidine 3, 5 diglucoside | *Glycine max* |
| Comp54 | Cyanidine-3-Oglucoside | *Glycine max* |
| Comp55 | Daidzein | *Glycine max* |
| Comp56 | Delphinidin-3-Oglucoside | *Glycine max* |
| Comp57 | Punicalagin | *Punica granatum* |
| Comp58 | Undecane | *Elettaria cardamomum* |
| Comp59 | Rutecarpine | *Evodia rutaecarpa* |
| Comp60 | Ellagic acid | *Geranium thunbergii* |
| Comp61 | Epicatechin | *Salacia reticulata* |
| Comp62 | Epigallocatechin | *Salacia reticulata* |
| Comp63 | Evodiamine | *Evodia rutaecarpa* |
| Comp64 | Exemestane | *Brassica oleracea var. italica* |
| Comp65 | Fructan | *Agave angustifolia* |
| Comp66 | Gallocatechin | *Vitis vinifera.* |
| Comp67 | Genistein | *Glycine max* |
| Comp68 | Gymnemic acids | *Gymnema sylvestre* |
| Comp69 | Hexadecanoic acid ethyl ester | [*Pistia stratiotes*](https://www.cabi.org/isc/datasheet/41496) |
| Comp70 | Hydroxycitric acid | *Garcinia cambogia* |
| Comp71 | Isolaureline | *Annona muricata* |
| Comp72 | Kaempherol | *Cosmos caudatus* |
| Comp73 | Vitisin A | *Vitis vinifera* |
| Comp74 | Riboflavin | *Spinacia oleracea* |
| Comp75 | Linoleic acid | *Punica granatum* |
| Comp76 | Luteolin | *Vitis vinifera* |
| Comp77 | Luteolin 4-O-glucopyranoside | *Olea europaea* |
| Comp78 | Malvidin | *Vitis vinifera* |
| Comp79 | Mangiferin | *Mangifera indica* |
| Comp80 | Methyl 8-oxooctadec-9-ynoate | *Hibiscus rosa-sinensis* |
| Comp81 | Methyl Dec-9-ynoate | *Hibiscus rosa-sinensis* |
| Comp82 | Methyl malvalate | *Sterculia foetida* |
| Comp83 | Methyl non-8-ynoate | *Hibiscus rosa-sinensis* |
| Comp84 | Methyl sterculate | *Gaultheria procumbens* |
| Comp85 | Trance anthenole | [*Foeniculum vulgare*](https://www.sciencedirect.com/topics/agricultural-and-biological-sciences/foeniculum-vulgare) |
| Comp86 | Niacin | *Oryza sativa* |
| Comp87 | Thiamine | [*Cucurbita pepo*](https://en.wikipedia.org/wiki/Cucurbita_pepo) |
| Comp88 | Normuciferine | *Annona muricata* |
| Comp89 | Octadecanoic acid ethyl ester | *Sida cordata* |
| Comp90 | Oleic acid2 | *Helianthus annuus* |
| Comp91 | Oleoyl-estrone | *Zingiber officinale* |
| Comp92 | P-synephrine | *Citrus aurantium* |
| Comp93 | Palmitic acid3 | *Elaeis guineensis* |
| Comp94 | Petunidin-3-Ogluco-side | *Glycine max* |
| Comp95 | Stigmast-5-en-3-ol | *Salvadora persica* |
| Comp96 | Phytohemagglutinin | *Phaseolus vulgaris* |
| Comp97 | Platycodins | *Platycodon grandiflorum* |
| Comp98 | Squalene | *Olea europaea* |
| Comp99 | Rutin | *Carpobrotus edulis* |
| Comp100 | Protopanaxadiol | *Panax ginseng* |
