# Supplementary material for: Computational study of potential inhibitors for fat mass and obesity-associated protein from seaweed and plant compounds
Source: PeerJ. 2022 Oct 21;10:e14256. doi: 10.7717/peerj.14256 (PMC9590420; doi:10.7717/peerj.14256)
Supplement: Supplemental Information 2 — Seaweed compounds from Seaweed Metabolite Database (http://swmd.co.in/). [file peerj-10-14256-s002.docx]

| S. No. | Accession Number | IUPAC name | Canonical Smiles |
| --- | --- | --- | --- |
| 1 | RL001 | (1R,9S)-4-bromo-1,5,12,12-tetramethyl-8-oxatricyclo[7.2.1.0^{2,7}]dodeca-2(7),3,5-triene | Cc1cc2O[C@H]3CC[C@@](C)(c2cc1Br)[C@]3(C)C |
| 2 | RL002 | 4-bromo-5-methyl-2-(4,5,5-trimethyl-1-cyclopentenyl)phenol | CC2CC=C(c1cc(Br)c(C)cc1O)C2(C)C |
| 3 | RL003 | 2-methyl-5-(1,2,3-trimethylcyclopent-2-en-1-yl)phenol | CC1(CCC(C)=C1C)c2cc(O)c(C)cc2 |
| 4 | RL004 | 4-bromo-6-[(1S,2R,5R)-1,2-dimethylbicyclo[3.1.0]hexan-2-yl]-2-iodo-3-methylphenol | C[C@]2(CC[C@@H]1C[C@@]12C)c3cc(Br)c(C)c(I)c3O |
| 5 | RL005 | (1R,9R,12R)-4,6-dibromo-9-(bromomethyl)-1,5,12- trimethyl-8-oxatricyclo[7.2.1.0^{2,7}]dodeca-2(7),3,5-triene | Cc1c(cc2c(c1Br)OC3(CCC2(C3C)C)CBr)Br |
| 6 | RL006 | 4-bromo-2-{3-bromo-5-[(1S,2S,5R)-1,2-dimethylbicyclo[3.1.0]hexan-2-yl]-6-hydroxy-2-methylphenyl}-6-[(1S,2S,5R)-1,2-dime | Cc1c(cc(c(c1c2c(c(cc(c2O)C3(CCC4C3(C4)C)C)Br)C)O)C5(CCC6C5(C6)C)C)Br |
| 7 | RL007 | 1-(1,3-dimethyl-2-methylene-cyclopentyl)-4-methyl-benzene | c1c(ccc(c1)C)C2(C(=C)C(C)CC2)C |
| 8 | RL008 | 2,4-dibromo-6-[(1S,2R,5R)-1,2-dimethylbicyclo[3.1.0]hex-2-yl]-3-methylphenol | C[C@]2(CC[C@@H]1C[C@@]12C)c3cc(Br)c(C)c(Br)c3O |
| 9 | RL009 | 1-[(1S,3S)-3-bromo-1,2,2-trimethylcyclopentyl]-4-methylbenzene | Br[C@H]2CC[C@@](C)(c1ccc(C)cc1)C2(C)C |
| 10 | RL010 | 1-[(1S,3R)-3-bromo-1,2,2-trimethylcyclopentyl]-4-methylbenzene | Br[C@@H]2CC[C@@](C)(c1ccc(C)cc1)C2(C)C |
| 11 | RL011 | 4-methyl-1-(6-methylhepta-1,5-dien-2-yl)cyclohex-3-en-1-ol | CC1=CCC(CC1)(C(=C)CCC=C(C)C)O |
| 12 | RL012 | 2-[(1R,2R,3R)-3-hydroxy-1,2,3-trimethylcyclopentyl]-5-methylphenol | C[C@]1(CC[C@@](C)(O)[C@@H]1C)c2ccc(C)cc2O |
| 13 | RL013 | (1R,2R,3R,9aR)-2-bromo-1,4,7,9a-tetramethyl-2,3,5,6,9,9a-hexahydro-1H-benzo[7]annulen-3-ol | O[C@@H]2C(\C)=C1\CCC(/C)=C\C[C@]1(C)[C@@H](C)[C@H]2Br |
| 14 | RL014 | (2aR,3S,4aR,8R,8aS)-3-bromo-2a,4a,5,8-tetramethyl-1,2,2a,3,4,4a-hexahydrocyclobuta[c]inden-7(8H)-one | C[C@H]1C(=O)\C=C(\C)[C@@]2(C)C[C@H](Br)[C@]3(C)CC[C@]123 |
| 15 | RL015 | 5,5'-dibromo-3-[(1R,5S)-1,2-dimethylbicyclo[3.1.0]hex-2-yl]-3'-[(5R)-1,2-dimethylbicyclo[3.1.0]hex-2-yl]-6,6'-dimethylbiphenyl-2,2'-diol | C[C@@]65C[C@@H]6CC[C@]5(C)c1cc(Br)c(C)c(c1O)c2c(C)c(Br)cc(c2O)[C@]4(C)CC[C@@H]3CC34C |
| 16 | RL016 | 2-[(1R,2S)-1,2-dimethyl-3-methylidenecyclopentyl]-5-methylphenol | Cc1ccc(c(c1)O)C2(CCC(=C)C2C)C |
| 17 | RL017 | 2-[(1R,2R,3E)-3-(bromomethylidene)-1,2-dimethylcyclopentyl]-5-methylphenol | C[C@]1(CC/C(=C\Br)[C@@H]1C)c2ccc(C)cc2O |
| 18 | RL018 | (2E)-2-tridecylheptadec-2-enal | CCCCCCCCCCCCCC(=C\CCCCCCCCCCCCCC)/C=O |
| 19 | RL019 | (3S,4R,4aR)-3-hydroxy-1,4,4a,7-tetramethyl-3,4,4a,5,8,9-hexahydro-2H-benzo[7]annulen-2-one | O[C@@H]2C(=O)C(/C)=C1/CCC(/C)=C\C[C@]1(C)[C@H]2C |
| 20 | RL020 | (3S,4R,4aR)-3-hydroxy-1,4,4a,7-tetramethyl-3,4,4a,5,8,9-hexahydro-2H-benzo[7]annulen-2-one | C\C1=C\C[C@@]2(C)C(/CC1)=C(/C)C(=O)[C@H](O)[C@@H]2C |
| 21 | RL021 | 4-bromo-2-[(1S,2R,5R)-1,2-dimethylbicyclo[3.1.0]hex-2-yl]-5-methylphenol | C[C@]2(CC[C@@H]1C[C@@]12C)c3cc(Br)c(C)cc3O |
| 22 | RL022 | (1R)-1,5,12,12-tetramethyl-8-oxatricyclo[7.2.1.02,7]dodeca-2,4,6-triene | Cc1cc2O[C@H]3CC[C@@](C)(c2cc1)C3(C)C |
| 23 | RL023 | 4-bromo-2-[(1R,2R,3E)-3-(bromomethylidene)-1,2-dimethylcyclopentyl]-5-methylphenol | C[C@]1(CC/C(=C\Br)[C@@H]1C)c2cc(Br)c(C)cc2O |
| 24 | RL024 | (1R,9S,12S)-4-bromo-1,5,9,12-tetramethyl-8-oxatricyclo[7.2.1.02,7]dodeca-2,4,6-triene | Cc1cc2O[C@@]3(C)CC[C@@](C)(c2cc1Br)[C@@H]3C |
| 25 | RL025 | (1R,9S,12S)-1,5,9,12-tetramethyl-8-oxatricyclo[7.2.1.02,7]dodeca-2,4,6-triene | Cc1cc2O[C@@]3(C)CC[C@@](C)(c2cc1)[C@@H]3C |
| 26 | RL026 | 4-bromo-2-[(1S,3R)-1,3-dimethyl-2-methylidenecyclopentyl]-5-methylphenol | C[C@@]1(CC[C@@H](C)C1=C)c2c(O)cc(C)c(Br)c2 |
| 28 | RL027 | 2-[(1S,3R)-1,3-dimethyl-2-methylidenecyclopentyl]-5-methylphenol | C[C@@]1(CC[C@@H](C)C1=C)c2c(O)cc(C)cc2 |
| 29 | RL028 | (1R,3S,4S,6S,8R,11R)-3,8-dibromo-4-chloro-4,11,12,12-tetramethyl-7-oxatricyclo[6.3.1.01,6]dodec-9-en-11-ol | Br[C@H]1C[C@@]32[C@](O)(/C=C\[C@](Br)(O[C@H]2C[C@@]1(Cl)C)C3(C)C)C |
| 30 | RL029 | (4E)-1-bromo-5-[(1S,3R)-3-bromo-2,2-dimethyl-6-methylidenecyclohexyl]-3-methylpent-4-ene-2,3-diol | C[C@@]1(C)[C@@H](/C=C/C(C)(O)C(O)CBr)C(=C)CC[C@H]1Br |
| 31 | RL030 | (3E)-1-bromo-5-[(1S)-2,2-dimethyl-6-methylidenecyclohex-3-en-1-yl]-3-methylpent-3-en-2-ol | CC1(C)C=CCC(=C)[C@@H]1C\C=C(/C)C(O)CBr |
| 32 | RL031 | (3E)-1-bromo-5-[(1S,3R)-3-bromo-2,2-dimethyl-6-methylidenecyclohexyl]-3-methylpent-3-en-2-ol | CC1(C)[C@@H](C\C=C(/C)C(O)CBr)C(=C)CC[C@H]1Br |
| 33 | RL032 | (2R,7S,9aS)-7-bromo-2-(bromomethyl)-3,6,6,9a-tetramethyl-2,5,5a,6,7,8,9,9a-octahydro-1-benzoxepine | C[C@@]1(C)C2CC=C(C)[C@H](CBr)O[C@@]2(C)CC[C@@H]1Br |
| 34 | RL033 | (3aR,4aS,7S)-7-bromo-4a,8,8-trimethyl-3a,4a,5,6,7,8,8a,9-octahydrofuro[3,4-b][1]benzoxepin-1(3H)-one | C[C@@]1(C)C2CC=C3C(=O)OC[C@@H]3O[C@@]2(C)CC[C@@H]1Br |
| 35 | RL034 | (3aR,4aS,7S)-7-bromo-4a,8,8-trimethyl-1-methylidene-1,3,3a,4a,5,6,7,8,8a,9-decahydrofuro[3,4-b][1]benzoxepine | C[C@@]1(C)C2CC=C3C(=C)OC[C@@H]3O[C@@]2(C)CC[C@@H]1Br |
| 36 | RL035 | (2R,7S,9aS)-7-bromo-2-(bromomethyl)-6,6,9a-trimethyl-3-methylidenedecahydro-1-benzoxepin-4-ol | C[C@@]1(C)C2CC(O)C(=C)[C@H](CBr)O[C@@]2(C)CC[C@@H]1Br |
| 37 | RL036 | (2R,7S,9aS)-7-bromo-2-(bromomethyl)-3,6,6,9a-tetramethyl-2,5,5a,6,7,8,9,9a-octahydro-1-benzoxepin-5-yl acetate | CC(=O)O[C@@H]1/C=C(/C)[C@H](CBr)O[C@@]2(C)CC[C@H](Br)C(C)(C)[C@H]12 |
| 38 | RL037 | (1aR,3aR,7R,7aR,7bS)-3a-hydroxy-1,1,7,7a-tetramethyldecahydro-3H-cyclopropa[a]naphthalen-3-one | CC2(C)[C@@H]1CC(=O)[C@@]3(O)CCC[C@@H](C)[C@]3(C)[C@@H]12 |
| 39 | RL038 | (1aR,7R,7aR,7bS)-1,1,7,7a-tetramethyl-1,1a,3a,5,6,7,7a,7b-octahydro-4H-cyclopropa[a]naphthalen-4-one | CC2(C)[C@@H]1/C=C\[C@H]3C(=O)CC[C@@H](C)[C@]3(C)[C@@H]12 |
| 40 | RL039 | (1aR,7R,7aR,7bS)-1,1,7,7a-tetramethyl-1,1a,2,5,6,7,7a,7b-octahydro-4H-cyclopropa[a]naphthalen-4-one | CC3(C)[C@@H]1[C@H]3C\C=C2\C(=O)CC[C@@H](C)[C@]12C |
| 41 | RL040 | (1aR,7R,7aR,7bS)-1,1,7,7a-tetramethyl-1,1a,2,5,6,7,7a,7b-octahydro-3H-cyclopropa[a]naphthalen-3-one | O=C2C[C@@H]3[C@H]([C@]1(C)C2=CCC[C@H]1C)[C@]3(C)C |
| 42 | RL041 | (1aR,7R,7aR,7bS)-1,1,7,7a-tetramethyl-1a,2,3,5,6,7,7a,7b-octahydro-1H-cyclopropa[a]naphthalen-3-ol | C[C@@H]1CCC=C2C(O)C[C@@H]3[C@H]([C@@]12C)[C@]3(C)C |
| 43 | RL042 | (1aR,7R,7aR,7bS)-1,1,7,7a-tetramethyl-1a,5,6,7,7a,7b-hexahydro-1H-cyclopropa[a]naphthalene | C[C@@H]2CCC=C1C=C[C@@H]3[C@H]([C@]12C)[C@]3(C)C |
| 44 | RL043 | (1aR,7R,7aR,7bS)-1,1,7,7a-tetramethyl-1a,2,6,7,7a,7b-hexahydro-1H-cyclopropa[a]naphthalene | C[C@@H]2CC=CC1=CC[C@@H]3[C@H]([C@]12C)[C@]3(C)C |
| 45 | RL044 | (1aR,7R,7aR,7bS)-1,1,7,7a-tetramethyl-1,1a,4,5,6,7,7a,7b-octahydro-2H-cyclopropa[a]naphthalen-2-one | O=C2C=C1CCC[C@@H](C)[C@]1(C)[C@H]3[C@@H]2[C@@]3(C)C |
| 46 | RL045 | (1S,2S,3S,4S)-4-bromo-2-(3-hydroxy-3-methyl-pent-4-enyl)-3-[(E)-4-hydroxy-4-methyl-pent-2-enyl]-1,3-dimethyl-cyclohexanol | CC1(CCC(C(C1CCC(C)(C=C)O)(C)CC=CC(C)(C)O)Br)O |
| 47 | RL046 | (1S,4S,5S,6S,9S)-9-bromo-4-{[(1S,4S)-4-bromo-1- hydroxy-3,3-dimethylcyclohexyl]methyl}-6-methyl-11- oxatricyclo[4.3.2.0^{1,5}]undecan-10-ol | CC1(C)C[C@](O)(CC[C@@H]1Br)C[C@@H]3CC[C@@]24[C@@H](Br)CC[C@@](C)(OC2O)[C@@H]34 |
| 48 | RL047 | (2R,5S,7R,10S,12R,14S)-5-bromo-2,6,6,10,14- pentamethyl-13- oxatetracyclo[8.6.0.0^{2,7}.0^{12,14}]hexadecane | C[C@]13CC[C@H]4[C@](C)(C)[C@@H](Br)CC[C@]4(C)C3CC[C@]2(C)O[C@@H]2C1 |
| 49 | RL048 | (4aR,6aR,7aS,8aR,10aS,10bR)-4,4,6a,8a,10b-pentamethyltetradecahydro-1H-naphtho[1',2':5,6]cyclohepta[1,2-b]oxirene | C[C@]13CC[C@H]4[C@@](C)(C)[C@@H2]CC[C@]4(C)C3CC[C@]2(C)O[C@@H]2C1 |
| 50 | RL049 | (2R,3Z)-1-bromo-5-[(3S)-3-bromo-2,2-dimethyl-6-methylidenecyclohexyl]-3-methylpent-3-en-2-ol | C[C@]1(C)C(C\C=C(\C)[C@@H](O)CBr)C(=C)CC[C@@H]1Br |
| 51 | RL050 | (1R,2S,3S,4S)-4-bromo-2-(3-hydroxy-3-methylpent-4-en-1-yl)-1,3-dimethyl-3-(4-methylpent-3-en-1-yl)cyclohexanol | C=CC(C)(O)CC[C@@H]1[C@](C)(O)CC[C@H](Br)[C@@]1(C)CC\C=C(/C)C |
| 52 | RL051 | (3Z)-1-bromo-3-[(5S,7aS)-5-bromo-4,4,7a-trimethylhexahydro-1-benzofuran-2(3H)-ylidene]butan-2-one | BrCC(=O)C(/C)=C1/CC2[C@](C)(C)[C@@H](Br)CC[C@]2(C)O1 |
| 53 | RL052 | (2S,5S,7aS)-5-bromo-2-(furan-3-yl)-4,4,7a-trimethyloctahydro-1-benzofuran | C[C@]1(C)[C@@H](Br)CC[C@]2(C)O[C@@H](CC12)c3ccoc3 |
| 54 | RL053 | (1aR,2R,3aS,6S,8aR)-6-bromo-2-(bromomethyl)-1a,3a,7,7-tetramethyldecahydrooxireno[c][1]benzoxepine | C[C@@]2(C)C3C[C@H]1O[C@@]1(C)[C@H](CBr)O[C@@]3(C)CC[C@@H]2Br |
| 55 | RL054 | (1aR,2E,3aS,6S,8aR)-6-bromo-2-(bromomethylidene)-1a,3a,7,7-tetramethyldecahydrooxireno[c][1]benzoxepine | C[C@]13O[C@@H]3CC2[C@](C)(C)[C@@H](Br)CC[C@]2(C)OC1=[C@H]Br |
| 56 | RL055 | (1S,4aS,7S)-7-bromo-4a,8,8-trimethyl-1,3,3a,4a,5,6,7,8,8a,9-decahydrofuro[3,4-b][1]benzoxepin-1-ol | Br[C@H]1CC[C@]2(C)OC3CO[C@H](O)C3=CCC2[C@]1(C)C |
| 57 | RL056 | (1S,2R,7S,10S,12S)-2,6,6,10-tetramethyl-13- methylidenetricyclo[8.5.0.0^{2,7}]pentadec-4-en-12- ol | C=C1CC[C@@H]2[C@@]3(C)CC=C[C@](C)(C)[C@@H]3CC[C@@]2(C)C[C@@H]1O |
| 58 | RL057 | [(2R,7S,9aS)-7-bromo-2-(bromomethyl)-6,6,9a-trimethyl-2,5,5a,6,7,8,9,9a-octahydro-1-benzoxepin-3-yl]methanol | C[C@@]1(C)C2[C@H2]C=C(CO)[C@H](CBr)O[C@@]2(C)CC[C@@H]1Br |
| 59 | RL058 | 3,5,6-Tribromo-1-methyl-1H-indole | Brc1cc2c(cc1Br)n(C)cc2Br |
| 60 | RL059 | 3,5,6-Tribromo-1-methyl-1H-indole | Brc1cc2c(cc1Br)n(C)cc2Br |
| 61 | RL060 | 2,3,6-Tribromo-1H-indole | Brc1ccc2c(c1)nc(Br)c2Br |
| 62 | RL061 | 2,3,5-tribromo-1-methyl-1H-indole | Brc1cc2c(cc1)n(C)c(Br)c2Br |
| 63 | RL062 | 2,3,5,6-tetrabromo-1H-indole | Brc1cc2c(cc1Br)nc(Br)c2Br |
| 64 | RL063 | (2R,3S,6R)-2-bromo-8-chloro-1,1,9-trimethyl-5-methylidenespiro[5.5]undec-8-en-3-ol | CC1=C(Cl)C[C@]2(CC1)C(=C)C[C@H](O)[C@H](Br)C2(C)C |
| 65 | RL064 | (2Z,6Z,8Z,12E)-N-(1,3-dihydroxypropan-2-yl)-13-[(1S,2S)-2-methylcyclopropyl]trideca-2,6,8,12-tetraenamide | OCC(CO)NC(=O)/C=C\CC\C=C/C=C\CC\C=C\[C@H]1C[C@@H]1C |
| 66 | RL065 | (2S,3S,5R)-2-[(2R,4aS,6R,8aR)-6-{5-[5-(2-hydroxypropan-2-yl)-2-methyloxolan-2-yl]-2-methyloxolan-2-yl}-4a-methyl-octahydropyrano[3,2-b]pyran-2-yl]-5-bromo-2,6,6-trimethyloxan-3-ol | Br[C@@H]1C[C@H](O)[C@](C)(O[C@]1(C)C)[C@H]2CC[C@]3(C)O[C@H](CC[C@H]3O2)C4(C)CCC(O4)C5(C)CCC(O5)C(C)(C)O |
| 67 | RL066 | (1R,4S,4aS,7R,8aR)-4-bromo-7-[(1R,3R,5S)-3-bromo-5-hydroxy-1,2,2-trimethylcyclopentyl]-1,4a-dimethyl-decahydronaphthalene-1,7,8a-triol | C[C@]3(C)[C@H](Br)C[C@H](O)[C@]3(C)[C@@]1(O)CC[C@]2(C)[C@@H](Br)CC[C@@](C)(O)[C@@]2(O)C1 |
| 68 | RL067 | 2-[5-(5-{5-[(4aS,7R,9aR)-7-bromo-2,6,6,9a-tetramethyl-octahydro-2H-pyrano[3,2-b]oxepin-2-yl]oxolan-2-yl}-5-methyloxolan-2-yl)-5-methyloxolan-2-yl]propan-2-ol | CC(C)(O)C1CCC(C)(O1)C2CCC(C)(O2)C3CCC(O3)C4(C)CC[C@@H]5OC(C)(C)[C@H](Br)CC[C@@]5(C)O4 |
| 69 | RL068 | (1R,5R)-3-bromo-9-chloro-2,2,6,9-tetramethyl-7-oxatricyclo[6.3.1.01,6]dodec-3-en-5-ol | C[C@]3(C)C(Br)=C[C@@H](O)[C@@]2(C)OC1C[C@@]23CCC1(C)Cl |
| 70 | RL069 | (2S,5R,7S,8S)-7-bromo-8-chloro-8-methyl-4-methylidene-2-(2-methylprop-1-en-1-yl)-1-oxaspiro[4.5]decane | C/C(C)=C\[C@@H]2CC(=C)[C@@]1(C[C@H](Br)[C@@](C)(Cl)CC1)O2 |
| 71 | RL070 | (2R,5R,7S,8S)-7-bromo-8-chloro-8-methyl-4-methylidene-2-(2-methylprop-1-en-1-yl)-1-oxaspiro[4.5]decane | C/C(C)=C\[C@H]2CC(=C)[C@@]1(C[C@H](Br)[C@@](C)(Cl)CC1)O2 |
| 72 | RL071 | (3S,6S)-4,8-dibromo-1,5,5,9-tetramethylspiro[5.5]undeca-1,8-dien-3-ol | CC1=C(Br)C[C@@]2(CC1)C(C)=C[C@H](O)C(Br)C2(C)C |
| 73 | RL072 | (3S,6S)-4,8-dibromo-9-chloro-1,5,5,9-tetramethylspiro[5.5]undec-1-en-3-ol | CC2=C[C@H](O)C(Br)[C@](C)(C)[C@@]12CCC(C)(Cl)C(Br)C1 |
| 76 | RL075 | (2R,4R,7S,9aS)-7-bromo-2-(bromomethyl)-6,6,9a-trimethyl-3-methylidenedecahydro-1-benzoxepin-4-ol | CC1(C)C2C[C@@H](O)C(=C)[C@H](CBr)O[C@@]2(C)CC[C@@H]1Br |
| 74 | RL073 | 4-[(2S,5S,7aS)-5-bromo-4,4,7a-trimethyloctahydro-1-benzofuran-2-yl]-3-hydroxyfuran-2(5H)-one | CC1(C)[C@@H](Br)CC[C@]2(C)O[C@@H](CC12)C1=C(O)C(=O)OC1 |
| 75 | RL074 | 4-[(2R,5S,7aS)-5-bromo-4,4,7a-trimethyloctahydro-1-benzofuran-2-yl]-3-hydroxyfuran-2(5H)-one | CC1(C)[C@@H](Br)CC[C@]2(C)O[C@H](CC12)C1=C(O)C(=O)OC1 |
| 77 | RL076 | (3E)-4-[(1S,3R)-3-bromo-2,2-dimethyl-6-methylidenecyclohexyl]but-3-en-2-one | CC1(C)[C@@H](/C=C/C(C)=O)C(=C)CC[C@H]1Br |
| 78 | RL077 | (1S,3aS,4R,5S)-1,5-dimethyl-4-(2-methylprop-1-en-1-yl)octahydro-3aH-inden-3a-ol | C/C(C)=C\[C@H]1[C@@H](C)CCC2[C@@H](C)CC[C@@]12O |
| 79 | RL078 | 6-[(2S,5R)-5-[(2R,4aR,6R,8aS)-6-[(2S,5R)-5-bromo-2,6,6-trimethyloxan-2-yl]-8a-methyl-octahydropyrano[3,2-b]pyran-2-yl]-5-methyloxolan-2-yl]-2-methylheptane-2,3,6-triol | Br[C@@H]1CC[C@](C)(O[C@]1(C)C)[C@H]2CC[C@]3(C)O[C@H](CC[C@H]3O2)[C@@]4(C)CC[C@H](O4)C(C)(O)CCC(O)C(C)(C)O |
| 80 | RL079 | (1S,4S)-4-[(2R,4aR,6R,8aS)-6-[(2S,5R)-5-bromo-2,6,6-trimethyloxan-2-yl]-8a-methyl-octahydropyrano[3,2-b]pyran-2-yl]-1-[(2R,4S,5R)-4-hydroxy-5-(2-hydroxypropan-2-yl)-2-methyloxolan-2-yl]pentane-1,4-diol | Br[C@@H]1CC[C@](C)(O[C@]1(C)C)[C@H]2CC[C@]3(C)O[C@H](CC[C@H]3O2)[C@@](C)(O)CC[C@H](O)[C@@]4(C)C[C@H](O)[C@@H](O4)C(C)(C)O |
| 81 | RL080 | (1S,4S)-4-[(2R,4aR,6R,8aS)-6-[(2S,5R)-5-bromo-2,6,6-trimethyloxan-2-yl]-8a-methyl-octahydropyrano[3,2-b]pyran-2-yl]-1-[(2R,5R)-5-(2-hydroxypropan-2-yl)-2-methyloxolan-2-yl]pentane-1,4-diol | Br[C@@H]1CC[C@](C)(O[C@]1(C)C)[C@H]2CC[C@]3(C)O[C@H](CC[C@H]3O2)[C@@](C)(O)CC[C@H](O)[C@@]4(C)C[C@@H2][C@@H](O4)C(C)(C)O |
| 82 | RL081 | (2S,3S,5Z,8S)-3-chloro-2-(pent-2-en-4-yn-1-yl)-8-[(1E)-prop-1-en-1-yl]-3,4,7,8-tetrahydro-2H-oxocine | C\C=C\[C@@H]1CC=CC[C@H](Cl)[C@H](C/C=C\C#C)O1 |
| 83 | RL082 | 2-[(2S,5R)-5-[(2S,5R)-5-[(2S,5S)-5-[(2R,4aS,7R,9aR)-7-bromo-2,6,6,9a-tetramethyl-octahydro-2H-pyrano[3,2-b]oxepin-2-yl]oxolan-2-yl]-5-methyloxolan-2-yl]-5-methyloxolan-2-yl]propan-2-ol | CC(C)(O)[C@@H]1CC[C@@](C)(O1)[C@@H]2CC[C@@](C)(O2)[C@@H]3CC[C@H](O3)[C@@]4(C)CC[C@@H]5O[C@](C)(C)[C@H](Br)CC[C@@]5(C)O4 |
| 84 | RL083 | (2S)-2-[(2R,5S)-5-[(2R,5S)-5-[(2R,5S)-5-[(2R,5S)-5-(2-hydroxypropan-2-yl)-2-methyloxolan-2-yl]-2-methyloxolan-2-yl]oxolan-2-yl]-5-methyloxolan-2-yl]-6-methylhept-5-en-2-ol | C/C(C)=C\CC[C@](C)(O)[C@H]1CC[C@](C)(O1)[C@H]2CC[C@H](O2)[C@@]3(C)CC[C@H](O3)[C@]4(C)O[C@@H](CC4)C(C)(C)O |
| 85 | RL084 | (1S,3E)-4-[(2R,4aR,6R,8aS)-6-[(2S,5R)-5-bromo-2,6,6-trimethyloxan-2-yl]-8a-methyl-octahydropyrano[3,2-b]pyran-2-yl]-1-[(2R,5R)-5-(2-hydroxypropan-2-yl)-2-methyloxolan-2-yl]pent-3-en-1-ol | Br[C@@H]1CC[C@](C)(O[C@]1(C)C)[C@H]2CC[C@]3(C)O[C@H](CC[C@H]3O2)C(\C)=C\C[C@H](O)[C@@]4(C)CC[C@@H](O4)C(C)(C)O |
| 86 | RL085 | 2,3,4,6-tetrabromo-1-methyl-1H-indole | Brc1cc2c(c(Br)c1)c(Br)c(Br)n2C |
| 87 | RL086 | (1S,4S)-4-bromo-1-{[(1S,4S,5S,6S,9S)-9-bromo-10- hydroxy-6-methyl-11- oxatricyclo[4.3.2.0^{1,5}]undecan-4-yl]methyl}-3,3- dimethylcyclohexyl acetate | CC(=O)O[C@]1(CC(C)(C)[C@@H](Br)CC1)C[C@@H]3CC[C@@]24[C@@H](Br)CC[C@@](C)(OC2O)[C@@H]34 |
| 88 | RL087 | 2,3,5,6-tetrabromo-1-methyl-1H-indole | Brc1cc2c(cc1Br)n(C)c(Br)c2Br |
| 89 | RL088 | (1aR,7R,7aR,7bS)-1,1,7,7a-tetramethyl-1a,2,3,5,6,7,7a,7b-octahydro-1H-cyclopropa[a]naphthalene | C[C@@H]2CCC=C1CC[C@@H]3[C@H]([C@]12C)C3(C)C |
| 90 | RL089 | (6S)-2,9-dibromo-8-chloro-1,1,9-trimethyl-5-methylidenespiro[5.5]undecane | C=C2CCC(Br)[C@](C)(C)[C@@]12CCC(Br)(C)C(Cl)C1 |
| 91 | RL090 | 2-[(1R,2R,3E)-3-(bromomethylidene)-1,2-dimethylcyclopentyl]-5-methylphenol | C[C@]1(CC/C(=C\Br)[C@@H]1C)c2ccc(C)cc2O |
| 92 | RL091 | (2E)-2-tridecylheptadec-2-enal | CCCCCCCCCCCCCC(=C\CCCCCCCCCCCCCC)/C=O |
| 93 | RL092 | (2R,7S,9aS)-7-bromo-2-(bromomethyl)-3,6,6,9a-tetramethyl-2,5,5a,6,7,8,9,9a-octahydro-1-benzoxepine | C[C@@]1(C)C2CC=C(C)[C@H](CBr)O[C@@]2(C)CC[C@@H]1Br |
| 94 | RL093 | (3aR,4aS,7S)-7-bromo-4a,8,8-trimethyl-3a,4a,5,6,7,8,8a,9-octahydrofuro[3,4-b][1]benzoxepin-1(3H)-one | C[C@@]1(C)C2CC=C3C(=O)OC[C@@H]3O[C@@]2(C)CC[C@@H]1Br |
| 95 | RL094 | (2R,7S,9aS)-7-bromo-2-(bromomethyl)-3,6,6,9a-tetramethyl-2,5,5a,6,7,8,9,9a-octahydro-1-benzoxepine | C[C@@]1(C)C2CC=C(C)[C@H](CBr)O[C@@]2(C)CC[C@@H]1Br |
| 96 | RL095 | (3aR,4aS,7S)-7-bromo-4a,8,8-trimethyl-3a,4a,5,6,7,8,8a,9-octahydrofuro[3,4-b][1]benzoxepin-1(3H)-one | C[C@@]1(C)C2CC=C3C(=O)OC[C@@H]3O[C@@]2(C)CC[C@@H]1Br |
| 97 | RL096 | (2R,7S,9aS)-7-bromo-2-(bromomethyl)-3,6,6,9a-tetramethyl-2,5,5a,6,7,8,9,9a-octahydro-1-benzoxepine | C[C@@]1(C)C2CC=C(C)[C@H](CBr)O[C@@]2(C)CC[C@@H]1Br |
| 98 | RL097 | (3aR,4aS,7S)-7-bromo-4a,8,8-trimethyl-1-methylidene-1,3,3a,4a,5,6,7,8,8a,9-decahydrofuro[3,4-b][1]benzoxepine | C[C@@]1(C)C2CC=C3C(=C)OC[C@@H]3O[C@@]2(C)CC[C@@H]1Br |
| 99 | RL098 | (3aR,4aS,7S)-7-bromo-4a,8,8-trimethyl-1-methylidene-1,3,3a,4a,5,6,7,8,8a,9-decahydrofuro[3,4-b][1]benzoxepine | C[C@@]1(C)C2CC=C3C(=C)OC[C@@H]3O[C@@]2(C)CC[C@@H]1Br |
| 100 | RL099 | (2R,7S,9aS)-7-bromo-2-(bromomethyl)-3,6,6,9a-tetramethyl-2,5,5a,6,7,8,9,9a-octahydro-1-benzoxepin-5-yl acetate | CC(=O)O[C@@H]1/C=C(/C)[C@H](CBr)O[C@@]2(C)CC[C@H](Br)C(C)(C)[C@H]12 |
| 101 | RL100 | (1aR,3aR,7R,7aR,7bS)-3a-hydroxy-1,1,7,7a-tetramethyldecahydro-3H-cyclopropa[a]naphthalen-3-one | CC2(C)[C@@H]1CC(=O)[C@@]3(O)CCC[C@@H](C)[C@]3(C)[C@@H]12 |
| 102 | RL101 | (1aR,7R,7aR,7bS)-1,1,7,7a-tetramethyl-1,1a,3a,5,6,7,7a,7b-octahydro-4H-cyclopropa[a]naphthalen-4-one | CC2(C)[C@@H]1/C=C\[C@H]3C(=O)CC[C@@H](C)[C@]3(C)[C@@H]12 |
| 103 | RL102 | (1aR,7R,7aR,7bS)-1,1,7,7a-tetramethyl-1,1a,2,5,6,7,7a,7b-octahydro-3H-cyclopropa[a]naphthalen-3-one | O=C2C[C@@H]3[C@H]([C@]1(C)C2=CCC[C@H]1C)[C@]3(C)C |
| 104 | RL103 | (1aR,7R,7aR,7bS)-1,1,7,7a-tetramethyl-1a,2,3,5,6,7,7a,7b-octahydro-1H-cyclopropa[a]naphthalen-3-ol | C[C@@H]1CCC=C2C(O)C[C@@H]3[C@H]([C@@]12C)[C@]3(C)C |
| 105 | RL104 | (1aR,7R,7aR,7bS)-1,1,7,7a-tetramethyl-1a,5,6,7,7a,7b-hexahydro-1H-cyclopropa[a]naphthalene | C[C@@H]2CCC=C1C=C[C@@H]3[C@H]([C@]12C)[C@]3(C)C |
| 106 | RL105 | (1aR,7R,7aR,7bS)-1,1,7,7a-tetramethyl-1a,2,6,7,7a,7b-hexahydro-1H-cyclopropa[a]naphthalene | C[C@@H]2CC=CC1=CC[C@@H]3[C@H]([C@]12C)[C@]3(C)C |
| 107 | RL106 | (2R,7S,9aS)-7-bromo-2-(bromomethyl)-3,6,6,9a-tetramethyl-2,5,5a,6,7,8,9,9a-octahydro-1-benzoxepin-5-yl acetate | CC(=O)O[C@@H]1/C=C(/C)[C@H](CBr)O[C@@]2(C)CC[C@H](Br)C(C)(C)[C@H]12 |
| 108 | RL107 | 2,3,5-tribromo-1-methyl-1H-indole | Brc1cc2c(cc1)n(C)c(Br)c2Br |
| 109 | RL108 | (2R,3S,6R)-2-bromo-8-chloro-1,1,9-trimethyl-5-methylidenespiro[5.5]undec-8-en-3-ol | ClC=1C[C@]2(CCC=1C)C(=C)C[C@H](O)[C@H](Br)[C@]2(C)C |
| 110 | RL109 | (1S,4S)-4-[(2R,4aR,6R,8aS)-6-[(2S,5R)-5-bromo-2,6,6-trimethyloxan-2-yl]-8a-methyl-octahydropyrano[3,2-b]pyran-2-yl]-1-[(2R,5R)-5-(2-hydroxypropan-2-yl)-2-methyloxolan-2-yl]pentane-1,4-diol | Br[C@@H]1CC[C@](C)(O[C@]1(C)C)[C@H]2CC[C@]3(C)O[C@H](CC[C@H]3O2)[C@@](C)(O)CC[C@H](O)[C@@]4(C)C[C@@H2][C@@H](O4)C(C)(C)O |
| 111 | BP001 | 17-(5-hydroperoxy-5-propan-2-ylhept-6-en-2-yl)-10,13-dimethyl-2,3,4,7,8,9,11,12,14,15,16,17-dodecahydro-1H-cyclopenta[a]phenanthren-3-ol | CC(C)C(C=C)(OO)CC[C@@H](C)[C@H]1CCC2C3CC=C4C[C@@H](O)CC[C@]4(C)[C@H]3CC[C@]12C |
| 112 | RL110 | {4-[(1S,4S)-4-bromo-3,3-dimethylcyclohexyl]phenyl}methanol | Br[C@H]2CC[C@H](c1ccc(cc1)CO)CC2(C)C |
| 113 | RL111 | 4-[(1S,4S)-4-bromo-3,3-dimethylcyclohexyl]benzyl acetate | C[C@]1(C)C[C@H](CC[C@@H]1Br)c2ccc(COC(C)=O)cc2 |
| 114 | RJ001 | 6-(1-bromo-2-hydroxyethyl)-2a,4-dihydroxy-6,8b-dimethyl-1,2,2a,2b,3,4,4a,5,6,7,8b,9,10,10a-tetradecahydrocyclobuta[a]phenanthren-10-yl acetate | BrC(C1(CC2/C(=C\C1)C4(C(CC2O)C3(O)C(CC3)C(OC(=O)C)C4)C)C)CO |
| 115 | RJ002 | 6-[2-(acetyloxy)-1-bromoethyl]-2a,4-dihydroxy-6,8b-dimethyl-1,2,2a,2b,3,4,4a,5,6,7,8b,9,10,10a-tetradecahydrocyclobuta[a]phenanthren-10-yl acetate | BrC(C1(CC2/C(=C\C1)C4(C(CC2O)C3(O)C(CC3)C(OC(=O)C)C4)C)C)COC(=O)C |
| 116 | RJ003 | (2aR,2bR,4S,4aS,6R,8bS,10S,10aR)-6-[2-(acetyloxy)-1-bromoethyl]-2a-hydroxy-6,8b-dimethyl-1,2,2a,2b,3,4,4a,5,6,7,8b,9,10,10a-tetradecahydrocyclobuta[a]phenanthrene-4,10-diyl diacetate | CC(=O)OCC(Br)[C@]1(C)C\C=C4/[C@H](C1)[C@H](C[C@@H]2[C@]4(C)C[C@H](OC(C)=O)[C@H]3CC[C@@]23O)OC(C)=O |
| 117 | RJ004 | 15-bromo-7,18-dihydroxy-3,19-cyclopimar-9(11)-ene-2,16-diyl diacetate | BrC(C1(CC2/C(=C\C1)C4(C(CC2O)C3(C(C3)C(OC(=O)C)C4)CO)C)C)COC(=O)C |
| 118 | RJ005 | (2alpha,3beta,5beta,7alpha,8alpha,10alpha,13alpha)-15-bromo-18-hydroxy-3,19-cyclopimar-9(11)-ene-2,7,16-triyl triacetate | BrC(COC(=O)C)[C@]1(C)C[C@@H]4\C(=C/C1)[C@]2([C@@H]([C@@]3([C@H]([C@H](OC(=O)C)C2)C3)CO)C[C@H]4OC(=O)C)C |
| 119 | RJ006 | 15-bromo-7,16-dihydroxy-3,19-cyclopimar-9(11)-en-2-yl acetate | BrC(C1(CC2/C(=C\C1)C4(C(CC2O)C3(C(C3)C(OC(=O)C)C4)C)C)C)CO |
| 120 | RJ007 | (3beta)-15-bromo-16-hydroxy-3,19-cyclopimar-9(11)-ene-2,7-diyl diacetate |  |
| 121 | RL112 | 5-[(1R,3R,5R)-3,5-dibromo-2,2-dimethyl-6-methylidenecyclohexyl]-3-methylpent-1-en-3-ol | BrC1C[C@@H](Br)C(=C)C(CCC(C)(O)C=C)C1(C)C |
| 122 | RL113 | 5-[(1R,3S,5S)-3,5-dibromo-2,2-dimethyl-6-methylidenecyclohexyl]-3-methylpent-1-en-3-ol | Br[C@H]1CC(Br)C(=C)C(CCC(C)(O)C=C)C1(C)C |
| 123 | RL114 | (3E)-5-bromo-3-(3-hydroxy-3-methylpent-4-en-1-ylidene)-2,4,4-trimethylcyclohexanone | CC1(C)C(=C\CC(C)(O)C=C)\C(C)C(=O)CC1Br |
| 124 | RL115 | (1Z)-3-methyl-1-[(1S,6R)-2,2,6-trimethyl-7-oxabicyclo[4.1.0]hept-1-yl]penta-1,4-dien-3-yl acetate | O=C(OC(/C=C\[C@@]21O[C@@]2(CCCC1(C)C)C)(\C=C)C)C |
| 125 | RL116 | 5-[(5S)-5-bromo-2,6,6-trimethylcyclohex-2-en-1-yl]-3-methylpent-1-en-3-ol | Br[C@H]1C\C=C(\C)C(CCC(C)(O)C=C)C1(C)C |
| 126 | RL117 | 5-(5-bromo-2,6,6-trimethylcyclohex-2-en-1-yl)-3-methylpent-1-en-3-yl acetate | C[C@@]1(C)C(CCC(C)(C=C)OC(C)=O)C(C)=CCC1Br |
| 127 | RL118 | 5-(3-bromo-2,2-dimethyl-6-methylidenecyclohexyl)-3-methylpent-1-en-3-ol | C[C@@]1(C)C(CCC(C)(O)C=C)C(=C)CCC1Br |
| 128 | RL119 | (1S,1aS,3R,3aR,4S,6bS)-4-bromo-3,6-dimethyl-1-(propan-2-yl)-1a,2,3,4,5,6b-hexahydrocyclopropa[e]inden-3a(1H)-ol | CC(C)[C@@H]3[C@H]2/C1=C(\C)C[C@H](Br)[C@@]1(O)[C@H](C)C[C@H]23 |
| 129 | RL120 | (4S,5S,7S,8S)-2-[(1R)-1-bromopropyl]-5,7-dichloro-8-[(2Z)-pent-2-en-4-yn-1-yl]oxocan-4-yl acetate | CC(=O)O[C@H]1CC(O[C@@H](C/C=C\C#C)[C@@H](Cl)C[C@@H]1Cl)[C@H](Br)CC |
| 130 | RL121 | (2S,3S,5Z,8S)-8-[(1R)-1-bromopropyl]-3-chloro-2-[(2E)-pent-2-en-4-yn-1-yl]-3,4,7,8-tetrahydro-2H-oxocine | Br[C@H](CC)[C@@H]1CC=CC[C@H](Cl)[C@H](C\C=C\C#C)O1 |
| 131 | RL122 | (1R,7S)-3,6-di-O-acetyl-1,7-anhydro-1-[(1R)-1-bromopropyl]-4-chloro-2,4,5-trideoxy-7-[(2E)-pent-2-en-4-yn-1-yl]-L-arabino-heptitol | CC(=O)O[C@@H]1C[C@H](Cl)[C@H](C[C@@H](O[C@H]1C\C=C\C#C)[C@H](Br)CC)OC(C)=O |
| 132 | RL123 | [(2R,4S,5S,7R,8S)-7-acetoxy-2-(1-bromopropyl)-5-chloro-8-[(Z)-pent-2-en-4-ynyl]oxocan-4-yl] acetate | BrC([C@@H]1O[C@@H](C/C=C\C#C)[C@H](OC(=O)C)C[C@H](Cl)[C@@H](OC(=O)C)C1)CC |
| 133 | RL124 | (2R,4S,5S,7S,8S)-2-[(1R)-1-bromopropyl]-5,7-dichloro-8-(pent-2-en-4-yn-1-yl)oxocan-4-yl acetate | CC(=O)O[C@H]1C[C@@H](O[C@@H](C\C=C\C#C)[C@@H](Cl)C[C@@H]1Cl)[C@H](Br)CC |
| 134 | RL125 | (1S,4S)-4-[(2R,4aR,6R,8aS)-6-[(2S,5R)-5-bromo-2,6,6-trimethyloxan-2-yl]-8a-methyl-octahydropyrano[3,2-b]pyran-2-yl]-1-[(2R,5R)-5-(2-hydroxypropan-2-yl)-2-methyloxolan-2-yl]pentane-1,4-diol | Br[C@@H]1CC[C@](C)(O[C@]1(C)C)[C@H]2CC[C@]3(C)O[C@H](CC[C@H]3O2)[C@@](C)(O)CC[C@H](O)[C@@]4(C)C[C@@H2][C@@H](O4)C(C)(C)O |
| 135 | RL126 | (1R,4S)-4-{(2R,4aR,6R,8aS)-6-[(2S,5R)-5-bromo-2,6,6-trimethyltetrahydro-2H-pyran-2-yl]-8a-methyloctahydropyrano[3,2-b]pyran-2-yl}-1-[(2S,5R)-5-(2-hydroxypropan-2-yl)-2-methyltetrahydrofuran-2-yl]penta ne-1,4-diol | C[C@]1(CC[C@@H](O1)C(C)(C)O)[C@H](O)CC[C@](C)(O)[C@H]2CC[C@H]3O[C@H](CC[C@]3(C)O2)[C@]4(C)CC[C@@H](Br)C(C)(C)O4 |
| 136 | RL127 | (1R)-4-{(2R,4aS,6S,8aR)-6-[(2R,5S)-5-bromo-2,6,6-trimethyltetrahydro-2H-pyran-2-yl]-8a-methyloctahydropyrano[3,2-b]pyran-2-yl}-1-[(2R,5R)-5-(2-hydroxypropan-2-yl)-2-methyltetrahydrofuran-2-yl]pent-4-e n-1-ol | Br[C@H]1CC[C@@](C)(OC1(C)C)[C@@H]2CC[C@@]3(C)O[C@H](CC[C@@H]3O2)C(=C)CC[C@@H](O)[C@@]4(C)CC[C@@H](O4)C(C)(C)O |
| 137 | RL128 | (1R)-4-{(2R,4aR,6R,8aS)-6-[(2S,5R)-5-bromo-2,6,6-trimethyltetrahydro-2H-pyran-2-yl]-8a-methyloctahydropyrano[3,2-b]pyran-2-yl}-1-[(2S,5R)-5-(2-hydroxypropan-2-yl)-2-methyltetrahydrofuran-2-yl]pent-4-e n-1-ol | C[C@]1(CC[C@@H](O1)C(C)(C)O)[C@H](O)CC/C([C@H]2CC[C@H]3O[C@H](CC[C@]3(C)O2)[C@]4(C)CC[C@@H](Br)[C@](C)(C)O4)=C |
| 138 | RL129 | (1R,3Z)-4-{(2R,4aR,6R,8aS)-6-[(2S,5R)-5-bromo-2,6,6-trimethyltetrahydro-2H-pyran-2-yl]-8a-methyloctahydropyrano[3,2-b]pyran-2-yl}-1-[(2S,5R)-5-(2-hydroxypropan-2-yl)-2-methyltetrahydrofuran-2-yl]pent- 3-en-1-ol | Br[C@@H]1CC[C@](C)(O[C@]1(C)C)[C@H]2CC[C@]3(C)O[C@H](CC[C@H]3O2)C(=C)CC[C@@H](O)[C@]4(C)C[C@@H2][C@@H](O4)C(C)(C)O |
| 139 | RL130 | (1R)-4-{(2R,4aR,6R,8aS)-6-[(2S)-2-hydroxy-6-methylhept-5-en-2-yl]-8a-methyloctahydropyrano[3,2-b]pyran-2-yl}-1-[(2S,5R)-5-(2-hydroxypropan-2-yl)-2-methyltetrahydrofuran-2-yl]pent-4-en-1-yl acetate | CC(C)(O)[C@H]1CC[C@](C)(O1)[C@H](OC(C)=O)CCC(=C)[C@H]2CC[C@H]3O[C@H](CC[C@]3(C)O2)[C@@](C)(O)CC\C=C(/C)C |
| 140 | RL131 | (2R,3S,6R)-2-(3-{(2R,4aR,6R,8aS)-6-[(2S,5R)-5-bromo-2,6,6-trimethyltetrahydro-2H-pyran-2-yl]-8a-methyloctahydropyrano[3,2-b]pyran-2-yl}but-3-en-1-yl)-6-(2-hydroxypropan-2-yl)-3-methyltetrahydro-2H-pyr an-3-ol | CC(C)(O)[C@H]1CC[C@@](O)(C)[C@H](O1)CCC(=C)C2CCC3OC(CC[C@]3(C)O2)[C@]4(C)CC[C@@H](Br)[C@](C)(C)O4 |
| 141 | RL132 | (1S,3S)-4-{(2R,4aR,6R,8aS)-6-[(2S,5R)-5-bromo-2,6,6-trimethyltetrahydro-2H-pyran-2-yl]-8a-methyloctahydropyrano[3,2-b]pyran-2-yl}-1-[(2S,5R)-5-(2-hydroxypropan-2-yl)-2-methyltetrahydrofuran-2-yl]pent- 4-ene-1,3-diol | CC(C)(O)[C@H]1CC[C@](C)(O1)[C@@H](O)C[C@H](O)C(=C)[C@H]2CC[C@H]3O[C@H](CC[C@]3(C)O2)[C@]4(C)CC[C@@H](Br)[C@](C)(C)O4 |
| 142 | RL133 | (1S,3E)-4-{(2R,4aR,6R,8aR)-6-[(2S,5R)-5-bromo-2,6,6-trimethyltetrahydro-2H-pyran-2-yl]-8a-methyloctahydropyrano[3,2-b]pyran-2-yl}-1-[(2R,5R)-5-(2-hydroxypropan-2-yl)-2-methyltetrahydrofuran-2-yl]pent- 3-en-1-ol | CC(C)(O)[C@H]1CC[C@@](C)(O1)[C@@H](O)C\C=C(/C)[C@H]2CC[C@H]3O[C@H](CC[C@@]3(C)O2)[C@]4(C)CC[C@@H](Br)[C@](C)(C)O4 |
| 143 | RL134 | (2R,5S)-2-{(4aR,6R,8aS)-6-[(2S,5R)-5-bromo-2,6,6-trimethyltetrahydro-2H-pyran-2-yl]-8a-methyl-4,4a,6,7,8,8a-hexahydropyrano[3,2-b]pyran-2-yl}-5-[(2R,5R)-5-(1-hydroxy-1-methylethyl)-2-methyltetrahydrof uran-2-yl]pentane-1,2,5-triol | Br[C@@H]1CC[C@](OC1(C)C)([C@@H]2O[C@@H]3C/C=C(\O[C@@]3(C)CC2)[C@@](O)(CC[C@H](O)[C@@]4(O[C@@H](C(O)(C)C)CC4)C)CO)C |
| 144 | RL135 | (2S,5S)-2-{(4aR,6R,8aS)-6-[(2S,5R)-5-bromo-2,6,6-trimethyltetrahydro-2H-pyran-2-yl]-8a-methyl-4,4a,6,7,8,8a-hexahydropyrano[3,2-b]pyran-2-yl}-5-[(2R,5R)-5-(2-hydroxypropan-2-yl)-2-methyltetrahydrofura n-2-yl]pentane-1,2,5-triol | Br[C@@H]1CC[C@](OC1(C)C)([C@@H]2O[C@@H]3C/C=C(\O[C@@]3(C)CC2)[C@](O)(CC[C@H](O)[C@@]4(O[C@@H](C(O)(C)C)CC4)C)CO)C |
| 145 | RL136 | (1S)-4-{(2R,4aR,6R)-6-[(2S,5R)-5-bromo-2,6,6-trimethyltetrahydro-2H-pyran-2-yl]-8a-methyloctahydropyrano[3,2-b]pyran-2-yl}-1-[(2R,5R)-5-(2-hydroxypropan-2-yl)-2-methyltetrahydrofuran-2-yl]pent-4-en-1- ol | CC(C)(O)[C@H]1CCC(C)(O1)[C@@H](O)CCC(=C)C2CCC3OC(CCC3(C)O2)[C@]4(C)CC[C@@H](Br)[C@](C)(C)O4 |
| 146 | RL137 | (2S,4aR,8R,8aS)-2-ethenyl-2,8,8a-trimethyl-5-methylideneoctahydro-2H-chromene | C=C[C@]1(C)CC[C@@H]2C(=C)CC[C@@H](C)[C@]2(C)O1 |
| 147 | RL138 | (6R,10R)-2-bromo-3,7,10-trimethyl-11-methylidenespiro[5.5]undec-7-en-3-ol | C=C2[C@H](C)CC=C(C)[C@]12CC(Br)C(C)(O)CC1 |
| 148 | RL139 | (3S,3aR,8bS)-3,3a,6,8b-tetramethyl-2,3,3a,8b-tetrahydro-1H-benzo[b]cyclopenta[d]furan-3-ol | Cc2cc3O[C@]1(C)[C@@](C)(CC[C@]1(C)O)c3cc2 |
| 149 | RL140 | (3R,3aR,8bS)-7-bromo-3,3a,6,8b-tetramethyl-2,3,3a,8b-tetrahydro-1H-benzo[b]cyclopenta[d]furan-3-ol | Brc1cc3c(cc1C)O[C@]2(C)[C@@]3(C)CC[C@@]2(C)O |
| 150 | RL141 | 4-bromo-5-methyl-2-[(4S)-4,5,5-trimethylcyclopent-1-en-1-yl]phenol | C[C@H]2CC=C(c1cc(Br)c(C)cc1O)[C@@]2(C)C |
| 151 | RL142 | 1-methyl-4-[(4S)-4,5,5-trimethylcyclopent-1-en-1-yl]benzene | C[C@H]2CC=C(c1ccc(C)cc1)[C@@]2(C)C |
| 152 | RL144 | (3S,3aS,8bS)-3,3a,6,8b-tetramethyl-2,3,3a,8b-tetrahydro-1H-benzo[b]cyclopenta[d]furan | Cc1cc2O[C@@]3(C)[C@@H](C)CC[C@@]3(C)c2cc1 |
| 153 | RL145 | 4-bromo-2-[(1S,2R,5R)-1,2-dimethylbicyclo[3.1.0]hex-2-yl]-5-methylphenol | C[C@]2(CC[C@@H]1C[C@@]12C)c3cc(Br)c(C)cc3O |
| 154 | RL146 | (1R,3S,4S)-4-bromo-3-chloro-1-[(4S)-4-hydroxy-6-methylhepta-1,5-dien-2-yl]-4-methylcyclohexanol | Br[C@@]1(C)CC[C@@](O)(C[C@@H]1Cl)C(=C)C[C@H](O)/C=C(\C)C |
| 155 | RL147 | (1R,3S,4S)-4-bromo-3-chloro-1-(5-hydroxy-6-methylhepta-1,6-dien-2-yl)-4-methylcyclohexanol | Br[C@@]1(C)CC[C@@](O)(C[C@@H]1Cl)C(=C)CCC(O)C(=C)C |
| 156 | RL148 | (1S,3S,4S)-4-bromo-3-chloro-1-(5-hydroxy-6-methylhepta-1,6-dien-2-yl)-4-methylcyclohexanol | CC(=C)C(O)CCC(=C)[C@]1(O)CC[C@](C)(Br)[C@@H](Cl)C1 |
| 157 | RL149 | (2S,3aS,5aS,6R,8aS)-1,2,3a,6-tetramethyldecahydrocyclopenta[c]pentalen-1-ol | C[C@@H]3CC[C@]21[C@H]3CC[C@@]2(C)C[C@H](C)[C@]1(C)O |
| 158 | RL150 | (1R,2S,3R,5S,8S,9R)-2,3,5,9-tetramethyltricyclo[6.3.0.01,5]undecan-3-ol | C[C@@H]1CC[C@]23[C@H](C)[C@](C)(O)C[C@]3(C)CC[C@@H]12 |
| 159 | RL151 | (1S,2R,4R)-2-bromo-1-methyl-4-{3-[(1R,3S,6S)-6-methyl-3-(prop-1-en-2-yl)-7-  oxabicyclo[4.1.0]heptan-2-yl]prop-1-en-2-yl}cyclohexan-1-ol | C[C@@]23CC[C@H](C(\C)=C)C(C/C(=C)[C@@H]1CC[C@](C)(O)[C@H](Br)C1)[C@H]3O2 |
| 160 | RL152 | (1S,2R,4R)-2-bromo-1-methyl-4-{3-[(1S,3S,5R)-5-methyl-3-(prop-1-en-2-yl)-6-  oxabicyclo[3.1.1]heptan-2-yl]prop-1-en-2-yl}cyclohexan-1-ol | C=C(C)[C@H]3C[C@@]1(C)C[C@H](O1)C3C\C(=C)[C@@H]2CC[C@](C)(O)[C@H](Br)C2 |
| 161 | RL153 | (1R,7S,8R,9R)-8-chloro-7,9-dihydroxy-2,2,9-trimethyltricyclo[6.3.1.01,6]dodec-5-en-4-one | O=C2C[C@](C)(C)[C@@]13CC[C@@](C)(O)[C@@](Cl)(C1)[C@@H](O)C3=C2 |
| 162 | RL154 | (1R,8R,9R)-8,9-dichloro-2,2,9-trimethyltricyclo[6.3.1.01,6]dodec-5-ene-4,7-dione | O=C1C3=CC(=O)C[C@](C)(C)[C@]23C[C@]1(Cl)[C@](C)(Cl)CC2 |
| 163 | RL155 | (1R,8R,9S)-8,9-dichloro-2,2,9-trimethyltricyclo[6.3.1.01,6]dodec-5-ene-4,7-dione | O=C1C3=CC(=O)C[C@](C)(C)[C@]23C[C@]1(Cl)[C@@](C)(Cl)CC2 |
| 164 | RS001 | (1S,4R,4aS,4bS,8R,8aR,10aS)-1-bromo-8a-  (bromomethyl)-4,10a-dimethyl-8-(propan-2-yl)-1,2,3,4,4a,4b,5,8,8a,9,10,10a-  dodecahydrophenanthren-4-ol | CC(C)[C@@H]3C=C[C@@H2][C@@H]2[C@@]3(CC[C@]1(C)[C@@H](Br)CC[C@@](C)(O)[C@H]12)CBr |
| 165 | RS002 | (1S,3R,4S,4aS,4bS,5S,8R,8aS,10aS)-1-bromo-8a-(bromomethyl)-5-hydroperoxy-4,10a-dimethyl-8-(propan-2-yl)-1,2,3,4,4a,4b,5,8,8a,9,10,10a-dodecahydrophenanthrene-3,4-diol | CC(C)[C@@H]3C=C[C@H](OO)[C@@H]2[C@@]3(CC[C@]1(C)[C@@H](Br)C[C@@H](O)[C@@](C)(O)[C@H]12)CBr |
| 166 | RS003 | (1S,3S,4S,4aS,4bS,5S,8R,8aS,10aS)-1-bromo-8a-(bromomethyl)-5-hydroperoxy-4,10a-dimethyl-8-(propan-2-yl)-1,2,3,4,4a,4b,5,8,8a,9,10,10a-dodecahydrophenanthrene-3,4-diol | CC(C)[C@@H]3C=C[C@H](OO)[C@@H]2[C@@]3(CC[C@]1(C)[C@@H](Br)C[C@H](O)[C@@](C)(O)[C@H]12)CBr |
| 167 | RS004 | (1S,4R,4aS,4bS,5S,8R,8aS,10aS)-1-bromo-8a-(bromomethyl)-4,10a-dimethyl-8-(propan-2-yl)-1,2,3,4,4a,4b,5,8,8a,9,10,10a-dodecahydrophenanthrene-4,5-diol | CC(C)[C@@H]3C=C[C@H](O)[C@@H]2[C@@]3(CC[C@]1(C)[C@@H](Br)C[C@@H2][C@@](C)(O)[C@H]12)CBr |
| 168 | RS005 | (1S,3R,4S,4aS,4bS,8S,8aS,10aS)-1-bromo-8a-(bromomethyl)-4,10a-dimethyl-8-(propan-2-yl)-1,2,3,4,4a,4b,7,8,8a,9,10,10a-dodecahydrophenanthrene-3,4-diol | CC(C)[C@@H]3CC=C[C@@H]2[C@@]3(CC[C@]1(C)[C@@H](Br)C[C@@H](O)[C@@](C)(O)[C@H]12)CBr |
| 169 | RS006 | (1S,3S,4S,4aS,4bS,8S,8aS,10aS)-1-bromo-8a-(bromomethyl)-4,10a-dimethyl-8-(propan-2-yl)-1,2,3,4,4a,4b,7,8,8a,9,10,10a-dodecahydrophenanthrene-3,4-diol | CC(C)[C@@H]3CC=C[C@@H]2[C@@]3(CC[C@]1(C)[C@@H](Br)C[C@H](O)[C@@](C)(O)[C@H]12)CBr |
| 170 | RS007 | (1S,4R,4aS,4bS,8S,8aS,10aS)-1-bromo-8a-  (bromomethyl)-4,10a-dimethyl-8-(propan-2-yl)-1,2,3,4,4a,4b,7,8,8a,9,10,10a-  dodecahydrophenanthren-4-ol | CC(C)[C@@H]3CC=C[C@@H]2[C@@]3(CC[C@]1(C)[C@@H](Br)CC[C@@](C)(O)[C@H]12)CBr |
| 171 | RS008 | (1R,4S,4aS,4bS,8S,8aS,10aS)-8a-(bromomethyl)-1,4-dihydroxy-4,10a-dimethyl-8-(propan-2-yl)-1,2,3,4,4a,4b,7,8,8a,9,10,10a-dodecahydrophenanthren-3-one | CC(C)[C@@H]3CC=C[C@@H]2[C@@]3(CC[C@]1(C)[C@H](O)CC(=O)[C@@](C)(O)[C@H]12)CBr |
| 172 | RS009 | (4S,4aS,4bS,8S,8aS,10aR)-8a-(bromomethyl)-4-hydroxy-4,10a-dimethyl-8-(propan-2-yl)-3,4,4a,4b,7,8,8a,9,10,10a-decahydrophenanthren-3-one | CC(C)[C@@H]3CC=C[C@@H]2[C@@]3(CC[C@]1(C)C=CC(=O)[C@@](C)(O)[C@H]12)CBr |
| 173 | RL156 | (2R,3aR,5R,6S,8Z,10aR)-6-bromo-2-[(1R)-3-bromopropadienyl]-5-ethyl-2,3,3a,5,6,7,10,10a-octahydrofuro[3,2-b]oxonine | Br\C=C=C/[C@H]1C[C@H]2O[C@H](CC)[C@@H](Br)CC=CC[C@H]2O1 |
| 174 | RL157 | (2R,3R)-5-(3-bromopropadienyl)-2-[(2Z,5E)-octa-2,5-dien-1-yl]tetrahydrofuran-3-yl acetate | CC(=O)O[C@@H]1CC(O[C@@H]1C/C=C\C\C=C\CC)/C=C=C\Br |
| 175 | RL158 | (1S,2R,4aR,5R,8aS)-5-bromo-4a,8-dimethyl-2-(propan-2-yl)-1,2,3,4,4a,5,6,8a-octahydronaphthalen-1-ol | CC(C)[C@H]1CC[C@@]2(C)[C@H](Br)CC=C(C)[C@@H]2[C@H]1O |
| 176 | RL159 | 4-bromo-2-(5-bromo-3-hydroxy-2,6,6-trimethyloxan-2-yl)-5-chloro-5-methylcyclohexyl acetate | BrC1CC(O)C(C)(OC1(C)C)C2CC(Br)C(C)(Cl)CC2OC(C)=O |
| 177 | RL160 | 5-bromo-2-(5-bromo-4-chloro-2-hydroxy-4-  methylcyclohexyl)-2,6,6-trimethyloxan-3-yl acetate | BrC1CC(OC(C)=O)C(C)(OC1(C)C)C2CC(Br)C(C)(Cl)CC2O |
| 178 | RL161 | 2-[3-(acetyloxy)-5-bromo-2,6,6-trimethyloxan-2-yl]-4-bromo-5-chloro-5-methylcyclohexyl acetate | BrC1CC(OC(C)=O)C(C)(OC1(C)C)C2CC(Br)C(C)(Cl)CC2OC(C)=O |
| 179 | RL162 | 4-bromo-2-(5-bromo-2,6,6-trimethyloxan-2-yl)-5-chloro-5-methylcyclohexyl acetate | BrC1CCC(C)(OC1(C)C)C2CC(Br)C(C)(Cl)CC2OC(C)=O |
| 180 | RL163 | 5-bromo-2-(3-bromo-4-chloro-4-methylcyclohexyl)-2,6,6-trimethyloxan-3-ol | BrC1CC(O)C(C)(OC1(C)C)C2CC(Br)C(C)(Cl)CC2 |
| 181 | RL164 | 5-bromo-2-(3-bromo-4-chloro-4-methylcyclohexyl)-2,6,6-trimethyloxan-3-yl acetate | BrC1CC(OC(C)=O)C(C)(OC1(C)C)C2CC(Br)C(C)(Cl)CC2 |
| 182 | RL165 | 3-bromo-6-(3-bromo-4-chloro-4-methylcyclohexyl)-2,2,6-trimethyloxane | BrC1CCC(C)(OC1(C)C)C2CC(Br)C(C)(Cl)CC2 |
| 183 | RL166 | 3-bromo-6-(3-bromo-4-chloro-4-methylcyclohexyl)-2,2,6-trimethyloxane | BrC1CCC(C)(OC1(C)C)C2CCC(C)(Cl)C(Br)C2 |
| 184 | RL167 | 5-bromo-2-(4-bromo-3-chloro-4-methylcyclohexyl)-2,6,6-trimethyloxan-3-ol | BrC1CC(O)C(C)(OC1(C)C)C2CCC(Br)(C)C(Cl)C2 |
| 185 | RL168 | 3-bromo-6-(4-bromo-3-chloro-4-methylcyclohexyl)-2,2,6-trimethyloxane | BrC1CCC(C)(OC1(C)C)C2CCC(Br)(C)C(Cl)C2 |
| 186 | RL169 | (5S)-4-(3-bromo-4-chloro-4-methylcyclohexyl)-5-methoxy-5-methyl-2,5-dihydrofuran-2-one | C[C@]2(OC)OC(=O)C=C2C1CCC(C)(Cl)C(Br)C1 |
| 187 | RL170 | (5R)-4-(3-bromo-4-chloro-4-methylcyclohexyl)-5-  methoxy-5-methyl-2,5-dihydrofuran-2-one | C[C@@]2(OC)OC(=O)C=C2C1CCC(C)(Cl)C(Br)C1 |
| 188 | RL171 | (5S)-4-(3-bromo-4-chloro-4-methylcyclohexyl)-5-hydroxy-5-methyl-2,5-dihydrofuran-2-one | C[C@]2(O)OC(=O)C=C2C1CC(Br)C(C)(Cl)CC1 |
| 189 | RL172 | 2-(3-bromo-4-chloro-4-methylcyclohexyl)-2,6,6-trimethyl-3,6-dihydro-2H-pyran-3-one | CC1(Cl)CCC(CC1Br)C2(C)OC(C)(C)C=CC2=O |
| 190 | RG001 | 2,15-dimethyl-14-[(2R)-6-methylhept-5-en-2- yl]tetracyclo[8.7.0.0^{2,7}.0^{11,15}]heptadec-7- en-5-ol | C/C(C)=C\CC[C@@H](C)C1CCC2C3CC=C4CC(O)CCC4(C)C3CCC12C |
| 191 | RG002 | 14-[(2R)-4-(3,3-dimethyloxiran-2-yl)butan-2-yl]- 2,15- dimethyltetracyclo[8.7.0.0^{2,7}.0^{11,15}]heptadec-7- en-5-ol | OC3CC4=CCC2C5CCC([C@H](C)CCC1OC1(C)C)C5(C)CCC2C4(C)CC3 |
| 192 | RG003 | 14-[(2R)-5-hydroperoxy-6-methylhept-6-en-2-yl]- 2,15- dimethyltetracyclo[8.7.0.0^{2,7}.0^{11,15}]heptadec-7- en-5-ol | CC(C)[C@@H]3C=C[C@H](OO)[C@@H]2[C@@]3(CC[C@]1(C)[C@@H](Br)C[C@H](O)[C@@](C)(O)[C@H]12)CBr |
| 27 | RL143 | (3E,6S,7S,9Z,12E)-pentadeca-3,9,12-trien-1-yne-6,7-diyl diacetate | O=C(C)O[C@@H](C/C=C\C\C=C\CC)[C@@H](OC(=O)C)C\C=C\C#C |
| 193 | RG004 | 14-[(2R,4E)-6-hydroperoxy-6-methylhept-4-en-2-yl]- 2,15- dimethyltetracyclo[8.7.0.0^{2,7}.0^{11,15}]heptadec-7- en-5-ol | CC(C)(OO)/C=C/C[C@@H](C)C1CCC2C3CC=C4CC(O)CCC4(C)C3CCC12C |
| 194 | RG005 | 14-[(2R)-5-hydroxy-6-methylhept-6-en-2-yl]-2,15- dimethyltetracyclo[8.7.0.0^{2,7}.0^{11,15}]heptadec-7- en-5-ol | C=C(C)C(O)CC[C@@H](C)C1CCC2C3CC=C4CC(O)CCC4(C)C3CCC12C |
| 195 | RG006 | 14-[(2R,4E)-6-hydroxy-6-methylhept-4-en-2-yl]-2,15- dimethyltetracyclo[8.7.0.0^{2,7}.0^{11,15}]heptadec-7- en-5-ol | CC(C)(O)/C=C/C[C@@H](C)C1CCC2C3CC=C4CC(O)CCC4(C)C3CCC12C |
| 196 | RG007 | (6beta,24R)-6-hydroxy-24,25-epoxycholest-4-en-3-one | OC3CC2C5CCC([C@H](C)CCC1OC1(C)C)C5(C)CCC2C4(C)CCC(=O)C=C34 |
| 197 | RL173 | (2E)-3,7,11,15-tetramethylhexadec-2-en-1-ol | OC/C=C(/CCCC(CCCC(C)CCCC(C)C)C)C |
| 198 | RL174 | (2R,3R,6R,10S)-2,10-dibromo-3,7,11,11-tetramethylspiro[5.5]undec-7-en-3-ol | CC2=CC[C@H](Br)C(C)(C)[C@]12CC[C@@](C)(O)[C@H](Br)C1 |
| 199 | RL175 | (4S,6R,8R,9R)-4,8-dibromo-9-chloro-1,5,5,9-tetramethylspiro[5.5]undec-1-ene | CC2=CC[C@H](Br)[C@@](C)(C)[C@]12CC[C@@](C)(Cl)[C@H](Br)C1 |
| 200 | RL176 | (1S,5S,6R,8R,9R)-2,5,6,8-tetramethyl-7-oxatricyclo[6.2.2.01,6]dodec-2-en-9-ol | CC2=CC[C@H](C)[C@@]3(C)O[C@]1(C)CC[C@]23C[C@H]1O |
| 201 | RL177 | (2R,3S,6S)-2-bromo-1,1-dimethyl-5,9-dimethylidenespiro[5.5]undec-7-en-3-ol | C=C1CC[C@]2(C=C1)C(=C)C[C@H](O)[C@H](Br)C2(C)C |
| 202 | RL178 | (2R,3S,6S,9Z)-2-bromo-9-(bromomethylidene)-1,1-dimethyl-5-methylidenespiro[5.5]undec-7-en-3-ol | Br[C@H]=C1CC[C@]2(C=C1)C(=C)C[C@H](O)[C@H](Br)C2(C)C |
| 203 | RL179 | (2R,3S,6S,9E)-2-bromo-9-(bromomethylidene)-1,1-dimethyl-5-methylidenespiro[5.5]undec-7-en-3-ol | Br[C@H]=C1CC[C@]2(C=C1)C(=C)C[C@H](O)[C@H](Br)C2(C)C |
| 204 | RL180 | (1S,2R,3'S,4'S,6R)-3',4-dibromo-4'-chloro-1,3,3,4'-tetramethyl-7-oxaspiro[bicyclo[4.1.0]heptane-2,1'-cyclohexane]-4-ene | C[C@]1(Cl)CC[C@@]3(C[C@@H]1Br)[C@](C)(C)C(Br)=C[C@H]2O[C@]23C |
| 205 | RL181 | (6R,8R)-8-bromo-3,7,7-trimethyl-11-methylidenespiro[5.5]undec-2-ene | C=C2CC[C@@H](Br)[C@@](C)(C)[C@@]12CC=C(C)CC1 |
| 206 | RL182 | 1-[(1R,2S)-1,2-dimethyl-3-methylidenecyclopentyl]-4-methylcyclohexa-1,4-diene | C[C@H]1C(=C)CC[C@@]1(C)C1=CCC(C)=CC1 |
| 207 | RL183 | (1R,3S,4S,8R,11R)-3,8-dibromo-4-chloro-4,11,12,12- tetramethyl-7-oxatricyclo[6.3.1.0^{1,6}]dodec-9-en- 11-ol | O[C@]3(C)C=C[C@]2(Br)OC1C[C@](C)(Cl)[C@@H](Br)C[C@@]13[C@]2(C)C |
| 208 | RL184 | (1S,2R,4'S,5'S,6R)-5'-bromo-4'-chloro-1,3,3,4,4'-pentamethyl-7-oxaspiro[bicyclo[4.1.0]heptane-2,1'-cyclohexane]-4-en-2'-ol | C[C@@]3(C)C(C)=C[C@H]1O[C@@]1(C)[C@@]23C[C@H](Br)[C@@](C)(Cl)C[C@@H]2O |
| 209 | RL185 | (3beta,5beta,8alpha,10alpha,13alpha)-15-bromo-3,19-cyclopimar-9(11)-en-16-ol | Br[C@@H](CO)[C@]1(C)C[C@H]2CC[C@@H]3[C@@]4(C)C[C@H]4CC[C@@]3(C)C2=CC1 |
| 210 | RL186 | (2R)-2-bromo-2-[(1S,2S,5S,10R,11S,13R)-1,5,11- trimethyltetracyclo[8.5.0.0^{2,7}.0^{11,13}]pentadec-7- en-5-yl]ethan-1-ol | Br[C@@H](CO)[C@@]4(C)CC[C@H]2C(=CC[C@@H]1[C@@]3(C)C[C@H]3CC[C@]12C)C4 |
| 211 | RL187 | (1R,2S,8S,12R)-12-bromo-1,8,13,13-tetramethyl- 5,9,14- trioxatetracyclo[8.4.0.0^{2,6}.0^{4,8}]tetradecane | CC42OC(C)(C)C(Br)CC4OC3(C)CC1OC3CC12 |
| 212 | RL188 | (1S,7R,9R,10S,12S)-5-bromo-2,4,4,10-tetramethyl- 3,8,14- trioxatetracyclo[7.3.1.1^{7,10}.0^{2,7}]tetradecan- 12-ol | BrC3C[C@]14O[C@@]2(C)C[C@H](O)[C@H](C[C@H]2O1)C4(C)OC3(C)C |
| 213 | RL189 | (1R,7R,9S)-5-bromo-2,4,4,9-tetramethyl-3,8- dioxatricyclo[7.2.2.0^{2,7}]tridecan-10-ol | CC32OC(C)(C)C(Br)C[C@H]3O[C@@]1(C)CC[C@@H]2C[C@@H]1O |
| 214 | RL190 | (1S,7R,9S,11R)-5-bromo-13-hydroxy-2,4,4,9- tetramethyl-3,8- dioxatricyclo[7.2.2.0^{2,7}]tridecan-11-yl acetate | CC(=O)O[C@@H]3C[C@]2(C)O[C@@H]1CC(Br)C(C)(C)OC1(C)[C@H]3C[C@@H]2O |
| 215 | RL191 | 2-chloro-1,6,6-trimethyl-4-(propan-2-yl)hexahydro-1H-indene-1,3a,7a-triol | CC(C)C2CC(C)(C)CC1(O)C2(O)CC(Cl)C1(C)O |
| 216 | RL192 | (1S,3R,5S,6S,8S)-5-bromo-3-(1-bromopropyl)-8-[(2E)-pent-2-en-4-yn-1-yl]-2,7-dioxabicyclo[4.2.1]nonane | BrC(CC)[C@@H]2O[C@H]1C[C@H](O[C@H]1C\C=C\C#C)[C@@H](Br)C2 |
| 217 | RL193 | (2R,5S,7S)-2-(1-bromopropyl)-5,7-dichloro-8-[(2E)-pent-2-en-4-yn-1-yl]oxocan-4-yl acetate | CC(=O)OC1C[C@@H](OC(C\C=C\C#C)[C@@H](Cl)C[C@@H]1Cl)C(Br)CC |
| 218 | RL194 | (2R,3R,4R,5R,7S)-3-(bromomethyl)-5-chloro-7-[(3Z)-1-chlorohex-3-en-5-yn-1-yl]-2-ethyloxepan-4-ol | ClC(C/C=C\C#C)[C@@H]1C[C@@H](Cl)[C@H](O)[C@@H](CBr)[C@@H](CC)O1 |
| 219 | RL195 | (3S,6S)-6-bromo-6-[(5S)-5-bromo-2,6,6-trimethyltetrahydro-2H-pyran-2-yl]-3-methylhex-1-en-3-ol | C=C[C@@](C)(O)CC[C@H](Br)C1(C)CC[C@H](Br)[C@@](C)(C)O1 |
| 220 | RL196 | (2S,3R,4aR,6S,7S,8aR)-3-bromo-7-chloro-2-ethyl-6-[(2E)-pent-2-en-4-yn-1-yl]octahydropyrano[3,2-b]pyran | Cl[C@H]1C[C@H]2O[C@@H](CC)[C@H](Br)C[C@H]2O[C@H]1C\C=C\C#C |
| 221 | RL197 | (3R,5R,6R)-5-bromo-3-(1-bromopropyl)-8-[(E)-pent-2-en-4-ynyl]-2,7-dioxabicyclo[4.2.1]nonane | BrC(CC)[C@@H]2O[C@@H]1C[C@@H](O[C@@H]1C/C=C\C#C)[C@H](Br)C2 |
| 222 | RL198 | (1R,7S)-1,6:4,7-dianhydro-3-bromo-1-(1-bromopropyl)-2,3,5-trideoxy-7-[(2Z)-pent-2-en-4-yn-1-yl]-D-xylo-heptitol | BrC(CC)[C@@H]2O[C@H]1C[C@H](O[C@H]1C/C=C\C#C)[C@@H](Br)C2 |
| 223 | RL199 | 1,7:4,6-dianhydro-3-bromo-1-(1-bromopropyl)-2,3,5-trideoxy-7-[(2Z)-pent-2-en-4-yn-1-yl]heptitol | BrC(CC)[C@H]2C[C@@H](Br)[C@H]1C[C@H](O1)[C@H](C/C=C\C#C)O2 |
| 224 | RL200 | (2aR,3S,4aR,5R,6S,8S,8aS)-3,6-dibromo-2a,4a,5,8-tetramethyloctahydrocyclobuta[c]inden-7(8H)-one | C[C@@H]1C(=O)[C@@H](Br)[C@H](C)[C@@]2(C)C[C@H](Br)[C@]3(C)CC[C@]123 |
| 225 | RL201 | (2aR,3S,4aR,5R,6R,8R,8aS)-3,6-dibromo-2a,4a,5,8-tetramethyloctahydrocyclobuta[c]inden-7(8H)-one | C[C@H]1C(=O)[C@H](Br)[C@H](C)[C@@]2(C)C[C@H](Br)[C@]3(C)CC[C@]123 |
| 226 | RL202 | (2aR,3S,4aR,5R,6R,8R,8aS)-3-bromo-6-methoxy-2a,4a,5,8-tetramethyloctahydrocyclobuta[c]inden-7(8H)-one | C[C@H]1C(=O)[C@H](OC)[C@H](C)[C@@]2(C)C[C@H](Br)[C@]3(C)CC[C@]123 |
| 227 | RL203 | (2aR,3S,4aR,5S,8S,8aS)-3-bromo-2a,4a,5,8-tetramethyloctahydrocyclobuta[c]inden-7(8H)-one | C[C@@H]1C(=O)[C@H2][C@H](C)[C@@]2(C)C[C@H](Br)[C@]3(C)CC[C@]123 |
| 228 | RL204 | (2aR,3S,4aR,5S,8R,8aS)-3-bromo-2a,4a,5,8-tetramethyloctahydrocyclobuta[c]inden-7(8H)-one | C[C@H]1C(=O)[C@@H2][C@H](C)[C@@]2(C)C[C@H](Br)[C@]3(C)CC[C@]123 |
| 229 | RL205 | (3R,4R,4aR)-3-methoxy-1,4,4a,7-tetramethyl-3,4,4a,5,8,9-hexahydro-2H-benzo[7]annulen-2-one | O=C2C(C)=C1CCC(C)=CC[C@]1(C)[C@@H](C)[C@H]2OC |
| 230 | RL206 | (4S,4aR)-1,4,4a,7-tetramethyl-3,4,4a,5,8,9-hexahydro-2H-benzo[7]annulen-2-one | [H]C1[C@H](C)[C@@]2(C)CC=C(C)CCC2=C(C)C1=O |
| 231 | RL207 | (1R,2R,7S,8S,9aR)-2,8-dibromo-7-chloro-1,4,7,9a-tetramethyl-2,3,5,6,7,8,9,9a-octahydro-1H-benzo[7]annulen-3-ol | C[C@H]1[C@@H](Br)C(O)C(C)=C2CC[C@](C)(Cl)[C@@H](Br)C[C@]12C |
| 232 | RL208 | (1R,2R,7S,8S,9aR)-2,8-dibromo-7-chloro-1,4,7,9a-tetramethyl-2,3,5,6,7,8,9,9a-octahydro-1H-benzo[7]annulen-3-yl acetate | CC(=O)OC1[C@H](Br)[C@H](C)[C@@]2(C)C[C@H](Br)[C@@](C)(Cl)CCC2=C1C |
| 233 | RL209 | (1S,5S,6R,8R,9S)-9-bromo-2,5,6,8-tetramethyl-7-oxatricyclo[6.2.2.01,6]dodec-2-ene | CC2=CC[C@H](C)[C@@]3(C)O[C@]1(C)CC[C@]23C[C@@H]1Br |
| 234 | RL210 | 8-bromo-9-chloro-1,2,5,9-tetramethylspiro[5.5]undeca-1,4-diene | CC2=CCC(C)=C(C)C12CC(Br)C(C)(Cl)CC1 |
| 235 | RL211 | (4R,6S)-4,8-dibromo-9-chloro-1,5,5,9-tetramethylspiro[5.5]undec-1-ene | CC2=CC[C@@H](Br)[C@](C)(C)[C@@]12CCC(C)(Cl)C(Br)C1 |
| 236 | RL212 | (3S,4S,6S)-4,8-dibromo-9-chloro-1,5,5,9-tetramethylspiro[5.5]undec-1-en-3-ol | CC2=C[C@H](O)[C@@H](Br)[C@](C)(C)[C@@]12CCC(C)(Cl)C(Br)C1 |
| 237 | RL213 | (2R,6S)-2,8-dibromo-9-chloro-1,1,9-trimethyl-5-methylidenespiro[5.5]undecane | C=C2CC[C@@H](Br)[C@](C)(C)[C@@]12CCC(C)(Cl)C(Br)C1 |
| 238 | RL214 | (2R,6S)-2,9-dibromo-8-chloro-1,1,9-trimethyl-5-methylidenespiro[5.5]undecane | C=C2CC[C@@H](Br)[C@](C)(C)[C@@]12CCC(Br)(C)C(Cl)C1 |
| 239 | RL215 | (1S,2R,4R,6R)-3',4-dibromo-4'-chloro-1,3,3,4'-tetramethyl-7-oxaspiro[bicyclo[4.1.0]heptane-2,1'-cyclohexane] | CC1(Cl)CC[C@@]3(CC1Br)[C@@](C)(C)[C@H](Br)C[C@H]2O[C@]23C |
| 240 | RL216 | (6R)-4,10-dibromo-3-chloro-3,7,11,11-  tetramethylspiro[5.5]undeca-7,9-dien-1-ol | CC2=CC=C(Br)[C@@](C)(C)[C@]12CC(Br)C(C)(Cl)CC1O |
| 241 | RL217 | (3S,4S,8R,11R)-3,8-dibromo-4-chloro-4,11,12,12-tetramethyl-7-oxatricyclo[6.3.1.01,6]dodec-9-en-11-ol | O[C@]3(C)C=C[C@]2(Br)OC1C[C@](C)(Cl)[C@@H](Br)CC13[C@]2(C)C |
| 242 | RL218 | (6S,7S)-3,7-dibromo-6-chloro-6,8b,9,9-tetramethyloctahydro-4aH-3,8a-methanooxireno[d][1]benzoxepin-2-ol | C[C@@]3(C)C42C[C@H](Br)[C@@](C)(Cl)CC4O[C@@]3(Br)C(O)[C@H]1O[C@]12C |
| 243 | RL219 | (3Z,6Z,9Z,12Z)-pentadeca-3,6,9,12-tetraen-1-yne | CC\C=C/C\C=C/C/C=C\C/C=C\C#C |
| 244 | RL220 | (3E,6Z,9Z,12Z)-pentadeca-3,6,9,12-tetraen-1-yne | CC\C=C/C\C=C/C/C=C\C\C=C\C#C |
| 245 | RL221 | (2R,3S,5R,6S,8R)-3,6-dibromo-8-[(3Z)-1-chlorohex-3-en-5-yn-1-yl]-2-ethyloxocan-5-yl acetate | C#C\C=C/CC(Cl)[C@H]1C[C@H](Br)[C@@H](C[C@H](Br)[C@@H](CC)O1)OC(C)=O |
| 246 | RL222 | (2R,4R,5R,8R)-2-[(3Z)-1-chlorohex-3-en-5-yn-1-yl]-8-ethyl-3,4,5,8-tetrahydro-2H-oxocine-4,5-diol | C#C\C=C/CC(Cl)[C@H]1C[C@@H](O)[C@H](O)C=C[C@@H](CC)O1 |
| 247 | RL223 | (2R,4R,5R,8R)-5-(acetyloxy)-2-[(3Z)-1-chlorohex-3-en-5-yn-1-yl]-8-ethyl-3,4,5,8-tetrahydro-2H-oxocin-4-yl acetate | CC(=O)O[C@@H]1C=C[C@@H](CC)O[C@H](C[C@H]1OC(C)=O)C(Cl)C/C=C\C#C |
| 248 | RL224 | (2R,4R,5R,8R)-2-[(3Z)-1-chlorohex-3-en-5-yn-1-yl]-8-ethyl-4-hydroxy-3,4,5,8-tetrahydro-2H-oxocin-5-yl acetate | CC(=O)O[C@@H]1C=C[C@@H](CC)O[C@H](C[C@H]1O)C(Cl)C/C=C\C#C |
| 249 | RL225 | (2R,4R,5R,8R)-2-[(3Z)-1-chlorohex-3-en-5-yn-1-yl]-8-ethyl-5-hydroxy-3,4,5,8-tetrahydro-2H-oxocin-4-yl acetate | CC(=O)O[C@@H]1C[C@@H](O[C@@H](C=C[C@H]1O)CC)C(Cl)C/C=C\C#C |
| 250 | RL226 | (2R,4R,5R,8R)-4-bromo-2-[(3Z)-1-chlorohex-3-en-5-yn-1-yl]-8-ethyl-3,4,5,8-tetrahydro-2H-oxocin-5-yl acetate | CC(=O)O[C@@H]1C=C[C@@H](CC)O[C@H](C[C@H]1Br)C(Cl)C/C=C\C#C |
| 251 | RL227 | (6S)-10-hydroxy-2,2,10-trimethyl-8-oxatricyclo[7.3.1.01,6]tridec-11-ene-4,7-dione | O=C2OC1CC3(C=CC1(C)O)[C@@](C)(C)CC(=O)C[C@H]23 |
| 252 | RL228 | (1S,5S)-5,5'-dibromo-4'a,6,6,8'-tetramethyl-2-methylidene-octahydro-1'H-spiro[cyclohexane-1,2'-naphthalene]-7',8'-diol | C=C3CC[C@H](Br)[C@](C)(C)[C@]13CC2C(C)(O)C(O)CC(Br)C2(C)CC1 |
| 253 | RL229 | (6R,8S)-8-bromo-2-chloro-3,7,7-trimethyl-11-methylidenespiro[5.5]undec-2-ene | C=C2CC[C@H](Br)[C@@](C)(C)[C@@]12CC(Cl)=C(C)CC1 |
| 254 | RL230 | (2S,4aS,8R,8aS)-2-ethenyl-2,5,8,8a-tetramethyl-3,4,4a,7,8,8a-hexahydro-2H-chromene | C[C@]1(CC[C@H]2C(C)=CC[C@@H](C)[C@]2(C)O1)C=C |
| 255 | RL231 | (2S,3aR,6aS)-6-bromo-5-[(1Z,3Z)-4-bromohexa-1,3-dien-1-yl]-2-[(1S)-3-bromopropadienyl]-2,3,3a,6a-tetrahydrofuro[3,2-b]furan | [H][C@@]12C[C@H](O[C@]1([H])C(Br)=C(O2)\C=C/C=C(\Br)CC)C=C=CBr |
| 256 | RL232 | (3R,3'R,4'S)-3'-bromo-1-chloro-2',2',6-trimethyl-6'-methylidene-7-oxaspiro[bicyclo[4.1.0]heptane-3,1'-cyclohexan]-4'-ol | C=C3C[C@H](O)[C@H](Br)[C@@](C)(C)[C@]13CCC2(C)OC2(Cl)C1 |
| 257 | RL233 | (1R,3R,4S,8S)-3-bromo-4-hydroxy-2,2,8-trimethyltricyclo[6.2.2.01,6]dodec-6-en-9-one | O=C3C[C@]21CC[C@@]3(C)C=C2C[C@H](O)[C@H](Br)[C@]1(C)C |
| 258 | RL234 | (2R,3S,6R)-2-bromo-8-chloro-1,1,9-trimethyl-5-methylidenespiro[5.5]undec-8-en-3-ol | CC1=C(Cl)C[C@]2(CC1)C(=C)C[C@H](O)[C@H](Br)C2(C)C |
| 259 | RL235 | (2R,3S,6R)-2-bromo-1,1,9-trimethyl-5-methylidenespiro[5.5]undec-8-en-3-ol | CC1=CC[C@]2(CC1)C(=C)C[C@H](O)[C@H](Br)C2(C)C |
| 260 | RL236 | (6R,8S)-8-bromo-3,7,7-trimethyl-11-methylidenespiro[5.5]undec-2-ene | C=C2CC[C@H](Br)[C@@](C)(C)[C@@]12CC=C(C)CC1 |
| 261 | RL237 | (2R,6S)-2,8-dibromo-9-chloro-1,1,9-trimethyl-5-methylidenespiro[5.5]undecane | C=C2CC[C@@H](Br)[C@](C)(C)[C@@]12CCC(C)(Cl)C(Br)C1 |
| 262 | RL238 | (2R,6S)-2,9-dibromo-8-chloro-1,1,9-trimethyl-5-methylidenespiro[5.5]undecane | C=C2CC[C@@H](Br)[C@](C)(C)[C@@]12CCC(Br)(C)C(Cl)C1 |
| 263 | RL239 | (4S,6R)-4-bromo-1,5,5,9-tetramethylspiro[5.5]undeca-1,8-diene | CC2=CC[C@H](Br)[C@@](C)(C)[C@@]12CC=C(C)CC1 |
| 264 | RL240 | (1aS,4aR,5R,7bR)-3,3,5,7b-tetramethyldecahydro-4aH-cyclopropa[e]azulen-4a-ol | C[C@@]2(C)C[C@H]1C[C@@]1(C)C3CC[C@@H](C)[C@]3(O)C2 |
| 265 | RL241 | 2-(1-bromoethyl)-2,5-dimethyl-6-[(2E)-penta-2,4-dien-1-yl]tetrahydro-2H-pyran | Br[C@H](C)C1(C)CCC(C)C(C/C=C/C=C)O1 |
| 266 | RL242 | 3S,3aS,4R,7S,7aS)-7-bromo-3-{[(1S,4S)-4-bromo-1-hydroxy-3,3-dimethylcyclohexyl]methyl}-4,7a-dimethyl-octahydro-1H-inden-4-ol | C[C@]1(C)C[C@](O)(CC[C@@H]1Br)C[C@@H]2CC[C@]3(C)[C@@H](Br)CC[C@@](C)(O)[C@@H]23 |
| 267 | RL243 | (1S,4S)-4-bromo-1-{[(1S,4S,5S,6R,9S)-9-bromo-10- hydroxy-6-methyl-11- oxatricyclo[4.3.2.0^{1,5}]undecan-4-yl]methyl}-3,3- dimethylcyclohexyl acetate | CC(=O)O[C@]1(C[C@](C)(C)[C@@H](Br)CC1)C[C@@H]3CC[C@@]24[C@@H](Br)CC[C@@](C)(OC2O)[C@@H]34 |
| 268 | RL244 | (1S,1aS,3R,3aR,4S,6bS)-4-bromo-3,6-dimethyl-1-(propan-2-yl)-1a,2,3,4,5,6b-hexahydrocyclopropa[e]inden-3a(1H)-ol | CC(C)[C@@H]3[C@H]2C1=C(C)C[C@H](Br)[C@@]1(O)[C@H](C)C[C@H]23 |
| 269 | RL245 | (1S,1aS,2R,5S,5aS,6aS)-5-bromo-2,5a-dimethyl-1-(propan-2-yl)decahydrocyclopropa[a]inden-2-ol | CC(C)[C@@H]2[C@H]1C3[C@](C)(O)CC[C@H](Br)[C@@]3(C)C[C@H]12 |
| 270 | RL246 | (1S,8aR)-4,7-dimethyl-1-(propan-2-yl)-1,2,3,5,6,8a-hexahydronaphthalene | CC(C)[C@@H]2CCC(C)=C1CCC(C)=C[C@H]12 |
| 271 | RL247 | (1R,4S,4aR)-1,6-dimethyl-4-(propan-2-yl)-1,2,3,4,4a,7,8,8a-octahydronaphthalen-1-ol | CC(C)[C@@H]1CC[C@@](C)(O)C2CCC(C)=C[C@@H]12 |
| 272 | RL248 | 3,7-dimethyl-5-(2-methylpropyl)-1H-indene | CC(C)Cc2cc1c(CC=C1C)c(C)c2 |
| 273 | RL249 | (1S,1aS,6S,6aS,6bS)-3,6-dimethyl-1-(propan-2-yl)-1,1a,2,4,5,6,6a,6b-octahydrocyclopropa[e]inden-6-ol | CC(C)C3[C@@H]2CC(C)=C1CCC(C)(O)C1[C@@H]23 |
| 274 | RL250 | (4R,6R)-6-[(1R)-1-bromo-2-methylpropyl]-1,4-dimethyl-5,6-dihydro-4H-indene | CC(C)[C@@H](Br)[C@H]2C=C1C(C)=CC=C1[C@H](C)C2 |
| 275 | RL251 | (2S,3aR,5R,6S,8R,9R,10aR)-6-bromo-2-[(1R)-3-bromopropadienyl]-5-ethyldecahydrofuro[3,2-b]oxonine-8,9-diol | Br\C=C=C\[C@@H]1C[C@H]2O[C@H](CC)[C@@H](Br)C[C@@H](O)[C@H](O)C[C@H]2O1 |
| 276 | RL252 | (2S,3aR,5R,7S,8R,9aR)-2-(3-bromopropa-1,2-dien-1-yl)-5-(1-bromopropyl)-octahydro-2H-furo[3,2-b]oxocine-7,8-diol | BrC(CC)[C@H]1C[C@H](O)[C@H](O)C[C@H]2O[C@H](\C=C=C\Br)C[C@H]2O1 |
| 277 | RL253 | (1S,3R,4S,6R,10R)-4-bromo-3-ethyl-8-[(2Z)-pent-2-en-4-yn-1-yl]-2,7-dioxabicyclo[4.2.2]dec-10-yl hydroperoxide | C#C\C=C/C[C@H]2O[C@@H]1C[C@H](Br)[C@H](O[C@H]2C[C@H]1OO)CC |
| 278 | RL254 | (2S,3S,4aR,6S,7S,8aR)-3-bromo-7-chloro-2-ethyl-6-[(2Z)-pent-2-en-4-yn-1-yl]octahydropyrano[3,2-b]pyran | Cl[C@H]1C[C@H]2O[C@@H](CC)[C@@H](Br)C[C@H]2O[C@H]1C/C=C\C#C |
| 279 | RL255 | (2S,3S,4aR,6S,7S,8aR)-3,7-dibromo-2-ethyl-6-[(2Z)-pent-2-en-4-yn-1-yl]octahydropyrano[3,2-b]pyran | Br[C@H]1C[C@H]2O[C@@H](C/C=C\C#C)[C@@H](Br)C[C@H]2O[C@H]1CC |
| 280 | RL256 | (2S,3R,6S,9Z)-2-bromo-9-(bromomethylidene)-1,1-dimethyl-5-methylidenespiro[5.5]undec-7-en-3-ol | Br[C@H]=C1CC[C@]2(C=C1)C(=C)C[C@@H](O)[C@@H](Br)[C@@]2(C)C |
| 281 | RL257 | (6S,8S,9R)-8-bromo-9-hydroxy-7,7-dimethyl-11-methylidenespiro[5.5]undec-1-en-3-one | O=C1CC[C@]2(C=C1)C(=C)C[C@@H](O)[C@@H](Br)[C@@]2(C)C |
| 282 | RL258 | (2S,3R,6S)-2,8-dibromo-9-chloro-1,1,9-trimethyl-5-methylidenespiro[5.5]undecan-3-ol | C=C2C[C@@H](O)[C@@H](Br)[C@](C)(C)[C@@]12CCC(C)(Cl)C(Br)C1 |
| 283 | RL259 | (2S,3R,6S,9E)-2-bromo-9-(bromomethylidene)-1,1-dimethyl-5-methylidenespiro[5.5]undec-7-en-3-ol | Br[C@H]=C1CC[C@]2(C=C1)C(=C)C[C@@H](O)[C@@H](Br)[C@@]2(C)C |
| 284 | RL260 | (1R,2R,5S)-5-[(1S,4S)-4-bromo-3,3-dimethylcyclohexyl]-2-methylbicyclo[3.1.0]hexan-2-ol | C[C@]1(C)C[C@H](CC[C@@H]1Br)[C@@]23C[C@H]3[C@](C)(O)CC2 |
| 285 | RL261 | [4'-bromo-3',3'-dimethyl-1,1'-bi(cyclohexane)-1,3-dien-4-yl]methyl acetate | CC1(C)CC(CCC1Br)C2=CC=C(COC(C)=O)CC2 |
| 286 | RL262 | 4-(4-bromo-3,3-dimethylcyclohexyl)benzyl acetate | CC1(C)CC(CCC1Br)c2ccc(COC(C)=O)cc2 |
| 287 | RL263 | [(1S,4S)-4-(4-bromo-3,3-dimethylcyclohexyl)-2,3-dioxabicyclo[2.2.2]oct-5-en-1-yl]methyl acetate | BrC1CCC(CC1(C)C)[C@]23C=C[C@](COC(C)=O)(CC2)OO3 |
| 288 | RL264 | [4-(4-bromo-3,3-dimethylcyclohexyl)phenyl]methanol | CC1(C)CC(CCC1Br)c2ccc(CO)cc2 |
| 289 | RL265 | [(1S,4S)-4-(4-bromo-3,3-dimethylcyclohexyl)-2,3-dioxabicyclo[2.2.2]oct-5-en-1-yl]methanol | BrC1CCC(CC1(C)C)[C@]23C=C[C@](CO)(CC2)OO3 |
| 290 | RL266 | (1S,2R,4'S,5'S,6R)-4,5'-dibromo-4'-chloro-1,3,3,4'-tetramethyl-7-oxaspiro[bicyclo[4.1.0]heptane-2,1'-cyclohexane]-4-en-2'-ol | C[C@@]3(C)C(Br)=C[C@H]1O[C@@]1(C)[C@@]23C[C@H](Br)[C@@](C)(Cl)C[C@@H]2O |
| 291 | RL267 | (1R,1'R,2S,2'R,4S,4'S,5S,7'R)-5,7'-dibromo-4- chloro-4,4',6',6'-tetramethyl-3',8'- dioxaspiro[cyclohexane-1,5'- tricyclo[5.1.0.0^{2,4}]octane]-2-ol | C[C@]3(C)[C@@]1(C[C@H](Br)[C@@](C)(Cl)C[C@@H]1O)[C@]4(C)O[C@@H]4[C@H]2O[C@]23Br |
| 292 | RL268 | (1R,3S,4S,8R,11R)-3,8-dibromo-4-chloro-4,11,12,12-tetramethyl-7-oxatricyclo[6.3.1.01,6]dodec-9-en-11-ol | O[C@]3(C)C=C[C@]2(Br)O[C@H]1C[C@](C)(Cl)[C@@H](Br)C[C@@]13[C@]2(C)C |
| 293 | RL269 | (1S,3R,4S,6S,7S)-4-bromo-6-chloro-3-ethyl-9-[(2E)-pent-2-en-4-yn-1-yl]-2,8-dioxabicyclo[5.2.1]decane | C#C\C=C\CC2O[C@H]1C[C@@H]2O[C@H](CC)[C@@H](Br)C[C@@H]1Cl |
| 294 | RL270 | (2R,4S,5R,6aR,8S,9aR)-4-bromo-8-(3-bromopropa-1,2-dien-1-yl)-2-(1-bromopropyl)-decahydrocyclopenta[b]oxocin-5-ol | BrC(CC)[C@H]1C[C@H](Br)[C@H](O)C[C@H]2C[C@@H](C[C@H]2O1)/C=C=[C@H]Br |
| 295 | RL271 | (2R,3R,5R)-5-(1-bromopropyl)-3-chloro-2-[(2Z,5E)-octa-2,5-dien-7-yn-1-yl]oxolane | Cl[C@@H]1C[C@@H](O[C@@H]1C/C=C\C\C=C\C#C)C(Br)CC |
| 296 | RL272 | (5E)-1-[(2R,3R,5R,6S)-5-bromo-3-chloro-6-ethyltetrahydro-2H-pyran-2-yl]-3-chlorooct-5-en-7-yn-2-yl acetate | Br[C@@H]1C[C@@H](Cl)[C@@H](CC(OC(C)=O)C(Cl)C\C=C\C#C)O[C@H]1CC |
| 297 | RL273 | (2R,3R,5S,6S)-3-bromo-2-[(2E)-3-bromopent-2-en-1-yl]-5-chloro-6-[(2Z)-pent-2-en-4-yn-1-yl]tetrahydro-2H-pyran | Br\C(=C\C[C@H]1O[C@@H](C/C=C\C#C)[C@@H](Cl)C[C@H]1Br)CC |
| 298 | RL274 | (5E)-5-(1-bromopropylidene)-9-[(1S,2Z)-1-chloropent-2-en-4-yn-1-yl]-4,8-dioxatricyclo[4.2.1.03,7]nonane | C#C\C=C/[C@H](Cl)C3C1CC2O\C(=C(\Br)CC)C3C2O1 |
| 299 | RL275 | (3R,4R,6R)-4-bromo-3-chloro-3,7,7-trimethyl-11-methylidene-10-oxospiro[5.5]undec-8-en-1-yl acetate | CC(=O)O[C@H]1C[C@@](C)(Cl)[C@H](Br)C[C@@]12C(=C)C(=O)C=C[C@]2(C)C |
| 300 | RL276 | (2S,3R,6S)-2-bromo-1,1,9-trimethyl-5-methylidenespiro[5.5]undec-8-en-3-ol | CC=1CC[C@]2(CC=1)C(=C)C[C@@H](O)[C@@H](Br)[C@@]2(C)C |
| 301 | RL277 | (2S,3R,6S,9Z)-2-bromo-9-(bromomethylidene)-1,1-dimethyl-5-methylidenespiro[5.5]undec-7-en-3-ol | Br[C@H]=C1CC[C@]2(C=C1)C(=C)C[C@@H](O)[C@@H](Br)[C@@]2(C)C |
| 302 | RL278 | (2S,3R,6S,9E)-2-bromo-9-(bromomethylidene)-1,1-dimethyl-5-methylidenespiro[5.5]undec-7-en-3-ol | Br[C@H]=C1CC[C@]2(C=C1)C(=C)C[C@@H](O)[C@@H](Br)[C@@]2(C)C |
| 303 | RL279 | 4-bromo-2-[(1S,2R,5R)-1,2-dimethylbicyclo[3.1.0]hex-2-yl]-5-methylphenol | C[C@]2(CC[C@@H]1C[C@@]12C)c3cc(Br)c(C)cc3O |
| 304 | RL280 | 4-bromo-2-[(1R,3S)-1,3-dimethyl-2-methylidenecyclopentyl]-5-methylphenol | C[C@]1(CC[C@H](C)C1=C)c2cc(Br)c(C)cc2O |
| 305 | RL281 | (1R,2R,3R,4S)-3-{2-[(1R,3R,4S)-3-bromo-4-hydroxy-4-methylcyclohexyl]prop-2-en-1-yl}-1-methyl-4-(prop-1-en-2-yl)cyclohexane-1,2-diol | C[C@]1(O)CC[C@H](C[C@H]1Br)C(/C[C@@H]2[C@H](CC[C@@](C)(O)[C@@H]2O)C(=C)C)=C |
| 306 | RL282 | (1S,2S,3R,4S)-3-{2-[(1R,3R,4S)-3-bromo-4-hydroxy-4-methylcyclohexyl]prop-2-en-1-yl}-1-methyl-4-(prop-1-en-2-yl)cyclohexane-1,2-diol | C[C@]1(O)CC[C@H](C[C@H]1Br)C(/C[C@@H]2[C@H](CC[C@](C)(O)[C@H]2O)C(=C)C)=C |
| 307 | RL283 |  | C[C@]1(O)CC[C@H](C[C@H]1Br)[C@]3(C)CC(C)=C2CC[C@](C)(O)[C@@H](O)C[C@H]23 |
| 308 | RL284 | (3S,3aR,5S,6S,8aS)-3-[(1R,3R,4S)-3-bromo-4-hydroxy-4-methylcyclohexyl]-3,6-dimethyl-1-methylidene-decahydroazulene-5,6-diol | C[C@]1(O)CC[C@H](C[C@H]1Br)[C@]3(C)CC(=C)[C@H]2CC[C@](C)(O)[C@@H](O)C[C@H]23 |
| 309 | RL285 | (1R,3aR,5S,6aR)-6-bromo-5-{1-[(1R,3R,4S)-3-bromo-4-hydroxy-4-methylcyclohexyl]ethenyl}-1,4,4-trimethyl-octahydropentalen-1-ol | C[C@]1(O)CC[C@H](C[C@H]1Br)\C(=C)[C@H]3C(Br)[C@@H]2[C@@H](CC[C@@]2(C)O)[C@]3(C)C |
| 310 | RL286 | (1S,3aR,5S,6aR)-6-bromo-5-{1-[(1R,3R,4S)-3-bromo-4-hydroxy-4-methylcyclohexyl]ethenyl}-1,4,4-trimethyl-octahydropentalen-1-ol | C[C@]1(O)CC[C@H](C[C@H]1Br)\C(=C)[C@H]3C(Br)[C@@H]2[C@@H](CC[C@]2(C)O)[C@]3(C)C |
| 311 | RL287 | (1S,2R,4R)-2-bromo-4-[(2E,4E)-5-[(1R,4R)-4-bromo-1,3,3-trimethylcyclohexyl]penta-2,4-dien-2-yl]-1-methylcyclohexan-1-ol | C[C@]1(O)CC[C@H](C[C@H]1Br)C(\C)=C\C=C\[C@]2(C)CC[C@@H](Br)[C@](C)(C)C2 |
| 312 | RL288 | (5Z)-1-[(2R,3R,5S,6R)-5-bromo-6-ethyl-3-hydroxytetrahydro-2H-pyran-2-yl]oct-5-en-7-yn-2-one | Br[C@H]1C[C@@H](O)[C@@H](CC(=O)CC/C=C\C#C)O[C@@H]1CC |
| 313 | RL289 | (2R,4aS,6S,8aS)-6-bromo-2-ethenyl-2,5,5,8a-tetramethyloctahydro-2H-chromene | C=C[C@@]1(C)CC[C@H]2[C@](C)(C)[C@@H](Br)CC[C@]2(C)O1 |
| 314 | RL290 | (3S,4R,7S,7aS)-7-bromo-4,7a-dimethyl-3-(2-methylprop-1-en-1-yl)octahydro-1H-inden-4-ol | C/C(C)=C\[C@@H]1CC[C@@]2(C)C1[C@](C)(O)CC[C@@H]2Br |
| 315 | RL291 | (6S,7aR)-3-(2-hydroperoxypropan-2-yl)-7-[(3R)-3-hydroxy-3-methylpent-4-en-1-yl]-6,7a-dimethyl-3a,4,5,6,7,7a-hexahydro-1H-inden-7-ol | CC(C)(OO)C2=CC[C@]1(C)C2CC[C@H](C)[C@@]1(O)CC[C@@](C)(O)C=C |
| 316 | RL292 | (1R,4S,6R,8R,9R)-8-bromo-9-chloro-1,4,9-trimethyl-5-methylidenespiro[5.5]undecan-1-ol | C=C2[C@@H](C)CC[C@@](C)(O)[C@]12CC[C@@](C)(Cl)[C@H](Br)C1 |
| 317 | RL293 | (2R,3R,3'R,4'R)-3'-bromo-4'-chloro-1,3,4,4'-tetramethyl-7-oxaspiro[bicyclo[2.2.1]heptane-2,1'-cyclohexane] | C[C@@]1(Cl)CC[C@@]3(C[C@H]1Br)[C@@H](C)[C@]2(C)CC[C@@]3(C)O2 |
| 318 | RL294 | (1S,4S,6S,7S)-4-bromo-6-chloro-3-ethyl-9-(pent-2-en-4-yn-1-yl)-2,8-dioxabicyclo[5.2.1]decane | C#C\C=C\CC2O[C@H]1C[C@@H]2O[C@H](CC)[C@@H](Br)C[C@@H]1Cl |
| 319 | RL295 | (2R,3'R,4'R)-3'-bromo-4'-chloro-1,3,3,4'-tetramethyl-7-oxaspiro[bicyclo[2.2.1]heptane-2,1'-cyclohexane] | C[C@@]1(Cl)CC[C@]3(C[C@H]1Br)[C@@]2(C)CC[C@H](O2)[C@]3(C)C |
| 320 | RL296 | (2R,4'R,5'R)-5'-bromo-4'-chloro-1,3,3,4'-tetramethyl-7-oxaspiro[bicyclo[2.2.1]heptane-2,1'-cyclohexan]-2'-yl acetate | CC(=O)O[C@@H]1C[C@@](C)(Cl)[C@H](Br)C[C@@]13[C@](C)(C)[C@@H]2CC[C@@]3(C)O2 |
| 321 | RL297 | (4S,4'S,5R,5'R)-4,4'-dibromo-5-ethyl-5'-[(2Z)-pent-2-en-4-yn-1-yl]octahydro-2,2'-bifuran | Br[C@H]1CC(O[C@@H]1CC)C2C[C@H](Br)[C@@H](C/C=C\C#C)O2 |
| 322 | RL298 | 7-bromo-4,8,16,16-tetramethyl-15-methylidene-11- oxatetracyclo[10.3.1.0^{1,10}.0^{3,8}]hexadecan-4- ol | CC1(O)CCC(Br)C2(C)CC3OC4CC\C(=C)C3(CC12)C4(C)C |
| 323 | RL299 | (3R)-3,5'-dibromo-2,2,4'a,8'-tetramethyl-6-methylidene-octahydro-1'H-spiro[cyclohexane-1,2'-naphthalene]-8'-ol | CC12CCC3(CC2C(C)(O)CCC1Br)C(/CC[C@@H](Br)C3(C)C)=C |
| 324 | RL300 | (1R,2S,5S,7R,10R,13S,15R)-5-bromo-2,6,6,10,15- pentamethyl-11,14- dioxatetracyclo[8.7.0.0^{2,7}.0^{13,15}]heptadecane | C[C@@]24CC[C@H](Br)[C@@](C)(C)[C@@H]4CC[C@@]1(C)OC[C@@H]3O[C@]3(C)CC[C@@H]12 |
| 325 | RL301 | (1R,2S,5S,7R,10R,12S,13R)-5-bromo-12- (chloromethyl)-2,6,6,10,13-pentamethyl-11- oxatricyclo[8.5.0.0^{2,7}]pentadecan-13-ol | C[C@@]23CC[C@H](Br)[C@@](C)(C)[C@@H]3CC[C@@]1(C)O[C@H](CCl)[C@](C)(O)CC[C@@H]12 |
| 326 | RL302 | (3Z)-1-bromo-5-(2,2-dimethyl-6-methylidenecyclohex-3-en-1-yl)-3-methylpent-3-en-2-ol | CC1(C)C=CCC(=C)C1C\C=C(\C)C(O)CBr |
| 327 | RL303 | (2S,5aS,7S,9aS)-7-bromo-2-ethyl-3,6,6,9a-tetramethyl-2,5,5a,6,7,8,9,9a-octahydro-1-benzoxepine | CC2(C)[C@@H](Br)CC[C@]1(C)O[C@@H](CC)C(C)=CC[C@H]12 |
| 328 | RL304 | (3aR,4aS,7S,8aS)-7-bromo-4a,8,8-trimethyl-1-methylidene-1,3,3a,4a,5,6,7,8,8a,9-decahydrofuro[3,4-b][1]benzoxepine | CC1(C)[C@@H](Br)CC[C@]2(C)O[C@H]3COC(=C)C3=CC[C@@H]12 |
| 329 | RL305 | (1S,3aS,4R,5S,7aR)-1,5-dimethyl-4-(2-methylprop-1-en-1-yl)octahydro-3aH-inden-3a-ol | C/C(C)=C\[C@H]1[C@@H](C)CC[C@@H]2[C@@H](C)CC[C@@]12O |
| 330 | RL306 | (3aR,4aS,7S,8aS)-7-bromo-4a,8,8-trimethyl-3a,4a,5,6,7,8,8a,9-octahydrofuro[3,4-b][1]benzoxepin-1(3H)-one | CC1(C)[C@@H](Br)CC[C@]2(C)O[C@H]3COC(=O)C3=CC[C@@H]12 |
| 331 | RL307 | (3E)-1-bromo-5-(2,2-dimethyl-6-methylidenecyclohex-3-en-1-yl)-3-methylpent-3-en-2-ol | CC1(C)C=CCC(=C)C1C\C=C(/C)C(O)CBr |
| 332 | RL308 | (3Z)-1-bromo-5-[(1R,3S)-3-bromo-2,2-dimethyl-6-methylidenecyclohexyl]-3-methylpent-3-en-2-ol | CC1(C)[C@H](C\C=C(\C)C(O)CBr)C(=C)CC[C@@H]1Br |
| 333 | RL309 | (3Z)-1-bromo-5-[(1R,3S)-3-bromo-2,2-dimethyl-6-methylidenecyclohexyl]-3-methylpent-3-en-2-yl acetate | CC1(C)[C@H](C\C=C(\C)C(CBr)OC(C)=O)C(=C)CC[C@@H]1Br |
| 334 | RL310 | (2R,6S)-6-bromo-1,1-dimethyl-3-methylidene-2-[(2Z)-3-methylpenta-2,4-dien-1-yl]cyclohexane | CC1(C)[C@H](C\C=C(\C)C=C)C(=C)CC[C@@H]1Br |
| 335 | RL311 | (3Z,6E)-1-bromo-3,7,11-trimethyldodeca-3,6,10-trien-2-ol | C\C(=C\C\C=C(/C)CC\C=C(/C)C)C(O)CBr |
| 336 | RL312 | (1S,2R,5S,7R,10S,12R,14S)-5-bromo-2,6,6,10,14- pentamethyl-13- oxatetracyclo[8.6.0.0^{2,7}.0^{12,14}]hexadecane | C[C@]24CC[C@H]1[C@](C)(C)[C@@H](Br)CC[C@]1(C)[C@H]4CC[C@]3(C)O[C@@H]3C2 |
| 337 | RL313 | 8-bromo-9-hydroxy-7,7-dimethyl-11-methylidenespiro[5.5]undec-1-en-3-one | O=C1CCC2(C=C1)C(=C)CC(O)C(Br)C2(C)C |
| 338 | RL314 | (3R)-8-bromo-3,7,7-trimethyl-11-methylidenespiro[5.5]undec-1-ene-3,9-diol | C[C@@]1(O)CCC2(C=C1)C(=C)CC(O)C(Br)C2(C)C |
| 339 | RL315 | (1R,2S,5R,7S,9S,10S,12R)-12-bromo-5-ethenyl- 1,5,7,11,11-pentamethyl-6- oxatricyclo[8.4.0.0^{2,7}]tetradecan-9-ol | C[C@]2(C)[C@H](Br)CC[C@@]1(C)[C@@H]3CC[C@](C)(C=C)O[C@@]3(C)C[C@H](O)[C@@H]12 |
| 340 | RL316 | (2S,3S,5Z,8S)-8-[(1R)-1-bromopropyl]-3-chloro-2-[(2Z)-pent-2-en-4-yn-1-yl]-3,4,7,8-tetrahydro-2H-oxocine | Br[C@H](CC)[C@@H]1CC=CC[C@H](Cl)[C@H](C/C=C\C#C)O1 |
| 341 | RL317 | (2R,4S,5S,7S,8S)-2-(1-bromopropyl)-5,7-dichloro-8-[(2Z)-pent-2-en-4-yn-1-yl]oxocan-4-yl acetate | CC(=O)O[C@H]1C[C@@H](O[C@@H](C/C=C\C#C)[C@@H](Cl)C[C@@H]1Cl)[C@@H](Br)CC |
| 342 | RL318 | (2R,4S,5S,7S,8S)-2-(1-bromopropyl)-5,7-dichloro-8-[(2E)-pent-2-en-4-yn-1-yl]oxocan-4-yl acetate | CC(=O)O[C@H]1C[C@@H](O[C@@H](C\C=C\C#C)[C@@H](Cl)C[C@@H]1Cl)[C@@H](Br)CC |
| 343 | RL319 | (2S,3R,5R)-2-{(R)-bromo[(2S,3S,5R,6S)-3,5-dibromo-6-ethyltetrahydro-2H-pyran-2-yl]methyl}-5-[(1S)-1-bromoprop-2-yn-1-yl]tetrahydrofuran-3-ol | O[C@@H]1C[C@@H](O[C@@H]1[C@@H](Br)[C@H]2O[C@@H](CC)[C@H](Br)C[C@@H]2Br)[C@@H](Br)C#C |
| 344 | RL320 | (1R,5S,7aS)-1,6,6-trimethyl-4-(propan-2-yl)-2,3,5,6,7,7a-hexahydro-1H-inden-5-ol | CC(C)C1=C2CC[C@@H](C)[C@@H]2C[C@](C)(C)[C@@H]1O |
| 345 | RL321 | (3R,4R)-3,5,5-trimethyl-7-(propan-2-yl)-2,3,4,5,6,7-hexahydro-1H-inden-4-ol | CC(C)C2C[C@@](C)(C)[C@@H](O)C1=C2CC[C@H]1C |
| 346 | RL322 | (2S,2'R)-2,2'-[(2R,2'S,2''S,5R,5'R,5''S)-2,2''-dimethyldodecahydro-2,2':5',2''-terfuran-5,5''-diyl]bis(6-methylhept-5-en-2-ol) | C[C@]1(CC[C@H](O1)[C@@](O)(C)CC\C=C(/C)C)[C@H]2CC[C@H](O2)[C@@]3(C)CC[C@@H](O3)[C@@](C)(O)CC\C=C(/C)C |
| 347 | RL323 | 2-[(2R,5R)-5-[(1S,4S)-4-[(2R,4aS,6R,8aR)-2-[(2S,5R)-5-bromo-2,6, 6-trimethyloxan-2-yl]-4a-methyl-3,4,6,7,8,8a-hexahydro-2H-pyrano[3, 2-b]pyran-6-yl]-1,4-dihydroxypentyl]-5-methyloxolan-2-yl]propan-2-yl acetate | BrC1CC[C@@](C)(O[C@@]1(C)C)[C@@H]2CC[C@@]3(C)OC(CC[C@@H]3O2)[C@@](C)(O)CC[C@@H](O)[C@@]4(C)CC[C@@H](O4)C(C)(C)OC(C)=O |
| 348 | RL324 | (1R,4S)-4-[(4aS,6S,8aR)-6-[(2R)-5-bromo-2,6,6-trimethyloxan-2-yl]-8a-methyl-octahydropyrano[3,2-b]pyran-2-yl]-1-[(2R,5R)-5-(2-hydroxypropan-2-yl)-2-methyloxolan-2-yl]pentane-1,4-diol | BrC1CC[C@@](C)(O[C@@]1(C)C)[C@@H]2CC[C@@]3(C)OC(CC[C@@H]3O2)[C@@](C)(O)CC[C@@H](O)[C@@]4(C)CC[C@@H](O4)C(C)(C)O |
| 349 | RL325 | (1R,4S)-4-[(4aS,6S,8aR)-6-[(2R)-5-bromo-2,6,6-trimethyloxan-2-yl]-8a-methyl-octahydropyrano[3,2-b]pyran-2-yl]-1-[(2R,5R)-5-[2-(acetyloxy)propan-2-yl]-2-methyloxolan-2-yl]-4-hydroxypentyl acetate | BrC1CC[C@@](C)(O[C@@]1(C)C)[C@@H]2CC[C@@]3(C)OC(CC[C@@H]3O2)[C@@](C)(O)CC[C@@H](OC(C)=O)[C@@]4(C)CC[C@@H](O4)C(C)(C)OC(C)=O |
| 350 | RL326 | (1R,4S)-4-[(4aS,6S,8aR)-6-[(2R)-5-bromo-2,6,6-trimethyloxan-2-yl]-8a-methyl-octahydropyrano[3,2-b]pyran-2-yl]-4-hydroxy-1-[(2R,5R)-5-(2-hydroxypropan-2-yl)-2-methyloxolan-2-yl]pentyl acetate | BrC1CC[C@@](C)(O[C@@]1(C)C)[C@@H]2CC[C@@]3(C)OC(CC[C@@H]3O2)[C@@](C)(O)CC[C@@H](OC(C)=O)[C@@]4(C)CC[C@@H](O4)C(C)(C)O |
| 351 | RL327 | (2S,2'S,4S,4'S,5S,5'S)-5-ethyl-5'-[(2E)-pent-2-en-4-yn-1-yl]octahydro-2,2'-bifuran-4,4'-diol | O[C@H]1C[C@H](O[C@H]1CC)[C@@H]2C[C@H](O)[C@H](C\C=C\C#C)O2 |
| 352 | RL328 | (1R,3S,4R,6S,7R,9R)-3-ethyl-9-[(2E)-pent-2-en-4-yn-1-yl]-2,8-dioxabicyclo[5.2.1]decane-4,6-diol | C#C\C=C\C[C@H]2O[C@@H]1C[C@H]2O[C@@H](CC)[C@H](O)C[C@@H]1O |
| 353 | RL329 | (1R,3S,4R,6S,7R,9R)-4-bromo-3-ethyl-9-[(2E)-pent-2-en-4-yn-1-yl]-2,8-dioxabicyclo[5.2.1]decan-6-ol | C#C\C=C\C[C@H]2O[C@@H]1C[C@H]2O[C@@H](CC)[C@H](Br)C[C@@H]1O |
| 354 | RL330 | (1R,4S)-4-[(2R,4aR,6R,8aS)-6-[(2S,5R)-5-bromo-2,6,6-trimethyloxan-2-yl]-8a-methyl-octahydropyrano[3,2-b]pyran-2-yl]-1-[(2S,5R)-5-(2-hydroxypropan-2-yl)-2-methyloxolan-2-yl]pentane-1,4-diol | Br[C@@H]1CC[C@](C)(O[C@]1(C)C)[C@H]2CC[C@]3(C)O[C@H](CC[C@H]3O2)[C@@](C)(O)CC[C@@H](O)[C@]4(C)CC[C@@H](O4)C(C)(C)O |
| 355 | RL331 | (1S,4S)-4-[(2R,4aR,6R,8aS)-6-[(2S,5R)-5-bromo-2,6,6-trimethyloxan-2-yl]-8a-methyl-octahydropyrano[3,2-b]pyran-2-yl]-1-[(2R,5R)-5-(2-hydroxypropan-2-yl)-2-methyloxolan-2-yl]pentane-1,4-diol | Br[C@@H]1CC[C@](C)(O[C@]1(C)C)[C@H]2CC[C@]3(C)O[C@H](CC[C@H]3O2)[C@@](C)(O)CC[C@H](O)[C@@]4(C)CC[C@@H](O4)C(C)(C)O |
| 356 | RL332 | 2-[(2R,5R)-5-[(1S,4S)-4-[(2R,4aR,6R,8aS)-6-[(2S,5R)-5-bromo-2,6,6-trimethyloxan-2-yl]-8a-methyl octahydropyrano[3,2-b]pyran-2-yl]-1,4-  dihydroxypentyl]-5-methyloxolan-2-yl]propan-2-yl acetate | Br[C@@H]1CC[C@](C)(O[C@]1(C)C)[C@H]2CC[C@]3(C)O[C@H](CC[C@H]3O2)[C@@](C)(O)CC[C@H](O)[C@@]4(C)CC[C@@H](O4)C(C)(C)OC(C)=O |
| 357 | RL333 | 5-(hydroxymethyl)furan-2-carbaldehyde | O=Cc1ccc(CO)o1 |
| 358 | RL334 | (1S)-3-methyl-6-{4-[(1R,2R,3R,4S)-1,3,4-trimethyl-7-oxabicyclo[2.2.1]hept-2-yl]butan-2-yl}cyclohex-2-en-1-ol | C[C@]13CC[C@](C)(O1)[C@H](C)[C@H]3CCC(C)C2CCC(C)=C[C@H]2O |
| 359 | RL335 | (2S,3R,6S)-2-bromo-9-(bromomethylidene)-1,1-dimethyl-5-methylidenespiro[5.5]undec-7-en-3-ol | Br[C@H]=C1CC[C@]2(C=C1)C(=C)C[C@@H](O)[C@@H](Br)[C@@]2(C)C |
| 360 | RL336 | (2S,3R,6S)-2-bromo-9-(bromomethylidene)-1,1-dimethyl-5-methylidenespiro[5.5]undec-7-en-3-ol | Br[C@H]=C1CC[C@]2(C=C1)C(=C)C[C@@H](O)[C@@H](Br)[C@@]2(C)C |
| 361 | RL337 | (2S,3R,6S)-2,8-dibromo-9-chloro-1,1,9-trimethyl-5-methylidenespiro[5.5]undecan-3-ol | C=C2C[C@@H](O)[C@@H](Br)[C@](C)(C)[C@@]12CCC(C)(Cl)C(Br)C1 |
| 362 | RL338 | (6S,8S,9R)-8-bromo-9-hydroxy-7,7-dimethyl-11-methylidenespiro[5.5]undec-1-en-3-one | O=C1CC[C@]2(C=C1)C(=C)C[C@@H](O)[C@@H](Br)[C@@]2(C)C |
| 363 | RL339 | (6R)-8-bromo-9-chloro-1,5,5,9-tetramethylspiro[5.5]undec-1-en-3-one | CC2=CC(=O)C[C@@](C)(C)[C@@]12CCC(C)(Cl)C(Br)C1 |
| 364 | RL340 | (6S,8S,9R)-8-bromo-3-(dibromomethyl)-7,7-dimethyl-11-methylidenespiro[5.5]undec-1-ene-3,9-diol | BrC(Br)C1(O)CC[C@]2(C=C1)C(=C)C[C@@H](O)[C@@H](Br)[C@@]2(C)C |
| 365 | RL341 | 4-bromo-2-[(1R,3S)-3-hydroxy-1,3-dimethyl-2-methylidenecyclopentyl]-5-methylphenol | C[C@]1(CC[C@](C)(O)C1=C)c2cc(Br)c(C)cc2O |
| 366 | RL342 | 4-bromo-2-[(1R,3R)-3-hydroxy-1,3-dimethyl-2-methylidenecyclopentyl]-5-methylphenol | C[C@]1(CC[C@@](C)(O)C1=C)c2cc(Br)c(C)cc2O |
| 367 | RL343 | 2-[(1R,3S)-3-hydroxy-1,3-dimethyl-2-methylidenecyclopentyl]-5-methylphenol | C[C@]1(CC[C@](C)(O)C1=C)c2ccc(C)cc2O |
| 368 | RL344 | 2-[(1R,3R)-3-hydroxy-1,3-dimethyl-2-methylidenecyclopentyl]-5-methylphenol | C[C@]1(CC[C@@](C)(O)C1=C)c2ccc(C)cc2O |
| 369 | RL345 | 4-bromo-2-[(1R,3S)-1,3-dimethyl-2-methylidenecyclopentyl]-5-methylphenol | C[C@]1(CC[C@H](C)C1=C)c2cc(Br)c(C)cc2O |
| 370 | RL346 | 5-methyl-2-[(1R)-1,2,3-trimethylcyclopent-2-en-1-yl]phenol | C[C@]1(CCC(C)=C1C)c2ccc(C)cc2O |
| 371 | RL347 | 2-[(1S,3S)-3-bromo-1,2,2-trimethylcyclopentyl]-5-methylphenol | C[C@]1(CC[C@H](Br)[C@]1(C)C)c2ccc(C)cc2O |
| 372 | RL348 | (3S,3aR,8bS)-3-bromo-3a,6,8b-trimethyl-2,3,3a,8b-tetrahydro-1H-benzo[b]cyclopenta[d]furan | Cc1cc2O[C@@]3(C)[C@@H](Br)CC[C@@]3(C)c2cc1 |
| 373 | RL349 | [(3S,3aS,8bS)-3,7-dibromo-6,8b-dimethyl-1,2,3,8b-tetrahydro-3aH-benzo[b]cyclopenta[d]furan-3a-yl]methanol | Brc1cc2c(cc1C)O[C@@]3(CO)[C@@H](Br)CC[C@@]23C |
| 374 | RL350 | [(3S,3aS,8bS)-3-bromo-6,8b-dimethyl-1,2,3,8b-tetrahydro-3aH-benzo[b]cyclopenta[d]furan-3a-yl]methanol | Cc1cc2O[C@@]3(CO)[C@@H](Br)CC[C@@]3(C)c2cc1 |
| 375 | RL351 | 4-bromo-2-[(1S,2R,5R)-1,2-dimethylbicyclo[3.1.0]hex-2-yl]-5-methylphenol | C[C@]2(CC[C@@H]1C[C@@]12C)c3cc(Br)c(C)cc3O |
| 376 | RL352 | 2-[(1S,2R,5R)-1,2-dimethylbicyclo[3.1.0]hex-2-yl]-5-methylphenol | C[C@]2(CC[C@@H]1C[C@@]12C)c3ccc(C)cc3O |
| 377 | RL353 | 2,4-dibromo-6-[(1S,2R,5R)-1,2-dimethylbicyclo[3.1.0]hex-2-yl]-3-methylphenol | C[C@]2(CC[C@@H]1C[C@@]12C)c3cc(Br)c(C)c(Br)c3O |
| 378 | RL354 | 4-bromo-2-{3-bromo-5-[(1R,2S,5S)-1,2-dimethylbicyclo[3.1.0]hexan-2-yl]-6-hydroxy-2-methylphenyl}-6-[(1S,2R,5R)-1,2-dimethylbicyclo[3.1.0]hexan-2-yl]-3-methylphenol | C[C@]2(CC[C@@H]1C[C@@]12C)c6cc(Br)c(C)c(c3c(C)c(Br)cc(c3O)[C@@]5(C)CC[C@H]4C[C@]45C)c6O |
| 379 | RG008 | 14-[(2R)-5-hydroperoxy-6-methylhept-6-en-2-yl]-8- hydroxy-2,15- dimethyltetracyclo[8.7.0.0^{2,7}.0^{11,15}]heptadec-6- en-5-one | C=C(C)C(OO)CC[C@@H](C)C2CCC3C4CC(O)C1=CC(=O)CCC1(C)C4CCC23C |
| 380 | RG009 | 14-[(2R,4E)-6-hydroperoxy-6-methylhept-4-en-2-yl]-8- hydroxy-2,15- dimethyltetracyclo[8.7.0.0^{2,7}.0^{11,15}]heptadec-6- en-5-one | CC(C)(OO)/C=C/C[C@@H](C)C2CCC3C4CC(O)C1=CC(=O)CCC1(C)C4CCC23C |
| 381 | RG010 | 14-[(2R)-5-hydroperoxy-6-methylhept-6-en-2-yl]- 2,15- dimethyltetracyclo[8.7.0.0^{2,7}.0^{11,15}]heptadec-6- ene-5,8-dione | C=C(C)C(OO)CC[C@@H](C)C2CCC1C3CC(=O)C4=CC(=O)CCC4(C)C3CCC12C |
| 382 | RG011 | 14-[(2R,4E)-6-hydroperoxy-6-methylhept-4-en-2-yl]- 2,15- dimethyltetracyclo[8.7.0.0^{2,7}.0^{11,15}]heptadec-6- ene-5,8-dione | CC(C)(OO)/C=C/C[C@@H](C)C2CCC1C3CC(=O)C4=CC(=O)CCC4(C)C3CCC12C |
| 383 | RG012 | 8-hydroxy-14-[(2R)-5-hydroxy-6-methylhept-6-en-2- yl]-2,15- dimethyltetracyclo[8.7.0.0^{2,7}.0^{11,15}]heptadec-6- en-5-one | C=C(C)C(O)CC[C@@H](C)C2CCC3C4CC(O)C1=CC(=O)CCC1(C)C4CCC23C |
| 384 | RG013 | 8-hydroxy-14-[(2R,4E)-6-hydroxy-6-methylhept-4-en-2- yl]-2,15- dimethyltetracyclo[8.7.0.0^{2,7}.0^{11,15}]heptadec-6- en-5-one | CC(C)(O)/C=C/C[C@@H](C)C2CCC3C4CC(O)C1=CC(=O)CCC1(C)C4CCC23C |
| 385 | RG014 | 2,15-dimethyl-14-[(2R)-6-methyl-5-oxohept-6-en-2- yl]tetracyclo[8.7.0.0^{2,7}.0^{11,15}]heptadec-6- ene-5,8-dione | C=C(C)C(=O)CC[C@@H](C)C2CCC1C3CC(=O)C4=CC(=O)CCC4(C)C3CCC12C |
| 386 | RL355 | (2R,3S,5S)-5-[(3Z)-1-chlorohex-3-en-5-yn-1-yl]-2-[(1Z)-3-chloropent-1-en-1-yl]oxolan-3-yl acetate | ClC(C/C=C\C#C)[C@@H]1C[C@H](OC(C)=O)[C@@H](\C=C/C(Cl)CC)O1 |
| 387 | RL356 | (2R,3S,5S)-5-[(3Z)-1-chlorohex-3-en-5-yn-1-yl]-2-[(1Z)-3-hydroxypent-1-en-1-yl]oxolan-3-ol | O[C@H]1C[C@H](O[C@@H]1/C=C\C(O)CC)C(Cl)C/C=C\C#C |
| 388 | RL357 | (2R,3S,5S)-5-[(3Z)-1-chlorohex-3-en-5-yn-1-yl]-2-[(1Z,3E)-penta-1,3-dien-1-yl]oxolan-3-yl acetate | ClC(C/C=C\C#C)[C@@H]1C[C@H](OC(C)=O)[C@@H](/C=C\C=C\C)O1 |
| 389 | RL358 | (2R,3R,5S)-3-bromo-2-[(1Z)-3-bromopent-1-en-1-yl]-5-[(3Z)-1-chlorohex-3-en-5-yn-1-yl]oxolane | C#C\C=C/CC(Cl)[C@@H]1C[C@@H](Br)[C@@H](\C=C/C(Br)CC)O1 |
| 390 | RL359 | (2R,3R,5S)-3-bromo-5-[(3Z)-1-chlorohex-3-en-5-yn-1-yl]-2-[(1Z)-3-methoxypent-1-en-1-yl]oxolane | C#C\C=C/CC(Cl)[C@@H]1C[C@@H](Br)[C@@H](\C=C/C(OC)CC)O1 |
| 391 | RL360 | (3Z,9Z,12E)-6-chloropentadeca-3,9,12-trien-1-yn-7-yl acetate | O=C(C)OC(C\C=C/C\C=C\CC)C(Cl)C/C=C\C#C |
| 392 | RL370 | (1R)-2-bromo-4-[(2E,4R)-4-(2,2-dimethyl-6-methylidenecyclohex-3-en-1-yl)-4-hydroxybut-2-en-2-yl]-1-methylcyclohexan-1-ol | C[C@@]1(O)CCC(CC1Br)C(\C)=C\[C@@H](O)C2C(=C)CC=CC2(C)C |
| 393 | RL361 | (2R)-2-[(1R,5S,7R,8R,10S,11S,13R,14R)-14- (acetyloxy)-11-[(acetyloxy)methyl]-8-hydroxy-1,5- dimethyltetracyclo[8.5.0.0^{2,7}.0^{11,13}]pentadec-2- en-5-yl]-2-bromoethyl acetate | CC(=O)OC[C@H](Br)[C@@]2(C)CC=C3[C@]4(C)C[C@@H](OC(C)=O)[C@@H]1C[C@]1(COC(C)=O)[C@H]4C[C@@H](O)[C@@H]3C2 |
| 394 | RL362 | (1R,5S,7R,8R,10S,11S,14S,15R)-5-[(1R)-1-bromo-2- hydroxyethyl]-1,5- dimethyltetracyclo[8.6.0.0^{2,7}.0^{11,14}]hexadec-2- ene-8,11,15-triol | Br[C@@H](CO)[C@@]2(C)CC=C3[C@]4(C)C[C@@H](O)[C@@H]1CC[C@]1(O)[C@H]4C[C@@H](O)[C@@H]3C2 |
| 395 | RL363 | (1R,5S,7R,8R,10S,11S,13R)-5-[(1R)-1-bromo-2- hydroxyethyl]-8-hydroxy-11-(hydroxymethyl)-1,5- dimethyltetracyclo[8.5.0.0^{2,7}.0^{11,13}]pentadec-2- en-14-one | Br[C@@H](CO)[C@@]2(C)CC=C3[C@]4(C)CC(=O)[C@@H]1C[C@]1(CO)[C@H]4C[C@@H](O)[C@@H]3C2 |
| 396 | RL364 | (1R,5S,7R,8R,10R,11S,13R)-5-[(1R)-1-bromo-2- hydroxyethyl]-1,5,11- trimethyltetracyclo[8.5.0.0^{2,7}.0^{11,13}]pentadec-2- en-8-ol | Br[C@@H](CO)[C@@]2(C)CC=C3[C@]4(C)CC[C@@H]1C[C@]1(C)[C@H]4C[C@@H](O)[C@@H]3C2 |
| 397 | RL365 | (1R,5S,7R,8R,10R,11R,13R,14R)-5-[(1R)-1-bromo-2- hydroxyethyl]-1,5,11- trimethyltetracyclo[8.5.0.0^{2,7}.0^{11,13}]pentadec-2- ene-8,14-diol | Br[C@@H](CO)[C@@]2(C)CC=C3[C@]4(C)C[C@@H](O)[C@@H]1C[C@]1(C)[C@H]4C[C@@H](O)[C@@H]3C2 |
| 398 | RL366 | (1R,5S,7R,8R,10R,13R,14R)-14-(acetyloxy)-5-[(1R)-1- bromo-2-hydroxyethyl]-8-hydroxy-1,5-dimethyl-11- methylidenetricyclo[8.5.0.0^{2,7}]pentadec-2-en-13- yl hexadecanoate | Br[C@@H](CO)[C@@]1(C)CC=C2[C@]3(C)C[C@@H](OC(C)=O)[C@@H](CC(=C)[C@H]3C[C@@H](O)[C@@H]2C1)OC(=O)CCCCCCCCCCCCCCC |
| 399 | RL367 | (1R,5S,7R,8R,10R,11R,13R,14R)-5-[(1R)-1-bromo-2- hydroxyethyl]-8-hydroxy-1,5,11- trimethyltetracyclo[8.5.0.0^{2,7}.0^{11,13}]pentadec-2- en-14-yl acetate | Br[C@@H](CO)[C@@]2(C)CC=C3[C@]4(C)C[C@@H](OC(C)=O)[C@@H]1C[C@]1(C)[C@H]4C[C@@H](O)[C@@H]3C2 |
| 400 | RL368 | (2R)-2-[(1R,5S,7R,8R,10S,11S,13R,14R)-8,14- bis(acetyloxy)-11-[(acetyloxy)methyl]-1,5- dimethyltetracyclo[8.5.0.0^{2,7}.0^{11,13}]pentadec-2- en-5-yl]-2-bromoethyl acetate | CC(=O)OC[C@H](Br)[C@@]2(C)CC=C3[C@]4(C)C[C@@H](OC(C)=O)[C@@H]1C[C@]1(COC(C)=O)[C@H]4C[C@@H](OC(C)=O)[C@@H]3C2 |
| 401 | RL369 | (1R,5S,7R,8R,10S,11S,13R,14R)-5-[(1R)-1-bromo-2- hydroxyethyl]-11-(hydroxymethyl)-1,5- dimethyltetracyclo[8.5.0.0^{2,7}.0^{11,13}]pentadec-2- ene-8,14-diol | Br[C@@H](CO)[C@@]2(C)CC=C3[C@]4(C)C[C@@H](O)[C@@H]1C[C@]1(CO)[C@H]4C[C@@H](O)[C@@H]3C2 |
| 402 | RL371 | (2R)-2-[(1R,5S,7R,8R,10S,11S,14S,15R)-8,15- bis(acetyloxy)-11-hydroxy-1,5- dimethyltetracyclo[8.6.0.0^{2,7}.0^{11,14}]hexadec-2- en-5-yl]-2-bromoethyl acetate | CC(=O)OC[C@H](Br)[C@@]2(C)CC=C3[C@]4(C)C[C@@H](OC(C)=O)[C@@H]1CC[C@]1(O)[C@H]4C[C@@H](OC(C)=O)[C@@H]3C2 |
| 403 | RL372 | 2-[(1R,5S,7R,8R,10S,11S,13R,14R)-8,14- bis(acetyloxy)-11-[(acetyloxy)methyl]-1,5- dimethyltetracyclo[8.5.0.0^{2,7}.0^{11,13}]pentadec-2- en-5-yl]ethyl acetate | CC(=O)OC[C@@H2][C@@]2(C)CC=C3[C@]4(C)C[C@@H](OC(C)=O)[C@@H]1C[C@]1(COC(C)=O)[C@H]4C[C@@H](OC(C)=O)[C@@H]3C2 |
| 404 | RL373 | [(1R,5S,7R,8R,10S,11S,13R,14R)-8,14-bis(acetyloxy)- 1,5-dimethyl-5-[(2S)-oxiran-2- yl]tetracyclo[8.5.0.0^{2,7}.0^{11,13}]pentadec-2- en-11-yl]methyl acetate | CC(=O)O[C@@H]2C[C@@]1(C)C4=CC[C@@](C)(C[C@H]4[C@@H](C[C@@H]1[C@@]3(COC(C)=O)C[C@@H]23)OC(C)=O)[C@H]5CO5 |
| 405 | RL374 | [(1R,5S,7R,8R,10S,11S,13R,14R)-8,14-bis(acetyloxy)-5- [(1R)-1-bromo-2-hydroxyethyl]-1,5- dimethyltetracyclo[8.5.0.0^{2,7}.0^{11,13}]pentadec-2- en-11-yl]methyl acetate | Br[C@@H](CO)[C@@]2(C)CC=C3[C@]4(C)C[C@@H](OC(C)=O)[C@@H]1C[C@]1(COC(C)=O)[C@H]4C[C@@H](OC(C)=O)[C@@H]3C2 |
| 406 | RL375 | [(1R,5S,7R,8R,10S,11S,13R,14R)-14-(acetyloxy)-8- hydroxy-1,5-dimethyl-5-[(2S)-oxiran-2- yl]tetracyclo[8.5.0.0^{2,7}.0^{11,13}]pentadec-2- en-11-yl]methyl acetate | CC(=O)O[C@@H]2C[C@@]1(C)C4=CC[C@@](C)(C[C@H]4[C@H](O)C[C@@H]1[C@@]3(COC(C)=O)C[C@@H]23)[C@H]5CO5 |
| 407 | RL376 | (1R,5S,7R,8R,10S,11S,13R,14R)-8-(acetyloxy)-11- (hydroxymethyl)-1,5-dimethyl-5-[(2S)-oxiran-2- yl]tetracyclo[8.5.0.0^{2,7}.0^{11,13}]pentadec-2- en-14-yl acetate | CC(=O)O[C@@H]2C[C@@]1(C)C4=CC[C@@](C)(C[C@H]4[C@@H](C[C@@H]1[C@@]3(CO)C[C@@H]23)OC(C)=O)[C@H]5CO5 |
| 408 | RL377 | (1R,5S,7R,8R,10S,11S,13R,14R)-8-(acetyloxy)-5- [(1R)-1-bromo-2-hydroxyethyl]-11-(hydroxymethyl)- 1,5- dimethyltetracyclo[8.5.0.0^{2,7}.0^{11,13}]pentadec-2- en-14-yl acetate | Br[C@@H](CO)[C@@]2(C)CC=C3[C@]4(C)C[C@@H](OC(C)=O)[C@@H]1C[C@]1(CO)[C@H]4C[C@@H](OC(C)=O)[C@@H]3C2 |
| 409 | RL378 | [(1R,5S,7R,8R,10S,11S,13R,14R)-14-(acetyloxy)-5- [(1R)-1-bromo-2-hydroxyethyl]-8-hydroxy-1,5- dimethyltetracyclo[8.5.0.0^{2,7}.0^{11,13}]pentadec-2- en-11-yl]methyl acetate | Br[C@@H](CO)[C@@]2(C)CC=C3[C@]4(C)C[C@@H](OC(C)=O)[C@@H]1C[C@]1(COC(C)=O)[C@H]4C[C@@H](O)[C@@H]3C2 |
| 410 | RL379 | (1R,5S,7R,8R,10S,11S,13R,14R)-8-hydroxy-11- (hydroxymethyl)-1,5-dimethyl-5-[(2S)-oxiran-2- yl]tetracyclo[8.5.0.0^{2,7}.0^{11,13}]pentadec-2- en-14-yl acetate | CC(=O)O[C@@H]2C[C@@]1(C)C4=CC[C@@](C)(C[C@H]4[C@H](O)C[C@@H]1[C@@]3(CO)C[C@@H]23)[C@H]5CO5 |
| 411 | RL380 | (1R,5S,7R,8R,10S,11S,13R,14R)-5-[(1R)-1-bromo-2- hydroxyethyl]-8-hydroxy-11-(hydroxymethyl)-1,5- dimethyltetracyclo[8.5.0.0^{2,7}.0^{11,13}]pentadec-2- en-14-yl acetate | Br[C@@H](CO)[C@@]2(C)CC=C3[C@]4(C)C[C@@H](OC(C)=O)[C@@H]1C[C@]1(CO)[C@H]4C[C@@H](O)[C@@H]3C2 |
| 412 | RL381 | [(1R,5S,7R,8R,10S,11S,13R,14R)-8,14-bis(acetyloxy)-5- [(1R)-1,2-dihydroxyethyl]-1,5- dimethyltetracyclo[8.5.0.0^{2,7}.0^{11,13}]pentadec-2- en-11-yl]methyl acetate | OC[C@H](O)[C@@]2(C)CC=C3[C@]4(C)C[C@@H](OC(C)=O)[C@@H]1C[C@]1(COC(C)=O)[C@H]4C[C@@H](OC(C)=O)[C@@H]3C2 |
| 413 | RL382 | (1R,5S,7R,8R,10S,11S,13R,14R)-11-(hydroxymethyl)- 1,5-dimethyl-5-[(2S)-oxiran-2- yl]tetracyclo[8.5.0.0^{2,7}.0^{11,13}]pentadec-2- ene-8,14-diol | C[C@]35C[C@@H](O)[C@@H]1C[C@]1(CO)[C@H]5C[C@@H](O)[C@@H]2C[C@](C)(CC=C23)[C@H]4CO4 |
| 414 | RL383 | (2R,3S,6R)-2-bromo-8-chloro-1,1,9-trimethyl-5-methylidenespiro[5.5]undec-8-en-3-ol | ClC=1C[C@]2(CCC=1C)C(=C)C[C@H](O)[C@H](Br)[C@]2(C)C |
| 415 | RL384 | (3E)-1-[(2R,7S,8R)-7-bromo-8-ethyl-3,6,7,8-tetrahydro-2H-oxocin-2-yl]hex-3-en-5-yn-1-yl acetate | CC(=O)OC(C\C=C\C#C)[C@H]1CC=CC[C@H](Br)[C@@H](CC)O1 |
| 416 | RL385 | (1S,2S,3aS,5aS,6R)-1,2,3a,6-tetramethyldecahydrocyclopenta[c]pentalen-1-ol | C[C@@H]3CCC21[C@H]3CC[C@@]2(C)C[C@H](C)[C@]1(C)O |
| 417 | RL386 | (2R,3S,6S,9Z)-2-bromo-9-(bromomethylidene)-1,1-dimethyl-5-methylidenespiro[5.5]undec-7-en-3-ol | Br\C=C1\CC[C@]2(C=C1)C(=C)C[C@H](O)[C@H](Br)[C@@]2(C)C |
| 418 | RL387 | (2R,3S,6S,9E)-2-bromo-9-(bromomethylidene)-1,1-dimethyl-5-methylidenespiro[5.5]undec-7-en-3-ol | Br\C=C1/CC[C@]2(C=C1)C(=C)C[C@H](O)[C@H](Br)[C@@]2(C)C |
| 419 | RL388 | (2R,3S,6S)-2-bromo-1,1-dimethyl-5,9-dimethylidenespiro[5.5]undec-7-en-3-ol | C=C1CC[C@]2(C=C1)C(=C)C[C@H](O)[C@H](Br)[C@@]2(C)C |
| 420 | RL389 | (2R,3S,6R,8S,9S)-2,8-dibromo-9-chloro-1,1,9-trimethyl-5-methylidenespiro[5.5]undecan-3-ol | C=C2C[C@H](O)[C@H](Br)[C@@](C)(C)[C@]12CC[C@](C)(Cl)[C@@H](Br)C1 |
| 421 | RL390 | (3R,4S,9S)-3-bromo-9-chloro-2,2,8-trimethyltricyclo[6.2.2.01,6]dodec-6-en-4-ol | Cl[C@H]3CC21CCC3(C)C=C2C[C@H](O)[C@H](Br)[C@]1(C)C |
| 422 | RL391 | 2,3,5,8-tetramethyltricyclo[5.3.1.01,5]undecan-2-ol | CC3CCC21CC3CC2(C)CC(C)C1(C)O |
| 423 | RL392 | (1R,2S,3aS,5aS,6R)-1,2,3a,6-tetramethyldecahydrocyclopenta[c]pentalen-1-ol | C[C@@H]3CCC21[C@H]3CC[C@@]2(C)C[C@H](C)[C@@]1(C)O |
| 424 | RL393 | (3R,3aS,5aS)-3,5a,7,8-tetramethyl-1,2,3,3a,4,5,5a,6-octahydrocyclopenta[c]pentalene | CC1=C(C)C[C@]3(C)CC[C@H]2[C@H](C)CCC123 |
| 425 | RL394 | (1R,3aS,5aS,8aS)-1,3a,6-trimethyl-8-oxo-1,3a,4,5,5a,6,7,8-octahydrocyclopenta[c]pentalene-2-carboxylic acid | O=C(O)C1=C[C@]3(C)CC[C@H]2[C@H](C)CC(=O)[C@]23[C@H]1C |
| 426 | RL395 | 1,1,5,9-tetramethylspiro[5.5]undecane | CC1CCC2(CC1)C(C)CCC[C@@]2(C)C |
| 427 | RL396 | (2R,3S,6R,8S,9S)-2,9-dibromo-8-chloro-1,1,9-trimethyl-5-methylidenespiro[5.5]undecan-3-ol | Br[C@@]1(C)CC[C@@]2(C[C@@H]1Cl)C(=C)C[C@H](O)[C@H](Br)[C@]2(C)C |
| 428 | RL397 | (3R,9S)-3-bromo-9-chloro-2,2,8-trimethyltricyclo[6.2.2.01,6]dodec-6-en-4-one | O=C3CC2=CC1(C)CCC2(C[C@@H]1Cl)[C@@](C)(C)[C@H]3Br |
| 429 | RL398 | (3R,4S,9S)-3-bromo-9-chloro-2,2,8-trimethyltricyclo[6.2.2.01,6]dodec-5-en-4-ol | Cl[C@H]3CC21CCC3(C)CC2=C[C@H](O)[C@H](Br)[C@]1(C)C |
| 430 | RL399 | (3R,9S)-3-bromo-9-chloro-2,2,8-trimethyltricyclo[6.2.2.01,6]dodec-5-en-4-one | O=C3C=C2CC1(C)CCC2(C[C@@H]1Cl)[C@](C)(C)[C@H]3Br |
| 431 | RL400 | 4-bromo-2-[(1S,2R,5R)-1,2-dimethylbicyclo[3.1.0]hex-2-yl]-5-methylphenol | C[C@]2(CC[C@@H]1C[C@@]12C)c3cc(Br)c(C)cc3O |
| 432 | RL401 | 2-[(1S,2R,5R)-1,2-dimethylbicyclo[3.1.0]hex-2-yl]-5-methylphenol | C[C@]2(CC[C@@H]1C[C@@]12C)c3ccc(C)cc3O |
| 433 | RL402 | 4-bromo-2-[(1R,3S)-1,3-dimethyl-2-methylidenecyclopentyl]-5-methylphenol | C[C@]1(CC[C@H](C)C1=C)c2cc(Br)c(C)cc2O |
| 434 | RL403 | (1R,2R,6R,7S,8S)-8-chloro-4,4,7-trimethyl-2-(propan-2-yl)-10-oxatricyclo[4.3.1.01,6]decan-7-ol | CC(C)[C@H]2C[C@@](C)(C)C[C@]31O[C@]23C[C@H](Cl)[C@@]1(C)O |
| 435 | RL404 | (1S,2R,6S,7R,8R)-8-chloro-4,4,7-trimethyl-2-(propan-2-yl)-10-oxatricyclo[4.3.1.01,6]decan-7-ol | CC(C)[C@H]2C[C@](C)(C)C[C@@]31O[C@@]23C[C@@H](Cl)[C@]1(C)O |
| 436 | RL405 | (1R,2R,6R,7S,8S)-8-bromo-4,4,7-trimethyl-2-(propan-2-yl)-10-oxatricyclo[4.3.1.01,6]decan-7-ol | CC(C)[C@H]2C[C@@](C)(C)C[C@]31O[C@]23C[C@H](Br)[C@@]1(C)O |
| 437 | RL406 | (3R,4S,7R)-3,5,5-trimethyl-7-(propan-2-yl)-2,3,4,5,6,7-hexahydro-1H-inden-4-ol | CC(C)[C@H]2C[C@](C)(C)[C@H](O)C=1[C@H](C)CCC=12 |
| 438 | RL407 | (3R,4R,7R)-3,5,5-trimethyl-7-(propan-2-yl)-2,3,4,5,6,7-hexahydro-1H-inden-4-ol | CC(C)[C@H]2C[C@@](C)(C)[C@@H](O)C=1[C@H](C)CCC=12 |
| 439 | RL408 | (6E)-6-[(3S,4S)-3-bromo-4-chloro-4-methylcyclohexylidene]-2-methylhept-2-en-4-ol | C[C@]1(Cl)CCC(\C[C@@H]1Br)=C(\C)CC(O)/C=C(\C)C |
| 440 | RL409 | (6E)-6-[(3S,4S)-4-bromo-3-chloro-4-methylcyclohexylidene]-2-methylhept-2-en-4-ol | Br[C@@]1(C)CCC(\C[C@@H]1Cl)=C(\C)CC(O)/C=C(\C)C |
| 441 | RL410 | (1S,2S,4S,5S,6S,8R)-4-hydroxy-2,5,6-trimethyl-11-methylidenetricyclo[6.2.1.01,6]undecan-3-one | O=C1[C@@H](C)[C@@]32CC[C@H](C[C@@]2(C)[C@H](C)[C@@H]1O)C3=C |
| 442 | RL411 | (3R,4R,4aR,9R)-3,9-dihydroxy-1,4,4a,7-tetramethyl-3,4,4a,5,8,9-hexahydro-2H-benzo[7]annulen-2-one | O=C2C(C)=C1[C@H](O)CC(C)=CC[C@]1(C)[C@@H](C)[C@H]2O |
| 443 | RL412 | (3R,4R,4aR)-3-hydroxy-1,4,4a,7-tetramethyl-3,4,4a,5,8,9-hexahydro-2H-benzo[7]annulen-2-one | O=C2C(C)=C1[C@H2]CC(C)=CC[C@]1(C)[C@@H](C)[C@H]2O |
| 444 | RL413 | 5-[(5S)-5-bromo-2,6,6-trimethylcyclohex-2-en-1-yl]-3-methylpent-1-en-3-ol | C[C@]1(C)C(CCC(C)(O)C=C)C(C)=CC[C@@H]1Br |
| 445 | RL414 | 5-[(3S)-3-bromo-2,2-dimethyl-6-methylidenecyclohexyl]-3-methylpent-1-en-3-ol | C[C@]1(C)C(CCC(C)(O)C=C)C(=C)CC[C@@H]1Br |
| 446 | RP001 | (6S,7aR)-6-hydroxy-4,4,7a-trimethyl-5,6,7,7a-tetrahydro-1-benzofuran-2(4H)-one | O=C1C=C2[C@@](C)(C)C[C@H](O)C[C@@]2(C)O1 |
| 447 | RB001 | (6S,7aR)-6-hydroxy-4,4,7a-trimethyl-5,6,7,7a-tetrahydro-1-benzofuran-2(4H)-one | O=C1C=C2[C@@](C)(C)C[C@H](O)C[C@@]2(C)O1 |
| 448 | RP002 | (6S,7aR)-6-hydroxy-4,4,7a-trimethyl-5,6,7,7a-tetrahydro-1-benzofuran-2(4H)-one | O=C1C=C2[C@@](C)(C)C[C@H](O)C[C@@]2(C)O1 |
| 449 | RG015 | (6S,7aR)-6-hydroxy-4,4,7a-trimethyl-5,6,7,7a-tetrahydro-1-benzofuran-2(4H)-one | O=C1C=C2[C@@](C)(C)C[C@H](O)C[C@@]2(C)O1 |
| 450 | RH001 | (6S,7aR)-6-hydroxy-4,4,7a-trimethyl-5,6,7,7a-tetrahydro-1-benzofuran-2(4H)-one | O=C1C=C2[C@@](C)(C)C[C@H](O)C[C@@]2(C)O1 |
| 451 | RC001 | (6S,7aR)-6-hydroxy-4,4,7a-trimethyl-5,6,7,7a-tetrahydro-1-benzofuran-2(4H)-one | O=C1C=C2[C@@](C)(C)C[C@H](O)C[C@@]2(C)O1 |
| 452 | RH002 | (6S,7aR)-6-hydroxy-4,4,7a-trimethyl-5,6,7,7a-tetrahydro-1-benzofuran-2(4H)-one | O=C1C=C2[C@@](C)(C)C[C@H](O)C[C@@]2(C)O1 |
| 453 | BT001 | (6S,7aR)-6-hydroxy-4,4,7a-trimethyl-5,6,7,7a-tetrahydro-1-benzofuran-2(4H)-one | O=C1C=C2[C@@](C)(C)C[C@H](O)C[C@@]2(C)O1 |
| 454 | BC001 | (6S,7aR)-6-hydroxy-4,4,7a-trimethyl-5,6,7,7a-tetrahydro-1-benzofuran-2(4H)-one | O=C1C=C2[C@@](C)(C)C[C@H](O)C[C@@]2(C)O1 |
| 455 | BD001 | (6S,7aR)-6-hydroxy-4,4,7a-trimethyl-5,6,7,7a-tetrahydro-1-benzofuran-2(4H)-one | O=C1C=C2[C@@](C)(C)C[C@H](O)C[C@@]2(C)O1 |
| 456 | BC002 | (6S,7aR)-6-hydroxy-4,4,7a-trimethyl-5,6,7,7a-tetrahydro-1-benzofuran-2(4H)-one | O=C1C=C2[C@@](C)(C)C[C@H](O)C[C@@]2(C)O1 |
| 457 | BS001 | (6S,7aR)-6-hydroxy-4,4,7a-trimethyl-5,6,7,7a-tetrahydro-1-benzofuran-2(4H)-one | O=C1C=C2[C@@](C)(C)C[C@H](O)C[C@@]2(C)O1 |
| 458 | GE001 | (6S,7aR)-6-hydroxy-4,4,7a-trimethyl-5,6,7,7a-tetrahydro-1-benzofuran-2(4H)-one | O=C1C=C2[C@@](C)(C)C[C@H](O)C[C@@]2(C)O1 |
| 459 | RL415 | 2-[(1R,2S)-1,2-dimethyl-3-methylidenecyclopentyl]-5-methylphenol | C[C@]1(CCC(=C)[C@@H]1C)c2ccc(C)cc2O |
| 460 | RL416 | 4-bromo-2-[(1R,2S)-1,2-dimethyl-3-methylidenecyclopentyl]-5-methylphenol | C[C@]1(CCC(=C)[C@@H]1C)c2cc(Br)c(C)cc2O |
| 461 | RL417 | 1-[(1R,2S)-1,2-dimethyl-3-methylidenecyclopentyl]-4-methylbenzene | C[C@]1(CCC(=C)[C@@H]1C)c2ccc(C)cc2 |
| 462 | RL418 | (1R,3R,5R,6R)-5-bromo-3-(1-bromopropyl)-8-(pent-2-en-4-yn-1-yl)-2,7-dioxabicyclo[4.2.1]nonane | BrC(CC)[C@@H]2O[C@@H]1C[C@@H](OC1C\C=C\C#C)[C@H](Br)C2 |
| 463 | RL419 | (1R,3R,4S,6R,7R)-4-bromo-3-ethyl-9-(pent-2-en-4-yn-1-yl)-2,8-dioxabicyclo[5.2.1]decan-6-ol | C#C\C=C/CC2O[C@@H]1C[C@H]2O[C@H](CC)[C@@H](Br)C[C@H]1O |
| 464 | RL420 | (1R,3R,4S,6R,7R)-4-bromo-3-ethyl-9-(pent-2-en-4-yn-1-yl)-2,8-dioxabicyclo[5.2.1]decan-6-yl acetate | CC(=O)O[C@@H]2C[C@H](Br)[C@@H](CC)O[C@@H]1C[C@H]2OC1C/C=C\C#C |
| 465 | RL421 | (1R,3R,4Z,7R)-3-ethyl-9-(pent-2-en-4-yn-1-yl)-2,8-dioxabicyclo[5.2.1]dec-4-en-6-ol | CC[C@@H]2C=CC(O)[C@@H]1OC(C/C=C\C#C)[C@@H](C1)O2 |
| 466 | RL422 | (3R,4R,7S,7aS)-7-bromo-3-(1-hydroxy-2-methylprop-2-en-1-yl)-4,7a-dimethyl-octahydro-1H-inden-4-ol | C=C(C)C(O)[C@@H]1CC[C@@]2(C)C1[C@](C)(O)CC[C@@H]2Br |
| 467 | RL423 | (3R,4R,7S,7aS)-7-bromo-3-(1-hydroxy-2-methylprop-2-en-1-yl)-4,7a-dimethyl-octahydro-1H-inden-4-ol | C=C(C)C(O)[C@@H]1CC[C@@]2(C)C1[C@](C)(O)CC[C@@H]2Br |
| 468 | BU001 | (2R,3aS,6S,7aS)-2,3a-dihydroxy-4,4,7a-trimethyl-3-oxooctahydro-1-benzofuran-6-yl acetate | CC(=O)O[C@H]1C[C@](C)(C)[C@@]2(O)C(=O)[C@H](O)O[C@@]2(C)[C@H2]1 |
| 469 | BU002 | (3aR,6S,7aS)-3a,6-dihydroxy-4,4,7a-trimethylhexahydro-1-benzofuran-2(3H)-one | O=C1C[C@@]2(O)[C@](C)(C)C[C@H](O)C[C@]2(C)O1 |
| 470 | BU003 | (3aS,6S,7aR)-3a,6-dihydroxy-4,4,7a-trimethylhexahydro-1-benzofuran-2(3H)-one | O=C1C[C@]2(O)[C@@](C)(C)C[C@H](O)C[C@@]2(C)O1 |
| 471 | BU004 | (6S,7aR)-6-hydroxy-4,4,7a-trimethyl-5,6,7,7a-tetrahydro-1-benzofuran-2(4H)-one | O=C1C=C2[C@@](C)(C)C[C@H](O)C[C@@]2(C)O1 |
| 472 | BU005 | (6S,7aS)-6-hydroxy-4,4,7a-trimethyl-5,6,7,7a-tetrahydro-1-benzofuran-2(4H)-one | O=C1C=C2[C@@](C)(C)C[C@H](O)C[C@]2(C)O1 |
| 473 | BD002 | 4R,5aR,6R,9aS,10aS)-4,6,9a-trihydroxy-5a,10a-dimethyl-9-methylidene-3-(propan-2-yl)-1H,2H,4H,5H,5aH,6H,7H,8H,9H,9aH,10H,10aH-cyclohexa[f]azulen-2-one | CC(C)C1=C2[C@H](O)C[C@]3(C)[C@H](O)CCC(=C)[C@@]3(O)C[C@@]2(C)CC1=O |
| 474 | BD003 | [(4R,4aS,6R,9aS,10aS)-4,6,10a-trihydroxy-9a-methyl-1-methylidene-8-oxo-7-(propan-2-yl)-1H,2H,3H,4H,4aH,5H,6H,8H,9H,9aH,10H,10aH-cyclohexa[f]azulen-4a-yl]methyl acetate | CC(C)C1=C2[C@H](O)C[C@]3(COC(C)=O)[C@H](O)CCC(=C)[C@@]3(O)C[C@@]2(C)CC1=O |
| 475 | BD004 | (6S,7aR)-6-hydroxy-4,4,7a-trimethyl-5,6,7,7a-tetrahydro-1-benzofuran-2(4H)-one | O=C1C=C2[C@@](C)(C)C[C@H](O)C[C@@]2(C)O1 |
| 476 | RL424 | (1aR,4aR,7R,7bR)-1,1,7-trimethyl-4-methylidenedecahydro-4aH-cyclopropa[e]azulen-4a-ol | CC2(C)[C@@H]1CCC(=C)[C@@]3(O)CC[C@@H](C)C3[C@@H]12 |
| 477 | RL425 | (1aR,4S,7R,7bR)-1,1,4,7-tetramethyl-1a,2,3,4,6,7,7a,7b-octahydro-1H-cyclopropa[e]azulen-4-ol | C[C@@H]1CC=C2C1[C@H]3[C@@H](CC[C@]2(C)O)C3(C)C |
| 478 | RL426 | (1R,2S,5R,7S,9S,10S,12R,14S)-12-bromo-5-ethenyl-9- hydroxy-1,5,7,11,11-pentamethyl-6- oxatricyclo[8.4.0.0^{2,7}]tetradecan-14-yl acetate | CC(=O)O[C@H]2C[C@@H](Br)[C@@](C)(C)[C@H]1[C@@H](O)C[C@]3(C)O[C@@](C)(C=C)CC[C@H]3[C@@]12C |
| 479 | RL427 | (1R,2S,5R,7S,9S,10S,12R)-12-bromo-5-ethenyl-9- hydroxy-1,5,7,11,11-pentamethyl-6- oxatricyclo[8.4.0.0^{2,7}]tetradecan-14-one | C[C@]2(C)[C@H](Br)CC(=O)[C@@]1(C)[C@@H]3CC[C@](C)(C=C)O[C@@]3(C)C[C@H](O)[C@@H]12 |
| 480 | RL428 | (4R,6S,8S,9S)-4,8-dibromo-9-chloro-1,5,5,9-tetramethylspiro[5.5]undec-1-ene | CC2=CC[C@@H](Br)[C@](C)(C)[C@@]12CC[C@](C)(Cl)[C@@H](Br)C1 |
| 481 | RL429 | (2R,3S,4aS,6R,8Z,10aS)-3-bromo-6-(3-bromopropadienyl)-2-ethyl-2,3,4,4a,6,7,10,10a-octahydropyrano[3,2-b]oxocine | Br\C=C=C\[C@H]1CC=CC[C@@H]2O[C@H](CC)[C@@H](Br)C[C@@H]2O1 |
| 482 | RL430 | (2S,6R,9R)-2,5,9-trimethyl-1-methylidenespiro[5.5]undeca-4,7-diene-2,9-diol | C=C2[C@@]1(CC[C@@](C)(O)C=C1)C(C)=CC[C@]2(C)O |
| 483 | RL431 | (3R,6S,8S,9S)-8-bromo-3,7,7-trimethyl-11-methylidenespiro[5.5]undec-1-ene-3,9-diol | C[C@@]1(O)CC[C@]2(C=C1)C(=C)C[C@H](O)[C@@H](Br)[C@@]2(C)C |
| 484 | RL432 | (3Z,6S,8R)-8-bromo-3-(bromomethylidene)-7,7-dimethyl-11-methylidenespiro[5.5]undec-1-ene | C=C2CC[C@@H](Br)[C@](C)(C)[C@]12C=CC(CC1)=[C@H]Br |
| 485 | RL433 | (3E,6S,8R)-8-bromo-3-(bromomethylidene)-7,7-dimethyl-11-methylidenespiro[5.5]undec-1-ene | C=C2CC[C@@H](Br)[C@](C)(C)[C@]12C=CC(CC1)=[C@H]Br |
| 486 | RL434 | (2S,3R,6S,9Z)-2-bromo-9-(bromomethylidene)-1,1-dimethyl-5-methylidenespiro[5.5]undec-7-en-3-ol | Br[C@H]=C1CC[C@]2(C=C1)C(=C)C[C@@H](O)[C@@H](Br)[C@@]2(C)C |
| 487 | RL435 | (2S,3R,6S,9E)-2-bromo-9-(bromomethylidene)-1,1-dimethyl-5-methylidenespiro[5.5]undec-7-en-3-ol | Br[C@H]=C1CC[C@]2(C=C1)C(=C)C[C@@H](O)[C@@H](Br)[C@@]2(C)C |
| 488 | RL436 | (6S,8S,9R)-8-bromo-9-hydroxy-7,7-dimethyl-11-methylidenespiro[5.5]undec-1-en-3-one | O=C1CC[C@]2(C=C1)C(=C)C[C@@H](O)[C@@H](Br)[C@@]2(C)C |
| 489 | RL437 | (6R)-1,5,5-trimethylspiro[5.5]undeca-1,7-diene-3,9-dione | CC2=CC(=O)C[C@@](C)(C)[C@]12C=CC(=O)CC1 |
| 490 | RL438 | (2R,3R,6R)-2-bromo-1,1,9-trimethyl-5-methylidenespiro[5.5]undec-8-en-3-ol | CC=1CC[C@@]2(CC=1)C(=C)C[C@@H](O)[C@H](Br)[C@]2(C)C |
| 491 | RL439 | (2S,3R,6S,8S,9S)-2,9-dibromo-8-chloro-1,1,9-trimethyl-5-methylidenespiro[5.5]undecan-3-ol | Br[C@@]1(C)CC[C@]2(C[C@@H]1Cl)C(=C)C[C@@H](O)[C@@H](Br)[C@@]2(C)C |
| 492 | RL440 | (2R,6S,8S,9S)-2,9-dibromo-8-chloro-1,1,9-trimethyl-5-methylidenespiro[5.5]undecane | C=C2CC[C@@H](Br)[C@](C)(C)[C@@]12CC[C@@](Br)(C)[C@@H](Cl)C1 |
| 493 | RL441 | (2E)-2-tridecylheptadec-2-enal | CCCCCCCCCCCCCC\C=C(/CCCCCCCCCCCCC)C=O |
| 494 | RL442 | (R)-[(1R,4S)-4-bromo-1-hydroxy-3,3- dimethylcyclohexyl][(1S,4R,6S,9S)-9-bromo-6-methyl- 10-oxo-11-oxatricyclo[4.3.2.0^{1,5}]undecan-4- yl]methyl acetate | C[C@]1(C)C[C@](O)(CC[C@@H]1Br)[C@H](OC(C)=O)[C@@H]3CC[C@]24C(=O)O[C@](C)(CC[C@@H]2Br)C34 |
| 495 | RL443 | (1S,4aS,6S,8aS)-1-bromo-6-[(2E,4E)-6-hydroxy-6-methylhepta-2,4-dien-2-yl]-8a-methyl-4-methylidene-decahydronaphthalen-4a-ol | CC(C)(O)/C=C/C=C(\C)[C@H]1CC[C@]2(C)[C@@H](Br)CCC(=C)[C@@]2(O)C1 |
| 496 | RL444 | 4-bromo-5-[(2R)-1,2-dimethylbicyclo[3.1.0]hex-2-yl]-2-methylphenol | C[C@]2(CCC1CC12C)c3cc(O)c(C)cc3Br |
| 497 | RL445 | 2,3,5-tribromo-1-methyl-1H-indole | Brc1cc2c(cc1)n(C)c(Br)c2Br |
| 498 | RL446 | (1S,4R)-3',4-dibromo-4'-chloro-1,3,3,4'-tetramethyl-7-oxaspiro[bicyclo[4.1.0]heptane-2,1'-cyclohexane] | CC1(Cl)CCC3(CC1Br)C(C)(C)[C@H](Br)CC2O[C@]23C |
| 499 | RL447 | (2R,3S,6R)-2-bromo-8-chloro-1,1,9-trimethyl-5-methylidenespiro[5.5]undec-8-en-3-ol | ClC=1C[C@]2(CCC=1C)C(=C)C[C@H](O)[C@H](Br)[C@]2(C)C |
| 500 | RL448 | (2R,3S,6R,8S,9S)-2,9-dibromo-8-chloro-1,1,9-trimethyl-5-methylidenespiro[5.5]undecan-3-ol | Br[C@@]1(C)CC[C@@]2(C[C@@H]1Cl)C(=C)C[C@H](O)[C@H](Br)[C@]2(C)C |
| 501 | RL449 | (5Z,6S,9R)-5-(1-bromopropylidene)-9-[(1S,2Z)-1-chloropent-2-en-4-yn-1-yl]-4,8-dioxatricyclo[4.2.1.03,7]nonane | C#C\C=C/[C@H](Cl)[C@H]3C1CC2O\C(=C(/Br)CC)[C@@H]3C2O1 |
| 502 | RL450 |  | C#C\C=C/[C@@H]2[C@H]4C1OC3C(C1)O[C@@](Br)([C@@]2(Br)CC)[C@H]34 |
| 503 | RL453 | (2R,3S,6R)-2-bromo-8-chloro-1,1,9-trimethyl-5-methylidenespiro[5.5]undec-8-en-3-ol | ClC=1C[C@]2(CCC=1C)C(=C)C[C@H](O)[C@H](Br)[C@]2(C)C |
| 504 | RL451 | (1S,2R,3'R,3'aS,4S,5R,5'S,6'aR)-3'-bromo-5'-(3-bromopropa-1,2-dien-1-yl)-4-ethyl-tetrahydro-3'H-3-oxaspiro[bicyclo[3.1.0]hexane-2,2'-furo[3,2-b]furan] | Br[C@@H]3[C@H]4O[C@@H](C[C@H]4O[C@@]31O[C@@H](CC)[C@@H]2C[C@H]12)/C=C=[C@H]Br |
| 505 | RL452 | 1-[(1S,4S,4aS,6R,8aS)-4-(acetyloxy)-6-bromo-5,5,8a-trimethyl-2-methylidene-decahydronaphthalen-1-yl]-3-hydroxy-3-methylpent-4-en-2-yl acetate | C=CC(C)(O)C(OC(C)=O)C[C@H]2C(=C)C[C@H](OC(C)=O)[C@H]1[C@@]2(C)CC[C@@H](Br)[C@@]1(C)C |
| 506 | RL454 | (2R,4S,5R,6aR,8S,9aR)-4-bromo-8-(3-bromopropa-1,2-dien-1-yl)-2-(1-bromopropyl)-decahydrocyclopenta[b]oxocin-5-ol | BrC(CC)[C@H]1C[C@H](Br)[C@H](O)C[C@H]2C[C@@H](C[C@H]2O1)/C=C=[C@H]Br |
| 507 | RL455 | (4R,6S,8S,9S)-4,8-dibromo-9-chloro-1,5,5,9-tetramethylspiro[5.5]undec-1-ene | CC2=CC[C@@H](Br)[C@](C)(C)[C@@]12CC[C@](C)(Cl)[C@@H](Br)C1 |
| 508 | RL456 | 4-bromo-2-[(1S,2R,5R)-1,2-dimethylbicyclo[3.1.0]hex-2-yl]-5-methylphenol | C[C@]2(CC[C@@H]1C[C@@]12C)c3cc(Br)c(C)cc3O |
| 509 | RL457 | (2S,3aR,5S,6aR)-2-[(1E,3E)-4-bromohexa-1,3-dien-1-yl]-5-[(1R)-3-bromopropadienyl]hexahydrofuro[3,2-b]furan | Br\C(CC)=C\C=C\[C@@H]1C[C@H]2O[C@@H](C[C@H]2O1)\C=C=[C@H]Br |
| 510 | RL458 | (2S,3aR,6aS)-6-bromo-2-[(1R)-3-bromopropadienyl]-5-{(1S,2R)-2-[(2S,3R)-3-methyloxiran-2-yl]cyclopropyl}-2,3,3a,6a-tetrahydrofuro[3,2-b]furan | C[C@H]1O[C@H]1[C@@H]4C[C@@H]4C=2O[C@@H]3C[C@H](O[C@@H]3C=2Br)/C=C=[C@H]Br |
| 511 | RL459 | [4-(4-bromo-3,3-dimethylcyclohexyl)-2,3-dioxabicyclo[2.2.2]oct-5-en-1-yl]methanol | BrC1CCC(CC1(C)C)C23C=CC(CO)(CC2)OO3 |
| 512 | RL460 | [4-(4-bromo-3,3-dimethylcyclohexyl)phenyl]methanol | CC1(C)CC(CCC1Br)c2ccc(CO)cc2 |
| 513 | RL461 | 4'-bromo-1-hydroxy-3',3'-dimethyl-1,1'-bi(cyclohexan)-2-en-4-one | CC1(C)CC(CCC1Br)C2(O)C=CC(=O)CC2 |
| 514 | RL462 | 1-(4-bromo-3,3-dimethylcyclohexyl)-5-chloro-2-oxabicyclo[2.2.2]octane-4,6-diol | OC12CCC(OC1)(C(O)C2Cl)C3CCC(Br)C(C)(C)C3 |
| 515 | RL463 | 5-(4-bromo-3,3-dimethylcyclohexyl)-5-chloro-2-(hydroxymethyl)-7-oxabicyclo[4.1.0]heptan-2-ol | OC2(CO)CCC(Cl)(C1CCC(Br)C(C)(C)C1)C3OC23 |
| 516 | RL464 | 6-(4-bromo-3,3-dimethylcyclohexyl)-3-chloro-3-(hydroxymethyl)-7-oxabicyclo[4.1.0]heptan-2-ol | ClC2(CCC1(OC1C2O)C3CCC(Br)C(C)(C)C3)CO |
| 517 | RL465 | [7-(4-bromo-3,3-dimethylcyclohexyl)-3,8-dioxatricyclo[5.1.0.02,4]oct-4-yl]methanol | BrC1CCC(CC1(C)C)C34CCC2(CO)OC2C4O3 |
| 518 | RL466 | [8,14-bis(acetyloxy)-5-(1-bromo-2-hydroxyethyl)- 1,5- dimethyltetracyclo[8.5.0.0^{2,7}.0^{11,13}]pentadec-2- en-11-yl]methyl acetate | Br[C@@H](CO)[C@@]2(C)CC=C3[C@]4(C)C[C@@H](OC(C)=O)[C@@H]1C[C@]1(COC(C)=O)[C@H]4C[C@@H](OC(C)=O)[C@@H]3C2 |
| 519 | RL467 | 2-[8,14-bis(acetyloxy)-11-[(acetyloxy)methyl]-1,5- dimethyltetracyclo[8.5.0.0^{2,7}.0^{11,13}]pentadec-2- en-5-yl]-2-bromoethyl acetate | CC(=O)OC[C@H](Br)[C@@]2(C)CC=C3[C@]4(C)C[C@@H](OC(C)=O)[C@@H]1C[C@]1(COC(C)=O)[C@H]4C[C@@H](OC(C)=O)[C@@H]3C2 |
| 520 | RL468 | [6-(acetyloxy)-3-(3-bromo-4-hydroxy-2,2- | Br[C@@H](CO)[C@@]2(C)CC=C3[C@]4(C)C[C@@H](OC(C)=O)[C@@H]1C[C@]1(COC(C)=O)[C@H]4C[C@@H](O)[C@@H]3C2 |
| 521 | RL469 | 2-[14-(acetyloxy)-11-[(acetyloxy)methyl]-8-hydroxy- 1,5- dimethyltetracyclo[8.5.0.0^{2,7}.0^{11,13}]pentadec-2- en-5-yl]-2-bromoethyl acetate | CC(=O)OC[C@H](Br)[C@@]2(C)CC=C3[C@]4(C)C[C@@H](OC(C)=O)[C@@H]1C[C@]1(COC(C)=O)[C@H]4C[C@@H](O)[C@@H]3C2 |
| 522 | RL470 | 2-[14-(acetyloxy)-8-hydroxy-1,5,11- trimethyltetracyclo[8.5.0.0^{2,7}.0^{11,13}]pentadec-2- en-5-yl]-2-bromoethyl acetate | CC(=O)OC[C@H](Br)[C@@]2(C)CC=C3[C@]4(C)C[C@@H](OC(C)=O)[C@@H]1C[C@]1(C)[C@H]4C[C@@H](O)[C@@H]3C2 |
| 523 | RL471 | 2-bromo-2-{1,5,11- trimethyltetracyclo[8.5.0.0^{2,7}.0^{11,13}]pentadec-2- en-5-yl}ethan-1-ol | Br[C@@H](CO)[C@]1(C)C[C@H]2[C@H2]C[C@@H]3[C@@]4(C)C[C@H]4[C@H2]C[C@@]3(C)C2=CC1 |
| 524 | RL472 | 2-bromo-2-{1,5,11- trimethyltetracyclo[8.5.0.0^{2,7}.0^{11,13}]pentadec-7- en-5-yl}ethan-1-ol | Br[C@@H](CO)[C@@]4(C)CC[C@H]2C(=CC[C@@H]1[C@@]3(C)C[C@H]3CC[C@]12C)C4 |
| 525 | RL473 | (4S)-4-[(2R,4aR,6R)-6-(5-bromo-2,6,6-trimethyloxan-2-yl)-4a,6,8a-trimethyl-octahydropyrano[3,2-b]pyran-2-yl]-1-[(2R)-5-(2-hydroxypropan-2-yl)-2-methyloxolan-2-yl]pentane-1,4-diol | BrC1CCC(C)(O[C@]1(C)C)[C@@]2(C)CCC3(C)O[C@H](CC[C@@]3(C)O2)[C@@](C)(O)CCC(O)[C@@]4(C)CCC(O4)C(C)(C)O |
| 526 | RL474 | 2-(5-{4-[6-(5-bromo-2,6,6-trimethyloxan-2-yl)-4a,6,8a-trimethyl-octahydropyrano[3,2-b]pyran-2-yl]-1,4-dihydroxypentyl}-5-methyloxolan-2-yl)propan-2-yl acetate | BrC1CCC(C)(O[C@]1(C)C)[C@@]2(C)CCC3(C)O[C@H](CC[C@@]3(C)O2)[C@@](C)(O)CCC(O)[C@@]4(C)CCC(O4)C(C)(C)OC(C)=O |
| 527 | RL475 | (2S,3aR,5R,10aR)-6-bromo-2-(3-bromopropa-1,2-dien-1-yl)-5-ethyl-2H,3H,3aH,5H,6H,7H,10H,10aH-furo[3,2-b]oxonine | Br\C=C=C\[C@@H]1C[C@H]2O[C@H](CC)C(Br)CC=CC[C@H]2O1 |
| 528 | RL476 | 1-bromo-6-[(2E,4E)-6-hydroxy-6-methylhepta-2,4-dien-2-yl]-8a-methyl-4-methylidene-decahydronaphthalen-4a-ol | CC(C)(O)/C=C/C=C(\C)[C@@H]1CC[C@@]2(C)[C@H](Br)CCC(=C)[C@]2(O)C1 |
| 529 | RL477 | (4R,8S,9S)-4,8-dibromo-9-chloro-1,5,5,9-tetramethylspiro[5.5]undec-1-ene | CC2=CC[C@@H](Br)[C@](C)(C)C12CC[C@](C)(Cl)[C@@H](Br)C1 |
| 530 | RL478 | (2Z)-6-bromo-2-[(3E)-1-bromohex-3-en-5-yn-1-ylidene]-5-ethyl-hexahydro-2H-furo[3,2-b]pyran | Br/C(C\C=C\C#C)=C1/CC2O[C@H](CC)C(Br)CC2O1 |
| 531 | RL479 | (2Z)-6-bromo-2-[(3E)-1-bromohex-3-en-5-yn-1-ylidene]-5-ethyl-hexahydro-2H-furo[3,2-b]pyran | Br/C(C\C=C\C#C)=C1/CC2O[C@H](CC)C(Br)CC2O1 |
| 532 | RL480 | (3aS,5R,6S,7aS)-6-bromo-2-[(3E)-1-bromohex-3-en-5-yn-1-yl]-5-ethyl-2-methoxyhexahydro-2H-furo[3,2-b]pyran | BrC(C\C=C\C#C)C1(C[C@@H]2O[C@H](CC)[C@@H](Br)C[C@@H]2O1)OC |
| 533 | RL481 | (2E,5R)-3-bromo-5-(1-bromopropyl)-2-[(3Z)-hex-3-en-5-yn-1-ylidene]hexahydrofuro[3,2-b]furan | BrC(CC)[C@H]1CC2O\C(=C\C/C=C\C#C)C(Br)C2O1 |
| 534 | RL482 | (2E,5R)-3-bromo-5-(1-bromopropyl)-2-[(3Z)-hex-3-en-5-yn-1-ylidene]-hexahydrofuro[3,2-b]furan | BrC(CC)[C@H]1CC2O\C(=C\C/C=C\C#C)C(Br)C2O1 |
| 535 | RL483 | 6-bromo-2-[(3E)-1-bromohex-3-en-5-yn-1-yl]-5-ethyl-hexahydro-2H-furo[3,2-b]pyran-2-ol | BrC(C\C=C\C#C)C1(O)CC2O[C@H](CC)C(Br)CC2O1 |
| 541 | RL489 | (3Z)-8-bromo-3-(bromomethylidene)-7,7,9-trimethyl-11-methylidenespiro[5.5]undec-1-ene | C=C2C[C@@H](C)[C@@H](Br)[C@](C)(C)[C@]12C=CC(CC1)=[C@H]Br |
| 542 | RL490 | (6R,9Z)-9-(bromomethylidene)-1,5,5-trimethylspiro[5.5]undeca-1,7-dien-3-one | CC2=CC(=O)C[C@@](C)(C)[C@]12C=CC(CC1)=[C@H]Br |
| 543 | RL491 | (6R)-1,5,5-trimethylspiro[5.5]undeca-1,7-diene-3,9-dione | CC2=CC(=O)C[C@@](C)(C)[C@]12C=CC(=O)CC1 |
| 544 | RL492 | (2S,3R,9Z)-2-bromo-9-(bromomethylidene)-1,1-dimethyl-5-methylidenespiro[5.5]undecan-3-ol | Br[C@H]=C1CC[C@]2(CC1)C(=C)C[C@@H](O)[C@@H](Br)[C@]2(C)C |
| 545 | RL493 | (6R,9E)-9-(bromomethylidene)-1,5,5-trimethylspiro[5.5]undeca-1,7-dien-3-one | CC2=CC(=O)C[C@@](C)(C)[C@]12C=CC(CC1)=[C@H]Br |
| 546 | RL494 | (2R,3S,6R)-2-bromo-8-chloro-1,1,9-trimethyl-5-methylidenespiro[5.5]undec-8-en-3-ol | ClC=1C[C@]2(CCC=1C)C(=C)C[C@H](O)[C@H](Br)[C@]2(C)C |
| 547 | RL495 | 4,5,6-tribromo-2-methanesulfinyl-3-(methylsulfanyl)-1H-indole | CS(=O)c2nc1cc(Br)c(Br)c(Br)c1c2SC |
| 548 | RL496 | 2,4,6-tribromo-3-methanesulfinyl-1H-indole | CS(=O)c2c1c(cc(Br)cc1Br)nc2Br |
| 549 | RL497 | 4,6-dibromo-2,3-dimethanesulfinyl-1H-indole | CS(=O)c2c1c(cc(Br)cc1Br)nc2S(C)=O |
| 550 | RL498 | 4,6-dibromo-3-(4,6-dibromo-2-methanesulfinyl-1H-indol-3-yl)-2-(methylsulfanyl)-1H-indole | CS(=O)c4nc1cc(Br)cc(Br)c1c4c3c2c(cc(Br)cc2Br)nc3SC |
| 551 | RL499 | 4,6-dibromo-3-(4,6-dibromo-2-methanesulfinyl-1H-indol-3-yl)-2-methanesulfinyl-1H-indole | CS(=O)c4nc1cc(Br)cc(Br)c1c4c3c2c(cc(Br)cc2Br)nc3S(C)=O |
| 552 | BE001 | benzene-1,3,5-triol | Oc1cc(O)cc(O)c1 |
| 553 | BE002 | 5,6,11,14-tetraoxapentaphene-1,3,7,9,12-pentol | Oc5cc(O)cc4Oc1c(cc(O)c2Oc3cc(O)cc(O)c3Oc12)Oc45 |
| 554 | BE003 | 4-(3,5-dihydroxyphenoxy)oxanthrene-1,3,6,8-tetrol | Oc1cc(cc(O)c1)Oc3c(O)cc(O)c2Oc4cc(O)cc(O)c4Oc23 |
| 555 | BE004 | 7,16-bis(3,5-dihydroxyphenoxy)-9,14,21- trioxapentacyclo[11.8.0.0^{2,10}.0^{3,8}.0^{15,20}]henicosa- 1(13),2(10),3(8),4,6,11,15(20),16,18-nonaene- 4,6,12,17,19-pentol | 7,16-bis(3,5-dihydroxyphenoxy)-9,14,21- trioxapentacyclo[11.8.0.0^{2,10}.0^{3,8}.0^{15,20}]henicosa- 1(13),2(10),3(8),4,6,11,15(20),16,18-nonaene- 4,6,12,17,19-pentol |
| 556 | BE005 | 4-(4-{[6-(3,5-dihydroxyphenoxy)-4,7,9-  trihydroxyoxanthren-2-yl]oxy}-3,5-  dihydroxyphenoxy)oxanthrene-1,3,6,8-tetrol | Oc6cc7Oc8c(O)cc(O)c(Oc5cc(O)c(Oc3cc2Oc4c(O)cc(O)c(Oc1cc(O)cc(O)c1)c4Oc2c(O)c3)c(O)c5)c8Oc7c(O)c6 |
| 557 | BS002 | (2R,6R,8R)-5-[4-(3,3-dimethyloxiran-2-yl)-4- hydroxybut-1-en-2-yl]-2,8- dimethyltricyclo[5.3.0.0^{2,6}]decan-3-ol | CC4(C)OC4C(O)CC(=C)C2CC(O)[C@]3(C)C1CC[C@@H](C)C1[C@H]23 |
| 558 | BS003 | (2R,5R,6R,8R)-5-{1-[(2S,3S)-3-[(2S)-3,3- dimethyloxiran-2-yl]oxiran-2-yl]ethenyl}-2,8- dimethyltricyclo[5.3.0.0^{2,6}]decan-3-ol | C[C@@]5(C)O[C@H]5[C@H]4O[C@H]4C(=C)[C@@H]2CC(O)[C@]3(C)C1CC[C@@H](C)C1[C@H]23 |
| 559 | BS004 | (2R,6R,8R)-2,8-dimethyl-5-(6-methylhepta-1,5-dien-2- yl)tricyclo[5.3.0.0^{2,6}]decan-3-ol | C/C(C)=C\CCC(=C)C2CC(O)[C@]3(C)C1CC[C@@H](C)C1[C@H]23 |
| 560 | BS005 | (2R,6R,8R)-5-[(4E)-6-hydroxy-6-methylhepta-1,4- dien-2-yl]-2,8- dimethyltricyclo[5.3.0.0^{2,6}]decan-3-ol | CC(C)(O)/C=C/CC(=C)C2CC(O)[C@]3(C)C1CC[C@@H](C)C1[C@H]23 |
| 561 | BS006 | (2R,6R,8R)-5-(4-hydroxy-6-methylhepta-1,5-dien-2- yl)-2,8-dimethyltricyclo[5.3.0.0^{2,6}]decan-3-ol | C\C(C)=C/C(O)CC(=C)C2CC(O)[C@]3(C)C1CC[C@@H](C)C1[C@H]23 |
| 562 | BS007 | 2,8-dimethyl-3-oxo-5-[(4E)-3,6,7-trihydroxy-6- methylhepta-1,4-dien-2- yl]tricyclo[5.3.0.0^{2,6}]decan-1-yl | CC(O)(CO)/C=C/C(O)C(=C)C2CC(=O)[C@]3(C)C1CC[C@@H](C)C1[C@H]23 |
| 563 | BS008 | (3E)-6-{5-hydroxy-6,10- dimethyltricyclo[5.3.0.0^{2,6}]decan-3-yl}-2- methylhepta-3,6-diene-1,2,5-triol | CC(O)(CO)/C=C/C(O)C(=C)C2CC(O)[C@]3(C)C1CC[C@@H](C)C1[C@H]23 |
| 564 | BS009 | (2E)-4,5-dihydroxy-6-{5-hydroxy-6,10- dimethyltricyclo[5.3.0.0^{2,6}]decan-3-yl}-2- methylhepta-2,6-dien-1-yl acetate | CC(=O)OCC(/C)=C/C(O)C(O)C(=C)C2CC(O)[C@]3(C)C1CC[C@@H](C)C1[C@H]23 |
| 565 | BS010 | 5-(5-hydroxy-6-methylhepta-1,6-dien-2-yl)-2,8- dimethyltricyclo[5.3.0.0^{2,6}]decan-3-ol | C=C(C)C(O)CCC(=C)C2CC(O)[C@]3(C)C1CC[C@@H](C)C1[C@H]23 |
| 566 | BS011 | 5-{1-[3-(3,3-dimethyloxiran-2-yl)oxiran-2- yl]ethenyl}-2,8-dimethyltricyclo[5.3.0.0^{2,6}]dec-4- en-3-ol | C[C@@]5(C)O[C@H]5[C@H]4O[C@H]4C(=C)[C@@H]2CC(O)[C@]3(C)C1CC[C@@H](C)C1[C@H]23 |
| 567 | BS012 | 2-[5-(acetyloxy)-6,10- dimethyltricyclo[5.3.0.0^{2,6}]decan-3-yl]-6- methylhepta-1,5-dien-4-yl acetate | C/C(C)=C/[C@@H](OC(C)=O)CC(=C)[C@@H]2CC(OC(C)=O)[C@]3(C)C1CC[C@@H](C)C1[C@H]23 |
| 568 | BS013 | 5-(4-hydroxy-6-methylhepta-1,5-dien-2-yl)-2,8- dimethyltricyclo[5.3.0.0^{2,6}]decan-3-ol | C/C(C)=C/[C@@H](O)CC(=C)[C@@H]2CC(O)[C@]3(C)C1CC[C@@H](C)C1[C@H]23 |
| 569 | BS014 | (6E,10E,14E)-2-hydroxy-2,6,10-trimethyl-14-[2-(5-methyl-3,6-dioxocyclohexa-1,4-dien-1-yl)ethylidene]-3-oxopentadeca-6,10-dien-1-ylidyne | O=C1C(C)=CC(=O)C=C1C\C=C(/C)CC\C=C(/C)CC\C=C(/C)CCC(=O)C(C)(C)O |
| 570 | BS015 | (6E,10E,14E)-14-[2-(2,5-dihydroxy-3-methylphenyl)ethylidene]-2-hydroxy-2,6,10-trimethyl-3-oxopentadeca-6,10-dien-1-ylidyne | Oc1c(C)cc(O)cc1C\C=C(/C)CC\C=C(/C)CC\C=C(/C)CCC(=O)C(C)(C)O |
| 571 | BS016 | 18-{4-hydroxy-3-methyl-5-[(2E,6E,10Z)-11,14,15-trihydroxy-3,7,15-trimethylhexadeca-2,6,10-trien-1-yl]phenoxy}-18-oxooctadecylidyne | Oc1c(C)cc(cc1C\C=C(/C)CC\C=C(/C)CC\C=C(/O)CC[C@@H](O)C(C)(C)O)OC(=O)CCCCCCCCCCCCCCCCC |
| 572 | BS017 | (9Z)-18-{4-hydroxy-3-methyl-5-[(2E,6E,10Z)-11,14,15-trihydroxy-3,7,15-trimethylhexadeca-2,6,10-trien-1-yl]phenoxy}-18-oxooctadec-9-en-1-ylidyne | Oc1c(C)cc(cc1C\C=C(/C)CC\C=C(/C)CC\C=C(/O)CC[C@@H](O)C(C)(C)O)OC(=O)CCCCCCC/C=C\CCCCCCCC |
| 573 | BS018 | (6E,10E,14E)-14-[2-(2,5-dihydroxy-3-methylphenyl)ethylidene]-2,3-dihydroxy-2,6,10-trimethylpentadeca-6,10-dien-1-ylidyne | Oc1c(C)cc(O)cc1C\C=C(/C)CC\C=C(/C)CC\C=C(/C)CC[C@@H](O)C(C)(C)O |
| 574 | RD001 | (1R)-1-[(5R)-4-bromo-5-(iodomethyl)-5-methoxy-2-oxo-2,5-dihydrofuran-3-yl]butyl acetate | IC[C@]1(OC)OC(=O)C(=C1Br)[C@@H](CCC)OC(C)=O |
| 575 | RD002 | (5R)-4-bromo-3-[(1R)-1-hydroxybutyl]-5-(iodomethyl)-5-methoxy-2,5-dihydrofuran-2-one | IC[C@]1(OC)OC(=O)C(=C1Br)[C@H](O)CCC |
| 576 | RD003 | (5S)-4-bromo-3-[(1R)-1-hydroxybutyl]-5-(iodomethyl)-5-methoxy-2,5-dihydrofuran-2-one | IC[C@@]1(OC)OC(=O)C(=C1Br)[C@H](O)CCC |
| 577 | RD004 | 1-(4-bromo-5-methoxy-5-methyl-2-oxo-2,5-dihydrofuran-3-yl)butyl acetate | BrC1=C(C(=O)O[C@]1(C)OC)[C@@H](CCC)OC(C)=O |
| 578 | RD005 | 1-(4-bromo-5-methoxy-5-methyl-2-oxo-2,5-dihydrofuran-3-yl)butyl acetate | BrC1=C(C(=O)O[C@@]1(C)OC)[C@@H](CCC)OC(C)=O |
| 579 | RD006 | 1-[4-bromo-5-(dibromomethyl)-5-methoxy-2-oxo-2,5-dihydrofuran-3-yl]butyl acetate | BrC(Br)[C@]1(OC)OC(=O)C(=C1Br)[C@@H](CCC)OC(C)=O |
| 580 | RD007 | 1-[4-bromo-5-(dibromomethyl)-5-methoxy-2-oxo-2,5-dihydrofuran-3-yl]butyl acetate | BrC(Br)[C@@]1(OC)OC(=O)C(=C1Br)[C@@H](CCC)OC(C)=O |
| 581 | RD008 | (5R)-4-bromo-3-butyl-5-(dibromomethyl)-5-methoxy-2,5-dihydrofuran-2-one | BrC(Br)[C@]1(OC)OC(=O)C(CCCC)=C1Br |
| 582 | RD009 | 1-[(5Z)-4-bromo-5-(bromomethylidene)-2-oxo-2,5-dihydrofuran-3-yl]butyl acetate | O=C1O/C(=C\Br)C(Br)=C1[C@H](OC(C)=O)CCC |
| 583 | RD010 | 1-[(5E)-4-bromo-5-(bromomethylidene)-2-oxo-2,5-dihydrofuran-3-yl]butyl acetate | O=C1OC(C(Br)=C1C(OC(C)=O)CCC)=[C@H]Br |
| 584 | RD011 | (5Z)-4-bromo-5-(bromomethylidene)-3-(1-hydroxybutyl)-2,5-dihydrofuran-2-one | BrC=1C(=C\Br)\OC(=O)C=1[C@H](O)CCC |
| 585 | RD012 | (5E)-4-bromo-5-(bromomethylidene)-3-(1-hydroxybutyl)-2,5-dihydrofuran-2-one | BrC=1C(=C\Br)/OC(=O)C=1[C@H](O)CCC |
| 586 | RD013 | (5Z)-4-bromo-5-(bromomethylidene)-3-butyl-2,5-dihydrofuran-2-one | O=C1O/C(=C\Br)C(Br)=C1CCCC |
| 587 | RD014 | (5Z)-4-bromo-3-(1-hydroxybutyl)-5-(iodomethylidene)-2,5-dihydrofuran-2-one | BrC=1C(=C\I)\OC(=O)C=1[C@H](O)CCC |
| 588 | RD015 | (5E)-4-bromo-3-[(1R)-1-hydroxybutyl]-5-(iodomethylidene)-2,5-dihydrofuran-2-one | BrC=1C(OC(=O)C=1[C@H](O)CCC)=[C@H]I |
| 589 | RD016 | 1-[(5Z)-4-bromo-5-(iodomethylidene)-2-oxo-2,5-dihydrofuran-3-yl]butyl acetate | O=C1O/C(=C\I)C(Br)=C1[C@H](OC(C)=O)CCC |
| 590 | RD017 | (5Z)-4-bromo-5-(chloromethylidene)-3-(1-hydroxybutyl)-2,5-dihydrofuran-2-one | BrC=1C(=C\Cl)\OC(=O)C=1[C@H](O)CCC |
| 591 | RD018 | (5E)-4-bromo-5-(chloromethylidene)-3-[(1R)-1-hydroxybutyl]-2,5-dihydrofuran-2-one | BrC=1C(OC(=O)C=1[C@H](O)CCC)=[C@H]Cl |
| 592 | RD019 | 4-bromo-5-(dibromomethylidene)-3-(1-hydroxybutyl)-2,5-dihydrofuran-2-one | BrC=1C(\OC(=O)C=1[C@H](O)CCC)=C(/Br)Br |
| 593 | RD020 | 1-[4-bromo-5-(dibromomethylidene)-2-oxo-2,5-dihydrofuran-3-yl]butyl acetate | O=C1O/C(C(Br)=C1[C@H](OC(C)=O)CCC)=C(/Br)Br |
| 594 | RD021 | 4-bromo-3-butyl-5-(dibromomethylidene)-2,5-dihydrofuran-2-one | O=C1O/C(C(Br)=C1CCCC)=C(/Br)Br |
| 595 | RD022 | 7,8,8-tribromo-6-oxooct-7-en-3-yl acetate | O=C(C)OC(CC)CCC(=O)C(\Br)=C(/Br)Br |
| 596 | RD023 | 1,1,2-tribromonon-1-en-3-one | CCCCCCC(=O)C(\Br)=C(/Br)Br |
| 597 | RD024 | (3Z)-1,1,3-tribromododec-3-en-2-ol | BrC(Br)C(O)C(\Br)=C\CCCCCCCC |
| 598 | RD026 | (5E)-4-bromo-5-(bromomethylidene)-3-(2-propyloxiran-2-yl)-2,5-dihydrofuran-2-one | BrC=1C(OC(=O)C=1C2(CCC)CO2)=[C@H]Br |
| 599 | RD027 | (5E)-4-bromo-5-(bromomethylidene)-3-(4-methyloxolan-2-yl)-2,5-dihydrofuran-2-one | CC1CC(OC1)C=2C(=O)OC(=[C@H]Br)C=2Br |
| 600 | RD028 | (5E)-4-bromo-5-(bromomethylidene)-3-(3-ethyloxetan-2-yl)-2,5-dihydrofuran-2-one | CCC2COC2C=1C(=O)OC(=[C@H]Br)C=1Br |
| 601 | RD029 | (5E)-4-bromo-5-(bromomethylidene)-3-(4-methyl-5-oxooxolan-2-yl)-2,5-dihydrofuran-2-one | BrC=2C(OC(=O)C=2C1CC(C)C(=O)O1)=[C@H]Br |
| 602 | RD025 | (5E)-4-bromo-5-(bromomethylidene)-3-(3-ethyl-4-oxooxetan-2-yl)-2,5-dihydrofuran-2-one | BrC=1C(OC(=O)C=1C2OC(=O)C2CC)=[C@H]Br |
| 603 | BS019 | (2R,5S,14R,15R)-14-[(2R,4Z)-6-hydroxy-5-(propan-2- yl)hept-4-en-2-yl]-2,15- dimethyltetracyclo[8.7.0.0^{2,7}.0^{11,15}]heptadec-7- en-5-ol | CC(C)\C(=C\C[C@@H](C)[C@H]1CCC2C3CC=C4C[C@@H](O)CC[C@]4(C)C3CC[C@]12C)C(C)O |
| 604 | BS020 | (2R,6R,14R)-6-hydroxy-2,14-dimethyl-13-[(5E)-5- (propan-2-yl)hept-5-en-2-yl]-5- oxatetracyclo[7.7.0.0^{2,6}.0^{10,14}]hexadecan-4- one | CC(C)C(=C\C)\CC[C@H](C)C1CCC2C3CC[C@@]4(O)OC(=O)C[C@]4(C)C3CC[C@]12C |
| 605 | BP002 | 3-hydroxy-1-methoxy-8-methyl-5-(6-methylhept-5-en-2-yl)-1H,3H,3aH,4H,5H,6H,7H,10H-cyclodeca[c]furan-6-yl acetate | CC(=O)OC1CC(C)=CCC=C2C(CC1C(C)CC\C=C(/C)C)C(O)OC2OC |
| 606 | BP003 | 1,3-dimethoxy-7-methyl-4-(6-methylhept-5-en-2-yl)-1H,3H,3aH,4H,5H,6H,9H-cyclonona[c]furan-5-ol | C/C(C)=C\CCC(C)C1CC2C(OC)OC(OC)C2=CCC=C(C)CC1O |
| 607 | RG016 | (2R)-N-[(3S,4R,6E)-11-(2-butylcyclopropyl)-3,4-dihydroxy-1-{[(3S,5S)-3,4,5-trihydroxy-6-(hydroxymethyl)oxan-2-yl]oxy}undec-6-en-2-yl]-2-hydroxypentacosanamide | CCCCC2CC2CCCC\C=C\C[C@@H](O)[C@@H](O)C(COC1OC(CO)[C@@H](O)C(O)[C@@H]1O)NC(=O)[C@H](O)CCCCCCCCCCCCCCCCCCCCCCC |
| 608 | RG017 | N-[(6E)-11-(2-butylcyclopropyl)-1,3,4-trihydroxyundec-6-en-2-yl]-2-hydroxy-16-(octyloxy)hexadecanamide | CCCCCCCCCCCCCCCCCCCCCCC[C@@H](O)C(=O)NC(CO)[C@H](O)[C@H](O)C/C=C/CCCCC1CC1CCCC |
| 609 | RG018 | (2R)-10-(2-butylcyclopropyl)-N-[(4R)-1,4-dihydroxy-3-methylhexacosan-2-yl]-2-hydroxydecanamide | CCCCCCCCCCCCCCCCCCCCCC[C@@H](O)[C@@H](O)C(CO)NC(=O)[C@H](O)CCCCCCCCC1CC1CCCC |
| 610 | RP017 | 3,4-dibromo-5-(hydroxymethyl)benzene-1,2-diol | Brc1c(CO)cc(O)c(O)c1Br |
| 611 | RP018 | 2,3-dibromo-4,5-dihydroxybenzaldehyde | Brc1c(C=O)cc(O)c(O)c1Br |
| 612 | RP003 | 3,6-dibromo-4-(hydroxymethyl)benzene-1,2-diol | Oc1c(Br)c(CO)cc(Br)c1O |
| 613 | RP004 | 3,4-dihydroxybenzaldehyde | Oc1ccc(C=O)cc1O |
| 614 | RP005 | 3-bromo-4,5-dihydroxybenzaldehyde | Oc1cc(cc(Br)c1O)C=O |
| 615 | RP006 | 2,5-dibromo-3,4-dihydroxybenzaldehyde | Oc1c(Br)c(cc(Br)c1O)C=O |
| 616 | RP007 | tetrabromobenzene-1,2-diol | Brc1c(O)c(O)c(Br)c(Br)c1Br |
| 617 | RP008 | 4-hydroxy-3-methoxybenzaldehyde | Oc1ccc(cc1OC)C=O |
| 618 | RP009 | 3-bromo-4-hydroxy-5-methoxybenzaldehyde | Oc1c(OC)cc(cc1Br)C=O |
| 619 | RP010 | 2,3-dibromo-4-hydroxy-5-methoxybenzaldehyde | Brc1c(C=O)cc(OC)c(O)c1Br |
| 620 | RP011 | 3-bromo-5-(hydroxymethyl)benzene-1,2-diol | Oc1cc(CO)cc(Br)C1o |
| 621 | RG019 | (3R,6R,9R,12R,15R)-1,7-dimethyl-3,6,9,12,15-pentakis(2-methylpropyl)-1,4,7,10,13-pentaazacyclopentadecane-2,5,8,11,14-pentone | CC(C)C[C@H]1NC(=O)[C@@H](CC(C)C)N(C)C(=O)[C@H](NC(=O)[C@H](NC(=O)[C@@H](CC(C)C)N(C)C1=O)CC(C)C)CC(C)C |
| 622 | BS021 | (1S,11R,20R)-1,8,11,15,19,19-hexamethyl-10- oxapentacyclo[12.8.0.0^{2,11}.0^{4,9}.0^{15,20}]docosa- 4,6,8-triene-6,18-diol | Oc2cc3CC4[C@@]5(C)CC[C@H]1[C@](C)(C)C(O)CCC1(C)C5CC[C@@]4(C)Oc3c(C)c2 |
| 623 | BS022 | (1S,2S,14S,19R)-5-methoxy-1,6,10,14,18,18- hexamethylpentacyclo[11.8.0.0^{2,10}.0^{4,9}.0^{14,19}]henicosa- 4(9),5,7-triene-8,17-diol | Oc4cc(C)c(OC)c5C[C@@H]3C(C)(CCC2[C@]3(C)CC[C@@H]1[C@@]2(C)CCC(O)[C@@]1(C)C)c45 |
| 624 | BS023 | (1S,2S,14S,19R)-17-hydroxy-1,6,10,14,18,18- hexamethylpentacyclo[11.8.0.0^{2,10}.0^{4,9}.0^{14,19}]henicosa- 4(9),6-diene-5,8-dione | O=C4C=C(C)C(=O)C=5C[C@@H]3C(C)(CCC2[C@]3(C)CC[C@@H]1[C@@]2(C)CCC(O)[C@@]1(C)C)C4=5 |
| 625 | BS024 | (2S,2'S,8'aR)-4,5,7'-trihydroxy-2',4'b,8',8',10'a-pentamethyl-3',4',4'a,4'b,5',6',7',8',8'a,9',10',10'a-dodecahydro-2'H,3H-spiro[1-benzofuran-2,1'-phenanthrene]-7-carbaldehyde | Oc1c(O)cc(C=O)c2O[C@@]4(Cc12)[C@@H](C)CCC3C5(C)CCC(O)[C@@](C)(C)[C@@H]5CCC34C |
| 626 | BS025 | (2S,2'S,8'aR)-3a-hydroperoxy-7'-hydroxy-5-methoxy-2',4'b,7,8',8',10'a-hexamethyl-3',3a,4,4',4'a,4'b,5',6',7',8',8'a,9',10',10'a-tetradecahydro-2'H,3H-spiro[1-benzofuran-2,1'-phenanthrene]-4-one | O=C1C(OC)=CC(C)=C2O[C@@]4(CC12OO)[C@@H](C)CCC3C5(C)CCC(O)[C@@](C)(C)[C@@H]5CCC34C |
| 627 | BS026 | (2S,2'S,8'aR)-7'-hydroxy-2',4'b,7,8',8',10'a-hexamethyl-3',4,4',4'a,4'b,5,5',6',7',8',8'a,9',10',10'a-tetradecahydro-2'H,3H-spiro[1-benzofuran-2,1'-phenanthrene]-4,5-dione | O=C1C(=O)C=C(C)C=2O[C@@]4(CC1=2)[C@@H](C)CCC3C5(C)CCC(O)[C@@](C)(C)[C@@H]5CCC34C |
| 628 | BS027 | 2-methyl-6-[(2E,6E,10E)-3,7,11,15-tetramethylhexadeca-2,6,10,14-tetraen-1-yl]cyclohexa-2,5-diene-1,4-dione | O=C1C(C)=CC(=O)C=C1C\C=C(/C)CC\C=C(/C)CC\C=C(/C)CC\C=C(/C)C |
| 629 | BS028 | (2R,2'S,8'aR)-2',4'b,7,8',8',10'a-hexamethyl-3',4',4'a,4'b,5',6',7',8',8'a,9',10',10'a-dodecahydro-2'H,3H-spiro[1-benzofuran-2,1'-phenanthrene]-5,7'-diol | Oc1cc2C[C@@]4(Oc2c(C)c1)[C@@H](C)CCC3C5(C)CCC(O)[C@@](C)(C)[C@@H]5CCC34C |
| 630 | BS029 | (2S,2'S,8'aR)-2',4'b,7,8',8',10'a-hexamethyl-3',4',4'a,4'b,5',6',7',8',8'a,9',10',10'a-dodecahydro-2'H,3H-spiro[1-benzofuran-2,1'-phenanthrene]-5,7'-diol | Oc1cc2C[C@]4(Oc2c(C)c1)[C@@H](C)CCC3C5(C)CCC(O)[C@@](C)(C)[C@@H]5CCC34C |
| 631 | BS030 | (2S,2'S,8'aR)-2',4'b,7,8',8',10'a-hexamethyl-3',4',4'a,4'b,5',6',7',8',8'a,9',10',10'a-dodecahydro-2'H,3H-spiro[1-benzofuran-2,1'-phenanthrene]-4,5,7'-triol | Oc1c(O)cc(C)c2O[C@@]4(Cc12)[C@@H](C)CCC3C5(C)CCC(O)[C@@](C)(C)[C@@H]5CCC34C |
| 632 | BS031 | (1R,2S,11S,14R,15R,18S,20R)-1,8,11,15,19,19- hexamethyl-10-oxapentacyclo[12.8.0.0^{2,11}.0^{4,9}.0^{15,20}]docosa-4,6,8-triene-6,18-diol | Oc1cc2C[C@H]3[C@]4(C)CC[C@H]5[C@](C)(C)[C@@H](O)CC[C@]5(C)[C@H]4CC[C@]3(C)Oc2c(C)c1 |
| 633 | BS032 | (1S,2S,11R,20R)-1,8,11,15,19,19-hexamethyl-10- oxapentacyclo[12.8.0.0^{2,11}.0^{4,9}.0^{15,20}]docosa- 4,6,8-triene-6,18-diol | Oc2cc3C[C@H]4[C@@]5(C)CC[C@H]1[C@](C)(C)C(O)CCC1(C)C5CC[C@@]4(C)Oc3c(C)c2 |
| 634 | BS033 | (1S,2S,11R,20R)-1,8,11,15,19,19-hexamethyl-10- oxapentacyclo[12.8.0.0^{2,11}.0^{4,9}.0^{15,20}]docosa- 4,6,8-triene-6,18-diol | Oc2cc3C[C@H]4[C@@]5(C)CC[C@H]1[C@](C)(C)C(O)CCC1(C)C5CC[C@@]4(C)Oc3c(C)c2 |
| 635 | BS034 | (2S,11R,20R)-1,8,11,15,19,19-hexamethyl-10- oxapentacyclo[12.8.0.0^{2,11}.0^{4,9}.0^{15,20}]docosa- 4,6,8-triene-6,18-diol | Oc1cc2C[C@H]3C4(C)CC[C@H]5[C@](C)(C)C(O)CCC5(C)C4CC[C@@]3(C)Oc2c(C)c1 |
| 636 | BS035 | (1S,11R,20R)-6-(acetyloxy)-1,8,11,15,19,19- hexamethyl-10- oxapentacyclo[12.8.0.0^{2,11}.0^{4,9}.0^{15,20}]docosa- 4,6,8-trien-18-yl acetate | CC(=O)Oc2cc3CC4[C@@]5(C)CC[C@H]1[C@](C)(C)C(OC(C)=O)CCC1(C)C5CC[C@@]4(C)Oc3c(C)c2 |
| 637 | BS036 | 1S,2S,14S,19R)-8-(acetyloxy)-5-methoxy- 1,6,10,14,18,18- hexamethylpentacyclo[11.8.0.0^{2,10}.0^{4,9}.0^{14,19}]henicosa- 4(9),5,7-trien-17-yl acetate | CC(=O)Oc4cc(C)c(OC)c5C[C@@H]3C(C)(CCC2[C@]3(C)CC[C@@H]1[C@@]2(C)CCC(OC(C)=O)[C@@]1(C)C)c45 |
| 638 | BT004 | (2R,5S,15R)-2,15-dimethyl-14-[(2R,5E)-5-(propan-2- yl)hept-5-en-2- yl]tetracyclo[8.7.0.0^{2,7}.0^{11,15}]heptadec-7- en-5-ol | CC(C)C(=C\C)\CC[C@@H](C)C1CCC2C3CC=C4C[C@@H](O)CC[C@]4(C)C3CC[C@]12C |
| 639 | BT005 | (2R,5S,15R)-14-[(2R)-5-hydroperoxy-5-(propan-2- yl)hept-6-en-2-yl]-2,15- dimethyltetracyclo[8.7.0.0^{2,7}.0^{11,15}]heptadec-7- en-5-ol | CC(C)C(C=C)(OO)CC[C@@H](C)C1CCC2C3CC=C4C[C@@H](O)CC[C@]4(C)C3CC[C@]12C |
| 640 | BT006 | (2R,5S,15R)-14-[(2R,5E)-7-hydroperoxy-5-(propan-2- yl)hept-5-en-2-yl]-2,15- dimethyltetracyclo[8.7.0.0^{2,7}.0^{11,15}]heptadec-7- en-5-ol | CC(C)C(=C\COO)\CC[C@@H](C)C1CCC2C3CC=C4C[C@@H](O)CC[C@]4(C)C3CC[C@]12C |
| 641 | BT007 | (2R,15R)-2,15-dimethyl-14-[(2R,5E)-5-(propan-2- yl)hept-5-en-2- yl]tetracyclo[8.7.0.0^{2,7}.0^{11,15}]heptadec-6- en-5-one | CC(C)C(=C\C)\CC[C@@H](C)C2CCC1C3CCC4=CC(=O)CC[C@]4(C)C3CC[C@@]12C |
| 642 | BT008 | (2R,15R)-14-[(2R)-5-hydroperoxy-5-(propan-2- yl)hept-6-en-2-yl]-2,15- dimethyltetracyclo[8.7.0.0^{2,7}.0^{11,15}]heptadec-6- en-5-one | CC(C)C(C=C)(OO)CC[C@@H](C)C2CCC1C3CCC4=CC(=O)CC[C@]4(C)C3CC[C@@]12C |
| 643 | BT009 | (2R,15R)-2,15-dimethyl-14-[(2R,5E)-5-(propan-2- yl)hept-5-en-2- yl]tetracyclo[8.7.0.0^{2,7}.0^{11,15}]heptadec-6- ene-5,8-dione | CC(C)C(=C\C)\CC[C@@H](C)C2CCC1C3CC(=O)C4=CC(=O)CC[C@]4(C)C3CC[C@@]12C |
| 644 | BT010 | (2R,15R)-14-[(2R)-5-hydroperoxy-5-(propan-2- yl)hept-6-en-2-yl]-2,15- dimethyltetracyclo[8.7.0.0^{2,7}.0^{11,15}]heptadec-6- ene-5,8-dione | CC(C)C(C=C)(OO)CC[C@@H](C)C2CCC1C3CC(=O)C4=CC(=O)CC[C@]4(C)C3CC[C@@]12C |
| 645 | BT011 | (2R,15R)-8-hydroxy-2,15-dimethyl-14-[(2R,5E)-5- (propan-2-yl)hept-5-en-2- yl]tetracyclo[8.7.0.0^{2,7}.0^{11,15}]heptadec-6- en-5-one | CC(C)C(=C\C)\CC[C@@H](C)C2CCC3C4CC(O)C1=CC(=O)CC[C@]1(C)C4CC[C@]23C |
| 646 | BT012 | (2R,15R)-14-[(2R)-5-hydroperoxy-5-(propan-2- yl)hept-6-en-2-yl]-8-hydroxy-2,15- dimethyltetracyclo[8.7.0.0^{2,7}.0^{11,15}]heptadec-6- en-5-one | CC(C)C(C=C)(OO)CC[C@@H](C)C2CCC3C4CC(O)C1=CC(=O)CC[C@]1(C)C4CC[C@]23C |
| 647 | BS037 | methyl 3-[(1S,4aR,5S,6S,8aR)-5-[(2-hydroxy-5-methoxy-3-methylphenyl)methyl]-5,6,8a-trimethyl-2-oxo-decahydronaphthalen-1-yl]propanoate | O=C(OC)CC[C@@H]2C(=O)CC[C@@H]3[C@@](C)(Cc1cc(OC)cc(C)c1O)[C@@H](C)CC[C@@]23C |
| 648 | BS038 | methyl 3-[(1S,4aR,5S,6S,8aR)-5-[(2,5-dihydroxy-3-methylphenyl)methyl]-5,6,8a-trimethyl-2-oxo-decahydronaphthalen-1-yl]propanoate | O=C(OC)CC[C@@H]2C(=O)CC[C@@H]3[C@@](C)(Cc1cc(O)cc(C)c1O)[C@@H](C)CC[C@@]23C |
| 649 | BS039 | methyl 3-[(1S,4aR,5S,6S,8aR)-5-[(2,5-dihydroxy-3-methylphenyl)methyl]-5,6,8a-trimethyl-2-(propan-2-ylidene)-decahydronaphthalen-1-yl]propanoate | C/C(C)=C2\CC[C@@H]3[C@@](C)(Cc1cc(O)cc(C)c1O)[C@@H](C)CC[C@@]3(C)[C@@H]2CCC(=O)OC |
| 650 | BD007 | 3S,3aR,5S)-3,5-dihydroxy-7-methyl-4-(6-methylhept-5-en-2-yl)-1H,3H,3aH,4H,5H,6H,9H-cyclonona[c]furan-1-one | C/C(C)=C\CCC(C)[C@H]2[C@@H](O)CC(C)=CCC=C1C(=O)O[C@H](O)[C@@H]12 |
| 651 | BD008 | (3aS,5R,6S,8aR)-5-(2-hydroxy-6-methylhept-5-en-2-yl)-3-methyl-8-methylidene-1,3a,4,5,6,7,8,8a-octahydroazulene-4,6-diol | C/C(C)=C\CCC(C)(O)[C@@H]2[C@@H](O)CC(=C)\[C@@H]1CC=C(C)[C@H]1[C@@H]2O |
| 652 | BD009 | 3-hydroxy-4-[(3E,5E,7E,9E,11E,13E,15E)-18-{4-  hydroxy-2,2,6-trimethyl-7-oxabicyclo[4.1.0]heptan-1-  yl}-3,7,12,16-tetramethyl-17-oxooctadeca-  1,3,5,7,9,11,13,15-octaen-1-ylidene]-3,5,5-  trimethylcyclohexyl acetate | CC(=O)OC3CC(C)(C)\C(=C=CC(\C)=C\C=C\C(\C)=C\C=C\C=C(/C)\C=C\C=C(/C)C(=O)CC12OC2(C)CC(O)CC1(C)C)C(C)(O)C3 |
| 653 | BD010 | (2S,4R,6S,8R,9R)-8-hydroxy-6-methyl-9-[(2R)-6- methylhept-5-en-2-yl]-12- oxatricyclo[8.3.0.0^{2,4}]tridec-1(10)-en-13-one | C/C(C)=C\CC[C@@H](C)[C@H]3[C@H](O)C[C@@H](C)C[C@@H]1C[C@@H]1C2=C3COC2=O |
| 654 | BD011 | (1E,4R,6S,9R,10R,11R)-11,13-dimethoxy-6-methyl-9- [(2S)-6-methylhept-5-en-2-yl]-5,12- dioxatricyclo[8.3.0.0^{4,6}]tridec-1-ene | C/C(C)=C\CC[C@H](C)[C@H]1CC[C@@]3(C)O[C@H]3CC=C2[C@@H]1[C@H](OC)OC2OC |
| 655 | BD012 | (3R,3aR,4R)-1,3-dimethoxy-7-methyl-4-[(2S)-6-methylhept-5-en-2-yl]-1H,3H,3aH,4H,5H,6H,9H-cyclonona[c]furan | C/C(C)=C\CC[C@H](C)[C@H]1CCC(C)=CCC=C2[C@@H]1[C@H](OC)OC2OC |
| 656 | BE006 | benzene-1,3,5-triol | Oc1cc(O)cc(O)c1 |
| 657 | BE007 | 4-(3,5-dihydroxyphenoxy)oxanthrene-1,3,6,8-tetrol | Oc1cc(cc(O)c1)Oc3c(O)cc(O)c2Oc4cc(O)cc(O)c4Oc23 |
| 658 | BE008 | 2-{2-[3,5-dihydroxy-2-(2-hydroxy-4,6-dimethylphenyl)phenoxy]-3,5-dihydroxyphenoxy}benzene-1,3,5-triol | Oc4cc(O)cc(O)c4c3c(Oc2c(O)cc(O)cc2Oc1c(O)cc(O)cc1O)cc(O)cc3O |
| 659 | BE009 | 7,16-bis(3,5-dihydroxyphenoxy)-9,14,21- trioxapentacyclo[11.8.0.0^{2,10}.0^{3,8}.0^{15,20}]henicosa- 1,3(8),4,6,10,12,15(20),16,18-nonaene-4,6,12,17,19- pentol | Oc1cc(cc(O)c1)Oc5c(O)cc(O)c4c6c3Oc7c(O)cc(O)c(Oc2cc(O)cc(O)c2)c7Oc3c(O)cc6oc45 |
| 660 | BE010 | 4-(3,5-dihydroxyphenoxy)-8-(2,4,6-trihydroxyphenoxy)oxanthrene-1,3,6-triol | Oc5cc(O)cc(O)c5Oc3cc2Oc4c(O)cc(O)c(Oc1cc(O)cc(O)c1)c4Oc2c(O)c3 |
| 661 | BE011 | 4-(4-{[6-(3,5-dihydroxyphenoxy)-4,7,9-trihydroxyoxanthren-2-yl]oxy}-3,5-dihydroxyphenoxy)oxanthrene-1,3,6,8-tetrol | Oc6cc7Oc8c(O)cc(O)c(Oc5cc(O)c(Oc3cc2Oc4c(O)cc(O)c(Oc1cc(O)cc(O)c1)c4Oc2c(O)c3)c(O)c5)c8Oc7c(O)c6 |
| 662 | BE012 | 4-(3,5-dihydroxyphenoxy)-9-[6-(3,5-  dihydroxyphenoxy)-2,4,7,9-tetrahydroxyoxanthren-1-  yl]oxanthrene-1,3,6,8-tetrol | Oc1cc(cc(O)c1)Oc2c(O)cc(O)c3Oc4c(Oc23)c(O)cc(O)c4c7c6Oc8c(O)cc(O)c(Oc5cc(O)cc(O)c5)c8Oc6c(O)cc7O |
| 663 | BD013 | (1E,4E,7R,8S,9S)-7-hydroxy-9-[(R)- hydroxy[(2S,4S,5R,7R,8S,12R)-7-hydroxy-5-methyl-8- [(2R)-6-methylhept-5-en-2-yl]-10-oxo-11- oxatricyclo[7.3.0.0^{2,4}]dodec-1(9)-en-12- yl]methyl]-5-methyl-8-[(2R)-6-methylhept-5-en-2- yl]cyclonona-1,4-diene-1-carbaldehyde | C/C(C)=C\CC[C@@H](C)[C@@H]4[C@H](O)CC(C)=CCC=C(C=O)[C@H]4[C@@H](O)[C@@H]3OC(=O)C1=C3[C@H]2C[C@H]2[C@H](C)C[C@@H](O)[C@H]1[C@H](C)CC\C=C(/C)C |
| 664 | BD014 | (2S,4S,5R,8S)-5-methyl-8-[(2R)-6-methylhept-5-en-2- yl]-11-oxatricyclo[7.3.0.0^{2,4}]dodec-1(9)-en-10- one | C/C(C)=C\CC[C@@H](C)[C@@H]3[C@H2]C[C@@H](C)[C@@H]1C[C@@H]1C=2COC(=O)C=23 |
| 665 | BD015 | (2S,4S,5R,7R,8S)-5-methyl-8-[(2R)-6-methylhept-5- en-2-yl]-10-oxo-11-oxatricyclo[7.3.0.0^{2,4}]dodec- 1(9)-en-7-yl acetate | C/C(C)=C\CC[C@@H](C)[C@@H]3[C@@H](C[C@@H](C)[C@@H]1C[C@@H]1C=2COC(=O)C=23)OC(C)=O |
| 666 | BD016 | [(1S,2E,5E,8R,9S)-2-formyl-8-hydroxy-6-methyl-9-[(2R)-6-methylhept-5-en-2-yl]cyclonona-2,5-dien-1-yl]methyl acetate | CC(=O)OC[C@@H]1C(=CCC=C(C)C[C@@H](O)[C@H]1[C@H](C)CC\C=C(/C)C)C=O |
| 667 | BD017 | (1S,2E,5E,8R,9S)-8-hydroxy-6-methyl-9-[(2R)-6-methylhept-5-en-2-yl]cyclonona-2,5-diene-1,2-dicarbaldehyde | C/C(C)=C\CC[C@@H](C)[C@@H]1[C@H](O)CC(C)=CCC=C(C=O)[C@H]1C=O |
| 668 | BD018 | [(1S,3E,6S,7R,9S)-4-formyl-7-hydroxy-9-methyl-6-[(2R)-6-methylhept-5-en-2-yl]-10-oxabicyclo[7.1.0]dec-3-en-5-yl]methyl acetate | CC(=O)OC[C@@H]1C(=CC[C@@H]2O[C@]2(C)C[C@@H](O)[C@H]1[C@H](C)CC\C=C(/C)C)C=O |
| 669 | BD019 | (1E,4S,6S,9S,10S,11S)-6-methyl-9-[(2R)-6- methylhept-5-en-2-yl]-5,12-dioxatricyclo[8.3.0.0^{4,6}]tridec-1-en-11-ol | C/C(C)=C\CC[C@@H](C)[C@@H]2CC[C@@]3(C)O[C@H]3CC=C1CO[C@H](O)[C@H]12 |
| 670 | BD020 | [(2E,5E,8S)-2-formyl-8-hydroxy-6-methyl-9-[(2S)-6-methylhept-5-en-2-yl]cyclonona-2,5-dien-1-yl]methyl acetate | CC(=O)OC[C@H]1C(=CCC=C(C)C[C@H](O)[C@@H]1[C@@H](C)CC\C=C(/C)C)C=O |
| 671 | BD021 | (1S,10R,10aR)-7-methyl-10-[(2R)-6-methylhept-5-en-2-yl]-1H,3H,5H,8H,9H,10H,10aH-cyclonona[c]furan-1-ol | C/C(C)=C\CC[C@@H](C)[C@H]2CCC(C)=CCC=C1CO[C@H](O)[C@@H]12 |
| 672 | RL500 | (3R,4S)-4-bromo-3-chloro-3,7,7-trimethyl-11-methylidene-10-oxospiro[5.5]undec-8-en-1-yl acetate | CC(=O)O[C@@H]1C[C@@](C)(Cl)[C@@H](Br)CC12C(=C)C(=O)C=C[C@]2(C)C |
| 673 | RL501 | (8S,9R)-8-bromo-9-chloro-5,5,9-trimethyl-1-methylidenespiro[5.5]undec-3-en-2-one | C=C2C(=O)C=C[C@](C)(C)C12CC[C@@](C)(Cl)[C@@H](Br)C1 |
| 674 | RL502 | 2-[(1R,3S)-1,3-dimethyl-2-methylidenecyclopentyl]-5-methylphenol | C[C@]1(CC[C@H](C)C1=C)c2ccc(C)cc2O |
| 675 | RL503 | (9E)-2-bromo-9-(bromomethylidene)-1,1-dimethyl-5-methylidenespiro[5.5]undec-2-en-3-ol | C=C2CC(O)=C(Br)[C@](C)(C)[C@]12CCC(CC1)=[C@H]Br |
| 676 | RL504 | (6R,9Z)-9-(bromomethylidene)-1,5,5-trimethylspiro[5.5]undeca-1,7-dien-3-one | CC2=CC(=O)C[C@@](C)(C)[C@]12C=CC(CC1)=[C@H]Br |
| 677 | RL505 | (2R,3S,6S,9Z)-2-bromo-9-(bromomethylidene)-1,1-dimethyl-5-methylidenespiro[5.5]undec-7-en-3-ol | Br[C@H]=C1CC[C@]2(C=C1)C(=C)C[C@H](O)[C@H](Br)[C@]2(C)C |
| 678 | RL506 | (2R,3S,6S,9E)-2-bromo-9-(bromomethylidene)-1,1-dimethyl-5-methylidenespiro[5.5]undec-7-en-3-ol | Br[C@H]=C1CC[C@]2(C=C1)C(=C)C[C@H](O)[C@H](Br)[C@]2(C)C |
| 679 | RL507 | (1R,3S,4S,6R,8R,11R)-3,8-dibromo-4-chloro- 4,11,12,12-tetramethyl-7- oxatricyclo[6.3.1.0^{1,6}]dodec-9-en-11-ol | O[C@]3(C)C=C[C@]2(Br)OC1C[C@](C)(Cl)[C@@H](Br)C[C@@]13[C@]2(C)C |
| 680 | RL508 | (1S,2R,6R)-4,5'-dibromo-1,3,3,4'-tetramethyl-7-oxaspiro[bicyclo[4.1.0]heptane-2,1'-cyclohexane]-4,4'-dien-2'-ol | C[C@@]3(C)C(Br)=C[C@H]1O[C@@]1(C)[C@@]23CC(Br)=C(C)C[C@@H]2O |
| 681 | RL509 | (3R,4S,6S,8S,9S)-4,8-dibromo-9-chloro-1,5,5,9-tetramethylspiro[5.5]undec-1-en-3-ol | CC2=C[C@@H](O)[C@@H](Br)[C@](C)(C)[C@@]12CC[C@](C)(Cl)[C@@H](Br)C1 |
| 682 | RL510 | (3S,4S,6R)-4-bromo-8-chloro-1,5,5,9-tetramethylspiro[5.5]undeca-1,8-dien-3-ol | ClC=1C[C@]2(CCC=1C)C(C)=C[C@H](O)[C@@H](Br)[C@@]2(C)C |
| 683 | RL511 | (3S,4S,6R,9S)-4,9-dibromo-8-chloro-1,5,5,9-tetramethylspiro[5.5]undec-1-en-3-ol | Br[C@@]1(C)CC[C@@]2(CC1Cl)C(C)=C[C@H](O)[C@@H](Br)[C@@]2(C)C |
| 539 | RL487 | (1S,3R)-4-[(2R,4aR,6R,8aS)-6-[(2S,5R)-5-bromo-2,6,6-trimethyloxan-2-yl]-8a-methyl-octahydropyrano[3,2-b]pyran-2-yl]-1-[(2R,5R)-5-(2-hydroxypropan-2-yl)-2-methyloxolan-2-yl]pent-4-ene-1,3-diol | Br[C@@H]1CC[C@](C)(O[C@]1(C)C)[C@H]2CC[C@]3(C)O[C@H](CC[C@H]3O2)C(=C)[C@H](O)C[C@H](O)[C@@]4(C)CC[C@@H](O4)C(C)(C)O |
| 538 | RL486 | (1S)-4-[(1R,3R,6R,8S,11S,14R)-14-bromo-1,8,13,13- tetramethyl-2,7,12- trioxatricyclo[9.5.0.0^{3,8}]hexadecan-6-yl]-1- [(2R,5R)-5-(2-hydroxypropan-2-yl)-2-methyloxolan-2- yl]pent-4-en-1-ol | CC(C)(O)[C@H]1CC[C@@](C)(O1)[C@@H](O)CCC(=C)[C@H]2CC[C@H]3O[C@]4(C)CC[C@@H](Br)[C@@](C)(C)O[C@H]4CC[C@]3(C)O2 |
| 536 | RL484 | 5-[(2R,6S)-6-[(2R,4aR,8aS)-6-[(2S,5R)-5-hydroxy-2,6,6-trimethyloxan-2-yl]-8a-methyl-2H,3H,4H,4aH,8H,8aH-pyrano[3,2-b]pyran-2-yl]-2,6-dimethyl-5,6-dihydro-2H-pyran-2-yl]-2-methylpentane-2,3-diol | CC(C)(O)[C@H](O)CC[C@]1(C)C=CC[C@](C)(O1)[C@@H]2O[C@@]3(C)CC=C(O[C@@H]3CC2)[C@]4(C)CC[C@@H](O)[C@@](C)(C)O4 |
| 537 | RL485 | (1S)-4-[(2R,4aR,6R,8aS)-6-[(2S,5S)-5-(2-hydroxypropan-2-yl)-2-methyloxolan-2-yl]-8a-methyl-octahydropyrano[3,2-b]pyran-2-yl]-1-[(2R,5R)-5-(2-hydroxypropan-2-yl)-2-methyloxolan-2-yl]pent-4-en-1-ol | CC(C)(O)[C@H]1CC[C@@](C)(O1)[C@@H](O)CCC(=C)[C@H]2CC[C@H]3O[C@H](CC[C@]3(C)O2)[C@]4(C)CC[C@H](O4)C(C)(C)O |
| 695 | RL523 | (2Z)-2-chloropentadec-2-enal | Cl/C(=C\CCCCCCCCCCCC)C=O |
| 696 | RL524 | (3R)-3,7,7-trimethyl-11-methylidenespiro[5.5]undec-8-en-2-one | O=C2[C@H](C)[C@@H2][C@H2]C1(C(=C)\[C@@H2]C=C[C@@]1(C)C)[C@H2]2 |
| 697 | RL525 | (2R,3R,6R,8S)-2,8-dibromo-3,7,7-trimethyl-11-methylidenespiro[5.5]undecan-3-ol | C=C2CC[C@H](Br)[C@@](C)(C)[C@]12CC[C@@](C)(O)[C@H](Br)C1 |
| 698 | RL526 | (3Z)-1-bromo-5-(2,2-dimethyl-6-methylidenecyclohex-3-en-1-yl)-3-methylpent-3-en-2-ol | C[C@@]1(C)C=CCC(=C)C1C\C=C(\C)C(O)CBr |
| 699 | RL527 | 2-[(1S,2R,5R)-1,2-dimethylbicyclo[3.1.0]hexan-2-yl]-5-methylphenol | C[C@]2(CC[C@@H]1C[C@@]12C)c3ccc(C)cc3O |
| 700 | RL528 | 8-bromo-3,7,7-trimethyl-11-methylidenespiro[5.5]undecan-2-one | C=C2CCC(Br)C(C)(C)C12CC(=O)C(C)CC1 |
| 701 | RL529 | (1S,3aS,4R,5S,7aR)-1,5-dimethyl-4-(2-methylprop-1-en-1-yl)-octahydro-1H-inden-3a-ol | C/C(C)=C/[C@H]1[C@@H](C)CC[C@@H]2[C@@H](C)CC[C@@]12O |
| 702 | RL530 | 1-[(1S,3R)-3-bromo-1,2,2-trimethylcyclopentyl]-4-methylbenzene | C[C@]1(CC[C@@H](Br)[C@]1(C)C)c2ccc(C)cc2 |
| 703 | RL531 | (1R,4aR,6aR,7aS,7bR)-4a-hydroxy-1,7,7,7b-tetramethyl-decahydro-1H-cyclopropa[a]naphthalen-5-one | C[C@@H]2CCC[C@]3(O)C(=O)C[C@@H]1[C@@H]([C@]1(C)C)[C@@]23C |
| 704 | RL532 | (1R,4aR,6aR,7aS,7bR)-1,7,7,7b-tetramethyl-1H,2H,3H,4H,4aH,6aH,7H,7aH,7bH-cyclopropa[a]naphthalen-4-one | C[C@@H]2CCC(=O)[C@@H]3C=C[C@@H]1[C@@H]([C@]1(C)C)[C@@]23C |
| 705 | RL533 | 2,2',5,5',6,6'-hexabromo-1H,1'H-3,3'-biindole | Brc3cc4nc(Br)c(c1c2cc(Br)c(Br)cc2nc1Br)c4cc3Br |
| 706 | RL534 | 3,5-dibromo-1-methyl-1H-indole | Brc1cc2c(cc1)n(C)cc2Br |
| 707 | RL535 | (2S,3R)-2-bromo-8-chloro-1,1,9-trimethyl-5-methylidenespiro[5.5]undec-8-en-3-ol | ClC=1CC2(CCC=1C)C(=C)C[C@@H](O)[C@@H](Br)C2(C)C |
| 708 | RL536 | (2S,3R,6S,9Z)-2-bromo-9-(bromomethylidene)-1,1-dimethyl-5-methylidenespiro[5.5]undec-7-en-3-ol | Br\C=C1\CC[C@]2(C=C1)C(=C)C[C@@H](O)[C@@H](Br)[C@@]2(C)C |
| 709 | RL537 | (2R,3S,6S,9E)-2-bromo-9-(bromomethylidene)-1,1-dimethyl-5-methylidenespiro[5.5]undec-7-en-3-ol | Br\C=C1/CC[C@]2(C=C1)C(=C)C[C@H](O)[C@H](Br)[C@@]2(C)C |
| 710 | RL538 | (2R,3S)-2-bromo-1,1-dimethyl-5,9-dimethylidenespiro[5.5]undec-7-en-3-ol | C=C1CCC2(C=C1)C(=C)C[C@H](O)[C@H](Br)[C@]2(C)C |
| 711 | RL539 | (8R,9S)-8-bromo-9-hydroxy-7,7-dimethyl-11-methylidenespiro[5.5]undec-1-en-3-one | O=C1CCC2(C=C1)C(=C)C[C@H](O)[C@H](Br)[C@]2(C)C |
| 712 | RL540 | 2,9-dibromo-8-chloro-1,1,9-trimethyl-5-methylidenespiro[5.5]undecan-3-ol | BrC1(C)CCC2(CC1Cl)C(=C)CC(O)C(Br)C2(C)C |
| 715 | RL543 | (6S)-4,8-dibromo-9-chloro-1,5,5,9-tetramethylspiro[5.5]undeca-1,3-diene | CC2=CC=C(Br)C(C)(C)[C@@]12CCC(C)(Cl)C(Br)C1 |
| 716 | RL544 | (6R)-4,8-dibromo-9-chloro-1,5,5,9-tetramethylspiro[5.5]undeca-1,3-diene | CC2=CC=C(Br)C(C)(C)[C@]12CCC(C)(Cl)C(Br)C1 |
| 717 | RL545 | (4R,6S)-4,8-dibromo-9-chloro-1,5,5,9-tetramethylspiro[5.5]undec-1-ene | CC2=CC[C@@H](Br)C(C)(C)[C@@]12CCC(C)(Cl)C(Br)C1 |
| 718 | RL546 | (3S,4S,6S)-4,8-dibromo-9-chloro-1,5,5,9-tetramethylspiro[5.5]undec-1-en-3-ol | CC2=C[C@H](O)[C@@H](Br)C(C)(C)[C@@]12CCC(C)(Cl)C(Br)C1 |
| 719 | RL547 | (1S,2R,6R)-4,5'-dibromo-4'-chloro-1,3,3,4'-tetramethyl-7-oxaspiro[bicyclo[4.1.0]heptane-2,1'-cyclohexan]-4-ene | CC1(Cl)CC[C@@]3(CC1Br)C(C)(C)C(Br)=C[C@H]2O[C@]23C |
| 720 | RL548 | (1S,2R,4R,6R)-4,5'-dibromo-4'-chloro-1,3,3,4'-tetramethyl-7-oxaspiro[bicyclo[4.1.0]heptane-2,1'-cyclohexane] | CC1(Cl)CC[C@@]3(CC1Br)C(C)(C)[C@H](Br)C[C@H]2O[C@]23C |
| 721 | RL549 | (1S,3R,5S,6S)-5-bromo-3-(1-bromopropyl)-8-[(2E)-pent-2-en-4-yn-1-yl]-2,7-dioxabicyclo[4.2.1]nonane | BrC(CC)[C@@H]2O[C@H]1C[C@H](OC1C\C=C\C#C)[C@@H](Br)C2 |
| 722 | RL550 | (2R,5S,14R,15R)-2,15-dimethyl-14-(6-methylheptan-2- yl)tetracyclo[8.7.0.0^{2,7}.0^{11,15}]heptadec-7- en-5-ol | CC(C)CCCC(C)[C@H]1CCC2C3C(CC[C@]12C)[C@@]4(C)CC[C@H](O)CC4=C[C@H2]3 |
| 723 | RL551 | (2R,5S,9S,14R,15R)-2,15-dimethyl-14-(6- methylheptan-2- yl)tetracyclo[8.7.0.0^{2,7}.0^{11,15}]heptadec-7- ene-5,9-diol | CC(C)CCCC(C)[C@H]1CCC2C3C(CC[C@]12C)[C@@]4(C)CC[C@H](O)CC4=C[C@H]3O |
| 724 | BD022 | (3S,3aR,7aR)-7a-methyl-3-[(2R)-6-methylhept-5-en-2-yl]-2,3,3a,6,7,7a-hexahydro-1H-indene-3a,4-dicarbaldehyde | C/C(C)=C\CC[C@@H](C)[C@@H]1CC[C@@]2(C)[C@@H2]CC=C(C=O)[C@@]12C=O |
| 725 | BD023 | (3S,3aR,7S,7aR)-7-hydroxy-3-[(2R,5S)-5-hydroxy-6-methylhept-6-en-2-yl]-7a-methyl-2,3,3a,6,7,7a-hexahydro-1H-indene-3a,4-dicarbaldehyde | C=C(C)[C@@H](O)CC[C@@H](C)[C@@H]2CC[C@]1(C)[C@@]2(C=O)C(=CC[C@@H]1O)C=O |
| 726 | BD024 | (3S,3aR,7S,7aR)-7-hydroxy-3-[(2R,5R)-5-hydroxy-6-methylhept-6-en-2-yl]-7a-methyl-2,3,3a,6,7,7a-hexahydro-1H-indene-3a,4-dicarbaldehyde | C=C(C)[C@H](O)CC[C@@H](C)[C@@H]2CC[C@]1(C)[C@@]2(C=O)C(=CC[C@@H]1O)C=O |
| 727 | BD025 | (3S,3aR,7R,7aR)-7-hydroxy-3-[(2R,5S)-5-hydroxy-6-methylhept-6-en-2-yl]-7a-methyl-2,3,3a,6,7,7a-hexahydro-1H-indene-3a,4-dicarbaldehyde | C=C(C)[C@@H](O)CC[C@@H](C)[C@@H]2CC[C@]1(C)[C@@]2(C=O)C(=CC[C@H]1O)C=O |
| 728 | BD026 | (3S,3aR,7R,7aR)-7-hydroxy-3-[(2R,5R)-5-hydroxy-6-methylhept-6-en-2-yl]-7a-methyl-2,3,3a,6,7,7a-hexahydro-1H-indene-3a,4-dicarbaldehyde | C=C(C)[C@H](O)CC[C@@H](C)[C@@H]2CC[C@]1(C)[C@@]2(C=O)C(=CC[C@H]1O)C=O |
| 729 | BD027 | (3S,3aR,7S,7aR)-7-hydroxy-7a-methyl-3-[(2R)-6-methylhept-5-en-2-yl]-2,3,3a,6,7,7a-hexahydro-1H-indene-3a,4-dicarbaldehyde | C/C(C)=C\CC[C@@H](C)[C@@H]2CC[C@]1(C)[C@@]2(C=O)C(=CC[C@@H]1O)C=O |
| 730 | BD028 | (3S,3aR,7R,7aR)-7-hydroxy-7a-methyl-3-[(2R)-6-methylhept-5-en-2-yl]-2,3,3a,6,7,7a-hexahydro-1H-indene-3a,4-dicarbaldehyde | C/C(C)=C\CC[C@@H](C)[C@@H]2CC[C@]1(C)[C@@]2(C=O)C(=CC[C@H]1O)C=O |
| 731 | BD029 | 6-[(3S)-3,5-dimethylhex-4-en-1-yl]-6a-(1-hydroxy-3-oxopropyl)-3,3a,4,5,6,6a-hexahydropentalene-1-carbaldehyde | C/C(C)=C/[C@@H](C)CCC2CCC1CC=C(C=O)C12C(O)CC=O |
| 732 | BD030 | 1-{3-[(3S)-3,5-dimethylhex-4-en-1-yl]-4-formyl-1,2,3,3a,6,6a-Hexahydropentalen-3a-yl}-3-oxopropyl acetate | C/C(C)=C/[C@@H](C)CCC2CCC1CC=C(C=O)C12C(CC=O)OC(C)=O |
| 733 | BT002 | (1R,2R,10R,13R,14R,19R)-1,6,10,14,18,18-Hexamethylpentacyclo[11.8.0.0^{2,10}.0^{4,9}.0^{14,19}]henicosa- 4(9),6-Diene-5,8,17-trione | O=C4C=C(C)C(=O)C=5C[C@H]1[C@@](C)(CC[C@H]2[C@@]3(C)CCC(=O)[C@](C)(C)[C@@H]3CC[C@@]12C)C4=5 |
| 734 | BT003 | (1S,2R,10R,13R,14R,19R)-1,6,10,14,18,18- Hexamethylpentacyclo[11.8.0.0^{2,10}.0^{4,9}.0^{14,19}]henicosa- 4(9),6-Diene-5,8,17-trione | O=C4C=C(C)C(=O)C=5C[C@H]1[C@@](C)(CC[C@H]2[C@@]3(C)CCC(=O)[C@](C)(C)[C@@H]3CC[C@]12C)C4=5 |
| 735 | RC002 | 2-[(4-hydroxyphenyl)methyl]-1H,2H,3H,4H,9H-pyrido[3,4-b]indole-3-carboxylic acid | O=C(O)C2Cc3c4ccccc4nc3CN2Cc1ccc(O)cc1 |
| 738 | BD084 | (5R,6S,10S)-2,10-dimethyl-7-(propan-2-yl)spiro[4.5]dec-1-en-6-ol | CC(C)C2CC[C@H](C)[C@@]1(CCC(C)=C1)[C@H]2O |
| 737 | BD083 | (4R)-3-methyl-5-(6-methylhept-5-en-2-yl)-8-methylidene-1,3a,4,5,6,7,8,8a-octahydroazulen-4-ol | C/C(C)=C\CCC(C)C2CCC(=C)C1CC=C(C)C1[C@@H]2O |
| 736 | BD082 | (4R)-3,8-dimethyl-5-(6-methylhept-5-en-2-yl)-1,3a,4,5,6,8a-hexahydroazulen-4-ol | C/C(C)=C\CCC(C)C2CC=C(C)C1CC=C(C)C1[C@@H]2O |
| 739 | BD031 | (4R)-3-methyl-5-(6-methylhept-5-en-2-yl)-8-methylidene-1,3a,4,5,6,7,8,8a-octahydroazulen-4-ol | C/C(C)=C\CCC(C)C2CCC(=C)C1CC=C(C)C1[C@@H]2O |
| 740 | BD032 | (4R)-3,8-dimethyl-5-(6-methylhept-5-en-2-yl)-1,3a,4,5,6,8a-hexahydroazulen-4-ol | C/C(C)=C\CCC(C)C2CC=C(C)C1CC=C(C)C1[C@@H]2O |
| 741 | BD033 | (1S,4aR,5aS,10aS)-4a-hydroxy-5a,10a-dimethyl-4-methylidene-8-(propan-2-yl)-1H,2H,3H,4H,4aH,5H,5aH,6H,7H,9H,10H,10aH-cyclohexa[f]azulen-1-yl acetate | CC(C)C=1CC[C@@]2(C)C[C@@]3(O)C(=C)CC[C@H](OC(C)=O)[C@]3(C)CCC=12 |
| 742 | BD034 | (5S,9aS)-3,7,9a-trimethyl-12-(propan-2-yl)-1H,2H,5H,6H,9H,9aH,10H,11H-cyclopenta[11]annulen-5-ol | CC(C)C=1CC[C@@]2(C)CC=C(C)C[C@H](O)C=C(C)CCC=12 |
| 743 | BD035 | (4S,6R)-3-methyl-5-(6-methylhept-5-en-2-yl)-8-methylidene-1,3a,4,5,6,7,8,8a-octahydroazulene-4,6-diol | C/C(C)=C\CCC(C)C2[C@H](O)CC(=C)C1CC=C(C)C1[C@@H]2O |
| 744 | BD036 | (1S,4aR,5aS,10aS)-5a,10a-dimethyl-4-methylidene-8-(propan-2-yl)-1H,2H,3H,4H,4aH,5H,5aH,6H,7H,9H,10H,10aH-cyclohexa[f]azulene-1,4a-diol | CC(C)C=1CC[C@@]2(C)C[C@@]3(O)C(=C)CC[C@H](O)[C@]3(C)CCC=12 |
| 745 | BD037 | 7-methyl-10-(6-methylhept-5-en-2-yl)-1H,3H,5H,8H,9H,10H,10aH-cyclonona[c]furan-1-ol | C/C(C)=C\CCC(C)C1CCC(C)=CCC=C2COC(O)C12 |
| 746 | BD038 | (4R,8S)-3,8-dimethyl-5-(6-methylhept-5-en-2-yl)-1,3a,4,5,6,7,8,8a-octahydroazulene-4,8-diol | C/C(C)=C\CCC(C)C2CC[C@](C)(O)C1CC=C(C)C1[C@@H]2O |
| 747 | BD039 | (2R,4S,5aS,6S,9aR,10aS)-4-(acetyloxy)-6,9a-dihydroxy-5a,10a-dimethyl-9-methylidene-3-(propan-2-yl)-1H,2H,4H,5H,5aH,6H,7H,8H,9H,9aH,10H,10aH-cyclohexa[f]azulen-2-yl acetate | CC(=O)O[C@H]1C[C@@]3(C)[C@@H](O)CCC(=C)[C@]3(O)C[C@]2(C)C[C@@H](OC(C)=O)C(=C12)C(C)C |
| 748 | BD047 | (3aR,7S,8R,8aS)-8-hydroxy-1-methyl-7-(6-methylhept-5-en-2-yl)-3,3a,6,7,8,8a-hexahydroazulene-4-carbaldehyde | C/C(C)=C\CCC(C)[C@@H]1CC=C(C=O)[C@@H]2CC=C(C)[C@H]2[C@@H]1O |
| 749 | BD048 | (1R,3E,6E,8R,9R)-7,8-diformyl-3-methyl-9-(6-methylhept-5-en-2-yl)cyclonona-3,6-dien-1-yl acetate | C/C(C)=C\CCC(C)[C@@H]1[C@@H](C=O)C(=CCC=C(C)C[C@H]1OC(C)=O)C=O |
| 750 | BD049 | (1R,2E,5E,9R)-6-methyl-9-(6-methylhept-5-en-2-yl)cyclonona-2,5-diene-1,2-dicarbaldehyde | C/C(C)=C\CCC(C)[C@H]1[C@H2]CC(C)=CCC=C(C=O)[C@@H]1C=O |
| 751 | BD050 | (10R)-7-methyl-10-(6-methylhept-5-en-2-yl)-1H,3H,4H,5H,8H,9H,10H-cyclonona[c]furan-1-one | C/C(C)=C\CCC(C)[C@H]2CCC(C)=CCCC=1COC(=O)C=12 |
| 752 | BD051 | (5S)-7-formyl-5-[(2S)-6-methylhept-5-en-2-yl]-2-methylidenebicyclo[4.3.1]dec-7-en-10-yl acetate | C/C(C)=C\CC[C@H](C)[C@@H]1CC/C(=C)C2CC=C(C=O)C1C2OC(C)=O |
| 753 | BD040 | (2S,6S,7S)-1,3-dimethyl-8-[(2R)-6-methylhept-5-en-2-yl]tricyclo[4.4.0.0^{2,7}]dec-3-ene | C/C(C)=C\CC[C@@H](C)[C@@H]2CC[C@@H](C)c1ccc(C)cc12 |
| 754 | BD041 | (1R)-1,6-dimethyl-4-[(2R)-6-methylhept-5-en-2-yl]-1,2,3,4-tetrahydronaphthalene | C/C(C)=C\CC[C@@H](C)[C@@H]2CCC3(C)[C@@H]1C(C)=CC[C@H]3[C@@H]12 |
| 755 | BD042 | (1S,4S,4aS,5aR,8S,8aS,10aS)-4,5a,10a-trimethyl-8-(prop-1-en-2-yl)-tetradecahydrocyclohexa[f]azulene-1,4-diol | C=C(C)[C@H]1CC[C@]2(C)C[C@@H]3[C@@](C)(O)CC[C@H](O)[C@@]3(C)CC[C@@H]12 |
| 756 | BD043 | (1S,4R,4aS,5aR,8S,8aS,10aS)-4,5a,10a-trimethyl-8-(prop-1-en-2-yl)-tetradecahydrocyclohexa[f]azulene-1,4-diol | C=C(C)[C@H]1CC[C@]2(C)C[C@@H]3[C@](C)(O)CC[C@H](O)[C@@]3(C)CC[C@@H]12 |
| 757 | BD044 | (1S,4aR,5aR,8S,8aS,10aS)-5a,10a-dimethyl-4-methylidene-8-(prop-1-en-2-yl)-tetradecahydrocyclohexa[f]azulen-1-ol | C=C(C)[C@H]1CC[C@]2(C)C[C@@H]3C(=C)CC[C@H](O)[C@@]3(C)CC[C@@H]12 |
| 758 | BD045 | (1S,4aR,5aR,8S,8aS,10aS)-,5a,10a-trimethyl-8-(prop-1-en-2-yl)-1H,2H,4aH,5H,5aH,6H,7H,8H,8aH,9H,10H,10aH-cyclohexa[f]azulen-1-ol | C=C(C)[C@H]1CC[C@]2(C)C[C@@H]3C(C)=CC[C@H](O)[C@@]3(C)CC[C@@H]12 |
| 759 | BD046 | (1S,5aR,8S,8aS,10aS)-4,5a,10a-trimethyl-8-(prop-1-en-2-yl)-1H,2H,3H,5H,5aH,6H,7H,8H,8aH,9H,10H,10aH-cyclohexa[f]azulen-1-ol | C=C(C)[C@H]1CC[C@]2(C)CC3=C(C)CC[C@H](O)[C@@]3(C)CC[C@@H]12 |
| 760 | BB004 | (2E,6E,10E,12S)-3,7,11,15-tetramethylhexadeca-2,6,10,14-tetraene-1,12-diol | C/C(C)=C\C[C@H](O)C(\C)=C\CCC(\C)=C\CCC(\C)=C\CO |
| 761 | GC005 | (3E,5E,7E,9E,11E,13E,15E,17E)-1-[(4R)-4-hydroxy-2,6,6-trimethylcyclohex-1-en-1-yl]-18-[(1R,4R)-4-hydroxy-2,6,6-trimethylcyclohex-2-en-1-yl]-3-(hydroxymethyl)-7,12,16-trimethyloctadeca-3,5,7,9,11,13,15,17-octaen-2-one | C[C@]2(C)C[C@H](O)CC(C)=C2CC(=O)C(=C\C=C\C(\C)=C\C=C\C=C(/C)\C=C\C=C(/C)\C=C\[C@H]1C(C)=C[C@H](O)C[C@]1(C)C)\CO |
| 762 | GC006 | (2E)-4-[(4R)-4-hydroxy-2,6,6-trimethylcyclohex-1-en-1-yl]-2-[(2E,4E,6E,8E,10E,12E,14E)-15-[(1R,4R)-4-  hydroxy-2,6,6-trimethylcyclohex-2-en-1-yl]-4,9,13-trimethylpentadeca-2,4,6,8,10,12,14-heptaen-1-ylidene]-3-oxobutyl (2E)-dodec-2-enoate | C[C@]2(C)C[C@H](O)CC(C)=C2CC(=O)C(=C\C=C\C(\C)=C\C=C\C=C(/C)\C=C\C=C(/C)\C=C\[C@H]1C(C)=C[C@H](O)C[C@]1(C)C)\COC(=O)\C=C\CCCCCCCCC |
| 763 | BU006 | (1S,3R)-3-hydroxy-4-[(3E,5E,7E,9E,11E,13E,15E)-18-[(1S,4S,6R)-4-hydroxy-2,2,6-trimethyl-7-oxabicyclo[4.1.0]heptan-1-yl]-3,7,12,16-tetramethyl-17-oxooctadeca-1,3,5,7,9,11,13,15-octaen-1-ylidene]-3,5,5-trimethylcyclohexyl acetate | CC(=O)O[C@H]3C[C@@](C)(C)\C(=C=CC(\C)=C\C=C\C(\C)=C\C=C\C=C(/C)\C=C\C=C(/C)C(=O)C[C@]12O[C@]2(C)C[C@@H](O)C[C@@]1(C)C)[C@](C)(O)C3 |
| 764 | BC003 | (1S,4'R,4aS,7aS)-3-[(2E)-4-(2-hydroxy-5-methoxy-3-methylphenyl)-2-methylbut-2-en-1-yl]-4a,5',5',7a-tetramethyl-5,6,7,7a-tetrahydro-4aH-spiro[cyclopenta[c]pyran-1,2'-oxolane]-4'-ol | COc4cc(C\C=C(/C)CC3=C[C@]1(C)CCC[C@]1(C)[C@@]2(C[C@@H](O)[C@@](C)(C)O2)O3)c(O)c(C)c4 |
| 765 | BC004 | (1R,4'S,4aR,7aR)-3-[(2E)-4-(5-hydroxy-2-methoxy-3-methylphenyl)-2-methylbut-2-en-1-yl]-4a,5',5',7a-tetramethyl-5,6,7,7a-tetrahydro-4aH-spiro[cyclopenta[c]pyran-1,2'-oxolane]-4'-ol | Oc4cc(C\C=C(/C)CC3=C[C@@]1(C)CCC[C@@]1(C)[C@]2(C[C@H](O)C(C)(C)O2)O3)c(OC)c(C)c4 |
| 766 | BC005 | 2-[(2E)-3-[(1R,4'S,4aR,7aR)-4'-hydroxy-4a,5',5',7a-tetramethyl-5,6,7,7a-tetrahydro-4aH-spiro[cyclopenta[c]pyran-1,2'-oxolane]-3-ylmethyl]but-2-en-1-yl]-6-methylbenzene-1,4-diol | Oc4cc(C\C=C(/C)CC3=C[C@@]1(C)CCC[C@@]1(C)[C@]2(C[C@H](O)C(C)(C)O2)O3)c(O)c(C)c4 |
| 767 | BC006 | (1S,4'R,4aS,7aS)-3-[(2E)-4-(2,5-dimethoxy-3-methylphenyl)-2-methylbut-2-en-1-yl]-4a,5',5',7a-tetramethyl-5,6,7,7a-tetrahydro-4aH-spiro[cyclopenta[c]pyran-1,2'-oxolane]-4'-ol | COc4cc(C\C=C(/C)CC3=C[C@]1(C)CCC[C@]1(C)[C@@]2(C[C@@H](O)[C@@](C)(C)O2)O3)c(OC)c(C)c4 |
| 768 | BC007 | (1R,4'S,4aR,7aR)-3-[(2E)-4-[2,5-bis(acetyloxy)-3-methylphenyl]-2-methylbut-2-en-1-yl]-4a,5',5',7a-tetramethyl-5,6,7,7a-tetrahydro-4aH-spiro[cyclopenta[c]pyran-1,2'-oxolane]-4'-yl acetate | CC(=O)Oc4cc(C\C=C(/C)CC3=C[C@@]1(C)CCC[C@@]1(C)[C@]2(C[C@H](OC(C)=O)C(C)(C)O2)O3)c(OC(C)=O)c(C)c4 |
| 769 | BC008 | 2-[(2E)-3-[(1S,4'R,4aR,7aR)-4'-methoxy-4a,5',5',7a-tetramethyl-5,6,7,7a-tetrahydro-4aH-spiro[cyclopenta[c]pyran-1,2'-oxolane]-3-ylmethyl]but-2-en-1-yl]-6-methylcyclohexa-2,5-diene-1,4-dione | O=C4C=C(C\C=C(/C)CC3=C[C@@]1(C)CCC[C@@]1(C)[C@@]2(C[C@@H](OC)C(C)(C)O2)O3)C(=O)C(C)=C4 |
| 770 | BC009 | (1R,4aS,7aS)-3-[(2E)-4-(2,5-dimethoxy-3-  methylphenyl)-2-methylbut-2-en-1-yl]-4a,5',5',7a-tetramethyl-5,6,7,7a-tetrahydro-4aH,5'H-spiro[cyclopenta[c]pyran-1,2'-furan] | COc4cc(C\C=C(/C)CC3=C[C@]1(C)CCC[C@]1(C)[C@@]2(C=C[C@@](C)(C)O2)O3)c(OC)c(C)c4 |
| 771 | BC010 | 2-[(2E)-3-[(1S,4'R,4aS,7aS)-4'-methoxy-4a,5',5',7a-tetramethyl-5,6,7,7a-tetrahydro-4aH-spiro[cyclopenta[c]pyran-1,2'-oxolane]-3-ylmethyl]but-2-en-1-yl]-4-methoxy-6-methylphenol | COc4cc(C\C=C(/C)CC3=C[C@]1(C)CCC[C@]1(C)[C@@]2(C[C@@H](OC)[C@@](C)(C)O2)O3)c(O)c(C)c4 |
| 772 | BC011 | 2-[(2E)-3-[(1S,4'R,4aS,7aS)-4'-methoxy-4a,5',5',7a-tetramethyl-5,6,7,7a-tetrahydro-4aH-spiro[cyclopenta[c]pyran-1,2'-oxolane]-3-ylmethyl]but-2-en-1-yl]-4-methoxy-6-methylphenol | COc1cc2CCC(C)(Oc2c(C)c1)CC5=C[C@]3(C)CCC[C@]3(C)[C@@]4(C[C@@H](OC)C(C)(C)O4)O5 |
| 773 | BC012 | (1S,4'R,4aS,7aS)-4'-methoxy-4a,5',5',7a-tetramethyl-3-[(2E)-2-methyl-4-(2,3,5-trimethylphenyl)but-2-en-1-yl]-5,6,7,7a-tetrahydro-4aH-spiro[cyclopenta[c]pyran-1,2'-oxolane] | COc4cc(C\C=C(/C)CC3=C[C@]1(C)CCC[C@]1(C)[C@@]2(C[C@@H](OC)[C@@](C)(C)O2)O3)c(OC)c(C)c4 |
| 774 | BC013 | (1R,4aS,7aS)-3-[(6-methoxy-2,8-dimethyl-3,4-dihydro-2H-1-benzopyran-2-yl)methyl]-4a,5',5',7a-tetramethyl-5,6,7,7a-tetrahydro-4aH,5'H-spiro[cyclopenta[c]pyran-1,2'-furan] | COc1cc2CCC(C)(Oc2c(C)c1)CC5=C[C@]3(C)CCC[C@]3(C)[C@@]4(C=CC(C)(C)O4)O5 |
| 775 | BC014 | (1S,4'R,4aS,7aS)-3-[(6-methoxy-2,8-dimethyl-3,4-dihydro-2H-1-benzopyran-2-yl)methyl]-4a,5',5',7a-tetramethyl-5,6,7,7a-tetrahydro-4aH-spiro[cyclopenta[c]pyran-1,2'-oxolane]-4'-ol | COc1cc2CCC(C)(Oc2c(C)c1)CC5=C[C@]3(C)CCC[C@]3(C)[C@@]4(C[C@@H](O)C(C)(C)O4)O5 |
| 776 | BC015 | 2-[(2E)-3-[(1R,4aS,7aS)-4a,5',5',7a-tetramethyl-5,6,7,7a-tetrahydro-4aH,5'H-  spiro[cyclopenta[c]pyran-1,2'-furan]-3-ylmethyl]but-2-en-1-yl]-4-methoxy-6-methylphenol | COc4cc(C\C=C(/C)CC3=C[C@]1(C)CCC[C@]1(C)[C@@]2(C=C[C@@](C)(C)O2)O3)c(O)c(C)c4 |
| 777 | BC016 | (3aR,7aR)-6-[(2E)-4-(2,5-dimethoxy-3-methylphenyl)-2-methylbut-2-en-1-yl]-5-(2-hydroxy-2-methylpropyl)-3a,7a-dimethyl-2,3,3a,4,7,7a-hexahydro-1H inden-4-one | COc3cc(C\C=C(/C)CC=1C[C@@]2(C)CCC[C@@]2(C)C(=O)C=1CC(C)(C)O)c(OC)c(C)c3 |
| 778 | BD052 | (1S,4aR,5aS,10aS)-4a-hydroxy-5a,10a-dimethyl-4-methylidene-8-(propan-2-yl)-  1H,2H,3H,4H,4aH,5H,5aH,6H,10H,10aH-cyclohexa[f]azulen-1-yl acetate | CC(C)C1=CC[C@@]2(C)C[C@@]3(O)C(=C)CC[C@H](OC(C)=O)[C@]3(C)CC=C12 |
| 779 | BD053 | (4S,5aS,6S,9aR,10aS)-6,9a-dihydroxy-5a,10a-dimethyl-9-methylidene-3-(propan-2-yl)-1H,2H,4H,5H,5aH,6H,7H,8H,9H,9aH,10H,10aH-cyclohexa[f]azulen-4-yl acetate | CC(=O)O[C@H]1C[C@@]3(C)[C@@H](O)CCC(=C)[C@]3(O)C[C@]2(C)CCC(=C12)C(C)C |
| 780 | BB001 | (2E,10E,12R)-3,11,15-trimethyl-7-methylidenehexadeca-2,10,14-triene-1,6,12-triol | C/C(C)=C\C[C@@H](O)C(\C)=C\CCC(=C)C(O)CCC(\C)=C\CO |
| 781 | BB002 | (2E,10E,12R)-3,7,11,15-tetramethylhexadeca-2,10,14-triene-1,7,12-triol | C/C(CCCC(C)(O)CC\C=C(/C)[C@H](O)C\C=C(/C)C)=C\CO |
| 782 | BB003 | (2E,6E,10E,12R)-3,7,11,15-tetramethylhexadeca-2,6,10,14-tetraene-1,12-diol | C/C(C)=C\C[C@@H](O)C(\C)=C\CCC(\C)=C\CCC(\C)=C\CO |
| 783 | RJ008 | (2R,7S,13S,14R,15S)-13-hydroxy-2,15-dimethyl-14- [(2R)-6-methylheptan-2- yl]tetracyclo[8.7.0.0^{2,7}.0^{11,15}]heptadecane- 5,8-dione | CC(C)CCC[C@@H](C)[C@H]2[C@@H](O)CC1C3CC(=O)[C@H]4CC(=O)CC[C@]4(C)C3CC[C@@]12C |
| 784 | RP012 | (1Z,3E,5S,6R,7E)-8-bromo-1,5,6-trichloro-2-(dichloromethyl)-6-methylocta-1,3,7-triene | Cl\C=C(\C=C\[C@H](Cl)[C@](C)(Cl)C=[C@H]Br)C(Cl)Cl |
| 785 | RP013 | (1Z,3E,5R,6R,7E)-8-bromo-1,5,6-trichloro-2-(dichloromethyl)-6-methylocta-1,3,7-triene | Cl\C=C(\C=C\[C@@H](Cl)[C@](C)(Cl)C=[C@H]Br)C(Cl)Cl |
| 786 | RP014 | (1Z,3E,5S,6R,7Z)-8-bromo-1,5,6-trichloro-2-(dichloromethyl)-6-methylocta-1,3,7-triene | Cl\C=C(\C=C\[C@H](Cl)[C@](C)(Cl)C=[C@H]Br)C(Cl)Cl |
| 787 | RP015 | (1Z,3E,5S,6R)-1,5,6-trichloro-2-(dichloromethyl)-6-methylocta-1,3,7-triene | Cl\C=C(\C=C\[C@H](Cl)[C@](C)(Cl)C=C)C(Cl)Cl |
| 788 | RP016 | (3R,4S)-3,4,6,7-tetrachloro-3,7-dimethyloct-1-ene | C=C[C@@](C)(Cl)[C@@H](Cl)CC(Cl)C(C)(C)Cl |
| 789 | BS049 | 2-methyl-6-[(2E,6E,10E)-3,7,11,15-tetramethylhexadeca-2,6,10,14-tetraen-1-yl]cyclohexa-2,5-diene-1,4-dione | O=C1C(C)=CC(=O)C=C1C\C=C(/C)CC\C=C(/C)CC\C=C(/C)CC\C=C(/C)C |
| 790 | BS050 | (2Z,6E,10E)-6,10-dimethyl-12-(5-methyl-3,6-dioxocyclohexa-1,4-dien-1-yl)-2-(4-methylpent-3-en-1-yl)dodeca-2,6,10-trienoic acid | O=C1C(C)=CC(=O)C=C1C\C=C(/C)CC\C=C(/C)CC\C=C(\CC\C=C(/C)C)C(=O)O |
| 791 | BS046 | (2Z,6E,10E)-12-(2,5-dihydroxy-3-methylphenyl)-6,10-dimethyl-2-(4-methylpent-3-en-1-yl)dodeca-2,6,10-trienoic acid | Oc1c(C)cc(O)cc1C\C=C(/C)CC\C=C(/C)CC\C=C(\CC\C=C(/C)C)C(=O)O |
| 792 | BS047 | (6E,10E,14E)-16-(2,5-dihydroxy-3-methylphenyl)-2-hydroxy-2,6,10,14-tetramethylhexadeca-6,10,14-trien-3-one | Oc1c(C)cc(O)cc1C\C=C(/C)CC\C=C(/C)CC\C=C(/C)CCC(=O)C(C)(C)O |
| 793 | BS048 | 2-[(2E,6E,10E,14R)-14,15-dihydroxy-3,7,11,15-tetramethylhexadeca-2,6,10-trien-1-yl]-6-methylbenzene-1,4-diol | Oc1c(C)cc(O)cc1C\C=C(/C)CC\C=C(/C)CC\C=C(/C)CC[C@@H](O)C(C)(C)O |
| 794 | BS040 | 2-[(2E,6E,10E,14R)-14,15-dihydroxy-3,7,11,15-tetramethylhexadeca-2,6,10-trien-1-yl]-6-methylcyclohexa-2,5-diene-1,4-dione | O=C1C(C)=CC(=O)C=C1C\C=C(/C)CC\C=C(/C)CC\C=C(/C)CC[C@@H](O)C(C)(C)O |
| 795 | BS041 | (3R,6E,10E)-13-(6-hydroxy-2,8-dimethyl-2H-chromen-2-yl)-2,6,10-trimethyltrideca-6,10-diene-2,3-diol | CC(C)(O)[C@H](O)CCC(\C)=C\CCC(\C)=C\CCC1(C)C=Cc2cc(O)cc(C)C2o1 |
| 796 | BS042 | 2-[(2E,6E,10E)-12-hydroxy-11-(hydroxymethyl)-3,7,15-trimethylhexadeca-2,6,10,14-tetraen-1-yl]-6-methylbenzene-1,4-diol | Oc1c(C)cc(O)cc1C\C=C(/C)CC\C=C(/C)CC\C=C(/CO)C(O)C\C=C(/C)C |
| 797 | BS043 | 2-[(2E,6E,10E)-12-hydroxy-11-(hydroxymethyl)-3,7,15-trimethylhexadeca-2,6,10,14-tetraen-1-yl]-6-methylcyclohexa-2,5-diene-1,4-dione | O=C1C(C)=CC(=O)C=C1C\C=C(/C)CC\C=C(/C)CC\C=C(/CO)C(O)C\C=C(/C)C |
| 798 | BS044 | (2Z,6E)-2-(3-chloro-4-methylpent-4-en-1-yl)-9-(6-hydroxy-2,8-dimethyl-2H-chromen-2-yl)-6-methylnona-2,6-dienoic acid | C=C(C)C(Cl)CC\C(=C\CCC(\C)=C\CCC1(C)C=Cc2cc(O)cc(C)c2O1)C(=O)O |
| 799 | BS045 | (2Z,6E)-9-(6-hydroxy-2,8-dimethyl-2H-chromen-2-yl)-6-methyl-2-(4-methylpent-3-en-1-yl)nona-2,6-dienoic acid | O=C(O)C(/CC\C=C(/C)C)=C\CCC(\C)=C\CCC1(C)C=Cc2cc(O)cc(C)C2o1 |
| 800 | BB005 | (2E,6E,10E,12S)-12-hydroxy-3,7,11,15-tetramethylhexadeca-2,6,10,14-tetraenoic acid | C/C(C)=C\C[C@H](O)C(\C)=C\CCC(\C)=C\CCC(\C)=C\C(=O)O |
| 801 | BB006 | methyl (2E,6E,10E,12S)-12-hydroxy-3,7,11,15-tetramethylhexadeca-2,6,10,14-tetraenoate | O=C(/C=C(\C)CC\C=C(/C)CC\C=C(/C)[C@@H](O)C\C=C(/C)C)OC |
| 802 | BB007 | (2E,6E,9E,11E)-3,7,11,15-tetramethylhexadeca-2,6,9,11,14-pentaen-1-ol | C/C(C)=C\C\C=C(/C)\C=C\CC(\C)=C\CCC(\C)=C\CO |
| 803 | BB008 | (2E,6E,10E,12S)-12-hydroxy-3,7,11,15-tetramethylhexadeca-2,6,10,14-tetraenal | C/C(C)=C\C[C@H](O)C(\C)=C\CCC(\C)=C\CCC(\C)=C\C=O |
| 804 | BB009 | (2Z,6E,10E,12S)-12-hydroxy-3,7,11,15-tetramethylhexadeca-2,6,10,14-tetraenal | C/C(C)=C\C[C@H](O)C(\C)=C\CCC(\C)=C\CCC(/C)=C\C=O |
| 805 | BB010 | (2E,6E,10E,13E)-3,7,11,15-tetramethylhexadeca-2,6,10,13,15-pentaen-1-ol | C=C(C)/C=C/CC(\C)=C\CCC(\C)=C\CCC(\C)=C\CO |
| 806 | BB011 | (2E,6E,10E)-3,7,11,15-tetramethyl-13-oxohexadeca-2,6,10,14-tetraenal | C/C(C)=C/C(=O)CC(\C)=C\CCC(\C)=C\CCC(\C)=C\C=O |
| 807 | BB012 | (2Z,6E,10E)-3,7,11,15-tetramethyl-13-oxohexadeca-2,6,10,14-tetraenal | C/C(C)=C/C(=O)CC(\C)=C\CCC(\C)=C\CCC(/C)=C\C=O |
| 808 | BD063 | (3aS,4S,5R,6R,8aR)-3-methyl-5-(6-methylhept-5-en-2-yl)-8-methylidene-1,3a,4,5,6,7,8,8a-octahydroazulene-4,6-diol | C/C(C)=C\CCC(C)[C@@H]2[C@H](O)[C@H2]C(=C)\[C@@H]1CC=C(C)[C@H]1[C@@H]2O |
| 809 | BZ001 | (5Z,8Z,11Z,14Z,17Z)-1-(2,4,6-trihydroxyphenyl)icosa-5,8,11,14,17-pentaen-1-one | O=C(CCC/C=C\C/C=C\C/C=C\C/C=C\C/C=C\CC)c1c(O)cc(O)cc1O |
| 810 | BZ002 | (5Z,8Z,11Z,13E,15S,17Z)-15-hydroxy-1-(2,4,6-trihydroxyphenyl)icosa-5,8,11,13,17-pentaen-1-one | O=C(CCC/C=C\C/C=C\C/C=C\C=C\[C@@H](O)C/C=C\CC)c1c(O)cc(O)cc1O |
| 811 | BZ003 | 15-hydroxy-1-(2,4,6-trihydroxyphenyl)icosan-1-one | O=C(CCCCCCCCCCCCCC(O)CCCCC)c1c(O)cc(O)cc1O |
| 812 | BZ004 | 5-[(1S,3Z)-1-hydroxyhex-3-en-1-yl]-8-[(2Z,5Z)-10-oxo-10-(2,4,6-trihydroxyphenyl)deca-2,5-dien-1-yl]-2-phenyl-1H,2H,3H,5H,8H-[1,2,4]triazolo[1,2-a]pyridazine-1,3-dione | Oc1cc(O)cc(O)c1C(=O)CCC\C=C/C\C=C/CC2C=CC([C@@H](O)C/C=C\CC)N3C(=O)N(C(=O)N23)c4ccccc4 |
| 814 | BC017 | (2R,3'aS,5'R,7'aS)-5'-[(2E)-4-(5-hydroxy-2-methoxy-3-methylphenyl)but-2-en-2-yl]-3'a,5,5,7'a-tetramethyl-1',2',3',3'a,5',6',7',7'a-octahydro-5H-spiro[furan-2,4'-indene]-6'-one | Oc4cc(C\C=C(/C)[C@H]3C(=O)[C@@H2][C@]1(C)CCC[C@]1(C)[C@]23C=C[C@](C)(C)O2)c(OC)c(C)c4 |
| 815 | BD066 | 2-{[(1S,4aS,8aR)-2,5,5,8a-tetramethyl-decahydronaphthalen-1-yl]methyl}benzene-1,4-diol | Oc3cc(C[C@H]1C(=C)CC[C@H]2[C@@](C)(C)CCC[C@]12C)c(O)cc3 |
| 816 | BD067 | 2-{[(1R,4aR,8aS)-2,5,5,8a-tetramethyl-decahydronaphthalen-1-yl]methyl}benzene-1,4-diol | Oc3cc(C[C@H]1C(C)=CC[C@H]2[C@@](C)(C)CCC[C@]12C)c(O)cc3 |
| 817 | BD068 | (5aR,7aS,11aS,11bR)-5a,8,8,11a-tetramethyl-6,7,7a,8,9,10,11,11a,11b,12-decahydro-5aH-5-oxatetraphen-2-ol | Oc1cc2C[C@@H]3[C@@]4(C)CCC[C@](C)(C)[C@@H]4CC[C@@]3(C)Oc2cc1 |
| 818 | BD069 | 2-{[(1S,4aS,8aR)-2,5,5,8a-tetramethyl-decahydronaphthalen-1-yl]methyl}cyclohexa-2,5-diene-1,4-dione | O=C3C=C(C[C@H]1C(=C)CC[C@H]2[C@@](C)(C)CCC[C@]12C)C(=O)C=C3 |
| 819 | BD070 | 2-{[(1R,4aR,8aS)-2,5,5,8a-tetramethyl-decahydronaphthalen-1-yl]methyl}cyclohexa-2,5-diene-1,4-dione | O=C3C=C(C[C@@H]1C(=C)CC[C@@H]2[C@](C)(C)CCC[C@@]12C)C(=O)C=C3 |
| 820 | BD071 | (5bS,9aS)-5b,9,9-trimethyl-1,4,5b,6,7,8,9,9a,10,11-decahydrotetraphene-1,4-dione | O=C3C=CC(=O)c4cc2c(CC[C@@H]1[C@]2(C)CCC[C@]1(C)C)cc34 |
| 821 | GC001 | (4aS,9aS)-6-bromo-1,1,4a-trimethyl-2,3,4,4a,9,9a-hexahydro-1H-xanthen-7-ol | Oc1cc2C[C@H]3[C@@](C)(C)CCC[C@]3(C)Oc2cc1Br |
| 822 | GC002 | 2-bromo-5-[(2E)-3,7-dimethylocta-2,6-dien-1-yl]benzene-1,4-diol | Oc1cc(C\C=C(/C)CC\C=C(/C)C)c(O)cc1Br |
| 823 | GC003 | (2S,4aR,9aS)-2,6-dibromo-1,1,4a-trimethyl-2,3,4,4a,9,9a-hexahydro-1H-xanthen-7-ol | Oc1cc2C[C@H]3[C@](C)(C)[C@@H](Br)CC[C@@]3(C)Oc2cc1Br |
| 824 | GC004 | (2S,4aS,9aS)-2,6-dibromo-1,1,4a-trimethyl-2,3,4,4a,9,9a-hexahydro-1H-xanthen-7-ol | Oc1cc2C[C@H]3[C@](C)(C)[C@@H](Br)CC[C@]3(C)Oc2cc1Br |
| 690 | BD056 | (1E,2R,4R,8S,11E,15S)-11-methyl-7-(4-methylpent-3- en-1-yl)-3,5- dioxatricyclo[6.6.1.0^{4,15}]pentadeca-1(14),11- dien-2-ol | C/C(C)=C\CC[C@H]3CO[C@@H]2O[C@@H](O)C1=CCC=C(C)CC[C@@H]3[C@@H]12 |
| 691 | BD057 | (2R)-2-[(1R,3E,6E)-2-(2-hydroxyethyl)-3-(hydroxymethyl)-7-methylcyclonona-3,6-dien-1-yl]-6-methylhept-5-en-1-yl acetate | C/C(C)=C\CC[C@@H](COC(C)=O)[C@@H]1CCC(C)=CCC=C(CO)C1CCO |
| 692 | BD058 | (1E,2R,4R,8S,11E,15S)-2-methoxy-11-methyl-7-(4- methylpentyl)-3,5- dioxatricyclo[6.6.1.0^{4,15}]pentadeca-1(14),11- diene | C/C(C)=C\CC[C@H]3CO[C@@H]2OC(OC)C1=CCC=C(C)CC[C@@H]3[C@@H]12 |
| 828 | BD075 | (2R,9S,10Z)-14-formyl-2,10-dimethyl-5-(propan-2- yl)tricyclo[9.4.0.0^{2,6}]pentadeca-4,10,14-trien-9- yl acetate | CC(C)C2=CC[C@@]3(C)C1C=C(CCC1=C(C)[C@H](CCC23)OC(C)=O)C=O |
| 829 | BD076 | (2R,9S,10Z)-9-(acetyloxy)-2,10-dimethyl-5-(propan-2- yl)tricyclo[9.4.0.0^{2,6}]pentadeca-4,10,14-triene- 14-carboxylic acid | CC(C)C2=CC[C@@]3(C)C1C=C(CCC1=C(C)[C@H](CCC23)OC(C)=O)C(=O)O |
| 830 | BD077 | (1S,3aS)-3a,6,10-trimethyl-11-methylidene-1-(propan-2-yl)-1H,2H,3H,3aH,4H,7H,8H,11H,12H,12aH-cyclopenta[11]annulen-1-ol | CC(C)[C@@]2(O)CC[C@@]1(C)CC=C(C)[C@H2]CC=C(C)C(=O)CC12 |
| 831 | BD078 | (9aS,12S)-12-hydroxy-3,7,9a-trimethyl-12-(propan-2-yl)-1H,2H,5H,6H,9H,9aH,10H,11H,12H,12aH-cyclopenta[11]annulen-2-one | CC(C)[C@@]2(O)CC[C@@]1(C)CC=C(C)[C@H2]CC=C(C)C(=O)CC12 |
| 832 | BD079 | (9aS,12S)-12-hydroxy-3,7,9a-trimethyl-12-(propan-2-yl)-1H,2H,5H,6H,9H,9aH,10H,11H,12H,12aH-cyclopenta[11]annulen-2-yl acetate | CC(C)[C@@]2(O)CC[C@@]1(C)CC=C(C)CCC=C(C)C(CC12)OC(C)=O |
| 833 | BD080 | (3S,5aS,9aR,10aS)-3-hydroxy-5a,9,10a-trimethyl-3-(propan-2-yl)-1H,2H,3H,3aH,4H,5H,5aH,9aH,10H,10aH-cyclohexa[f]azulen-5-one | CC(C)[C@@]2(O)CC[C@@]1(C)C[C@@H]3C(C)=CC=C[C@]3(C)C(=O)CC12 |
| 834 | BD081 | (4R,10S,12aS)-10-hydroxy-3,7,12a-trimethyl-8-oxo-10-(propan-2-yl)-1H,4H,5H,8H,9H,9aH,10H,11H,12H,12aH-cyclopenta[11]annulen-4-yl acetate | CC(C)[C@@]1(O)CC[C@@]2(C)CC=C(C)[C@@H](CC=C(C)C(=O)CC12)OC(C)=O |
| 835 | BD062 | (4R,10S,12aS)-10-hydroxy-3,7,12a-trimethyl-8-oxo-10-(propan-2-yl)-1H,4H,5H,8H,9H,9aH,10H,11H,12H,12aH-cyclopenta[11]annulen-4-yl acetate | CC(C)[C@@]1(O)CC[C@@]2(C)CC=C(C)[C@@H](CC=C(C)C(=O)CC12)OC(C)=O |
| 836 | BD065 | (10aS)-9-formyl-10a-methyl-3-(propan-2-yl)-1H,3aH,4H,5H,7H,8H,10H,10aH-cyclohexa[f]azulen-5-yl acetate | CC(C)C2=CC[C@@]3(C)CC1=C(CCC=C1C(CC23)OC(C)=O)C=O |
| 837 | BD072 | (3S,9aR,10aS)-3-hydroxy-9,10a-dimethyl-3-(propan-2-yl)-1H,2H,3H,3aH,4H,5H,5aH,9aH,10H,10aH-cyclohexa[f]azulen-5-one | CC(C)[C@@]3(O)CC[C@@]2(C)C[C@@H]1C(C=CC=C1C)C(=O)CC23 |
| 825 | BD073 | (3S,5aR,6S,9R,9aR,10aS)-3,6,9-trihydroxy-5a,9,10a-trimethyl-3-(propan-2-yl)-1H,2H,3H,3aH,4H,5H,5aH,6H,9H,9aH,10H,10aH-cyclohexa[f]azulen-5-one | CC(C)[C@@]2(O)CC[C@@]1(C)C[C@H]3[C@](C)(O)C=C[C@H](O)[C@]3(C)C(=O)CC12 |
| 826 | BD074 | (6R,9aS,12S)-6,12-dihydroxy-3,7,9a-trimethyl-12-(propan-2-yl)-1H,2H,5H,6H,9H,9aH,10H,11H,12H,12aH-cyclopenta[11]annulen-2-one | CC(C)[C@@]1(O)CC[C@@]2(C)CC=C(C)[C@H](O)CC=C(C)C(=O)CC12 |
| 827 | BD064 | (6R,9aS,12S)-6,12-dihydroxy-3,7,9a-trimethyl-12-(propan-2-yl)-1H,2H,5H,6H,9H,9aH,10H,11H,12H,12aH-cyclopenta[11]annulen-2-one | CC(C)[C@@]1(O)CC[C@@]2(C)CC=C(C)[C@H](O)CC=C(C)C(=O)CC12 |
| 540 | BD054 | (4S,5aS,6S,9aR,10aS)-6-(acetyloxy)-9a-hydroxy-5a,10a-dimethyl-9-methylidene-3-(propan-2-yl)-1H,2H,4H,5H,5aH,6H,7H,8H,9H,9aH,10H,10aH-cyclohexa[f]azulen-4-yl acetate | CC(=O)O[C@H]1C[C@@]3(C)[C@@H](OC(C)=O)CCC(=C)[C@]3(O)C[C@]2(C)CCC(=C12)C(C)C |
| 713 | BD055 | (4S,5aR,9aR,10aS)-9a-hydroxy-5a,10a-dimethyl-9-methylidene-3-(propan-2-yl)-  1H,2H,4H,5H,5aH,6H,7H,8H,9H,9aH,10H,10aH-cyclohexa[f]azulen-4-yl acetate | CC(C)C=1CC[C@@]2(C)C[C@@]3(O)C(=C)CCC[C@]3(C)C[C@H](OC(C)=O)C=12 |
| 714 | RP019 | (1Z,3E,5S,6R,7E)-1,8-dibromo-5,6-dichloro-2,6-dimethylocta-1,3,7-triene | Br\C=C\[C@@](C)(Cl)[C@@H](Cl)/C=C/C(/C)=C\Br |
| 684 | RP020 | (1Z,3E,5R,6R,7E)-1,8-dibromo-5,6-dichloro-2,6-dimethylocta-1,3,7-triene | Br\C=C\[C@@](C)(Cl)[C@H](Cl)/C=C/C(/C)=C\Br |
| 685 | RP021 | (1Z,3E,5R,6R)-1,5,6-trichloro-2-(dichloromethyl)-6-methylocta-1,3,7-triene | Cl\C=C(\C=C\[C@@H](Cl)[C@](C)(Cl)C=C)C(Cl)Cl |
| 686 | RP022 | (2Z,3E,5R,6R)-5,6-dichloro-2-(chloromethylidene)-6-methylocta-3,7-dienal | Cl[C@H](/C=C/C(=C/Cl)C=O)[C@](C)(Cl)C=C |
| 687 | RP023 | (1E,3E,5R,6R)-1-bromo-5,6-dichloro-2,6-dimethylocta-1,3,7-triene | C=C[C@@](C)(Cl)[C@H](Cl)/C=CC(\C)=C\Br |
| 688 | RP024 | (1E,3E,5R,6R)-1-bromo-5,6-dichloro-2,6-dimethylocta-1,3,7-triene | C=C[C@@](C)(Cl)[C@H](Cl)/C=CC(\C)=C\Br |
| 689 | RP025 | (3E,5R,6R)-5,6-dichloro-2-(dichloromethyl)-6-methylocta-1,3,7-triene | C=C(/C=C/[C@@H](Cl)[C@](C)(Cl)C=C)C(Cl)Cl |
| 693 | BD059 | (1S,2E,5E,9S)-6-methyl-9-[(2R)-6-methylhept-5-en-2-yl]cyclonona-2,5-diene-1,2-dicarbaldehyde | C/C(C)=C\CC[C@@H](C)[C@@H]1CCC(C)=CCC=C(C=O)[C@H]1C=O |
| 694 | BD060 | (3aS,4R,5S,8S,8aR)-3,8-dimethyl-5-(6-methylhept-5-en-2-yl)-1,3a,4,5,6,7,8,8a-octahydroazulene-4,8-diol | C/C(C)=C\CCC(C)[C@@H]1CC[C@](C)(O)[C@@H]2CC=C(C)[C@H]2[C@@H]1O |
| 813 | BD061 | 2-[(2R,5S)-5-[(3aS,4S,5R,8aR)-4-hydroxy-3-methyl-8-methylidene-1,3a,4,5,6,7,8,8a-octahydroazulen-5-yl]-5-methyloxolan-2-yl]propan-2-yl acetate | O=C(C)OC(C)(C)[C@H]1CC[C@](C)(O1)[C@@H]2CCC(=C)[C@@H]3CC=C(C)[C@H]3[C@@H]2O |
| 839 | RR002 | (3E)-4-(2,3-Dibromo-4,5-dihydroxyphenyl)but-3-en-2-one | Brc1c(/C=C/C(C)=O)cc(O)c(O)c1Br |
| 840 | RR003 | 2-(3-bromo-5-hydroxy-4-methoxyphenyl)-3-(2,3- dibromo-4,5-dihydroxyphenyl)propanoic acid | Brc1c(cc(O)c(O)c1Br)CC(c2cc(O)c(OC)c(Br)c2)C(=O)O |
| 841 | RR004 | Methyl 2-(3-bromo-5-hydroxy-4-methoxyphenyl)-3-(2,3-dibromo-4,5-dihydroxyphenyl)propanoate | Brc2c(CC(c1cc(O)c(OC)c(Br)c1)C(=O)OC)cc(O)c(O)c2Br |
| 842 | RR005 | 3-(2,3-Dibromo-4,5-dihydroxyphenyl)-2-phenylpropanoic acid | Brc1c(cc(O)c(O)c1Br)CC(c2ccccc2)C(=O)O |
| 843 | RR006 | (3-Bromo-5-hydroxy-4-methoxyphenyl)(2,3-dibromo-4,5-dihydroxyphenyl)acetic acid | Brc1c(cc(O)c(O)c1Br)C(c2cc(O)c(OC)c(Br)c2)C(=O)O |
| 844 | RR007 | (3-Bromo-5-hydroxy-4-methoxyphenyl)acetic acid | Brc1cc(cc(O)c1OC)CC(=O)O |
| 845 | RR008 | Methyl (3-bromo-5-hydroxy-4-methoxyphenyl)acetate | COc1c(O)cc(cc1Br)CC(=O)OC |
| 846 | RR009 | 3-bromo-5-hydroxy-4-methoxycyclohexane-1-carboxylic acid | COC1C(O)CC(CC1Br)C(O)=O |
| 847 | RR010 | 3,4-dibromo-5-(2-hydroxyethyl)benzene-1,2-diol | Brc1c(CCO)cc(O)c(O)c1Br |
| 848 | RR011 | 2-(2,3-dibromo-4,5-dihydroxyphenyl)ethyl hydrogen sulfate | Brc1c(CCOS(=O)(=O)O)cc(O)c(O)c1Br |
| 849 | RR012 | [2-(3-bromo-4,5-dihydroxyphenyl)ethoxy]sulfonic acid | Oc1cc(CCOS(=O)(=O)O)cc(Br)c1O |
| 850 | RR013 | (2-{3-bromo-2-[(2,3-dibromo-4,5- dihydroxyphenyl)methyl]-4,5- dihydroxyphenyl}ethoxy)sulfonic acid | Brc2c(Cc1c(CCOS(=O)(=O)O)cc(O)c(O)c1Br)cc(O)c(O)c2Br |
| 851 | RR014 | methyl (2S)-1-[3-bromo-2-(2,3-dibromo-4,5-dihydroxybenzyl)-4,5-dihydroxybenzyl]-5-oxopyrrolidine-2-carboxylate | Oc3cc(Cc2c(Br)c(O)c(O)cc2CN1C(=O)CCC1C(=O)OC)c(Br)c(Br)c3O |
| 852 | RR015 | N-(2,3-Dibromo-4,5-dihydroxybenzyl)-9-(&#946;-D-ribofuranosyl)-9H-purin-2-amine | Oc4cc(CNc2ncc3ncn([C@@H]1O[C@H](CO)[C@H](O)C1O)c3n2)c(Br)c(Br)c4O |
| 853 | RR016 | methyl 1-[(2,3-dibromo-4, 5-dihydroxyphenyl)methyl]-5-oxopyrrolidine-2-carboxylate | COC(=O)C1CCC(=O)N1CC2=CC(=C(C(=C2Br)Br)O)O |
| 854 | RR017 | Methyl 4-{(2,3-dibromo-4,5-dihydroxybenzyl)[(2,3-dibromo-4,5-dihydroxybenzyl)carbamoyl]amino}butanoate | Brc2c(CN(CCCC(=O)OC)C(=O)NCc1cc(O)c(O)c(Br)c1Br)cc(O)c(O)c2Br |
| 856 | RR018 | methyl 4-{[({3-bromo-2-[(2,3-dibromo-4,5- dihydroxycyclohexa-2,4-dien-1-yl)methyl]-4,5- dihydroxyphenyl}methyl)carbamoyl]amino}butanoate | O=C(OC)CCCNC(=O)NCc2cc(O)c(O)c(Br)c2CC1CC(O)=C(O)C(Br)=C1Br |
| 857 | RR019 | methyl 4-{[({3-bromo-2-[(2,3-dibromo-4,5- dihydroxyphenyl)methyl]-4,5- dihydroxyphenyl}methyl)[(2,3-dibromo-4,5- dihydroxyphenyl)methyl]carbamoyl]amino}butanoate | Brc3c(CN(Cc2cc(O)c(O)c(Br)c2Cc1cc(O)c(O)c(Br)c1Br)C(=O)NCCCC(=O)OC)cc(O)c(O)c3Br |
| 858 | RR001 | 3,4-dibromo-5-(methanesulfinylmethyl)benzene-1,2- diol | c1c(c(c(c(c1C[S](=O)C)Br)Br)O)O c1c(c(c(c(c1C[S](=O)C)Br)Br)O)O c1c(c(c(c(c1C[S](=O)C)Br)Br)O)O |
| 859 | BL002 | 3-bromo-4-[3,4-dibromo-5-(ethoxymethyl)-2-hydroxyphenoxy]-5-(ethoxymethyl)benzene-1,2-diol | Brc2c(COCC)cc(Oc1c(COCC)cc(O)c(O)c1Br)c(O)c2Br |
| 860 | BL003 | 3,4-dibromo-5-[[2,4-dihydroxy-6-(hydroxymethyl)-3-methoxyphenyl]methyl]benzene-1,2-diol | Brc2c(Cc1c(CO)cc(O)c(OC)c1O)cc(O)c(O)c2Br |
| 861 | BL004 | 3,4-dibromo-6-[(2,3-dibromo-4,5-dihydroxyphenyl)methyl]-5-(methoxymethyl)benzene-1,2-diol | Brc2c(COC)c(Cc1cc(O)c(O)c(Br)c1Br)c(O)c(O)c2Br |
| 862 | BL005 | 3,4,7,8-tetrabromo-9,10-dimethoxy-9,10-dihydroanthracene-1,2,5,6-tetrol | Oc3c2c(C(OC)c1c(O)c(O)c(Br)c(Br)c1C2OC)c(Br)c(Br)c3O |
| 863 | BL006 | 7-bromo-1-(2,3-dibromo-4,5-dihydroxyphenyl)-1,3-dihydro-2-benzofuran-5,6-diol | Oc3cc(C1OCc2cc(O)c(O)c(Br)c12)c(Br)c(Br)c3O |
| 864 | BL007 | (4aS,10aS)-6,7-Dibromo-4a-hydroxy-3,8-bis(hydroxymethyl)-10a-methoxy-4a,10a-dihydro-1(4H)-oxanthrenone | O=C1C=C(CO)C[C@]2(O)Oc3c(Br)c(Br)c(CO)cc3O[C@@]12OC |
| 865 | BL008 | (2E)-3-(2,3-Dibromo-4,5-dihydroxyphenyl)-2-methylacrylaldehyde | Brc1c(cc(O)c(O)c1Br)\C=C(/C)C=O |
| 866 | BL009 | 3,4-dibromo-5-(3-hydroxy-2-methylpropyl)benzene-1,2-diol | Brc1c(CC(C)CO)cc(O)c(O)c1Br |
| 867 | BL010 | 4,4'-Methylenebis(5,6-dibromo-1,2-benzenediol) | Brc2c(Cc1cc(O)c(O)c(Br)c1Br)cc(O)c(O)c2Br |
| 868 | BL011 | 4,4'-(oxydimethanediyl)bis(5,6-dibromobenzene-1,2-diol) | Brc2c(COCc1cc(O)c(O)c(Br)c1Br)cc(O)c(O)c2Br |
| 869 | BL012 | 3,4-dibromo-5-(hydroxymethyl)benzene-1,2-diol | Brc1c(CO)cc(O)c(O)c1Br |
| 870 | BL013 | 3,4-dibromo-5-(methoxymethyl)benzene-1,2-diol | Brc1c(COC)cc(O)c(O)c1Br |
| 871 | BL014 | 2-bromo-4,5-dihydroxybenzaldehyde | O=Cc1cc(O)c(O)cc1Br |
| 872 | BL015 | 3-Bromo-4-hydroxybenzoic acid | Oc1ccc(cc1Br)C(=O)O |
| 873 | BL016 | 2,3-dibromo-4,5-dihydroxybenzaldehyde | Brc1c(cc(O)c(O)c1Br)C=O |
| 874 | BL017 | 3-bromo-4,5-dihydroxybenzoic acid | Oc1cc(cc(Br)c1O)C(=O)OC |
| 855 | BL001 | (2E)-3-(2,3-dibromo-4,5-dihydroxyphenyl)-2- methylprop-2-enal | c1(c(c(c(cc1/C=C(/C=O)C)O)O)Br)Br |
| 875 | BL018 | 3,4-dibromo-5-[2-bromo-6-(ethoxymethyl)-3,4-dihydroxybenzyl]benzene-1,2-diol | Brc2c(Cc1c(COCC)cc(O)c(O)c1Br)cc(O)c(O)c2Br |
| 876 | BL019 | 3,4-Dibromo-5-[2-bromo-3,4-dihydroxy-6-(methoxymethyl)benzyl]benzene-1,2-diol | Brc2c(Cc1c(COC)cc(O)c(O)c1Br)cc(O)c(O)c2Br |
| 877 | BL020 | 3,4-Dibromo-5-(ethoxymethyl)-1,2-benzenediol | Brc1c(COCC)cc(O)c(O)c1Br |
| 878 | RL512 | (1S)-4-{(2R,4aR,6R,8aS)-6-[(2S,5R)-5-bromo-2,6,6-trimethyltetrahydro-2H-pyran-2-yl]-8a-methyloctahydropyrano[3,2-b]pyran-2-yl}-1-[(2R,5R)-5-(2-hydroxypropan-2-yl)-2-methyltetrahydrofuran-2-yl]pent-4-en-1-ol | Br[C@@H]1CC[C@](C)(O[C@]1(C)C)[C@H]2CC[C@]3(C)O[C@H](CC[C@H]3O2)C(=C)CC[C@H](O)[C@@]4(C)CC[C@@H](O4)C(C)(C)O |
| 879 | RL513 | (1S,4S)-4-[(4aR,6R,8aS)-6-[(2S,5R)-5-bromo-2,6,6- trimethyloxan-2-yl]-8a-methyl-4H,4aH,6H,7H,8H,8aH- pyrano[3,2-b]pyran-2-yl]-1-[(2R,5R)-5-(2- hydroxypropan-2-yl)-2-methyloxolan-2-yl]pentane- 1,5-diol | Br[C@@H]1CC[C@](C)(O[C@]1(C)C)[C@H]2CC[C@]3(C)OC(=CC[C@H]3O2)[C@@H](CC[C@H](O)[C@@]4(C)CC[C@@H](O4)C(C)(C)O)CO |
| 880 | RL514 | (4R,7S)-1-[(2R,3S,6R)-6-[(2S,5R)-5-bromo-2,6,6- trimethyloxan-2-yl]-3-hydroxy-3-methyloxan-2-yl]- 4,7-dihydroxy-4-(hydroxymethyl)-7-[(2R,5R)-5-(2- hydroxypropan-2-yl)-2-methyloxolan-2-yl]heptan-3- one | Br[C@@H]1CC[C@](C)(O[C@]1(C)C)[C@H]3CC[C@](C)(O)[C@@H](CCC(=O)[C@@](O)(CC[C@H](O)[C@@]2(C)CC[C@@H](O2)C(C)(C)O)CO)O3 |
| 881 | RL515 | (2R,4R,7S)-1-[(2R,3S,6R)-6-[(2S,5R)-5-bromo-2,6,6- trimethyloxan-2-yl]-3-hydroxy-3-methyloxan-2-yl]- 2,4,7-trihydroxy-4-(hydroxymethyl)-7-[(2R,5R)-5-(2- hydroxypropan-2-yl)-2-methyloxolan-2-yl]heptan-3- one | Br[C@@H]1CC[C@](C)(O[C@]1(C)C)[C@H]3CC[C@](C)(O)[C@@H](C[C@@H](O)C(=O)[C@@](O)(CC[C@H](O)[C@@]2(C)CC[C@@H](O2)C(C)(C)O)CO)O3 |
| 882 | RL516 | (2R,5S)-2-{(4aR,6R,8aS)-6-[(2S,5R)-5-Bromo-2,6,6-trimethyltetrahydro-2H-pyran-2-yl]-8a-methyl-4,4a,6,7,8,8a-hexahydropyrano[3,2-b]pyran-2-yl}-5-[(2R,5R)-5-(2-hydroxy-2-propanyl)-2-methyltetrahydro-2-f uranyl]-1,2,5-pentanetriol | Br[C@@H]1CC[C@](C)(O[C@]1(C)C)[C@H]2CC[C@]3(C)OC(=CC[C@H]3O2)[C@@](O)(CC[C@H](O)[C@@]4(C)CC[C@@H](O4)C(C)(C)O)CO |
| 883 | BS051 | (3E,5R)-1-(6-hydroxy-2,8-dimethyl-2H-chromen-2-yl)- 4,8,12-trimethyltrideca-3,7,11-triene-5,6-diol | C/C(C)=C\CCC(/C)=C/C(O)[C@H](O)C(\C)=C\CCC1(C)C=Cc2cc(O)cc(C)c2O1 |
| 884 | BS052 | 2-[(2E,6E,10E)-8,9-Dihydroxy-3,7,11,15-tetramethyl-2,6,10,14-hexadecatetraen-1-yl]-6-methyl-1,4-benzoquinone | O=C1C(C)=CC(=O)C=C1C\C=C(\C)CC\C=C(/C)C(O)[C@H](O)/C=C(\C)CC\C=C(/C)C |
| 885 | BS053 | (3E,5R,6R)-1-(6-methoxy-2,8-dimethyl-2H-chromen-2-yl)-4,8,12-trimethyltrideca-3,7,11-triene-5,6-diol | C/C(C)=C\CCC(/C)=C/[C@@H](O)[C@H](O)C(\C)=C\CCC1(C)C=Cc2cc(cc(C)c2O1)OC |
| 886 | BS054 | (2E)-5-(6-methoxy-2,8-dimethyl-2H-chromen-2-yl)-2-methylpent-2-enal | O=CC(\C)=C\CCC1(C)C=Cc2cc(cc(C)c2O1)OC |
| 887 | RR032 | methyl 4-{[(2,3-dibromo-4,5-dihydroxybenzyl)carbamoyl]amino}butanoate | Brc1c(CNC(=O)NCCCC(=O)OC)cc(O)c(O)c1Br |
| 888 | RR047 | methyl 3-bromo-4,5-dihydroxybenzoate | Oc1cc(cc(Br)c1O)C(=O)OC |
| 889 | RR048 | 3,4-dibromo-5-(methoxymethyl)benzene-1,2-diol | Brc1c(COC)cc(O)c(O)c1Br |
| 890 | RR049 | 4,4'-(oxydimethanediyl)bis(5,6-dibromobenzene-1,2-diol) | Brc2c(COCc1cc(O)c(O)c(Br)c1Br)cc(O)c(O)c2Br |
| 891 | RR050 | 3-bromo-4,5-dihydroxybenzaldehyde | Oc1cc(cc(Br)c1O)C=O |
| 892 | RP034 | (2R,3S,5S)-2-[(Z)-2-bromo-1-chloroethenyl]-5-(2- bromo-1-chloropropan-2-yl)-2-methyloxolan-3-ol | O[C@H]1C[C@H](O[C@@]1(C)C(\Cl)=C\Br)C(Br)(C)CCl |
| 893 | RP035 | (2S,3R,5Z,7Z)-2,8-dibromo-1,7-dichloro-2,6-dimethylocta-5,7-dien-3-ol | C\C(=C\C[C@@H](O)[C@@](Br)(C)CCl)\C(\Cl)=C\Br |
| 894 | RP036 | 6-(dibromomethyl)-2-(1,2-dichloropropan-2-yl)-5-methyl-3,6-dihydro-2H-pyran | CC1=CCC(OC1C(Br)Br)C(Cl)(C)CCl |
| 895 | RP037 | (1R,2R,4R)-2,4-dichloro-1-[(E)-2-chloroethenyl]-1-methyl-5-methylidenecyclohexane | C[C@]1(/C=C/Cl)CC(=C)[C@H](Cl)C[C@H]1Cl |
| 896 | RP038 | (1R,2R,4R,5R)-1,2,4-trichloro-5-[(E)-2-chloroethenyl]-1,5-dimethylcyclohexane | C[C@@]1(Cl)C[C@](C)(\C=C\Cl)[C@H](Cl)C[C@H]1Cl |
| 897 | RP039 | (1R,2R,4R,5S)-1,2,4-trichloro-5-[(E)-2-chloroethenyl]-1,5-dimethylcyclohexane | C[C@@]1(Cl)C[C@@](C)(\C=C\Cl)[C@H](Cl)C[C@H]1Cl |
| 898 | RP040 | (1S,2S,4R,5R)-2-bromo-1-(bromomethyl)-1,4-dichloro-5-[(E)-2-chloroethenyl]-5-methylcyclohexane | Cl[C@]1(CBr)C[C@](C)(\C=C\Cl)[C@H](Cl)C[C@@H]1Br |
| 899 | RP041 | (1R,2S,4R,5R)-2-bromo-4,5-dichloro-1-[(E)-2-chloroethenyl]-1,5-dimethylcyclohexane | C[C@]1(/C=C/Cl)C[C@@](C)(Cl)[C@H](Cl)C[C@@H]1Br |
| 900 | RP042 | (1R,2S,4R,5R)-1-(bromomethyl)-1,2,4-trichloro-5-[(E)-2-chloroethenyl]-5-methylcyclohexane | Cl[C@]1(CBr)C[C@](C)(\C=C\Cl)[C@H](Cl)C[C@@H]1Cl |
| 901 | RP062 | 2-(1-chloro-2-hydroxyethyl)-4,4-dimethylcyclohexa-2,5-dien-1-one | CC1(C)C=CC(=O)C(=C1)C(Cl)CO |
| 902 | RP063 | 4,5-dimethyl-1-benzofuran | Cc1c(C)ccc2occc12 |
| 903 | RP064 | (3R)-6-bromo-3-(bromomethyl)-3,7-dichloro-2,7-dimethyloct-1-ene | CC(C)(Cl)[C@H](Br)CC[C@@](Cl)(CBr)C(=C)C |
| 904 | RP065 | 2-chloro-2-(3,3-dimethyl-6-oxocyclohexa-1,4-dien-1-yl)ethyl acetate | CC1(C)C=CC(=O)C(=C1)C(Cl)COC(C)=O |
| 905 | RP066 | 2-(1,2-dibromoethyl)-4,4-dimethylcyclohexa-2,5-dien-1-one | CC1(C)C=CC(=O)C(=C1)C(Br)CBr |
| 906 | RP067 | 2-(2-bromo-1-chloroethyl)-4,4-dimethylcyclohexa-2,5-dien-1-one | CC1(C)C=CC(=O)C(=C1)C(Cl)CBr |
| 907 | RP068 | 2-(1,2-dichloroethyl)-4,4-dimethylcyclohexa-2,5-dien-1-one | CC1(C)C=CC(=O)C(=C1)C(Cl)CCl |
| 908 | RP069 | 2-[(Z)-2-bromoethenyl]-4,4-dimethylcyclohexa-2,5-dien-1-one | CC1(C)C=CC(=O)C(/C=C\Br)=C1 |
| 909 | RP070 | 2-[(Z)-2-chloroethenyl]-4,4-dimethylcyclohexa-2,5-dien-1-one | CC1(C)C=CC(=O)C(/C=C\Cl)=C1 |
| 910 | RR067 | 1-(2,3-Dibromo-4,5-dihydroxybenzyl)-5-oxoproline | O=C(O)C2CCC(=O)N2Cc1cc(O)c(O)c(Br)c1Br |
| 911 | RP026 | (2S,3Z,4S,6S)-6-bromo-2,4-dichloro-3-(2- chloroethylidene)-1,1-dimethylcyclohexane | C[C@]1(C)[C@@H](Cl)C(=C\CCl)/[C@@H](Cl)C[C@@H]1Br |
| 912 | RP027 | (2S,3E,4S,6S)-6-bromo-2,4-dichloro-3-(2-chloroethylidene)-1,1-dimethylcyclohexane | C[C@]1(C)[C@@H](Cl)C(=C\CCl)\[C@@H](Cl)C[C@@H]1Br |
| 913 | RP028 | (2S,3E,4S,6S)-6-bromo-3-(2-bromoethylidene)-2,4-dichloro-1,1-dimethylcyclohexane | C[C@]1(C)[C@@H](Cl)C(=C\CBr)\[C@@H](Cl)C[C@@H]1Br |
| 914 | RP029 | (3Z)-4-Bromo-2,6-dichloro-3-(2-chloroethylidene)-1,1-dimethylcyclohexane | C[C@@]1(C)C(Cl)\C(=C\CCl)C(Br)C[C@@H]1Cl |
| 915 | RP030 | (3S,6R)-6-bromo-3-(bromomethyl)-2,3,7-trichloro-7- methyloct-1-ene | CC(C)(Cl)[C@H](Br)CC[C@@](Cl)(CBr)C(=C)Cl |
| 916 | RP031 | (6E)-3,5-dichloro-6-(2-chloroethylidene)-4,4- dimethylcyclohex-1-ene | CC1(C)[C@H](Cl)C=C/C(=C\CCl)[C@@H]1Cl |
| 917 | RP032 | (1E,5E)-8-bromo-1,3,4,7-tetrachloro-7- (chloromethyl)-3-methylocta-1,5-diene | Cl\C=C\C(C)(Cl)C(Cl)/C=C/C(Cl)(CCl)CBr |
| 918 | RP033 | (1S,2S,4R,5R)-1,2,4-trichloro-5-[(E)-2- chloroethenyl]-1,5-dimethylcyclohexane | C[C@]1(Cl)C[C@](C)(\C=C\Cl)[C@H](Cl)C[C@@H]1Cl |
| 919 | RR020 | 7-({3-bromo-2-[(2,3-dibromo-4,5- dihydroxyphenyl)methyl]-4,5- dihydroxyphenyl}methyl)-2,3,6,7-tetrahydro-1H- purine-2,6-dione | Oc4cc(Cc3c(Br)c(O)c(O)cc3Cn2cnc1NC(=O)NC(=O)c12)c(Br)c(Br)c4O |
| 920 | RR021 | 7-(2,3-dibromo-4,5-dihydroxybenzyl)-3,7-dihydro-1H-purine-2,6-dione | Oc3cc(Cn2cnc1NC(=O)NC(=O)c12)c(Br)c(Br)c3O |
| 921 | RR022 | 9-(3-bromo-2-(2,3-dibromo-4,5-dihydroxybenzyl)-4,5-dihydroxybenzyl)adenine | Oc4cc(Cc3c(Br)c(O)c(O)cc3Cn2cnc1c(N)ncnc12)c(Br)c(Br)c4O |
| 922 | RR023 | (2S)-2-amino-3-(3-bromo-5-hydroxy-4-methoxyphenyl)propanoic acid | COc1c(O)cc(cc1Br)C[C@H](N)C(=O)O |
| 923 | RR024 | 8-bromo-6-hydroxy-7-methoxy-1,2,3,4- tetrahydroisoquinoline-3-carboxylic acid | O=C(O)[C@@H]1Cc2cc(O)c(OC)c(Br)c2CN1 |
| 924 | RR025 | methyl (3S)-8-bromo-6-hydroxy-7-methoxy-1,2,3,4-tetrahydroisoquinoline-3-carboxylate | O=C(OC)[C@@H]1Cc2cc(O)c(OC)c(Br)c2CN1 |
| 925 | RR026 | methyl 6-bromo-8-hydroxy-7-methoxy-1,2,3,4- tetrahydroisoquinoline-3-carboxylate | O=C(OC)[C@@H]1Cc2cc(Br)c(OC)c(O)c2CN1 |
| 926 | RR027 | 3,4-dibromo-5-({3-bromo-2-[(2,3-dibromo-4,5- dihydroxyphenyl)methyl]-4,5- dihydroxyphenyl}methyl)benzene-1,2-diol | Brc3c(Cc2c(Cc1cc(O)c(O)c(Br)c1Br)cc(O)c(O)c2Br)cc(O)c(O)c3Br |
| 927 | GA001 | 3-bromo-4-(3-bromo-4,5-dihydroxybenzyl)-5-(hydroxymethyl)benzene-1,2-diol | Oc1cc(cc(Br)c1O)Cc2c(CO)cc(O)c(O)c2Br |
| 928 | GA002 | 1-{[6-(aminomethyl)-3,4,5-trihydroxyoxan-2-yl]oxy}-3-  (octadecanoyloxy)propan-2-yl octadecanoate | CCCCCCCCCCCCCCCCCC(=O)OC[C@H](CO[C@H]1OC(CN)[C@@H](O)[C@H](O)C1O)OC(=O)CCCCCCCCCCCCCCCCC |
| 929 | GA003 | (3R,3'R,6'R)-3,19,3'-Trihydroxy-7,8-dihydro-b,e-caroten-8-one | CC2(C)C[C@H](O)CC(C)=C2CC(=O)C(=C\C=C\C(\C)=C\C=C\C=C(/C)\C=C\C=C(/C)\C=C\[C@H]1C(C)=C[C@H](O)CC1(C)C)\CO |
| 930 | RO001 | (3aR,6aR)-4-(2,3-dibromo-4,5-dihydroxybenzyl)-6a-methoxyhexahydropyrrolo[2,3-d]imidazol-2(1H)-one | Oc3cc(CN1CC[C@@]2(NC(=O)N[C@H]12)OC)c(Br)c(Br)c3O |
| 931 | RO002 | 3,4-dibromo-5-(hydroxymethyl)benzene-1,2-diol | Brc1c(CO)cc(O)c(O)c1Br |
| 932 | RO003 | 3,4-dibromo-5-(methoxymethyl)benzene-1,2-diol | Brc1c(COC)cc(O)c(O)c1Br |
| 933 | RO004 | 2,3-dibromo-4,5-dihydroxybenzaldehyde | Brc1c(cc(O)c(O)c1Br)C=O |
| 934 | RO005 | (3E)-4-(2,3-dibromo-4,5-dihydroxyphenyl)but-3-en-2-one | Brc1c(/C=C/C(C)=O)cc(O)c(O)c1Br |
| 935 | RO006 | (3S,3aS,6S,6aR)-3-(2,3-dibromo-4,5-dihydroxybenzyl)-3,3a,6-trihydroxytetrahydrofuro[3,2-b]furan-2(3H)-one | O[C@H]2CO[C@]3(O)[C@@](O)(Cc1cc(O)c(O)c(Br)c1Br)C(=O)O[C@H]23 |
| 936 | RR028 | 2,3-dibromo-4,5-dihydroxybenzaldehyde | Brc1c(C=O)cc(O)c(O)c1Br |
| 937 | RR029 | 2,3-dibromo-4,5-dimethoxybenzaldehyde | Brc1c(C=O)cc(OC)c(OC)c1Br |
| 938 | RR030 | 3,4-dibromo-5-(methoxymethyl)benzene-1,2-diol | Brc1c(COC)cc(O)c(O)c1Br |
| 939 | RR033 | 4,4'-[(3-bromo-4,5-dihydroxybenzene-1,2-diyl)dimethanediyl]bis(5,6-dibromobenzene-1,2-diol) | Brc3c(Cc2c(Cc1cc(O)c(O)c(Br)c1Br)cc(O)c(O)c2Br)cc(O)c(O)c3Br |
| 940 | RR034 | 3,4-dibromo-5-[2-bromo-3,4-dihydroxy-6-(hydroxymethyl)benzyl]benzene-1,2-diol | Brc2c(Cc1c(CO)cc(O)c(O)c1Br)cc(O)c(O)c2Br |
| 941 | RR035 | 3,4-dibromo-5-[2-bromo-6-(ethoxymethyl)-3,4-dihydroxybenzyl]benzene-1,2-diol | Brc2c(Cc1c(COCC)cc(O)c(O)c1Br)cc(O)c(O)c2Br |
| 942 | RR036 | 3-(2,3-dibromo-4,5-dihydroxyphenyl)-2-methylpropanal | Brc1c(CC(C)C=O)cc(O)c(O)c1Br |
| 943 | RR037 | 3,4-dibromo-5-[(2R)-3,3-dimethoxy-2-methylpropyl]benzene-1,2-diol | Brc1c(CC(C)C(OC)OC)cc(O)c(O)c1Br |
| 944 | RR038 | methyl 3-bromo-4,5-dihydroxybenzoate | Oc1cc(cc(Br)c1O)C(=O)OC |
| 945 | RR039 | 3-bromo-4,5-dihydroxybenzaldehyde | Oc1cc(cc(Br)c1O)C=O |
| 946 | RR040 | 3,4-dibromo-5-(hydroxymethyl)benzene-1,2-diol | Brc1c(CO)cc(O)c(O)c1Br |
| 947 | RR041 | 3,4-dibromo-5-(methoxymethyl)benzene-1,2-diol | Brc1c(COC)cc(O)c(O)c1Br |
| 948 | RR042 | 3,4-dibromo-5-(ethoxymethyl)benzene-1,2-diol | Brc1c(COCC)cc(O)c(O)c1Br |
| 949 | RR043 | 2,3-dibromo-4,5-dihydroxybenzaldehyde | Brc1c(cc(O)c(O)c1Br)C=O |
| 950 | RR044 | 4,4'-(oxydimethanediyl)bis(5,6-dibromobenzene-1,2-diol) | Brc2c(COCc1cc(O)c(O)c(Br)c1Br)cc(O)c(O)c2Br |
| 951 | RR045 | 3,4-dibromo-5-[2-bromo-3,4-dihydroxy-6-(methoxymethyl)benzyl]benzene-1,2-diol | Brc2c(Cc1c(COC)cc(O)c(O)c1Br)cc(O)c(O)c2Br |
| 952 | RR046 | 4,4'-methanediylbis(5,6-dibromobenzene-1,2-diol) | Brc2c(Cc1cc(O)c(O)c(Br)c1Br)cc(O)c(O)c2Br |
| 953 | RP044 | (3S,6R)-6-bromo-3-(bromomethyl)-2,3,7-trichloro-7-methyloct-1-ene | CC(C)(Cl)[C@H](Br)CC[C@@](Cl)(CBr)C(=C)Cl |
| 954 | RP045 | (3Z)-6-bromo-3-(bromomethylidene)-2-chloro-7-methylocta-1,6-diene | C=C(Cl)C(/CCC(/Br)=C(/C)C)=C\Br |
| 955 | RP046 | (3Z)-6-Bromo-3-(bromomethylene)-2,7-dichloro-7-methyl-1-octene | CC(C)(Cl)C(Br)CC\C(=C\Br)C(=C)Cl |
| 956 | RP047 | (3Z)-6-bromo-2-chloro-3-(chloromethylidene)-7-methylocta-1,6-diene | C=C(Cl)C(/CCC(/Br)=C(/C)C)=C\Cl |
| 957 | RP048 | (3S)-3-(bromomethyl)-2,3-dichloro-7-methylocta-1,6-diene | C\C(C)=C\CC[C@@](Cl)(CBr)C(=C)Cl |
| 958 | RP049 | (3S,6R)-6-bromo-3-(bromomethyl)-3,7-dichloro-7-methyloct-1-ene | CC(C)(Cl)[C@H](Br)CC[C@](Cl)(C=C)CBr |
| 959 | RP050 | (2R,3S,5R)-2-[(Z)-2-bromo-1-chloroethenyl]-5-(2- bromo-1-chloropropan-2-yl)-2-me | O[C@H]1C[C@@H](O[C@@]1(C)C(Cl)=[C@H]Br)C(Br)(C)CCl |
| 960 | RP051 | (1R)-1,4-Anhydro-1-[(2R)-2-bromo-1-chloro-2-propanyl]-4-[(Z)-2-bromo-1-chlorovinyl]-2,5-dideoxy-L-threo-pentitol | O[C@H]1C[C@@H](O[C@]1(C)C(\Cl)=C\Br)C(Br)(C)CCl |
| 961 | RP052 | (1R)-1,4-Anhydro-1-[(2R)-2-bromo-1-chloro-2-propanyl]-4-[(Z)-2-bromo-1-chlorovinyl]-2,5-dideoxy-D-erythro-pentitol | O[C@H]1C[C@H](O[C@@]1(C)C(\Cl)=C\Br)C(Br)(C)CCl |
| 1006 | RR069 | (3E)-4-(3-bromo-4,5-dihydroxyphenyl)but-3-en-2-one | Oc1cc(cc(Br)c1O)/C=C/C(C)=O |
| 1005 | RR068 | 3-bromo-5-hydroxy-4-methoxybenzaldehyde | COc1c(O)cc(cc1Br)C=O |
| 1004 | RR070 | 3-bromo-5-hydroxy-4-methoxybenzoic acid | COc1c(O)cc(cc1Br)C(=O)O |
| 987 | RP072 | 2-chloro-7-methyl-3-methylideneocta-1,6-diene | C=C(Cl)C(=C)CC\C=C(\C)C |
| 983 | RP071 | (3E)-6-bromo-2,4-dichloro-3-(2-chloroethylidene)-1,1-dimethylcyclohexane | CC1(C)C(Cl)C(=C\CCl)\C(Cl)CC1Br |
| 981 | RO010 | (6Z)-6-(bromomethylidene)-3-chloro-2-methylocta-1,7-diene | CC(=C)C(Cl)CC\C(=C\Br)C=C |
| 986 | RP061 | (6Z)-6-(bromomethylidene)-3-chloro-2-methylocta-1,7-diene | CC(=C)C(Cl)CC\C(=C\Br)C=C |
| 984 | RP059 | (3E)-3-(bromomethylidene)-7-methylocta-1,6-diene | C/C(C)=C\CCC(=C\Br)/C=C |
| 962 | RP053 | (2E,4R,6R)-4,6-dibromo-3,7-dimethylocta-2,7-dienal | CC(=C)C(Br)CC(Br)C(/C)=C/C=O |
| 963 | RP054 | (2E,4Z,6E)-4,8-dichloro-3,7-dimethylocta-2,4,6-trienal | Cl/C(=C\C=C(/C)CCl)/C(/C)=C/C=O |
| 964 | RP055 | (2E,4E)-8-bromo-6,7-dichloro-3,7-dimethylocta-2,4-dienal | ClC(/C=C/C(/C)=C/C=O)C(C)(Cl)CBr |
| 965 | RP056 | (2E,6E)-4-bromo-8-chloro-3,7-dimethylocta-2,6-dienal | C/C(CCl)=C\CC(Br)C(/C)=C/C=O |
| 966 | RP057 | (3R,5R,6E)-3,5-dibromo-8,8-dichloro-2,6-dimethylocta-1,6-diene | ClC(Cl)/C=C(\C)C(Br)CC(Br)C(=C)C |
| 967 | RP058 | (1E,3R,4S,5E)-1,4,8-tribromo-3,7-dichloro-3,7-dimethylocta-1,5-diene | C[C@@](Cl)(/C=C/Br)[C@@H](Br)/C=C/C(Cl)(C)CBr |
| 968 | BE013 | 5,6,11,14-tetraoxapentaphene-1,3,7,9,12-pentol | Oc5cc(O)cc4Oc1c(cc(O)c2Oc3cc(O)cc(O)c3Oc12)Oc45 |
| 838 | RP043 | 7-methyl-3-methylideneocta-1,6-diene | C/C(C)=C\CC/C(C=C)=C |
| 969 | BE014 | 4-(3,5-dihydroxyphenoxy)oxanthrene-1,3,6,8-tetrol | Oc1cc(cc(O)c1)Oc3c(O)cc(O)c2Oc4cc(O)cc(O)c4Oc23 |
| 970 | BE015 | 4,9-Bis(3,5-dihydroxyphenoxy)[1]benzofuro[3,2-a]oxanthrene-1,3,6,10,12-pentol | Oc1cc(cc(O)c1)Oc5c(O)cc(O)c4c6c3Oc7c(O)cc(O)c(Oc2cc(O)cc(O)c2)c7Oc3c(O)cc6oc45 |
| 971 | BE016 | 4-(4-{[6-(3,5-dihydroxyphenoxy)-4,7,9-  trihydroxyoxanthren-2-yl]oxy}-3,5-  dihydroxyphenoxy)oxanthrene-1,3,6,8-tetrol | Oc6cc7Oc8c(O)cc(O)c(Oc5cc(O)c(Oc3cc2Oc4c(O)cc(O)c(Oc1cc(O)cc(O)c1)c4Oc2c(O)c3)c(O)c5)c8Oc7c(O)c6 |
| 972 | BS055 | (1S,2S,11S,14S,15R,18S,20R)-1,8,11,15,19,19- hexamethyl-10- oxapentacyclo[12.8.0.0^{2,11}.0^{4,9}.0^{15,20}]docosa- 4(9),5,7-triene-6,18-diol | Oc2cc3C[C@H]4[C@@]5(C)CC[C@H]1[C@](C)(C)[C@@H](O)CC[C@]1(C)[C@@H]5CC[C@]4(C)Oc3c(C)c2 |
| 973 | BS056 | (2S,4aR,4bR,6aR,12aS,12bR,14aR)-1,1,4a,6a,8,12b-Hexamethyl-2,3,4,4a,4b,5,6,6a,12,12a,12b,13,14,14a-tetradecahydro-1H-naphtho[2,1-a]xanthene-2,10-diyl diacetate | CC(=O)Oc2cc3C[C@H]4[C@@]5(C)CC[C@H]1[C@](C)(C)[C@@H](OC(C)=O)CC[C@]1(C)[C@@H]5CC[C@]4(C)Oc3c(C)c2 |
| 974 | BS057 | 10-Hydroxy-1,1,4a,6a,8,12b-hexamethyl-2,3,4,4a,4b,5,6,6a,12,12a,12b,13,14,14a-tetradecahydro-1H-naphtho[2,1-a]xanthen-2-yl acetate | CC(=O)O[C@H]5CC[C@@]4(C)[C@@H](CC[C@@]1(C)[C@H]4CC[C@]2(C)Oc3c(C)cc(O)cc3C[C@@H]12)[C@]5(C)C |
| 975 | BS058 | (2S,2'S,4a'R,4b'S,7'S,8a'R,10a'R)-2',4b',7,8',8',10a'-Hexamethyl-3',4',4a',4b',5',6',7',8',8a',9',10',10a'-dodecahydro-2'H,3H-spiro[1-benzofuran-2,1'-phenanthrene]-4,5,7'-triyl triacetate | CC(=O)Oc1c(OC(C)=O)cc(C)c2O[C@@]4(Cc12)[C@@H](C)CC[C@@H]3[C@@]5(C)CC[C@H](OC(C)=O)[C@@](C)(C)[C@@H]5CC[C@]34C |
| 976 | BS059 | (2S,2'S,4'aR,4'bR,8'aR,10'aR)-5-(acetyloxy)-2',4'b,7,8',8',10'a-hexamethyl-7'-oxo-3',4',4'a,4'b,5',6',7',8',8'a,9',10',10'a-dodecahydro-2'H,3H-spiro[1-benzofuran-2,1'-phenanthrene]-4-yl acetate | CC(=O)Oc1c(OC(C)=O)cc(C)c2O[C@@]4(Cc12)[C@@H](C)CC[C@@H]3[C@@]5(C)CCC(=O)[C@](C)(C)[C@@H]5CC[C@]34C |
| 977 | BS060 | (2S,4'bS,7'S,10'aR)-2',4'b,7,8',8',10'a-hexamethyl- 3',4',4'a,4'b,5',6',7',8',8'a,9',10',10'a- dodecahydro-2'H,3H-spiro[1-benzofuran-2,1'- phenanthrene]-5,7'-diol | Oc1cc2C[C@]4(Oc2c(C)c1)[C@@H](C)CC[C@@H]3[C@@]5(C)CCC(O)[C@@](C)(C)[C@@H]5CC[C@]34C |
| 978 | RO007 | 7-methyl-3-methylideneocta-1,6-diene | C/C(C)=C\CC/C(C=C)=C |
| 979 | RO008 | (3E)-3-(bromomethylidene)-7-methylocta-1,6-diene | C/C(C)=C\CCC(=C\Br)/C=C |
| 980 | RO009 | (3Z)-3-(bromomethylidene)-7-methylocta-1,6-diene | C/C(C)=C\CCC(=C\Br)\C=C |
| 985 | RP060 | (3Z)-3-(bromomethylidene)-7-methylocta-1,6-diene | C/C(C)=C\CCC(=C\Br)\C=C |
| 982 | RO011 | (3E)-6-bromo-2,4-dichloro-3-(2-chloroethylidene)-1,1-dimethylcyclohexane | CC1(C)C(Cl)C(=C\CCl)\C(Cl)CC1Br |
| 988 | RR051 | 3,4-dibromo-5-[(methylsulfonyl)methyl]benzene-1,2-diol | Brc1c(CS(C)(=O)=O)cc(O)c(O)c1Br |
| 989 | RR052 | 3,4-dibromo-5-[(2,3-dihydroxypropoxy)methyl]benzene-1,2-diol | Brc1c(COCC(O)CO)cc(O)c(O)c1Br |
| 990 | RR053 | 5-(aminomethyl)-3,4-dibromobenzene-1,2-diol | Brc1c(CN)cc(O)c(O)c1Br |
| 991 | RR054 | (2,3-dibromo-4,5-dihydroxyphenyl)acetic acid | Brc1c(CC(=O)O)cc(O)c(O)c1Br |
| 992 | RR055 | 3-bromo-5-(hydroxymethyl)-2-methoxyphenol | COc1c(O)cc(cc1Br)CO |
| 993 | RR056 | (3E)-4-(2-bromo-4,5-dihydroxyphenyl)but-3-en-2-one | Brc1cc(O)c(O)cc1/C=C/C(C)=O |
| 994 | RR057 | (3E)-4-(2,3-dibromo-4,5-dihydroxyphenyl)but-3-en-2-one | Brc1c(/C=C/C(C)=O)cc(O)c(O)c1Br |
| 995 | RR058 | 3-(2,3-Dibromo-4,5-dihydroxyphenyl)-2-methylpropanal | Brc1c(CC(C)C=O)cc(O)c(O)c1Br |
| 996 | RR059 | 3,4-dibromo-5-(2-hydroxyethyl)benzene-1,2-diol | Brc1c(CCO)cc(O)c(O)c1Br |
| 997 | RR060 | 3,4-dibromo-5-(hydroxymethyl)  benzene-1,2-diol | Brc1c(CO)cc(O)c(O)c1Br |
| 998 | RR061 | 3,4-dibromo-5-(methoxymethyl)benzene-1,2-diol | Brc1c(COC)cc(O)c(O)c1Br |
| 999 | RR062 | 3,4-dibromo-5-(ethoxymethyl)benzene-1,2-diol | Brc1c(COCC)cc(O)c(O)c1Br |
| 1000 | RR063 | 2,3-dibromo-4,5-dihydroxybenzaldehyde | Brc1c(cc(O)c(O)c1Br)C=O |
| 1001 | RR064 | 2-amino-3-(3-bromo-5-hydroxy-4-methoxyphenyl)propanoic acid | COc1c(O)cc(cc1Br)CC(N)C(=O)O |
| 1002 | RR065 | (3-bromo-5-hydroxy-4-methoxyphenyl)acetic acid | COc1c(O)cc(cc1Br)CC(=O)O |
| 1003 | RR066 | methyl (3-bromo-5-hydroxy-4-methoxyphenyl)acetate | COc1c(O)cc(cc1Br)CC(=O)OC |
| 1007 | BS061 | (2R,5S,15R)-2,15-dimethyl-14-[(5Z)-5-(propan-2- yl)hept-5-en-2- yl]tetracyclo[8.7.0.0^{2,7}.0^{11,15}]heptadec-7- en-5-ol | CC(C)C(=C\C)/CCC(C)C1CCC2C3CC=C4C[C@@H](O)CC[C@]4(C)C3CC[C@]12C |
| 1008 | BS062 | 24-vinyl cholest-4-ene-3-one | CC(C)C(O)(C=C)CCC(C)C2CCC1C3CC=C4CC(=O)CC[C@]4(C)C3CC[C@@]12C |
| 1009 | BS063 | (3&#946;)-Stigmasta-5,28-diene-3,24-diol | CC(C)C(O)(C=C)CCC(C)C1CCC2C3CC=C4CC(O)CC[C@]4(C)C3CC[C@]12C |
| 1010 | BS064 | (3S,5R,6S,3'S,5'R,6'R)-3,5'-dihydroxy-8-oxo-6',7'-didehydro-5,6-epoxy-5,6,7,8,5',6'-hexahydro-&#946;,&#946;-caroten-3'-yl acetate | CC(=O)O[C@H]3CC(C)(C)/C(=C=C\C(\C)=C\C=C\C(\C)=C\C=C\C=C(/C)\C=C\C=C(/C)C(=O)C[C@]12O[C@]2(C)C[C@@H](O)CC1(C)C)[C@](C)(O)C3 |
| 1011 | BS065 | 4-(acetyloxy)-3-methyl-5-[(2E,6E,10E)-3,7,11,15-tetramethylhexadeca-2,6,10,14-tetraen-1-yl]phenyl acetate | O=C(C)Oc1c(C)cc(cc1C\C=C(/C)CC\C=C(/C)CC\C=C(/C)CC\C=C(/C)C)OC(C)=O |
| 1012 | BS066 | 2-[2'(E)-3',7',11',15'-tetramethylhexadec-2-en-1'-yl]-6-methyl-1,4-benzohydroquinone diacetate | O=C(C)Oc1c(C)cc(cc1C\C=C(/C)CCCC(C)CCCC(C)CCCC(C)C)OC(C)=O |
| 1013 | BS067 | (2S,4aR,4bS,6aR,12aR,12bS,14aR)-1,1,4a,6a,8,12b-Hexamethyl-2,3,4,4a,4b,5,6,6a,12,12a,12b,13,14,14a-tetradecahydro-1H-naphtho[2,1-a]xanthene-2,10-diyl diacetate | CC(=O)Oc2cc3C[C@H]4[C@@]5(C)CC[C@H]1[C@](C)(C)[C@@H](OC(C)=O)CC[C@]1(C)[C@@H]5CC[C@]4(C)Oc3c(C)c2 |
| 1014 | BS068 | (2S,4aR,4bR,6aR,12aS,12bR,14aR)-1,1,4a,6a,8,12b-Hexamethyl-2,3,4,4a,4b,5,6,6a,12,12a,12b,13,14,14a-tetradecahydro-1H-naphtho[2,1-a]xanthene-2,10-diyl diacetate | CC(=O)Oc2cc3C[C@@H]4[C@]5(C)CC[C@H]1[C@](C)(C)[C@@H](OC(C)=O)CC[C@]1(C)[C@H]5CC[C@@]4(C)Oc3c(C)c2 |
| 1015 | BS069 | (2S,2'S,4'aR,4'bS,7'S,8'aR,10'aR)-5-(acetyloxy)-2',4'b,7,8',8',10'a-hexamethyl-3',4',4'a,4'b,5',6',7',8',8'a,9',10',10'a-dodecahydro-2'H,3H-spiro[1-benzofuran-2,1'-phenanthrene]-7'-yl acetate | CC(=O)Oc1cc2C[C@]4(Oc2c(C)c1)[C@@H](C)CC[C@@H]3[C@@]5(C)CC[C@H](OC(C)=O)[C@@](C)(C)[C@@H]5CC[C@]34C |
| 1016 | BS070 | (2S,2'S,4a'R,4b'S,7'S,8a'R,10a'R)-2',4b',7,8',8',10a'-Hexamethyl-3',4',4a',4b',5',6',7',8',8a',9',10',10a'-dodecahydro-2'H,3H-spiro[1-benzofuran-2,1'-phenanthrene]-4,5,7'-triyl triacetate | CC(=O)Oc1c(OC(C)=O)cc(C)c2O[C@@]4(Cc12)[C@@H](C)CC[C@@H]3[C@@]5(C)CC[C@H](OC(C)=O)[C@@](C)(C)[C@@H]5CC[C@]34C |
| 1017 | BS071 | (2S,2'S,4a'R,4b'S,7'S,8a'R,10a'R)-6-Chloro-2',4b',7,8',8',10a'-hexamethyl-3',4',4a',4b',5',6',7',8',8a',9',10',10a'-dodecahydro-2'H,3H-spiro[1-benzofuran-2,1'-phenanthrene]-4,5,7'-triyl triacetate | CC(=O)Oc2c1C[C@]4(Oc1c(C)c(Cl)c2OC(C)=O)[C@@H](C)CC[C@@H]3[C@@]5(C)CC[C@H](OC(C)=O)[C@@](C)(C)[C@@H]5CC[C@]34C |
| 1018 | BS072 | (2S,2'S,4'aR,4'bR,8'aR,10'aR)-5-(acetyloxy)-2',4'b,7,8',8',10'a-hexamethyl-7'-oxo-3',4',4'a,4'b,5',6',7',8',8'a,9',10',10'a-dodecahydro-2'H,3H-spiro[1-benzofuran-2,1'-phenanthrene]-4-yl acetate | CC(=O)Oc1c(OC(C)=O)cc(C)c2O[C@@]4(Cc12)[C@@H](C)CC[C@@H]3[C@@]5(C)CCC(=O)[C@](C)(C)[C@@H]5CC[C@]34C |
| 1019 | BS073 | 2,8-dimethyl-2-[(3E,7E)-4,8,12-trimethyltrideca-3,7,11-trien-1-yl]-2H-chromen-6-yl acetate | CC(=O)Oc1cc(C)c2OC(C)(C=Cc2c1)CC\C=C(/C)CC\C=C(/C)CC\C=C(/C)C |
| 1020 | BS074 | 3,5-bis(acetyloxy)-4-hexadecanoylphenyl acetate | O=C(C)Oc1cc(cc(OC(C)=O)c1C(=O)CCCCCCCCCCCCCCC)OC(C)=O |
| 1021 | GA004 | 2,3-Dihydroxypropyl (5&#958;)-6-O-[(4Z,8E,13Z)-1-carboxy-10-{[(4Z,9E)-15-carboxy-8,11-dihydroxy-4,9-pentadecadien-7-yl]oxy}-11-hydroxy-4,8,13-nonadecatrien-7-yl]-&#945;-L-arabino-hexopyranoside | O=C(O)CCCCC(O)/C=C/C(O)C(C/C=C\CCC)OC(/C=C/C(C/C=C\CCCC(=O)O)OCC1OC(OCC(O)CO)C(O)C(O)C1O)C(O)C/C=C\CCCCC |
| 1022 | GA005 | 2,3-Dihydroxypropyl (5&#958;)-6-O-[(4Z,8E,13Z,16Z)-1-carboxy-10-{[(4Z,9E)-15-carboxy-8,11-dihydroxy-4,9-pentadecadien-7-yl]oxy}-11-hydroxy-4,8,13,16-nonadecatetraen-7-yl]-&#945;-L-arabino-hexopyranoside | O=C(O)CCCCC(O)/C=C/C(O)C(C/C=C\CCC)OC(/C=C/C(C/C=C\CCCC(=O)O)OCC1OC(OCC(O)CO)C(O)C(O)C1O)C(O)C/C=C\C/C=C\CC |
| 1023 | GA006 | 2,3-Dihydroxypropyl 6-O-[(5Z,9E,14Z)-11-{[(4Z,9E)-8,11-dihydroxy-16-methoxy-16-oxo-4,9-hexadecadien-7-yl]oxy}-12-hydroxy-1-methoxy-1-oxo-5,9,14-icosatrien-8-yl]-&#946;-D-galactopyranoside | O=C(OC)CCCCC(O)/C=C/C(O)C(C/C=C\CCC)OC(/C=C/C(C/C=C\CCCC(=O)OC)OCC1OC(OCC(O)CO)C(O)C(O)C1O)C(O)C/C=C\CCCCC |
| 1024 | GA007 | methyl (5Z,9E,14Z,17Z)-11-{[(4Z,9E)-8,11-dihydroxy-16-methoxy-16-oxohexadeca-4,9-dien-7-yl]oxy}-8-{[6-(2,3-dihydroxypropoxy)-3,4,5-trihydroxyoxan-2-yl]methoxy}-12-hydroxyicosa-5,9,14,17-tetraenoate | O=C(OC)CCCCC(O)/C=C/C(O)C(C/C=C\CCC)OC(/C=C/C(C/C=C\CCCC(=O)OC)OCC1OC(OCC(O)CO)C(O)C(O)C1O)C(O)C/C=C\C/C=C\CC |
| 1025 | RP073 | (1R,2S,4S,5R)-1-bromo-4,5-dichloro-2-[(E)-2-chloroethenyl]-1,5-dimethylcyclohexane | C[C@@]1(Cl)C[C@](Br)(C)[C@@H](C[C@@H]1Cl)\C=C\Cl |
| 1026 | RP074 | (1S,2S,4R,5R)-2-bromo-1-(bromomethyl)-1,4-dichloro-5-[(E)-2-chloroethenyl]-5-methylcyclohexane | Cl[C@]1(CBr)C[C@](C)(\C=C\Cl)[C@H](Cl)C[C@@H]1Br |
| 1027 | RP075 | (1R,2S,4S,5R)-2,4-dibromo-1-chloro-5-[(E)-2-chloroethenyl]-1,5-dimethylcyclohexane | C[C@@]1(Cl)C[C@](C)(\C=C\Cl)[C@@H](Br)C[C@@H]1Br |
| 1028 | RP076 | (1R,2S,4S,5R)-1,4-dibromo-5-chloro-2-[(E)-2-chloroethenyl]-1,5-dimethylcyclohexane | C[C@@]1(Cl)C[C@](Br)(C)[C@@H](C[C@@H]1Br)\C=C\Cl |
| 1029 | RP077 | (1S,2R,4S,5S)-2-bromo-4,5-dichloro-1-[(E)-2-chloroethenyl]-1,5-dimethylcyclohexane | C[C@@]1(/C=C/Cl)C[C@](C)(Cl)[C@@H](Cl)C[C@H]1Br |
| 1030 | RP078 | (1R,2S,4R,5S)-1,2,4-trichloro-5-[(E)-2-chloroethenyl]-1,5-dimethylcyclohexane | C[C@@]1(Cl)C[C@@](C)(\C=C\Cl)[C@H](Cl)C[C@@H]1Cl |
| 1031 | RC003 | (7S,8S,11R,12S,15S)-7,11-dibromo-12,21-dihydroxy- 15-(2-hydroxypropan-2-yl)-4,8,12-trimethyl-16- oxatricyclo[16.3.1.0^{3,8}]docosa-1(22),3,18,20- tetraen-17-one | CC(C)(O)[C@@H]2CC[C@](C)(O)[C@H](Br)CC[C@@]3(C)C(Cc1cc(ccc1O)C(=O)O2)=C(C)CC[C@@H]3Br |
| 1042 | GA009 | 6-bromo-3-(3-bromo-4-hydroxybenzyl)-4-(methoxymethyl)benzene-1,2-diol | Oc2c(Cc1ccc(O)c(Br)c1)c(cc(Br)c2O)COC |
| 1032 | RC004 | (3R,7S,8S,11R,12S,15S)-7,11-dibromo-15-(2- bromopropan-2-yl)-12,21-dihydroxy-8,12-dimethyl-4- methylidene-16-oxatricyclo[16.3.1.0^{3,8}]docosa- 1(22),18,20-trien-17-one | BrC(C)(C)[C@@H]2CC[C@](C)(O)[C@H](Br)CC[C@@]3(C)[C@H](Cc1cc(ccc1O)C(=O)O2)C(=C)CC[C@@H]3Br |
| 1033 | RC005 | (3R,7S,8S,11R,12S,15S)-7,11-dibromo-12,21- dihydroxy-8,12-dimethyl-4-methylidene-15-(prop-1- en-2-yl)-16-oxatricyclo[16.3.1.0^{3,8}]docosa- 1(22),18,20-trien-17-one | C=C(C)[C@@H]2CC[C@](C)(O)[C@H](Br)CC[C@@]3(C)[C@H](Cc1cc(ccc1O)C(=O)O2)C(=C)CC[C@@H]3Br |
| 1034 | RC006 | (3R,7S,8S,11S,13S,16S)-7-bromo-22-hydroxy-16-(2- hydroxypropan-2-yl)-8,13-dimethyl-4-methylidene- 12,17- dioxatetracyclo[17.3.1.0^{3,8}.0^{11,13}]tricosa- 1(23),19,21-trien-18-one | CC(C)(O)[C@H]1OC(=O)c4ccc(O)c(C[C@@H]3C(=C)CC[C@H](Br)[C@@]3(C)CC[C@@H]2O[C@@]2(C)CC1)c4 |
| 1035 | RC007 | (7S,8S,11R,12S,15S)-7,11-dibromo-12,15,22- trihydroxy-4,8,12,16,16-pentamethyl-17- oxatricyclo[17.3.1.0^{3,8}]tricosa-1(23),3,19,21- tetraen-18-one | O=C2OC(C)(C)[C@@H](O)CC[C@](C)(O)[C@H](Br)CC[C@@]3(C)C(Cc1cc2ccc1O)=C(C)CC[C@@H]3Br |
| 1036 | RC008 | (3R,7S,8S,11R,12S,15R)-7,11,15-tribromo-12,22- dihydroxy-8,12,16,16-tetramethyl-4-methylidene-17- oxatricyclo[17.3.1.0^{3,8}]tricosa-1(23),19,21- trien-18-one | O=C2OC(C)(C)[C@H](Br)CC[C@](C)(O)[C@H](Br)CC[C@@]3(C)[C@H](Cc1cc2ccc1O)C(=C)CC[C@@H]3Br |
| 1041 | GA008 | 6-bromo-3-(3-bromo-4-hydroxybenzyl)-4-(hydroxymethyl)benzene-1,2-diol | Oc2c(Cc1ccc(O)c(Br)c1)c(cc(Br)c2O)CO |
| 1037 | RC009 | (3R,7S,8S,11R,12S,15S)-7,11-dibromo-12,15,22- trihydroxy-8,12,16,16-tetramethyl-4-methylidene-17- oxatricyclo[17.3.1.0^{3,8}]tricosa-1(23),19,21- trien-18-one | O=C2OC(C)(C)[C@@H](O)CC[C@](C)(O)[C@H](Br)CC[C@@]3(C)[C@H](Cc1cc2ccc1O)C(=C)CC[C@@H]3Br |
| 1038 | RC010 | (7S,8S,11R,12S,15S)-7,11-dibromo-15-(2-bromopropan-2- yl)-12,21-dihydroxy-4,8,12-trimethyl-16- oxatricyclo[16.3.1.0^{3,8}]docosa-1(22),3,18,20- tetraen-17-one | O=C2OC(C)(C)[C@@H](O)CC[C@](C)(O)[C@H](Br)CC[C@@]3(C)[C@H](Cc1cc2ccc1O)C(=C)CC[C@@H]3Br |
| 1039 | RC011 | (7S,8S,11R,12S,15R)-7,11,15-Tribromo-12,22-dihydroxy-4,8,12,16,16-pentamethyl-17-oxatricyclo[17.3.1.03,8]tricosa-1(23),3,19,21-tetraen-18-one | O=C2OC(C)(C)[C@H](Br)CC[C@](C)(O)[C@H](Br)CC[C@@]3(C)C(Cc1cc2ccc1O)=C(C)CC[C@@H]3Br |
| 1040 | RC012 | (8E,11S,13S,16S)-22-hydroxy-16-(2-hydroxypropan-2- yl)-4,8,13-trimethyl-12,17- dioxatetracyclo[17.3.1.0^{3,7}.0^{11,13}]tricosa- 1(23),3,8,19,21-pentaen-18-one | CC(C)(O)[C@H]1OC(=O)c4ccc(O)c(CC3=C(C)CCC3C(C)=CC[C@@H]2O[C@@]2(C)CC1)c4 |
| 1043 | GA010 | 3-bromo-5-(hydroxymethyl)benzene-1,2-diol | Oc1cc(cc(Br)c1O)CO |
| 1044 | GA011 | 6-bromo-3-{3-bromo-5-[5-bromo-2-(3-bromo-4-hydroxybenzyl)-3,4-dihydroxybenzyl]-4-hydroxybenzyl}-4-(methoxymethyl)benzene-1,2-diol | Oc4c(Cc3cc(Cc2cc(Br)c(O)c(O)c2Cc1ccc(O)c(Br)c1)c(O)c(Br)c3)c(cc(Br)c4O)COC |
| 1045 | GA012 | 3-bromo-4-[5-bromo-2-(3-bromo-4-hydroxybenzyl)-3,4-dihydroxybenzyl]-6-(3-bromo-4-hydroxybenzyl)-5-(hydroxymethyl)benzene-1,2-diol | Oc2c(Cc1ccc(O)c(Br)c1)c(cc(Br)c2O)Cc4c(CO)c(Cc3ccc(O)c(Br)c3)c(O)c(O)c4Br |
| 1046 | GU001 | (1R,2R,4aS,8aS)-2,5,5,8a-tetramethyl-1-[(3S)-3-methylpent-4-en-1-yl]-decahydronaphthalen-2-ol | C=C[C@@H](C)CC[C@@H]1[C@@]2(C)CC[C@@H2][C@](C)(C)[C@@H]2CC[C@@]1(C)O |
| 1047 | GU002 | (2S,4aS,5R,6R,8aR)-1,1,4a,6-tetramethyl-5-[(3S)-3-methylpent-4-en-1-yl]-decahydronaphthalene-2,6-diol | C=C[C@@H](C)CC[C@H]2[C@](C)(O)CC[C@@H]1[C@]2(C)CC[C@H](O)[C@@]1(C)C |
| 1048 | GU003 | (1S,2R,4aS,8aS)-2,5,5,8a-tetramethyl-1-[(3S)-3-methylpent-4-en-1-yl]-decahydronaphthalene-1,2-diol | C=C[C@@H](C)CC[C@]1(O)[C@@]2(C)CC[C@@H2][C@](C)(C)[C@@H]2CC[C@@]1(C)O |
| 1049 | GU004 | (4aS,5R,6R,8aR)-6-hydroxy-1,1,4a,6-tetramethyl-5-[(3S)-3-methylpent-4-en-1-yl]-decahydronaphthalen-2-one | C=C[C@@H](C)CC[C@@H]1[C@@]2(C)CCC(=O)[C@](C)(C)[C@@H]2CC[C@@]1(C)O |
| 1050 | GU005 | (4aR,5S,6R,8aR)-1,1,4a,6-tetramethyl-5-(3-methylidenepent-4-en-1-yl)-decahydronaphthalen-2-one | C=C(C=C)CC[C@H]1[C@H](C)CC[C@H]2[C@@](C)(C)C(=O)CC[C@]12C |
| 1051 | GU006 | (4aR,5S,6R,8aS)-1,1,4a,6-tetramethyl-5-(3-methylidenepent-4-en-1-yl)-decahydronaphthalene | C=C(C=C)CC[C@H]1[C@H](C)CC[C@H]2[C@@](C)(C)[C@@H2]CC[C@]12C |
| 1052 | GU007 | (2R,4aR,5S,6R,8aR)-1,1,4a,6-tetramethyl-5-(3-methylidenepent-4-en-1-yl)-decahydronaphthalen-2-ol | C=C(C=C)CC[C@H]2[C@H](C)CC[C@@H]1[C@]2(C)CC[C@@H](O)[C@@]1(C)C |
| 1053 | GU008 | (1S,3aS,4R,8R,8aS)-8-isopropyl-1,4-dimethyl-3a,5,6,7,8,8a-hexahydro-1H-azulen-4-ol | CC(C)[C@H]1CCC[C@@](C)(O)[C@H]2C=C[C@H](C)[C@@H]12 |
| 1054 | GU009 | [(1S,3aS,4R,8S,8aR)-8-ethyl-1,4-dimethyl-1,3a,4,5,6,7,8,8a-octahydroazulen-4-yl]methanol | C[C@H]2C=C[C@H]1[C@@H]2[C@H](CCC[C@@]1(C)CO)CC |
| 1055 | GU010 | [(1S,3aS,4R,8S,8aR)-8-ethyl-1,4-dimethyl-1,3a,4,5,6,7,8,8a-octahydroazulen-4-yl]methyl acetate | CC(=O)OC[C@]2(C)CCC[C@H](CC)[C@H]1[C@@H]2C=C[C@@H]1C |
| 1056 | RC013 | 7-bromo-22-hydroxy-8,13-dimethyl-4-methylidene-16- (prop-1-en-2-yl)-12,17-dioxatetracyclo[17.3.1.0^{3,8}.0^{11,13}]tricosa- 1(23),19,21-trien-18-one | C=C(C)[C@H]1OC(=O)c4ccc(O)c(C[C@@H]3C(=C)CC[C@H](Br)[C@@]3(C)CC[C@@H]2O[C@@]2(C)CC1)c4 |
| 1057 | RC014 | 7-bromo-16-(2-bromopropan-2-yl)-22-hydroxy-8,13- dimethyl-4-methylidene-12,17- dioxatetracyclo[17.3.1.0^{3,8}.0^{11,13}]tricosa- 1(23),19,21-trien-18-one | BrC(C)(C)[C@H]1OC(=O)c4ccc(O)c(C[C@@H]3C(=C)CC[C@H](Br)[C@@]3(C)CC[C@@H]2O[C@@]2(C)CC1)c4 |
| 1058 | RC015 | (7S,8S,11S,13S,16R)-7,16-dibromo-23-hydroxy- 4,8,13,17,17-pentamethyl-12,18- dioxatetracyclo[18.3.1.0^{3,8}.0^{11,13}]tetracosa- 1(24),3,20,22-tetraen-19-one | O=C2OC(C)(C)[C@H](Br)CC[C@]4(C)O[C@H]4CC[C@@]3(C)C(Cc1cc2ccc1O)=C(C)CC[C@@H]3Br |
| 1059 | RC016 | 1S,10R,14S,15S,18R,19)-14,18-dibromo-7-hydroxy- 11,15,19,21,21-pentamethyl-2,20- dioxatetracyclo[17.2.2.1^{4,8}.0^{10,15}]tetracosa- 4(24),5,7,11-tetraen-3-one | CC4(C)O[C@]1(C)CC[C@@H]4OC(=O)c3cc(C[C@@H]2C(C)=CC[C@H](Br)[C@@]2(C)CC[C@H]1Br)c(O)cc3 |
| 1060 | RC033 | (3S,8R,11R,12S,15S)-11-bromo-15-(2-bromopropan-2- yl)-12,21-dihydroxy-4-methoxy-4,8,12-trimethyl-16- oxatetracyclo[16.3.1.0^{3,8}.0^{5,7}]docosa- 1(22),18,20-trien-17-one | BrC(C)(C)[C@@H]3CC[C@](C)(O)[C@H](Br)CC[C@]4(C)C1CC1C(C)(OC)[C@H]4Cc2cc(ccc2O)C(=O)O3 |
| 1061 | RC034 | (11R,12S,15S)-11-bromo-12,21-dihydroxy-15-(2- hydroxypropan-2-yl)-4,12-dimethyl-7-methylidene-16- oxatricyclo[16.3.1.0^{3,8}]docosa-1(22),3,18,20- tetraen-17-one | CC(C)(O)[C@@H]2CC[C@](C)(O)[C@H](Br)CCC3C(=C)CCC(C)=C3Cc1cc(ccc1O)C(=O)O2 |
| 1062 | RC035 | (11R,12S,15S)-11-bromo-12,21-dihydroxy-4,7,12- trimethyl-15-(prop-1-en-2-yl)-16- oxatricyclo[16.3.1.0^{3,8}]docosa-1(22),3,6,18,20- pentaen-17-one | C=C(C)[C@@H]2CC[C@](C)(O)[C@H](Br)CCC3C(C)=CCC(C)=C3Cc1cc(ccc1O)C(=O)O2 |
| 1063 | RC036 | (7S,8S,11R,12S,15S)-7,11-dibromo-12,21-dihydroxy- 4,8,12-trimethyl-15-(prop-1-en-2-yl)-16- oxatricyclo[16.3.1.0^{3,8}]docosa-1(22),3,18,20- tetraen-17-one | C=C(C)[C@@H]2CC[C@](C)(O)[C@H](Br)CC[C@@]3(C)C(Cc1cc(ccc1O)C(=O)O2)=C(C)CC[C@@H]3Br |
| 1064 | RC037 | (3R,7S,8S,11R,12S,15S)-7,11-dibromo-12,21- dihydroxy-4,8,12-trimethyl-15-(prop-1-en-2-yl)-16- oxatricyclo[16.3.1.0^{3,8}]docosa-1(22),4,18,20- tetraen-17-one | C=C(C)[C@@H]2CC[C@](C)(O)[C@H](Br)CC[C@@]3(C)[C@H](Cc1cc(ccc1O)C(=O)O2)C(C)=CC[C@@H]3Br |
| 1065 | RC038 | (3R,7S,8S,11R,12S,15S)-7,11-dibromo-15-(2- bromopropan-2-yl)-12,21-dihydroxy-4,8,12-trimethyl- 16-oxatricyclo[16.3.1.0^{3,8}]docosa-1(22),4,18,20- tetraen-17-one | BrC(C)(C)[C@@H]2CC[C@](C)(O)[C@H](Br)CC[C@@]3(C)[C@H](Cc1cc(ccc1O)C(=O)O2)C(C)=CC[C@@H]3Br |
| 1066 | RC039 | (1S,10R,14S,15S,18R,19R)-14,18-dibromo-7-hydroxy- 15,19,21,21-tetramethyl-11-methylidene-2,20- dioxatetracyclo[17.2.2.1^{4,8}.0^{10,15}]tetracosa- 4,6,8(24)-trien-3-one | CC4(C)O[C@]1(C)CC[C@@H]4OC(=O)c3cc(C[C@@H]2C(=C)CC[C@H](Br)[C@@]2(C)CC[C@H]1Br)c(O)cc3 |
| 1067 | RC021 | (1R,14S,15S)-14-bromo-15-[(3E)-4,8-dimethylnona- 3,7-dien-1-yl]-15-methyl-9- oxatricyclo[9.4.0.0^{3,8}]pentadeca-3,5,7,11- tetraene-5-carboxylic acid | O=C(O)c2cc3C[C@@H]1C(=CC[C@H](Br)[C@@]1(C)CC\C=C(/C)CC\C=C(/C)C)COc3cc2 |
| 1068 | RC022 | (1R,14S,15S)-14-bromo-15-[(3E)-4,8-dimethylnona- 3,7-dien-1-yl]-15-methyl-9- oxatricyclo[9.4.0.0^{3,8}]pentadeca-3,5,7,10- tetraene-5-carboxylic acid | O=C(O)c2cc3C[C@@H]1C(CC[C@H](Br)[C@@]1(C)CC\C=C(/C)CC\C=C(/C)C)=COc3cc2 |
| 1069 | RC023 | 14-bromo-15-[2-(3-bromo-6-hydroxy-2,2,6- trimethylcyclohexyl)ethyl]-15-methyl-9- oxatricyclo[9.4.0.0^{3,8}]pentadeca-3,5,7,11- tetraene-5-carboxylic acid | O=C(O)c3cc4C[C@@H]2C(=CC[C@H](Br)[C@@]2(C)CC[C@H]1[C@@](C)(C)[C@@H](Br)CC[C@]1(C)O)COc4cc3 |
| 1070 | RC024 | 14-bromo-15-[2-(3-bromo-6-hydroxy-2,2,6- trimethylcyclohexyl)ethyl]-15-methyl-9- oxatricyclo[9.4.0.0^{3,8}]pentadeca-3,5,7,10- tetraene-5-carboxylic acid | O=C(O)c3cc4C[C@@H]2C(CC[C@H](Br)[C@@]2(C)CC[C@H]1[C@@](C)(C)[C@@H](Br)CC[C@]1(C)O)=COc4cc3 |
| 1071 | RC025 | 14-bromo-15-[2-(3-bromo-2,2-dimethyl-6- methylidenecyclohexyl)ethyl]-15-methyl-9- oxatricyclo[9.4.0.0^{3,8}]pentadeca-3,5,7,10- tetraene-5-carboxylic acid | O=C(O)c3cc4C[C@@H]2C(CC[C@H](Br)[C@@]2(C)CC[C@@H]1C(=C)CC[C@H](Br)[C@@]1(C)C)=COc4cc3 |
| 1072 | RC026 | 3-[(3E,7E)-4,8,12-trimethyltrideca-3,7,11-trien-1- yl]-2,5-dihydro-1-benzoxepine-7-carboxylic acid | O=C(O)c1ccc2OCC(=CCc2c1)CC\C=C(/C)CC\C=C(/C)CC\C=C(/C)C |
| 1073 | RC027 | 3-{[(4aS,5R,6R,8aS)-6-bromo-5,8a-dimethyl-2- methylidene-5-(4-methylpent-3-en-1-yl)- decahydronaphthalen-1-yl]methyl}-4-hydroxybenzoic acid | O=C(O)c3cc(CC2C(=C)CC[C@H]1[C@@]2(C)CC[C@@H](Br)[C@]1(C)CC\C=C(/C)C)c(O)cc3 |
| 1074 | RC028 | 3-{[(4aS,5R,6R,8aS)-6-bromo-5,8a-dimethyl-2- methylidene-5-(4-methylpent-3-en-1-yl)- decahydronaphthalen-1-yl]methyl}-5-bromo-4- hydroxybenzoic acid | O=C(O)c3cc(CC2C(=C)CC[C@H]1[C@@]2(C)CC[C@@H](Br)[C@]1(C)CC\C=C(/C)C)c(O)c(Br)c3 |
| 1075 | RC029 | 2-{[(4aS,5R,6R,8aS)-6-bromo-5-(3-bromo-4-chloro-4- methylpentyl)-5,8a-dimethyl-2-methylidene- decahydronaphthalen-1-yl]methyl}-4,6-dibromophenol | Brc3cc(CC2C(=C)CC[C@H]1[C@@]2(C)CC[C@@H](Br)[C@]1(C)CCC(Br)C(C)(C)Cl)c(O)c(Br)c3 |
| 1076 | RC030 | 2-{[(4aS,5R,6R,8aS)-6-bromo-5-(4-bromo-3-chloro-4- methylpentyl)-5,8a-dimethyl-2-methylidene- decahydronaphthalen-1-yl]methyl}-4,6-dibromophenol | BrC(C)(C)C(Cl)CC[C@@]3(C)[C@H](Br)CC[C@@]2(C)C(Cc1cc(Br)cc(Br)c1O)C(=C)CC[C@@H]23 |
| 1077 | RC040 | (1S,14S,15S,18R,19R)-14,18-dibromo-7-hydroxy- 11,15,19,21,21-pentamethyl-2,20- dioxatetracyclo[17.2.2.1^{4,8}.0^{10,15}]tetracosa- 4,6,8(24),10-tetraen-3-one | CC4(C)O[C@]1(C)CC[C@@H]4OC(=O)c3cc(CC2=C(C)CC[C@H](Br)[C@@]2(C)CC[C@H]1Br)c(O)cc3 |
| 1078 | BS075 | (2Z,6E)-2-(3,4-dihydroxy-4-methylpentyl)-9-(6- hydroxy-2,8-dimethyl-2H-chromen-2-yl)-6-methylnona- 2,6-dienoic acid | Oc1c(C)cc(O)cc1C\C=C(/C)CC\C=C(/C)CC\C=C(\CC\C=C(/C)C)C(=O)O |
| 1079 | BS076 | (2E,6E,10E)-6,10-dimethyl-12-(5-methyl-3,6-dioxocyclohexa-1,4-dien-1-yl)-2-(4-methylpent-3-enyl)dodeca-2,6,10-trienoic acid | CC1=CC(=O)C=C(C1=O)CC=C(C)CCC=C(C)CCC=C(CCC=C(C)C)C(=O)O |
| 1080 | BS077 | (2Z,6E)-9-(6-hydroxy-2,8-dimethyl-2H-chromen-2-yl)-6- methyl-2-(4-methylpent-3-en-1-yl)nona-2,6-dienoic acid | O=C(O)C(/CC\C=C(/C)C)=C\CCC(\C)=C\CCC1(C)C=Cc2cc(O)cc(C)C2o1 |
| 1081 | BS078 | (2Z,6E)-2-(3,4-dihydroxy-4-methylpentyl)-9-(6- hydroxy-2,8-dimethyl-2H-chromen-2-yl)-6-methylnona- 2,6-dienoic acid | CC(C)(O)C(O)CC\C(=C\CCC(\C)=C\CCC1(C)C=Cc2cc(O)cc(C)c2O1)C(=O)O |
| 1082 | BS079 | (2E,6E)-2-(3,4-dihydroxy-4-methylpentyl)-7-[2- (furan-3-yl)ethyl]octa-2,6-dienedioic acid | CC(C)(O)C(O)CC/C(=C\CC\C=C(/CCc1ccoc1)C(=O)O)C(=O)O |
| 1083 | BS080 | methyl 2-(5-hydroxy-2-{[(6E)-13-hydroxy-3,7,11,15- tetramethyl-12-oxohexadeca-1,6,14-trien-3- yl]oxy}phenyl)acetate | C\C(C)=C/C(O)C(=O)C(C)CCCC(\C)=C\CCC(C)(Oc1ccc(O)cc1CC(=O)OC)C=C |
| 1084 | BS081 | methyl 2-(5-hydroxy-2-{[(6E)-13-hydroxy-3,7,11,15- tetramethyl-12-oxohexadeca-1,6,14-trien-3- yl]oxy}phenyl)acetate | C\C(C)=C/C(O)[C@H](O)C(\C)=C\CCC(\C)=C\CCC(C)(Oc1ccc(O)cc1CC(=O)OC)C=C |
| 1085 | BS082 | methyl 2-(2-{[(6E,10E,12R)-12,13-dihydroxy- 3,7,11,15-tetramethylhexadeca-1,6,10,14-tetraen-3- yl]oxy}-5-hydroxyphenyl)acetate | C\C(C)=C/C(O)[C@H](O)C(\C)=C\CCC(\C)=C\CCC(C)(Oc1ccc(O)cc1CC(=O)OC)C=C |
| 1086 | BS083 | methyl 2-(2-{[(6E,10E,12R)-12,13-dihydroxy- 3,7,11,15-tetramethylhexadeca-1,6,10,14-tetraen-3- yl]oxy}-5-hydroxyphenyl)acetate | C\C(C)=C/C(O)[C@H](O)C(\C)=C\CCC(\C)=C\CCC(C)(Oc1ccc(O)cc1CC(=O)OC)C=C |
| 1087 | BS084 | methyl 2-(5-hydroxy-2-{[(6E,12R)-12-hydroxy- 3,7,11,15-tetramethyl-13-oxohexadeca-1,6,14-trien-3- yl]oxy}phenyl)acetate | C\C(C)=C/C(=O)[C@H](O)C(C)CCCC(\C)=C\CCC(C)(Oc1ccc(O)cc1CC(=O)OC)C=C |
| 1088 | BS085 | methyl 2-(5-hydroxy-2-{[(6E)-12-hydroxy-3,7,11,15- tetramethyl-13-oxohexadeca-1,6,14-trien-3- yl]oxy}phenyl)acetate | C\C(C)=C/C(=O)C(O)C(C)CCCC(\C)=C\CCC(C)(Oc1ccc(O)cc1CC(=O)OC)C=C |
| 1089 | BS086 | methyl 2-(5-hydroxy-2-{[(6E,10E,13S)-13-hydroxy- 3,7,11,15-tetramethyl-12-oxohexadeca-1,6,10,14- tetraen-3-yl]oxy}phenyl)acetate | C/C(C)=C/[C@H](O)C(=O)C(\C)=C\CCC(\C)=C\CCC(C)(Oc1ccc(O)cc1CC(=O)OC)C=C |
| 1090 | BS087 | methyl 2-(5-hydroxy-2-{[(6E,10E,12S)-12-hydroxy- 3,7,11,15-tetramethyl-13-oxohexadeca-1,6,10,14- tetraen-3-yl]oxy}phenyl)acetate | C\C(C)=C/C(=O)[C@@H](O)C(\C)=C\CCC(\C)=C\CCC(C)(Oc1ccc(O)cc1CC(=O)OC)C=C |
| 1091 | BS088 | methyl 2-(5-hydroxy-2-{[(6E,13E)-12-hydroxy- 3,7,11,15-tetramethylhexadeca-1,6,13,15-tetraen-3- yl]oxy}phenyl)acetate | C=C(C)/C=C/C(O)C(C)CCCC(\C)=C\CCC(C)(Oc1ccc(O)cc1CC(=O)OC)C=C |
| 1092 | BS089 | methyl 2-(2-{[(6E)-3,7-dimethyl-8-[(1R,5R)-3- methyl-5-(2-methylprop-1-en-1-yl)-4-oxocyclopent-2- en-1-yl]octa-1,6-dien-3-yl]oxy}-5- hydroxyphenyl)acetate | C/C(C[C@@H]1C=C(C)C(=O)[C@H]1/C=C(\C)C)=C\CCC(C)(C=C)Oc2ccc(O)cc2CC(=O)OC |
| 1093 | BS090 | methyl 2-{3-[(2E,6E,10E,12R,13S)-12,13-dihydroxy- 3,7,11,15-tetramethylhexadeca-2,6,10,14-tetraen-1- yl]-2,5-dihydroxyphenyl}acetate | Oc1c(cc(O)cc1CC(=O)OC)C\C=C(/C)CC\C=C(/C)CC\C=C(/C)[C@@H](O)[C@@H](O)/C=C(/C)C |
| 1094 | BS091 | methyl 2-{2,5-dihydroxy-3-[(2E,6E,10E,13S)-13- hydroxy-3,7,11,15-tetramethyl-12-oxohexadeca- 2,6,10,14-tetraen-1-yl]phenyl}acetate | Oc1c(cc(O)cc1CC(=O)OC)C\C=C(/C)CC\C=C(/C)CC\C=C(/C)C(=O)[C@@H](O)/C=C(/C)C |
| 1095 | BS092 | methyl 2-{2,5-dihydroxy-3-[(2E,10E,13S)-13-hydroxy- 3,7,11,15-tetramethyl-6-oxohexadeca-2,10,14-trien-1- yl]phenyl}acetate | Oc1c(cc(O)cc1CC(=O)OC)C\C=C(/C)CCC(=O)C(C)CC\C=C(/C)C[C@H](O)/C=C(/C)C |
| 1096 | BS093 | (2Z,6E,10E)-12-(2,5-dihydroxy-3-methylphenyl)-6,10- dimethyl-2-(4-methylpent-3-en-1-yl)dodeca-2,6,10- trienoic acid | Oc1c(C)cc(O)cc1C\C=C(/C)CC\C=C(/C)CC\C=C(\CC\C=C(/C)C)C(=O)O |
| 1097 | BS094 | (6E,10E)-16-(2,5-Dihydroxy-3-methylphenyl)-4,14-dihydroxy-2,6,10,14-tetramethyl-2,6,10-hexadecatrien-5-one | Oc1c(C)cc(O)cc1CCC(C)(O)CC\C=C(/C)CC\C=C(/C)C(=O)C(O)/C=C(\C)C |
| 1098 | GU011 | 2,5,5-trimethyl-4-(4-methylpent-3-en-1-yl)cyclohex-2- en-1-ol | CC1(C)CC(O)C(C)=CC1CC\C=C(/C)C |
| 1099 | GU012 | 3,4,5,5-tetramethyl-4-(3-methylbutyl)cyclohex-2-en-1- ol | CC1(C)CC(O)C=C(C)C1(C)CCC(C)C |
| 1100 | GU013 | 1,5,5,6-tetramethyl-6-(3-methylbutyl)cyclohex-1- ene | CC1(C)CCC=C(C)C1(C)CCC(C)C |
| 1101 | GU014 | 3,4,5,5-tetramethyl-4-(3-oxopentyl)cyclohex-2-en-1- one | CC1(C)CC(=O)C=C(C)C1(C)CCC(=O)CC |
| 1102 | RP079 | 1,4-dibromo-5-chloro-2-[(E)-2-chloroethenyl]-1,5- dimethylcyclohexane | C[C@@]1(Cl)C[C@](Br)(C)[C@@H](CC1Br)\C=C\Cl |
| 1103 | RP080 | (3Z,5R,6R,7E)-5,8-dibromo-2,6-dichloro-2,6- dimethylocta-3,7-dienal | CC(Cl)(\C=C/[C@@H](Br)[C@](C)(Cl)\C=C\Br)C=O |
| 1104 | RP081 | (3E)-1,6-dibromo-2,7-dichloro-3,7-dimethyloct-3- ene | CC(C)(Cl)C(Br)C\C=C(/C)C(Cl)CBr |
| 1105 | RP082 | (3E)-1,6-dibromo-2,7-dichloro-3,7-dimethyloct-3- ene | C[C@@]1(Cl)CC(C)=C(C[C@@H]1Br)\C=C\Cl |
| 1106 | RP083 | 2-bromo-1-chloro-4-[(E)-2-chloroethenyl]-1-methyl-5- methylidenecyclohexane | C[C@@]1(Cl)CC(=C)[C@@H](C[C@@H]1Br)\C=C\Cl |
| 1107 | RP084 | 2,4-dibromo-1-chloro-5-[(E)-2-chloroethenyl]-1,5- dimethylcyclohexane | C[C@@]1(Cl)C[C@](C)(\C=C\Cl)[C@@H](Br)C[C@@H]1Br |
| 1108 | RP085 | 1-[(E)-2-chloroethenyl]-2,4-dimethylbenzene | Cl\C=C\c1ccc(C)cc1C |
| 1109 | RP086 | (1E,3E,5S,6R)-1,5,6-trichloro-2-(dichloromethyl)-6- methylocta-1,3,7-triene | Cl\C=C(/C=C/[C@H](Cl)[C@](C)(Cl)C=C)C(Cl)Cl |
| 1110 | RL488 | 4-bromo-2-[(1R,2S)-1,2-dimethyl-3-methylidenecyclopentyl]-5-methylphenol | C[C@]1(CCC(=C)[C@@H]1C)c2cc(Br)c(C)cc2O |
