# Supplementary material for: Computational study of potential inhibitors for fat mass and obesity-associated protein from seaweed and plant compounds
Source: PeerJ. 2022 Oct 21;10:e14256. doi: 10.7717/peerj.14256 (PMC9590420; doi:10.7717/peerj.14256)
Supplement: Supplemental Information 3 [file peerj-10-14256-s003.docx]

| S.No. | Compound ID | Compound Name | Binding Energy (kcal/mol) |
| --- | --- | --- | --- |
| 1 | BD071 | Cyclozonarone | -9.8 |
| 2 | BS067 | Epitaondiol diacetate | -9.8 |
| 3 | BT002 | Atomarianones A | -9.8 |
| 4 | BS031 | Taondiol | -9.7 |
| 5 | BS055 | Epitaondiol | -9.7 |
| 6 | BS056 | Epitaondiol diacetate | -9.7 |
| 7 | BS057 | Epitaondiol monoacetate | -9.6 |
| 8 | BE011 | Dieckol | -9.5 |
| 9 | BE004 | Phlorofucofuroeckol A | -9.4 |
| 10 | RL312 | 3-bromobarekoxide | -9.4 |
| 11 | BC011 | 2-[(2E)-3-[(1S,4'R,4aS,7aS)-4'-methoxy-4a,5',5',7a-tetramethyl-5,6,7,7a-tetrahydro-4aH-spiro[cyclopenta[c]pyran-1,2'-oxolane]-3-ylmethyl]but-2-en-1-yl]-4-methoxy-6-methylphenol | -9.3 |
| 12 | BE015 | Phlorofucofuroeckol A | -9.3 |
| 13 | BS023 | Flabellinone | -9.3 |
| 14 | RL047 | 3-bromobarekoxide | -9.3 |
| 15 | BD068 | Chromazonarol | -9.2 |
| 16 | BE005 | Dieckol | -9.2 |
| 17 | BE016 | Dieckol | -9.2 |
| 18 | BS032 | Isoepitaondiol | -9.2 |
| 19 | BS034 | Isotaondiol | -9.2 |
| 20 | BT003 | Atomarianones B | -9.2 |
| 21 | comp57 | Punicalagin | -9.2 |
| 22 | comp54 | Cyanidine-3-Oglucoside | -9.2 |
| 23 | RL048 | Barekoxide | -9.2 |
| 24 | BC013 | (1R,4aS,7aS)-3-[(6-methoxy-2,8-dimethyl-3,4-dihydro-2H-1-benzopyran-2-yl) methyl]-4a,5',5',7a-tetramethyl-5,6,7,7a-tetrahydro-4aH,5'H-spiro[cyclopenta[c]pyran-1,2'-furan] | -9.1 |
| 25 | BT007 | 24-ethylcholesta-4,24(28)-dien-3-one | -9.1 |
| 26 | comp39 | Celastrol | -9.1 |
| 27 | BE012 | 6,6'-Bieckoll | -9 |
| 28 | BS033 | Epitaondiol | -9 |
| 29 | RC034 | Bromophycolide K | -9 |
| 30 | RG001 | Desmosterol | -9 |
| 31 | RJ008 | 16β-Hydroxy-5α-cholestane-3,6-dione | -9 |
| 32 | RL335 | Vinyl bromide | -9 |
| 33 | BE009 | Phlorofucofuroeckol A | -8.9 |
| 34 | BS022 | Flabellinol | -8.9 |
| 35 | BS061 | Fucosterol | -8.9 |
| 36 | comp64 | Exemestane | -8.9 |
| 37 | comp7 | Punicalin | -8.9 |
| 38 | RC039 | Bromophycolide P | -8.9 |
| 39 | BC004 | Amentol 1'-methyl ether | -8.8 |
| 40 | BC014 | (1S,4'R,4aS,7aS)-3-[(6-methoxy-2,8-dimethyl-3,4-dihydro-2H-1-benzopyran-2-yl) methyl]-4a,5',5',7a-tetramethyl-5,6,7,7a-tetrahydro-4aH-spiro[cyclopenta[c]pyran-1,2'-oxolane]-4'-ol | -8.8 |
| 41 | BC017 | Claraenone | -8.8 |
| 42 | BD009 | Fucoxanthin | -8.8 |
| 43 | BS020 | (2R,6R,14R)-6-hydroxy-2,14-dimethyl-13-[(5E)-5- (propan-2-yl) hept-5-en-2-yl]-5- oxatetracyclo[7.7.0.0^{2,6}.0^{10,14}]hexadecan-4- one | -8.8 |
| 44 | BS021 | 2β,3α-epitaondiol | -8.8 |
| 45 | BT006 | 29-hydroperoxystigmasta-5,24(28)-dien-3β-ol | -8.8 |
| 46 | BT011 | 6β-hydroxy-24-ethylcholesta-4,24(28)-dien-3-one | -8.8 |
| 47 | comp26 | Beta rosasterol | -8.8 |
| 48 | comp50 | Corilagin | -8.8 |
| 49 | comp63 | Evodiamine | -8.8 |
| 50 | RC016 | Bromophycolide U | -8.8 |
| 51 | RG005 | Cholesta-5,25-diene-3β,24ε-diol | -8.8 |
| 52 | RG009 | 25-hydroperoxy-6β-hydroxycholesta-4,23(E)-dien-3-one | -8.8 |
| 53 | RG011 | 25-hydroperoxycholesta-4,23(E)-diene-3,6-dione | -8.8 |
| 54 | RG013 | 6β,25-Dihydroxycholesta-4,23-dien-3-one | -8.8 |
| 55 | RL065 | Callicladol | -8.8 |
| 56 | RL474 | Thyrsiferyl 23-acetate | -8.8 |
| 57 | BS062 | Saringosterone | -8.7 |
| 58 | BS064 | Fucoxanthin | -8.7 |
| 59 | comp97 | Platycodins | -8.7 |
| 60 | RC007 | Bromophycolide G | -8.7 |
| 61 | RG012 | 6β,24ε-Dihydroxycholesta-4,25-dien-3-one | -8.7 |
| 62 | RL067 | Enshuol | -8.7 |
| 63 | BC005 | Amentol | -8.6 |
| 64 | BC008 | 14-methoxyamentol quinone | -8.6 |
| 65 | BE002 | Eckstolonol | -8.6 |
| 66 | BE013 | Eckstolon | -8.6 |
| 67 | BS019 | (2R,5S,14R,15R)-14-[(2R,4Z)-6-hydroxy-5-(propan-2- yl) hept-4-en-2-yl]-2,15- dimethyltetracyclo[8.7.0.0^{2,7}.0^{11,15}]heptadec-7- en-5-ol | -8.6 |
| 68 | BS063 | Saringosterol | -8.6 |
| 69 | BS068 | Isoepitaondiol diacetate | -8.6 |
| 70 | BT004 | Fucosterol | -8.6 |
| 71 | BU006 | Fucoxanthin | -8.6 |
| 72 | GA003 | Siphonaxanthin | -8.6 |
| 73 | comp77 | Luteolin 4-O-glucopyranoside | -8.6 |
| 74 | RG004 | 25-hydroperoxycholesta-5,23(E)-dien-3β-ol | -8.6 |
| 75 | BS028 | (-)-Epistypodiol | -8.5 |
| 76 | BT012 | 24ε-hydroperoxy-6β-hydroxy-24-ethylcholesta-4, - 28(29)-dien-3-one | -8.5 |
| 77 | RC008 | Bromophycolide H | -8.5 |
| 78 | RC009 | Bromophycolide I | -8.5 |
| 79 | RC010 | Bromophycolide A | -8.5 |
| 80 | RG008 | 24ε-hydroperoxy-6β-hydroxycholesta-4,25-dien-3-one | -8.5 |
| 81 | RG014 | Cholesta-4,25-diene-3,6,24-trione | -8.5 |
| 82 | RL131 | Isodehydrothyrsiferol | -8.5 |
| 83 | RL133 | 10-Epi-15, 16-dehydrothyrsiferol | -8.5 |
| 84 | RL473 | Thyrsiferol | -8.5 |
| 85 | RL487 | 16-Epihydroxydehydrothysiferol | -8.5 |
| 86 | BC009 | 1'-methoxycystoketal | -8.4 |
| 87 | BS059 | Ketostypodiol diacetate | -8.4 |
| 88 | BT008 | 24ε-hydroperoxy-24-ethylcholesta-4,28(29)-dien-3-one | -8.4 |
| 89 | comp100 | Protopanaxadiol | -8.4 |
| 90 | RC003 | Bromophycolide C | -8.4 |
| 91 | RG006 | cholesta-5,23(E)-dien-3β,25-diol | -8.4 |
| 92 | RG007 | 24(R/S)-Epoxy-6β-hydroxycholest-4-en-3-one | -8.4 |
| 93 | RL056 | Laukarlaol | -8.4 |
| 94 | RL078 | Laurenmariannol | -8.4 |
| 95 | RL082 | Enshuol | -8.4 |
| 96 | RL128 | Dehydrovenustratriol | -8.4 |
| 97 | RL129 | 15, 16-Dehydrovenustatriol | -8.4 |
| 98 | RL134 | Thyrsenol A | -8.4 |
| 99 | RL150 | Triquinanes | -8.4 |
| 100 | RL325 | Thyrsiferyl acetate | -8.4 |
| 101 | RL326 | Thyrsiferol acetate | -8.4 |
| 102 | RL512 | Dehydrothyrsiferol | -8.4 |
| 103 | RL550 | Cholesterol | -8.4 |
| 104 | BC015 | (7S,11S,12S)-Cystoketal | -8.3 |
| 105 | BD069 | Zonarone | -8.3 |
| 106 | BS024 | Stypotriolaldehyde | -8.3 |
| 107 | BS026 | Stypoldione | -8.3 |
| 108 | BS044 | Fallachromenoic acid | -8.3 |
| 109 | BS072 | 14-ketostypotriol diacetate | -8.3 |
| 110 | BT009 | 24-ethylcholesta-4,24(28)-dien-3,6-dione | -8.3 |
| 111 | comp16 | Annonaine | -8.3 |
| 112 | comp99 | Rutin | -8.3 |
| 113 | RC037 | Bromophycolide N | -8.3 |
| 114 | RC040 | Bromophycolide Q | -8.3 |
| 115 | RL080 | Thyrsiferol | -8.3 |
| 116 | RL126 | Venustatriol | -8.3 |
| 117 | RL135 | Thyrsenol B | -8.3 |
| 118 | RL136 | 10-Epidehythysiferol | -8.3 |
| 119 | RL284 | Prevezol E | -8.3 |
| 120 | RL324 | Thyrsiferol | -8.3 |
| 121 | RL515 | 3-Hydroxyprethyrsenol A | -8.3 |
| 122 | RL551 | Cholest-5-en-3β, 7α-diol | -8.3 |
| 123 | BC006 | 1'-4'-dimethoxyamentol | -8.2 |
| 124 | BC007 | (1R,4'S,4aR,7aR)-3-[(2E)-4-[2,5-bis(acetyloxy)-3-methylphenyl]-2-methylbut-2-en-1-yl]-4a,5',5',7a-tetramethyl-5,6,7,7a-tetrahydro-4aH-spiro[cyclopenta[c]pyran-1,2'-oxolane]-4'-yl acetate | -8.2 |
| 125 | BC012 | 1',4',14-trimethoxyamentol | -8.2 |
| 126 | BE010 | 7-Phloro eckol | -8.2 |
| 127 | BP001 | Oxysterol | -8.2 |
| 128 | GC005 | Siphonaxanthin | -8.2 |
| 129 | comp27 | Beta sitosterol | -8.2 |
| 130 | RC027 | Callophycoic acid G | -8.2 |
| 131 | RC035 | Bromophycolide L | -8.2 |
| 132 | RL127 | Dehydrothyrsiferol | -8.2 |
| 133 | RL287 | O,15-cyclo-14-bromo-14,15-dihydrorogiol-3,11-diol | -8.2 |
| 134 | RL315 | (-)-Paniculatol | -8.2 |
| 135 | BS029 | Stypodiol | -8.1 |
| 136 | BS049 | Sargaquinone | -8.1 |
| 137 | BS060 | Stypodiol | -8.1 |
| 138 | BS069 | Stypodiol diacetate | -8.1 |
| 139 | GA012 | Isorawsonol | -8.1 |
| 140 | GC004 | 4-Isocymobarbatol | -8.1 |
| 141 | comp25 | Asimilobine | -8.1 |
| 142 | RC012 | Debromophycolide A | -8.1 |
| 143 | RC015 | Bromophycolide T | -8.1 |
| 144 | RC023 | Callophycoic acid C | -8.1 |
| 145 | RC026 | Callophycoic acid F | -8.1 |
| 146 | RG010 | 24ε-hydroperoxycholesta-4,25-diene-3,6-dione | -8.1 |
| 147 | RL008 | Dibromo-phenol | -8.1 |
| 148 | RL109 | Thyrsiferol | -8.1 |
| 149 | RL125 | Thyrsiferol | -8.1 |
| 150 | RL330 | Venustatriol | -8.1 |
| 151 | RL486 | Dioxepandehydrothysiferol | -8.1 |
| 152 | BC003 | 4'-methoxy amentol | -8 |
| 153 | BD067 | Isozonarol | -8 |
| 154 | BD072 | 9-hydroxydolabelladien-6-one | -8 |
| 155 | BS025 | Stypohydroperoxide | -8 |
| 156 | BS030 | Stypotriol | -8 |
| 157 | BT005 | 24ε-hydroperoxy-24-vinylcholesterol | -8 |
| 158 | RC005 | Bromophycolide E | -8 |
| 159 | RC013 | Bromophycolide R | -8 |
| 160 | RG002 | 24,25-epoxycholesterol | -8 |
| 161 | RL084 | 15,16-anhydrothyrsiferol | -8 |
| 162 | RL427 | (3R,5S,6S,8S,9S,10R,13R)-3-bromo-6-hydroxy-8,13-epoxylabd-14-en-1-one | -8 |
| 163 | RL485 | Pseudodehydrothyrsiferol | -8 |
| 164 | RR020 | 7-[3-bromo-2-(2,3-dibromo-4,5-dihydroxybenzyl)-4,5-dihydroxybenzyl]-3,7-dihydro-1H-purine-2,6-dione | -8 |
| 165 | BC010 | 4'-14-dimethoxyamentol | -7.9 |
| 166 | BD070 | Isozonarone | -7.9 |
| 167 | BS035 | (1S,11R,20R)-6-(acetyloxy)-1,8,11,15,19,19- hexamethyl-10- oxapentacyclo[12.8.0.0^{2,11}.0^{4,9}.0^{15,20}]docosa- 4,6,8-trien-18-yl acetate | -7.9 |
| 168 | BS071 | 4'-Chlorostypotriol triacetate | -7.9 |
| 169 | BS091 | Methyl {2,5-dihydroxy-3-[(2E,6E,10E,13S)-13-hydroxy-3,7,11,15-tetramethyl-12-oxo-2,6,10,14-hexadecatetraen-1-yl] phenyl} acetate | -7.9 |
| 170 | BT010 | 24ε-hydroperoxy-24-ethylcholesta-4,28(29)-dien-3,6-dione | -7.9 |
| 171 | GC001 | Debromoisocymobarbatol | -7.9 |
| 172 | comp36 | Carnosol | -7.9 |
| 173 | comp95 | Stigmast-5-en-3-ol | -7.9 |
| 174 | RC006 | Bromophycolide F | -7.9 |
| 175 | RL300 | Labdane type brominated diterpene | -7.9 |
| 176 | RL332 | Thysiferyl-23 acetate | -7.9 |
| 177 | BD061 | Dictyol H | -7.8 |
| 178 | BE008 | Fucodiphloroethol G | -7.8 |
| 179 | comp32 | Campesterol | -7.8 |
| 180 | comp60 | Ellagic acid | -7.8 |
| 181 | comp68 | Gymnemic acids | -7.8 |
| 182 | comp79 | Mangiferin | -7.8 |
| 183 | comp94 | Petunidin-3-Ogluco-side | -7.8 |
| 184 | RC002 | Callophycin A | -7.8 |
| 185 | RC011 | Bromophycolide B | -7.8 |
| 186 | RC014 | Bromophycolide S | -7.8 |
| 187 | RC036 | Bromophycolide M | -7.8 |
| 188 | RC038 | Bromophycolide O | -7.8 |
| 189 | RL331 | Thyrsiferol | -7.8 |
| 190 | RL402 | Isolaurinterol | -7.8 |
| 191 | RL476 | Aplysiadiol | -7.8 |
| 192 | RL532 | Aristolan-8-en-1-one | -7.8 |
| 193 | RR021 | 7-(2,3-dibromo-4,5-dihydroxybenzyl)-3,7-dihydro-1H-purine-2,6-dione | -7.8 |
| 194 | BD043 | (1R,4S,8S,14S)-1,4-Dihydroxy-17-dolastene | -7.7 |
| 195 | BD066 | Zonarol | -7.7 |
| 196 | BS036 | 1S,2S,14S,19R)-8-(acetyloxy)-5-methoxy- 1,6,10,14,18,18- hexamethylpentacyclo[11.8.0.0^{2,10}.0^{4,9}.0^{14,19}]henicosa- 4(9),5,7-trien-17-yl acetate | -7.7 |
| 197 | BS040 | 2-Geranylgeranyl-6-methylbenzoquinone | -7.7 |
| 198 | BS047 | (6E,10E,14E)-16-(2,5-dihydroxy-3-methylphenyl)-2-hydroxy-2,6,10,14-tetramethylhexadeca-6,10,14-trien-3-one | -7.7 |
| 199 | BS058 | Stypotriol triacetate | -7.7 |
| 200 | BS070 | Stypotriol triacetate | -7.7 |
| 201 | BS076 | Sargaquinonic acid | -7.7 |
| 202 | BS078 | Sargathunbergol A | -7.7 |
| 203 | GU005 | ent-Labda-13(16),14-diene-3-one | -7.7 |
| 204 | RC004 | Bromophycolide D | -7.7 |
| 205 | RC022 | Callophycoic acid B | -7.7 |
| 206 | RL322 | Teurilene | -7.7 |
| 207 | RL511 | (3S,4S,6R,9S)-4,9-dibromo-8-chloro-1,5,5,9-tetramethylspiro [5.5] undec-1-en-3-ol | -7.7 |
| 208 | BD008 | 8α,11-dihydroxypachydictyol A | -7.6 |
| 209 | BD034 | Dolabellatrienol | -7.6 |
| 210 | BD040 | Ent-erogorgiaene | -7.6 |
| 211 | BD052 | 4(S*)-Acetoxy-14(S*)-hydroxydolast-1(15), 7, 9-triene | -7.6 |
| 212 | BD063 | 8β-hydroxypachydictyol A | -7.6 |
| 213 | BS050 | Sargaquinoic acid | -7.6 |
| 214 | BS053 | Sargatriol Methyl Ether | -7.6 |
| 215 | GC006 | Siphonein | -7.6 |
| 216 | comp20 | Quercetin 3,30 diglucoside | -7.6 |
| 217 | RL130 | Predehydrovenustatriol acetate | -7.6 |
| 218 | RL248 | Indene-type sesquiterpene | -7.6 |
| 219 | RL261 | Tiomanene | -7.6 |
| 220 | RL301 | Labdane type brominated diterpene | -7.6 |
| 221 | BC016 | Cystalgerone | -7.5 |
| 222 | BD054 | 4(S*), 7(S*)-Diacetoxy-14(S*)-hydroxydolast-1(15), 8-diene | -7.5 |
| 223 | BD056 | Ciliolatale | -7.5 |
| 224 | BD060 | Dictyol C | -7.5 |
| 225 | BE003 | Eckol | -7.5 |
| 226 | BE007 | Eckol | -7.5 |
| 227 | BE014 | Eckol | -7.5 |
| 228 | BS042 | Fallahydroquinone | -7.5 |
| 229 | BS054 | (2E)-5-(6-methoxy-2,8-dimethyl-2H-chromen-2-yl)-2-methylpent-2-enal | -7.5 |
| 230 | GA011 | Rawsonol | -7.5 |
| 231 | GU004 | Labda-14-ene-8α-hydroxy-3-one | -7.5 |
| 232 | GU006 | ent-Labda-13(16),14-diene | -7.5 |
| 233 | comp35 | Carnosic acid | -7.5 |
| 234 | comp72 | Kaempherol | -7.5 |
| 235 | comp28 | Quercetin 3,4' diglucoside | -7.5 |
| 236 | comp40 | Quercetin 3,7 diglucoside | -7.5 |
| 237 | RJ003 | Isoparguerol-7,16-diacetate | -7.5 |
| 238 | RL024 | Filiformin | -7.5 |
| 239 | RL055 | 15-Hydroxypalisadin A | -7.5 |
| 240 | RL083 | Omaezakianol | -7.5 |
| 241 | RL111 | Acetylmajapolene B | -7.5 |
| 242 | RL242 | Irieol C | -7.5 |
| 243 | RL283 | Prevezols D | -7.5 |
| 244 | RL442 | 10-acetoxyangasiol | -7.5 |
| 245 | RR022 | 9-[3-bromo-2-(2,3-dibromo-4,5-dihydroxybenzyl)-4,5-dihydroxybenzyl] adenine | -7.5 |
| 246 | BD013 | Dictyotadimer A | -7.4 |
| 247 | BD019 | Xenicane | -7.4 |
| 248 | BD042 | (1S,4S,8S,14S)-1,4-Dihydroxy-17-dolastene | -7.4 |
| 249 | BD075 | 9-acetoxydolabella-3,7,12-trien-16-al | -7.4 |
| 250 | BD080 | 9-hydroxydolasta-1,3-dien-6-one | -7.4 |
| 251 | BD082 | Isopachydictyol A | -7.4 |
| 252 | BS089 | Methyl [2-({(6E)-3,7-dimethyl-8-[(1R,5R)-3-methyl-5-(2-methyl-1-propen-1-yl)-4-oxo-2-cyclopenten-1-yl]-1,6-octadien-3-yl} oxy)-5-hydroxyphenyl] acetate | -7.4 |
| 253 | GU001 | Labda-14-ene-8-ol | -7.4 |
| 254 | GU002 | Labda-14-ene-3α,8α-diol | -7.4 |
| 255 | comp62 | Epigallocatechin | -7.4 |
| 256 | comp66 | Gallocatechin | -7.4 |
| 257 | comp71 | Isolaureline | -7.4 |
| 258 | comp88 | Normuciferine | -7.4 |
| 259 | RL006 | Bromo Sesquiterpene | -7.4 |
| 260 | RL015 | 5,5'-dibromo-3-[(1R,5S)-1,2-dimethylbicyclo [3.1.0] hex-2-yl]-3'-[(5R)-1,2-dimethylbicyclo [3.1.0] hex-2-yl]-6,6'-dimethylbiphenyl-2,2'-diol | -7.4 |
| 261 | RL079 | (21α)-21-hydroxythyrsiferol | -7.4 |
| 262 | RL188 | Aldingenin B | -7.4 |
| 263 | RL285 | Neorogioldiol | -7.4 |
| 264 | RL323 | Thyrsiferyl 23-acetate | -7.4 |
| 265 | RL362 | (2aS,2bS,4R,4aR,6S,8bR,10R,10aS)-6-[(1R)-1-bromo-2-hydroxyethyl]-6,8b-dimethyl-1,2b,3,4,4a,5,6,7,8b,9,10,10a-dodecahydrocyclobuta[a]phenanthrene-2a,4,10(2H)-triol | -7.4 |
| 266 | RL371 | (2aR,2bR,4S,4aS,6R,8bS,10S,10aR)-6-[2-(acetyloxy)-1-bromoethyl]-2a-hydroxy-6,8b-dimethyl-1,2,2a,2b,3,4,4a,5,6,7,8b,9,10,10a-tetradecahydrocyclobuta[a]phenanthrene-4,10-diyl diacetate | -7.4 |
| 267 | RL375 | [(1R,5S,7R,8R,10S,11S,13R,14R)-14-(acetyloxy)-8- hydroxy-1,5-dimethyl-5-[(2S)-oxiran-2- yl] tetracyclo [8.5.0.0^ {2,7}.0^ {11,13}] pentadec-2- en-11-yl]methyl acetate | -7.4 |
| 268 | RL426 | (1S,3R,5S,6S,8S,9S,10R,13R)-1-acetoxy-3-bromo-6-hydroxy-8,13-epoxy-labd-14-ene | -7.4 |
| 269 | RL470 | 15-bromo-2,16-diacetoxyparguer-9(11)-en-7-ol | -7.4 |
| 270 | RL472 | 15-bromoparguer-7-en-16-ol | -7.4 |
| 271 | RR015 | 2-N-(2,3-Dibromo-4,5-dihydroxybenzyl)-9-&#946;-D-ribofuranosyldeoxyguanosine | -7.4 |
| 272 | RR027 | Rhodomevoidin | -7.4 |
| 273 | RR033 | 3-bromo-4,5-bis(2,3-dibromo-4,5-dihydroxybenzyl) pyrocatechol | -7.4 |
| 274 | BD011 | Dictyoepoxide | -7.3 |
| 275 | BD033 | Amijiol acetate | -7.3 |
| 276 | BD041 | 1,5-Cyclo-5,8,9,10-tetrahydroerogorgiaene | -7.3 |
| 277 | BD044 | (4S,8S,14R)-4-Hydroxy-1(15),17-dolastadiene | -7.3 |
| 278 | BD045 | (4S,8S,14R)-4-Hydroxy-1,17-dolastadiene | -7.3 |
| 279 | BD046 | (4S,8S)-4-Hydroxy-1(14),17-dolastadiene | -7.3 |
| 280 | BD064 | (6R,9aS,12S)-6,12-dihydroxy-3,7,9a-trimethyl-12-(propan-2-yl)-1H,2H,5H,6H,9H,9aH,10H,11H,12H,12aH-cyclopenta[11]annulen-2-one | -7.3 |
| 281 | BD074 | (6R,9aS,12S)-6,12-dihydroxy-3,7,9a-trimethyl-12-(propan-2-yl)-1H,2H,5H,6H,9H,9aH,10H,11H,12H,12aH-cyclopenta [11] annulen-2-one | -7.3 |
| 282 | BD076 | 9-acetoxydolabella-3,7,12-trien-16-oic acid | -7.3 |
| 283 | BL007 | rel-(4aS*,10aR*)- (+)-6,7-Dibromo-4a-hydroxy-3,8-dihydroxymethyl-10a-methoxy-1,4,4a,10a-tetrahydrodibenzo [b, e] [1,4] dioxin-1-one | -7.3 |
| 284 | BS004 | 5(R)-hydroxyspata 13,17-diene | -7.3 |
| 285 | BS018 | Plastoquinone | -7.3 |
| 286 | BS037 | Methyl 3-[(1S,4aR,5S,6S,8aR)-5-[(2-hydroxy-5-methoxy-3-methylphenyl) methyl]-5,6,8a-trimethyl-2-oxo-decahydronaphthalen-1-yl] propanoate | -7.3 |
| 287 | BS048 | 2-[(2E,6E,10E,14R)-14,15-dihydroxy-3,7,11,15-tetramethylhexadeca-2,6,10-trien-1-yl]-6-methylbenzene-1,4-diol | -7.3 |
| 288 | BS052 | Dihydroxysargaquinone | -7.3 |
| 289 | BS073 | Sargaol acetate | -7.3 |
| 290 | GU007 | ent-Labda-13(16),14-diene-3α-ol | -7.3 |
| 291 | comp53 | Cyanidine 3, 5 diglucoside | -7.3 |
| 292 | comp76 | Luteolin | -7.3 |
| 293 | comp74 | Riboflavin | -7.3 |
| 294 | RC024 | Callophycoic acid D | -7.3 |
| 295 | RJ004 | Parguerol-16-acetate | -7.3 |
| 296 | RL007 | Hydrocarbon | -7.3 |
| 297 | RL034 | Palisadin A | -7.3 |
| 298 | RL066 | Neoirietetraol | -7.3 |
| 299 | RL093 | Aplysistatin | -7.3 |
| 300 | RL095 | Aplysistatin | -7.3 |
| 301 | RL097 | Palisadin A | -7.3 |
| 302 | RL098 | Palisadin A | -7.3 |
| 303 | RL228 | 10-hydroxykahukuene B | -7.3 |
| 304 | RL250 | Indene-type sesquiterpene | -7.3 |
| 305 | RL304 | Palisadin A | -7.3 |
| 306 | RL306 | Aplysistatin | -7.3 |
| 307 | RL353 | Dibromophenol | -7.3 |
| 308 | RL354 | Dimer of Laurinterol | -7.3 |
| 309 | RL361 | 15-bromo-2, 16,19-triacetoxy-7-hydroxy-9(11)-paraguerene | -7.3 |
| 310 | RL368 | (2R)-2-[(1R,5S,7R,8R,10S,11S,13R,14R)-8,14- bis(acetyloxy)-11-[(acetyloxy)methyl]-1,5- dimethyltetracyclo[8.5.0.0^{2,7}.0^{11,13}]pentadec-2- en-5-yl]-2-bromoethyl acetate | -7.3 |
| 311 | RL372 | 2-[(1R,5S,7R,8R,10S,11S,13R,14R)-8,14- bis(acetyloxy)-11-[(acetyloxy)methyl]-1,5- dimethyltetracyclo[8.5.0.0^{2,7}.0^{11,13}]pentadec-2- en-5-yl]ethyl acetate | -7.3 |
| 312 | RL381 | [(1R,5S,7R,8R,10S,11S,13R,14R)-8,14-bis(acetyloxy)-5- [(1R)-1,2-dihydroxyethyl]-1,5- dimethyltetracyclo[8.5.0.0^{2,7}.0^{11,13}]pentadec-2- en-11-yl]methyl acetate | -7.3 |
| 313 | RL444 | Cupalaurenol | -7.3 |
| 314 | RL468 | 15-bromo-2,19-diacetoxyparguer-9(11)-en-7,16-diol | -7.3 |
| 315 | RL484 | Martiriol | -7.3 |
| 316 | RR014 | N-[3-bromo-2-(2,3-dibromo-4,5-dihydroxybenzyl)-4,5-dihydroxybenzyl] methyl pyroglutamate | -7.3 |
| 317 | BD007 | (1E,2R*,3R*,4S*,6E,18S*)-4,18-dihydroxydictyolactone | -7.2 |
| 318 | BD032 | Isopachydictyol A | -7.2 |
| 319 | BD035 | 8β-Hydroxypachydictyol A | -7.2 |
| 320 | BD038 | Dictyol C | -7.2 |
| 321 | BD058 | 17,18:18,19-bisepoxyxenic-methoxy-triene | -7.2 |
| 322 | BD065 | 9-Acetoxydolabella-3,7,12-trien-16-al | -7.2 |
| 323 | BS002 | 17,18-Epoxy, 5(R),16-dihydroxyspat 13(14)-ene | -7.2 |
| 324 | BS008 | 5(R),15,18(R/S), 19-tetrahydroxy spata  13,16-diene | -7.2 |
| 325 | BS027 | 2-Geranylgeranyl-6-methyl-1,4-benzoquinone | -7.2 |
| 326 | BS038 | Methyl 3-[(1S,4aR,5S,6S,8aR)-5-[(2,5-dihydroxy-3-methylphenyl) methyl]-5,6,8a-trimethyl-2-oxo-decahydronaphthalen-1-yl] propanoate | -7.2 |
| 327 | BS045 | Sargachromenol | -7.2 |
| 328 | BS046 | Sargahydroquinoic acid | -7.2 |
| 329 | BS083 | Nahocol D1 | -7.2 |
| 330 | BZ002 | (5Z,8Z,11Z,13E,17Z)-2'-eicosa-15(S)-hydroxy-5,8,11,13,17-pentaenoylphloroglucinol | -7.2 |
| 331 | comp29 | Brevifolin | -7.2 |
| 332 | RC025 | Callophycoic acid E | -7.2 |
| 333 | RC028 | Callophycoic acid H | -7.2 |
| 334 | RC029 | Callophycol A | -7.2 |
| 335 | RJ002 | Isoparguerol-16-acetate | -7.2 |
| 336 | RL003 | Cuparene Sesquiterpene | -7.2 |
| 337 | RL004 | Iodinated Sesquiterpene | -7.2 |
| 338 | RL033 | Aplysistatin | -7.2 |
| 339 | RL046 | 11-O-deacetylpinnaterpene C | -7.2 |
| 340 | RL051 | Luzonenone | -7.2 |
| 341 | RL110 | Majapolene B | -7.2 |
| 342 | RL151 | Prevezols A | -7.2 |
| 343 | RL247 | (+)-α-cadinol | -7.2 |
| 344 | RL263 | Acetylmajapolene A | -7.2 |
| 345 | RL265 | Majapolene A | -7.2 |
| 346 | RL364 | (3beta,5beta,7alpha,8alpha,10alpha,13alpha,15R)-15-bromo-3,19-cyclopimar-9(11)-ene-7,16-diol | -7.2 |
| 347 | RL367 | (2alpha,3beta,5beta,7alpha,8alpha,10alpha,13alpha,15R)-15-bromo-7,16-dihydroxy-3,19-cyclopimar-9(11)-en-2-yl acetate | -7.2 |
| 348 | RL373 | [(1R,5S,7R,8R,10S,11S,13R,14R)-8,14-bis(acetyloxy)- 1,5-dimethyl-5-[(2S)-oxiran-2- yl] tetracyclo [8.5.0.0^ {2,7}.0^ {11,13}] pentadec-2- en-11-yl] methyl acetate | -7.2 |
| 349 | RL459 | Majapolene A | -7.2 |
| 350 | RL467 | 15-bromo-2,7,16,19-tetraacetoxyparguer-9(11)-ene | -7.2 |
| 351 | RL469 | 15-bromo-2,16,19-triacetoxyparguer-9(11)-en-7-ol | -7.2 |
| 352 | RL513 | 15-Dehydroxythyrsenol A | -7.2 |
| 353 | RR003 | 2-(3-bromo-5-hydroxy-4-methoxyphenyl)-3-(2,3- dibromo-4,5-dihydroxyphenyl) propanoic acid | -7.2 |
| 354 | RR018 | methyl N′-[3-bromo-2-(2,3-dibromo-4,5-dihydroxybenzyl)-4,5-dihydroxybenzyl]-? -ureidobutyrate | -7.2 |
| 355 | RR019 | methyl 4- {[({3-bromo-2- [(2,3-dibromo-4,5- dihydroxyphenyl) methyl]-4,5- dihydroxyphenyl} methyl) [(2,3-dibromo-4,5- dihydroxyphenyl) methyl] carbamoyl] amino} butanoate | -7.2 |
| 356 | BD010 | 4α-hydroxycrenulatane | -7.1 |
| 357 | BD036 | Amijiol | -7.1 |
| 358 | BD051 | Acetylsanadaol | -7.1 |
| 359 | BD053 | 7(S*)-Acetoxy-14(S*)-dihydroxydolast-1(15), 8-diene | -7.1 |
| 360 | BL011 | 2,2′,3-tribromo-3′,4,4′,5-tetrahydroxy-6′-ethyl-oxymethyldiphenylmethane | -7.1 |
| 361 | BS011 | Spatol | -7.1 |
| 362 | BS015 | Plastoquinone | -7.1 |
| 363 | BS041 | (3R,6E,10E)-13-(6-hydroxy-2,8-dimethyl-2H-chromen-2-yl)-2,6,10-trimethyltrideca-6,10-diene-2,3-diol | -7.1 |
| 364 | BS065 | 2-(geranylgeranyl)-6-methyl-1,4-benzohydroquinone diacetate | -7.1 |
| 365 | BS088 | Methyl (5-hydroxy-2-{[(6E,13E)-12-hydroxy-3,7,11,15-tetramethyl-1,6,13,15-hexadecatetraen-3-yl]oxy}phenyl)acetate | -7.1 |
| 366 | BS093 | Sargahydroquinonic acid | -7.1 |
| 367 | comp19 | Anomurine | -7.1 |
| 368 | comp21 | Apigenin | -7.1 |
| 369 | comp67 | Genistein | -7.1 |
| 370 | comp2 | Quercetin | -7.1 |
| 371 | RC021 | Callophycoic acid A | -7.1 |
| 372 | RJ001 | Isoparguerol | -7.1 |
| 373 | RL021 | Laurinterol | -7.1 |
| 374 | RL025 | Debromofiliformin | -7.1 |
| 375 | RL026 | Allolaurinterol | -7.1 |
| 376 | RL027 | Debromoallolaurinterol | -7.1 |
| 377 | RL032 | Palisadin B | -7.1 |
| 378 | RL074 | 2-hydroxyluzofuranone B | -7.1 |
| 379 | RL092 | Palisadin B | -7.1 |
| 380 | RL094 | Palisadin B | -7.1 |
| 381 | RL096 | Palisadin B | -7.1 |
| 382 | RL132 | 16-Hydroxydehydrothyrsiferol | -7.1 |
| 383 | RL145 | Laurinterol | -7.1 |
| 384 | RL152 | Prevezols B | -7.1 |
| 385 | RL187 | Aldingenin A | -7.1 |
| 386 | RL262 | Acetylmajapolene B | -7.1 |
| 387 | RL279 | Laurinterol | -7.1 |
| 388 | RL303 | Palisadin B | -7.1 |
| 389 | RL328 | Laurefurenyne C | -7.1 |
| 390 | RL351 | Laurinterol | -7.1 |
| 391 | RL365 | (2alpha,3beta,5beta,7alpha,8alpha,10alpha,13alpha)-15-bromo-3,18-cyclopimar-9(11)-ene-2,7,16-triol | -7.1 |
| 392 | RL370 | Rogioldiol A | -7.1 |
| 393 | RL377 | (1R,5S,7R,8R,10S,11S,13R,14R)-8-(acetyloxy)-5- [(1R)-1-bromo-2-hydroxyethyl]-11-(hydroxymethyl)- 1,5-dimethyltetracyclo [8.5.0.0^ {2,7}.0^{11,13}]pentadec-2- en-14-yl acetate | -7.1 |
| 394 | RL378 | [(1R,5S,7R,8R,10S,11S,13R,14R)-14-(acetyloxy)-5- [(1R)-1-bromo-2-hydroxyethyl]-8-hydroxy-1,5- dimethyltetracyclo[8.5.0.0^{2,7}.0^{11,13}]pentadec-2- en-11-yl]methyl acetate | -7.1 |
| 395 | RL380 | (2beta,3alpha,7beta)-15-bromo-7,16,18-trihydroxy-3,19-cyclopimar-9(11)-en-2-yl acetate | -7.1 |
| 396 | RL400 | Laurinterol | -7.1 |
| 397 | RL412 | 3-epi-perforenone A | -7.1 |
| 398 | RL456 | Laurinterol | -7.1 |
| 399 | RL466 | 15-bromo-2,7,19-triacetoxyparguer-9(11)-en-16-ol | -7.1 |
| 400 | RL471 | 15-bromoparguer-9(11)-en-16-ol | -7.1 |
| 401 | RL499 | 3,3-bis(4,6-dibromo-2-methylsulfinyl) indole | -7.1 |
| 402 | BD003 | Dichotenone-B | -7 |
| 403 | BD020 | Hydroxyacetyldictyolal | -7 |
| 404 | BD031 | Pachydictyol A | -7 |
| 405 | BD062 | 5-acetoxy-12-hydroxydolabell-3,7Z-dienon | -7 |
| 406 | BD079 | 9-acetoxydolabella-3E,7E-dien-12-ol | -7 |
| 407 | BD081 | 5-acetoxy-12-hydroxydolabell-3,7E-dienon | -7 |
| 408 | BD083 | Pachydictyol a | -7 |
| 409 | BL010 | 2,2′,3,3′-tetrabromo-4,4′,5,5′-tetrahydroxydiphenyl-methane | -7 |
| 410 | BS005 | 5(R),18-dihydroxy spata13,16-diene | -7 |
| 411 | BS009 | 19-acetoxy, 5(R), 15,16-trihydroxy spata 13,17-diene | -7 |
| 412 | BS010 | 5(R), 17(S/R)-dihydroxy spata 13,18-diene | -7 |
| 413 | BS039 | methyl 3-[(1S,4aR,5S,6S,8aR)-5-[(2,5-dihydroxy-3-methylphenyl)methyl]-5,6,8a-trimethyl-2-(propan-2-ylidene)-decahydronaphthalen-1-yl]propanoate | -7 |
| 414 | GA001 | 5'-hydroxyisoavrainvilleol | -7 |
| 415 | GA008 | Avrainvilleol | -7 |
| 416 | comp37 | Catechin | -7 |
| 417 | comp42 | Chlorogenic acid | -7 |
| 418 | comp49 | Citroside A | -7 |
| 419 | comp61 | Epicatechin | -7 |
| 420 | comp78 | Malvidin | -7 |
| 421 | RC033 | Bromophycolide J | -7 |
| 422 | RL009 | (+)-α-Isobromo-cuparene | -7 |
| 423 | RL155 | Gomerone C | -7 |
| 424 | RL206 | Perforenone | -7 |
| 425 | RL218 | Johnstonol | -7 |
| 426 | RL260 | Bromocyclococanol | -7 |
| 427 | RL280 | Isolaurinterol | -7 |
| 428 | RL286 | Neorogioldiol B | -7 |
| 429 | RL298 | Kahukuene A | -7 |
| 430 | RL334 | Laurenditerpenol | -7 |
| 431 | RL339 | Enone | -7 |
| 432 | RL345 | Isolaurinterol | -7 |
| 433 | RL346 | Isolaurene | -7 |
| 434 | RL379 | (1R,5S,7R,8R,10S,11S,13R,14R)-8-hydroxy-11- (hydroxymethyl)-1,5-dimethyl-5-[(2S)-oxiran-2- yl] tetracyclo [8.5.0.0^ {2,7}.0^ {11,13}] pentadec-2- en-14-yl acetate | -7 |
| 435 | RL390 | Isorhodolaureol | -7 |
| 436 | RL443 | Aplysidiol | -7 |
| 437 | RL460 | Majapolene B | -7 |
| 438 | RL498 | 3,3'-bis(2'-methylsulfinyl-2-methylthio-4,6,4',6'-tetrabromo) indole | -7 |
| 439 | RO006 | Rhodomelol | -7 |
| 440 | RR005 | 3-(2,3-Dibromo-4,5-dihydroxyphenyl)-2-phenylpropanoic acid | -7 |
| 441 | RR044 | bis(2,3-dibromo-4,5-dihydroxybenzyl) ether | -7 |
| 442 | RR046 | 2,2',3,3'-tetrabromo-4,4',5,5'-tetrahydroxydiphenyl methane | -7 |
| 443 | RR049 | 4,4'-(oxydimethanediyl) bis(5,6-dibromobenzene-1,2-diol) | -7 |
