# Supplementary material for: Computational study of potential inhibitors for fat mass and obesity-associated protein from seaweed and plant compounds
Source: PeerJ. 2022 Oct 21;10:e14256. doi: 10.7717/peerj.14256 (PMC9590420; doi:10.7717/peerj.14256)
Supplement: Supplemental Information 5 — Class I: fatal if swallowed (LD50 ≤ 5); Class II: fatal if swallowed (5 < LD50 ≤ 50); Class III: toxic if swallowed (50 < LD50 ≤ 300); Class IV: harmful if swallowed (300 < LD50 ≤ 2,000); Class V: may be harmful if swallowed (2000 < LD50 ≤ 5,000); Class VI: non-toxic (LD50 > 5,000). [file peerj-10-14256-s005.docx]

| S.No. | Compound ID | Compound name | Toxicity Class | Predicted LD50 (mg/kg) |
| --- | --- | --- | --- | --- |
| 1 | BC009 | 1'-methoxycystoketal | Class VI | 5530 |
| 2 | BC010 | 4'-14-dimethoxyamentol | Class VI | 7500 |
| 3 | BC012 | 1',4',14-trimethoxyamentol | Class VI | 7500 |
| 4 | BC015 | (7S,11S,12S)-Cystoketal | Class VI | 5530 |
| 5 | BD020 | Hydroxyacetyldictyolal | Class VI | 6000 |
| 6 | BD061 | Dictyol H | Class VI | 37000 |
| 7 | BD064 | (6R,9aS,12S)-6,12-dihydroxy-3,7,9a-trimethyl-12-(propan-2-yl)-1H,2H,5H,6H,9H,9aH,10H,11H,12H,12aH-cyclopenta [11 ]annulen-2-one | Class VI | 9000 |
| 8 | BD074 | (6R,9aS,12S)-6,12-dihydroxy-3,7,9a-trimethyl-12-(propan-2-yl)-1H,2H,5H,6H,9H,9aH,10H,11H,12H,12aH-cyclopenta [11] annulen-2-one | Class VI | 9000 |
| 9 | BS069 | Stypodiol diacetate | Class VI | 7000 |
| 10 | BT008 | 24ε-hydroperoxy-24-ethylcholesta-4,28(29)-dien-3-one | Class VI | 5010 |
| 11 | BT010 | 24ε-hydroperoxy-24-ethylcholesta-4,28(29)-dien-3,6-dione | Class VI | 5010 |
| 12 | BT011 | 6β-hydroxy-24-ethylcholesta-4,24(28)-dien-3-one | Class VI | 5010 |
| 13 | BT012 | 24ε-hydroperoxy-6β-hydroxy-24-ethylcholesta-4,- 28(29)-dien-3-one | Class VI | 5010 |
| 14 | comp74 | Riboflavin | Class VI | 10000 |
| 15 | comp37 | Catechin | Class VI | 10000 |
| 16 | comp61 | epicatechin | Class VI | 10000 |
| 17 | comp62 | epigallocatechin | Class VI | 10000 |
| 18 | comp66 | Gallocatechin | Class VI | 10000 |
| 19 | RG008 | 24ε-hydroperoxy-6β-hydroxycholesta-4,25-dien-3-one | Class VI | 5010 |
| 20 | RG009 | 25-hydroperoxy-6β-hydroxycholesta-4,23(E)-dien-3-one | Class VI | 5010 |
| 21 | RG010 | 24ε-hydroperoxycholesta-4,25-diene-3,6-dione | Class VI | 5010 |
| 22 | RG011 | 25-hydroperoxycholesta-4,23(E)-diene-3,6-dione | Class VI | 5010 |
| 23 | RG012 | 6β,24ε-Dihydroxycholesta-4,25-dien-3-onerg | Class VI | 5010 |
| 24 | RG013 | 6β,25-Dihydroxycholesta-4,23-dien-3-one | Class VI | 5010 |
| 25 | RL009 | (+)-α-Isobromo-cuparene | Class VI | 6430 |
| 26 | RL074 | 2-hydroxyluzofuranone B | Class VI | 25000 |
| 27 | RL261 | Tiomanene | Class VI | 6060 |
| 28 | RL300 | Labdane type brominated diterpene | Class VI | 7800 |
| 29 | RL328 | Laurefurenyne C | Class VI | 39800 |
| 30 | RL442 | 10-acetoxyangasiol | Class VI | 50100 |
