# Supplementary material for: Computational study of potential inhibitors for fat mass and obesity-associated protein from seaweed and plant compounds
Source: PeerJ. 2022 Oct 21;10:e14256. doi: 10.7717/peerj.14256 (PMC9590420; doi:10.7717/peerj.14256)
Supplement: Supplemental Information 6 [file peerj-10-14256-s006.docx]

| Compound ID | Compound Name | Docking Score | H- Bond Interaction | Hydrophobic Interaction | Pi Interaction |
| --- | --- | --- | --- | --- | --- |
| BT012 | 24ε-hydroperoxy-6β-hydroxy-24-ethylcholesta-4,- 28(29)-dien-3-one | -9.12 | Thr92, Tyr106, Glu234 | Pro93, Tyr108, Leu109, Al228, His231 | - |
| BD064 | (6R,9aS,12S)-6,12-dihydroxy-3,7,9a-trimethyl-12-(propan-2-yl)-1H,2H,5H,6H,9H,9aH,10H,11H,12H,12aH-cyclopenta [11] annulen-2-one | -8.41 | Pro93, Tyr106, His231, Glu234 | Val94, Val228 | His231 |
| RL442 | 10-acetoxyangasiol | -8.01 | Pro93, Lys216, Ser229 | Tyr108, Leu109 | - |
| RL074 | 2-hydroxyluzofuranone B | -7.5 | Pro93, Asp233, Glu234 | Val94, Val228 | His231 |
| BC012 | 1',4',14-trimethoxyamentol | -7.43 | Asn205, Ser 229 | Thr92, Pro93, Val94, Tyr108, Leu109, Val28, His231, Val244, Val309, Thr320 | - |
| BT011 | 6β-hydroxy-24-ethylcholesta-4,24(28)-dien-3-one | -7.41 | Lys216 | Pro93, Leu109, Leu215, Lys216, Val28, His231 | - |
| RG010 | 24ε-hydroperoxycholesta-4,25-diene-3,6-dione | -7.35 | Lys216, Ser229 | Thr92, Pro93, Tyr108, Leu109, Lys216, Val228, His231 | - |
| RG009 | 25-hydroperoxy-6β-hydroxycholesta-4,23(E)-dien-3-one | -7.3 | Lys216, Ser229 | Tyr108, Leu109, Leu215, Val228 | - |
| RG013 | 6β,25-Dihydroxycholesta-4,23-dien-3-one | -7.28 | Lys216, Ser229 | Tyr108, Leu 109, Leu 215, Val228, His231 | - |
| BT010 | 24ε-hydroperoxy-24-ethylcholesta-4,28(29)-dien-3,6-dione | -7.25 | Val94, Lys216, | Pro93, Leu109, Lys216 |  |
| RG011 | 25-hydroperoxycholesta-4,23(E)-diene-3,6-dione | -7.2 | Arg96, Tyr108 | Pro 93, Tyr214, Leu215, Lys216, Val228 | - |
| BC015 | (7S,11S,12S)-Cystoketal | -7.2 | Tyr214, Lys216, | Thr 92, Leu109, Leu215, Val228 | - |
| RG008 | 24ε-hydroperoxy-6β-hydroxycholesta-4,25-dien-3-one | -7.15 | Ser229, His232 | Leu109, Lys216, Val228, His231 | - |
| BT008 | 24ε-hydroperoxy-24-ethylcholesta-4,28(29)-dien-3-one | -7.13 | Lys216, His232 | Pro93, Leu109, Lys216, Val228 | - |
| BS069 | Stypodiol diacetate | -7.13 | Gln86, Val94 | Ile85, Val94, Tyr108, Leu109, Val228, His231 | - |
| BC009 | 1'-methoxycystoketal | -7.1 | Tyr108 | Ile85, Leu90, Val94, Leu109, Val228, His231 | - |
| RL300 | Labdane type brominated diterpene | -7.09 | Lys300 | Thr92, Pro93, Leu109, Leu215, Lys216 | - |
| BC010 | 4'-14-dimethoxyamentol | -6.64 | Ser229 | Thr92, Pro93, Leu109, Val228, His231 | - |
| RL261 | Tiomanene | -6.6 | - | Pro93, Tyr108, Val 228 | - |
| BD061 | Dictyol H | -6.46 | Lys216 | Ile85, Pro93, Leu109, Leu215, His231 | - |
| RG012 | 6β,24ε-Dihydroxycholesta-4,25-dien-3-one | -6.41 | Tyr108, Tyr214 | Pro93, Leu106, Tyr214, Lys216, Val228 | - |
| comp61 | epicatechin | -6.36 | Val94, Arg96, | Tyr108, Val228 | - |
| BD074 | (6R,9aS,12S)-6,12-dihydroxy-3,7,9a-trimethyl-12-(propan-2-yl)-1H,2H,5H,6H,9H,9aH,10H,11H,12H,12aH-cyclopenta[11]annulen-2-one | -6.34 | His73, Leu78, | Leu78, Phe79, Arg80, Phe206 | - |
| comp62 | epigallocatechin | -6.27 | Val94, Tyr106, | Leu109, Val228, His231, Glu234 | - |
| comp66 | Gallocatechin | -6.23 | Val94, | Leu108, Val228, His231, Glu234 | Tyr108 |
| comp37 | Catechin | -6.05 | Ala227, Ser229, His232, Glu234 | Tyr108, Leu109, His231, Glu234 | His231 |
| RL009 | (+)-α-Isobromo-cuparene | -5.31 | - | Thr 92, Pro93, Val 94, Leu109, Lys 216 | - |
| comp108 | Riboflavin | -4.89 | Arg96 | Leu109, His231 | His231 |
| BD020 | Hydroxyacetyldictyolal | -4.72 | Leu91 | Ile85, Leu90, Pro93, Leu109 | - |
| RL328 | Laurefurenyne C | -4.59 | Ser229 | Thr92, Pro93, Lys216, Val228 | - |
